# *Clostridioides difficile* 630Δ*erm in silico* and *in vivo* – quantitative growth and extensive polysaccharide secretion

**DOI:** 10.1002/2211-5463.12208

**Published:** 2017-03-09

**Authors:** Henning Dannheim, Sabine E. Will, Dietmar Schomburg, Meina Neumann‐Schaal

**Affiliations:** ^1^ Braunschweig Integrated Centre of Systems Biology (BRICS)Department of Bioinformatics and BiochemistryTechnische Universität BraunschweigBraunschweigGermany

**Keywords:** *Clostridioides difficile*, *Clostridium difficile*, exopolysaccharide, genome‐scale, metabolic modeling, metabolism

## Abstract

Antibiotic‐associated infections with *Clostridioides difficile* are a severe and often lethal risk for hospitalized patients, and can also affect populations without these classical risk factors. For a rational design of therapeutical concepts, a better knowledge of the metabolism of the pathogen is crucial. Metabolic modeling can provide a simulation of quantitative growth and usage of metabolic pathways, leading to a deeper understanding of the organism. Here, we present an elaborate genome‐scale metabolic model of *C. difficile* 630Δ*erm*. The model *i*HD992 includes experimentally determined product and substrate uptake rates and is able to simulate the energy metabolism and quantitative growth of *C. difficile*. Dynamic flux balance analysis was used for time‐resolved simulations of the quantitative growth in two different media. The model predicts oxidative Stickland reactions and glucose degradation as main sources of energy, while the resulting reduction potential is mostly used for acetogenesis via the Wood–Ljungdahl pathway. Initial modeling experiments did not reproduce the observed growth behavior before the production of large quantities of a previously unknown polysaccharide was detected. Combined genome analysis and laboratory experiments indicated that the polysaccharide is an acetylated glucose polymer. Time‐resolved simulations showed that polysaccharide secretion was coupled to growth even during unstable glucose uptake in minimal medium. This is accomplished by metabolic shifts between active glycolysis and gluconeogenesis which were also observed in laboratory experiments.

**Abbreviations**
CDMM: *C. difficile* defined minimal medium
DFBA: dynamic flux balance analysis
FBA: flux balance analysis
GAM: growth‐associated maintenance
MDM: minimal defined medium
NGAM: nongrowth‐associated maintenance
OD: optical density at 600 nm
TCA: tricarboxylic acid

*Clostridioides difficile* (previously *Clostridium difficile* 1, 2) is a rod‐shaped, Gram‐positive, spore‐forming bacterium of the Clostridiales order and represents a major cause of infectious diarrhea developed after antibiotic treatment during hospitalization. The symptoms of *C. difficile* infection can range from mild diarrhea to pseudomembraneous colitis and toxic megacolon, bowel perforation, and sepsis 3. *Clostridioides difficile* can carry two large clostridial toxins named toxin A and toxin B as well as an actin‐specific ADP‐ribosyltransferase better known as binary toxin 4. Strains with the binary toxin but deficient for the other toxins are still virulent 5. Nevertheless, the toxins A and B are considered to be the main virulence factors 6. The production of the large toxins is known to be regulatory connected to the metabolism and strongly environment‐dependent 7, 8, 9, 10. This connection between metabolism and virulence illustrates the need of a deep understanding of the metabolism. The metabolism of *C. difficile* is strongly dependent on Stickland reactions, which involve the coupled oxidation and reduction of amino acids to short‐chain organic acids 11. Figure 1 shows the leucine degradation pathways in *C. difficile*, which are classical examples for oxidative and reductive Stickland pathways.

**Figure 1:**
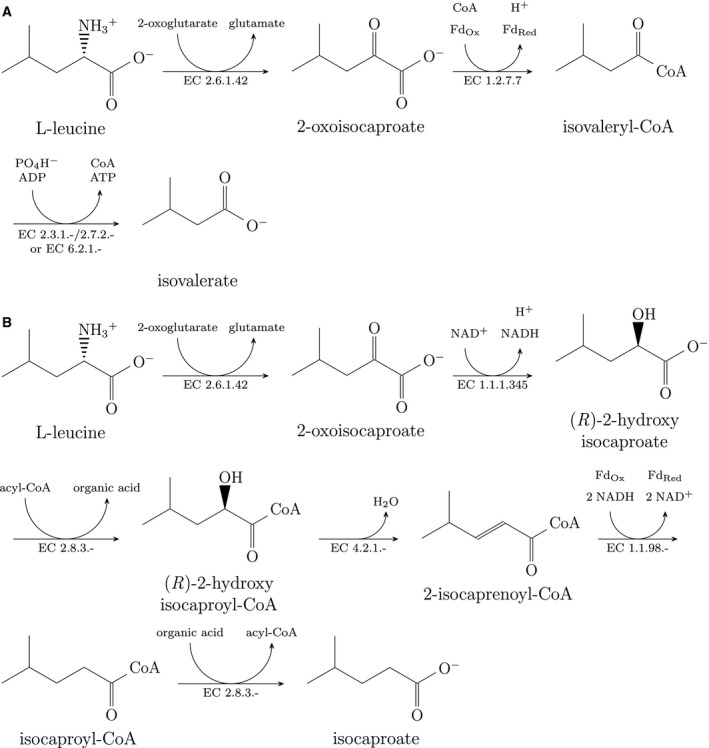
Leucine degradation in *C. difficile*, an example for classical Stickland degradation 65. (A) Oxidative Stickland pathway. (B) Reductive Stickland pathway. Electron bifurcation of the 2‐isocaprenoyl‐CoA hydrogenase was assumed based on homology 32, 33, 34.

As the metabolism is based on the genome, the first sequencing of a strain in 2006 12 opened new ways to characterize *C. difficile*. The erythromycin‐sensitive mutant strain 630Δ*erm* 13 was sequenced in 2014 14 and is currently one of the most popular research strains of *C. difficile*. Recently, we published time‐resolved exometabolome data 10 and the resequencing and reannotation of this strain 15. Due to the availability of these data, strain 630Δ*erm* is the perfect candidate for metabolic modeling.

Genome‐scale metabolic modeling can be used for a wide range of predictions. It can help to interpret experimental data or predict nutritional requirements, quantitative growth performance, mutant growth, and even proton transport stoichiometry 16. We used the so called constraint‐based stoichiometric flux balance analysis (FBA). In principle, the metabolic concentrations and fluxes can be mathematically described by ordinary differential equations describing the production and consumption rates of all metabolites by accurate stoichiometric reaction equations. Assuming – at least for a short time – a flux equilibrium where concentrations do not change as long as outer conditions or the protein repertoire is not changing, this reduces to a linear equation system. As there are more fluxes than metabolites in the cell, this equation system is underdetermined and can be solved by introducing constraints and applying biological knowledge about optimized use of nutrients during evolution. So, in most cases that combination of fluxes is calculated that maximizes the biomass production.

The prediction of quantitative growth in batch cultures has been done previously for *Escherichia coli* in a glucose minimal medium using dynamic flux balance analysis (DFBA) 17, 18. Using experimentally acquired maximum uptake rates for glucose and oxygen, the simulations reproduced the experimental results quite accurately with the growth either limited by oxygen (aerobic scenario) or by the sole carbon source glucose (anaerobic scenario). The anaerobic growth in combination with the need of several carbon sources prohibits an approach based on maximum uptake rates for *C. difficile*. An initial metabolic model of *C. difficile* 630 was published by Larocque *et al*. 19 and used to predict essential genes by computational knockout analysis to find drug targets 19.

Here, we present an improved and elaborate genome‐scale metabolic model *i*HD992 of *C. difficile* 630Δ*erm*, which consist of 992 genes, 786 reactions, 936 metabolites, and 163 transport reactions. It includes time‐resolved substrate uptake and biomass production rates, derived from laboratory data, as well as the production and secretion of a previously undescribed polysaccharide. The identity of the polysaccharide is determined based on combined bioinformatical analysis and laboratory experiments. In addition, DFBA is used for quantitative and time‐resolved simulation of batch cultures in minimal defined medium (MDM) and *C. difficile* defined minimal medium (CDMM). Both media consist of glucose as well as the same salts and buffers. MDM also contains seven amino acids strictly necessary for growth (cysteine, methionine, proline, leucine, isoleucine, valine, and tryptophan), while CDMM is a richer medium that includes casamino acids, cysteine, and tryptophan 10. The shifts between glycolysis and gluconeogenesis in MDM, experimentally shown with isotope‐labeled glucose, are explained based on modeling results. Finally, the importance of the one‐carbon metabolism as well as the fragmented tricarboxylic acid (TCA) cycle are discussed in detail.

## Results and Discussion

### The metabolic model *i*HD992

The model *i*HD992 of *C. difficile* 630Δ*erm* consists of 992 genes, 786 reactions, 936 metabolites, and 163 transport reactions. The model is included in the Supporting information (Model S1 and the exemplary Scenario S1). In comparison to the published model *i*MLTC806cdf of *C. difficile* 630 19, our model contains more reactions, metabolites, and genes (Table 1). These include mainly pathways specific for *C. difficile* (e.g., bile acid metabolism).

**Table 1:** Comparison of the *C. difficile* models *i*HD992 and *i*MLTC806cdf

Model	*i*HD992	*i*MLTC806cdf 19
Strain	630Δ*erm*	630
Metabolic reactions	786	760
Not sequence‐based	44	149
Spontaneous	20	8
Nonspontaneous	24	141
Transport reactions	163	145
Exchange reactions	161	171
Biomass reactions	15	9
Metabolites	936	704
Genes	992	806

Genome‐scale metabolic models contain reactions based on genome annotations, spontaneous reactions, and reactions neither based on the genome nor known to be spontaneous. While reactions in the first two groups form the core of each model, the number of reactions in the latter group should be low. These reactions are usually added to the model to close gaps in the metabolic pathways which are known or assumed to be functional in the organism. For example, this is the case for described pathways whose enzymes are not yet identified. In our model, we could decrease the number of these reactions compared to *i*MLTC806cdf by 83%, representing a significant improvement.

The two *C. difficile* models are significantly different as they share only 508 metabolic reactions (67% of *i*MLTC806cdf), while the model by Larocque *et al*. has 385 (51%) in common with the *Clostridium acetobutylicum* model *i*CAC490 19, 20. Of the 262 other metabolic reactions from *i*MLTC806cdf, 111 are not existing in *i*HD992 as they are not sequence based reactions and no other indication could be found for their existence in *C. difficile*. From the 151 sequence‐based reactions in the model from Larocque *et al*. not existing in *i*HD992, 140 are not in *i*HD992 because of differences in enzyme function predictions, six were excluded from *i*HD992 as they are metabolically cheaper side reactions compared to the main reactions of the enzymes and five were excluded as these would lead to the cost‐free reduction of NADP^+^ with NADH.

The published model *i*MLTC806cdf 19 was tested in an exemplary MDM growth scenario with the substrate consumption rates derived from our experiment at 3 h of cultivation and biomass production as the objective function. The model permits cost‐free energy generation (e.g., free secretion of protons in combination with the ATP synthase). The growth is nevertheless limited by the l‐leucine uptake as the model predicts *C. difficile* to be leucine auxotroph. The resulting predicted growth rate of 7.12 h^−1^ is significantly higher as the experimentally determined 0.414 h^−1^. This finding indicates the inability of the model *i*MLTC806cdf to describe the energy metabolism and quantitative growth of *C. difficile*.

### The incomplete tricarboxylic acid cycle

**Figure 2:**
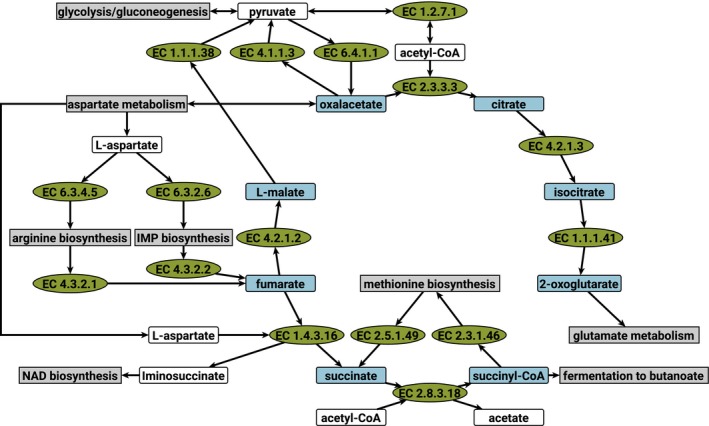
The TCA cycle of *C. difficile*. For simplification not all substrates and products are shown. The direction of the arrows represents the flux direction in *C. difficile*. Blue: metabolites of the TCA, white: other metabolites, green: reactions, gray: connected pathways.

Based on our genome annotation, *C. difficile* 630Δ*erm* has a fragmented TCA cycle (Fig. 2), which is still capable to produce necessary biomass precursors and degrade side products of biosynthesis, but no direct connection is existent between 2‐oxoglutarate and succinyl‐CoA. The reductive direction from oxaloacetate to L‐malate is also blocked as no nondecarboxylating L‐malate dehydrogenase (like EC 1.1.1.37) could be identified in *C. difficile*. The metabolites from L‐malate to succinyl‐CoA are nevertheless connected to the metabolism via L‐aspartate. It serves as ammonium donor for the arginine and IMP biosyntheses and is converted to fumarate. Fumarate can be degraded to pyruvate by the fumarate hydratase (EC 4.2.1.2) and malate dehydrogenase (oxaloacetate‐decarboxylating) (EC 1.1.1.38) or used as electron acceptor in the oxidation of L‐aspartate to produce iminosuccinate for the NAD^+^ biosynthesis. The resulting succinate fills the intracellular succinate/succinyl‐CoA pool for the methionine biosynthesis. Succinate can be recycled by the succinyl‐CoA:acetate CoA‐transferase (EC 2.8.3.18) or degraded to butanoate. A similar TCA cycle was previously described for the close relative *C. acetobutylicum* 21. Nevertheless, the TCA cycles of both organisms are different as no candidate enzyme for the transfer of Coenzyme A to succinate could be found in *C. acetobutylicum*. In addition, this organism has putative genes for malate dehydrogenase (EC 1.1.1.37) and 2‐oxoglutarate synthase (EC 1.2.7.3) 21, 22, 23, which are the missing enzymes in *C. difficile* to close the TCA cycle.

### Growth on ^13^C‐labeled glucose – gluconeogenesis is on despite glucose consumption

**Figure 3:**
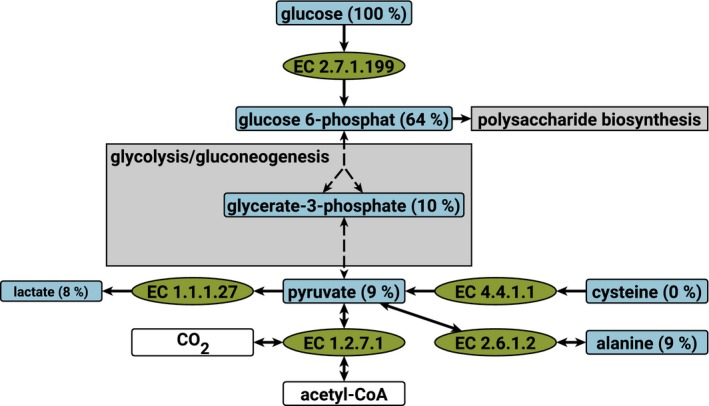
Origin of carbon atoms in the central metabolism. Abundances of isotope labeling species at an OD of 0.220 with complete ^13^C‐labeled glucose in MDM. Blue: metabolites with determined isotope labeling. The abundance of the full ^13^C‐labeled species is given brackets, white: other metabolites, green: reactions, gray: connected pathways.

The model predicts only glucose and cysteine as carbon sources usable for biomass production in MDM, whereas the other six amino acids are only used for energy generation via Stickland reactions or directly for protein synthesis. To study the carbon metabolism of *C. difficile* in detail, the organism was grown in naturally labeled MDM with completely ^13^C‐labeled glucose. The results of GC/MS analysis of some intracellular metabolites in samples taken at optical density (OD) 0.220 are shown in Fig. 3 (see Table S1 for details). All detected metabolites along the connection between cysteine and glucose were found either in the completely labeled or the completely unlabeled form. This indicates a recent switch between glycolysis and gluconeogenesis during cultivation as under stable conditions, the model predicts that the metabolites between glucose and pyruvate are derived either from glucose or from cysteine. The metabolites can be divided into two groups. Glucose 6‐phosphate is 64% labeled and of the glycerate 3‐phosphate, pyruvate, lactate, and alanine only about 9% are labeled. This indicates that the carbon of the central metabolism and the glucose are merged at glucose 6‐phosphate with about 40% derived from gluconeogenesis. The model predicts glucose 6‐phosphate only degradable via glycolysis, suggesting that the demand of activated sugars is not satisfied by the glucose import at the time of sampling.

### *Clostridioides difficile* secretes an acetylated glucose polymer

Initial modeling experiments using the experimental metabolite import and export rates predicted a significant higher growth rate (3.59 h^−1^ instead of 0.414 h^−1^ at 3 h of growth) compared to the experiment. In addition, large amounts of carbon taken up by the organism were not found in the known products or biomass. Due to the viscous flow behavior of the culture supernatant and the temporarily active gluconeogenesis (see previous subsection), we suspected the formation of a polysaccharide. *Clostridioides difficile* possesses an operon putatively involved in the synthesis of a polysaccharide. The operon consists of genes encoding a putative membrane‐bound O‐acyl transferase (EC 2.3.1.‐), a cellulose synthase (EC 2.4.1.12), an endoglucanase (EC 3.2.1.4), and two membrane proteins of unknown function. We assumed the gene cluster to encode for enzymes responsible for the biosynthesis of acetylated glucose polymer (putatively acetylated cellulose).

To test this hypothesis, glucose and acetate concentration of the culture supernatant were measured prior and after acidic (for the depolymerization) and alkaline (for acetate) hydrolysis. Table 3 shows that most of the consumed glucose in both media is converted to a polysaccharide consisting of acetylated glucose subunits. During cultivation in MDM, a constant 73% share of the consumed glucose was converted to polysaccharide. In CDMM, which contains additional amino acids compared to MDM, the high glucose uptake and polysaccharide synthesis were delayed and the final ratio of synthesized polysaccharide to glucose intake was only 40%. In both media, no degradation of the polysaccharide by *C. difficile* could be detected and approximately 0.5 mol acetate per mol polysaccharide glucose was detected after alkaline hydrolysis. These findings support the structure indicated by the genome.

In agreement with the existence of a similar operon (Table 2) in its genome, an acetylated polysaccharide has been observed in cultures of *C. acetobutylicum* 24. In contrast to *C. difficile*,* C. acetobutylicum* is able to degrade its polysaccharide 24 and possesses a cellulosome. As *C. acetobutylicum* is unable to grow on cellulose 25 and its cellulosome does not degrade crystalline cellulose 26, its cellulosome is probably optimized to degrade the secreted polysaccharide.

**Table 2:** Polysacharide operons of *C. difficile* and *C. acetobutylicum*

Product	Locus		Identity (%)	Query coverage (%)
	*C. difficile*	*C. acetobutylicum*		
Membrane protein	CDIF630erm_02794	CA_C1565	51	93
Putative membrane‐bound O‐acyl transferase	CDIF630erm_02795	CA_C1564	61	100
Membrane protein	CDIF630erm_02796	CA_C1562	27	90
Cellulose synthase	CDIF630erm_02797	CA_C1561	53	95
Endoglucanase	CDIF630erm_02798	CA_C1563	46	17

### The growth‐associated polysaccharide secretion in MDM

**Figure 4:**
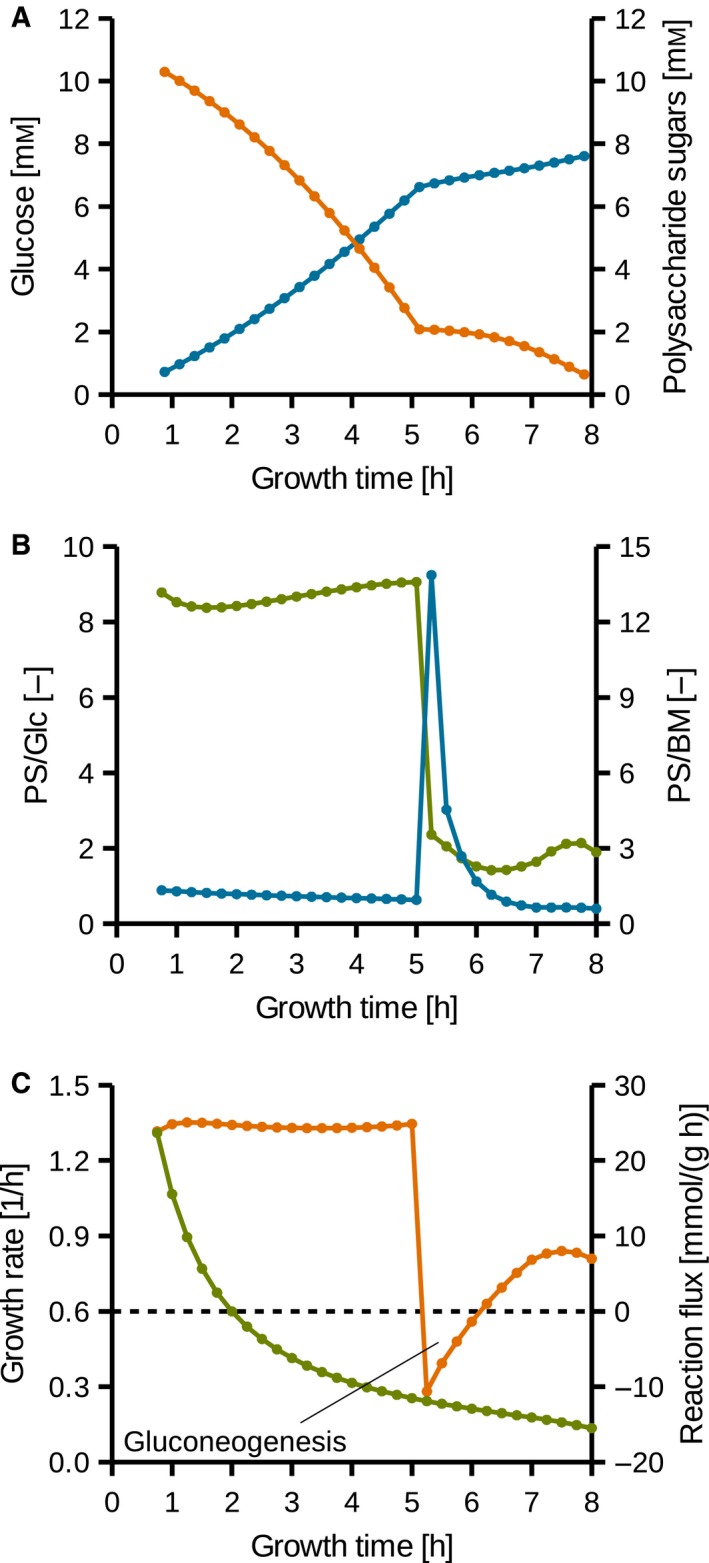
Growth of *C. difficile* 630Δ*erm* in MDM. (A) Orange: glucose concentration used for the simulations, blue: predicted exopolysaccharide concentration. (B) Blue: predicted ratio of polysaccharide sugar secretion rate and glucose uptake rate (PS/Glc), green: predicted ratio of polysaccharide carbon secretion rate and biomass carbon production rate (PS/BM). (C) Green: growth rate based on OD, orange: predicted reaction flux catalyzed by the phosphoglycerate kinase (EC 2.7.2.3) per biomass.

The availability of time‐resolved cultivation data of *C. difficile* 630Δ*erm* 10 allows to use time‐resolved FBA to investigate the metabolic state in more detail. As the available growth data are more precise than the polysaccharide production data, the simulations for the growth were optimized for the synthesis of the polysaccharide and the growth rate was set to growth rates derived from literature 10. Figure 4A shows the glucose as well as the exopolysaccharide in the culture broth during cultivation. The majority of the glucose is converted to the exopolysaccharide. The model predicts 76% of the glucose uptake converted to polysaccharide until an OD at 600 nm of 0.15 is reached. This is close to the measured polysaccharide/glucose ratio determined in the laboratory experiments (Table 3). At an OD of 0.22, 81% of the consumed glucose is predicted to be converted to polysaccharide, which is slightly higher than experimentally determined.

**Table 3:** Polysaccharide formation in MDM and CDMM. The initial glucose concentration was determined to be 11.48 ± 0.10 mm in MDM and 11.68 ± 0.14 mm in CDMM

Medium	MDM		
Growth phase	Early	Late	Stationary
OD (600 nm)	0.151	0.218	0.377
Glucose prior hydrolysis [mm]	5.16 ± 0.19	2.10 ± 0.16	0.24 ± 0.08
Glucose after hydrolysis [mm]	9.82 ± 0.16	8.88 ± 0.16	8.54 ± 0.19
Acetate prior hydrolysis [mm]	1.36 ± 0.16	2.14 ± 0.06	5.88 ± 0.27
Acetate after hydrolysis [mm]	3.87 ± 0.09	5.06 ± 0.07	9.18 ± 0.09
Glucose (bound/total)	0.73 ± 0.05	0.72 ± 0.03	0.74 ± 0.02
Acetylations per bound glucose	0.54 ± 0.05	0.43 ± 0.02	0.39 ± 0.04

Medium	CDMM		
Growth phase	Early	Late	Stationary
OD (600 nm)	0.326	0.725	1.169
Glucose prior hydrolysis [mm]	11.47 ± 0.26	3.05 ± 0.12	0.07 ± 0.14
Glucose after hydrolysis [mm]	11.49 ± 0.31	6.13 ± 0.19	4.70 ± 0.50
Acetate prior hydrolysis [mm]	2.37 ± 0.05	14.01 ± 0.41	18.89 ± 0.24
Acetate after hydrolysis [mm]	2.35 ± 0.11	15.88 ± 0.33	21.05 ± 0.26
Glucose (bound/total)	–	0.36 ± 0.03	0.40 ± 0.05
Acetylations per bound glucose	–	0.61 ± 0.18	0.47 ± 0.09

Figure 4B shows the ratios of polysaccharide secretion compared to glucose uptake and biomass production in MDM during the linear growth phase, where the increase of biomass is constant over time. The model predicts a linear decrease of the ratio of produced polysaccharide sugar to glucose intake of 89% (at 0.75 h) to 40% (at 8 h). This is temporarily interrupted by a phase when more glucose is used for polysaccharide synthesis than imported (Fig. 4). Here, a switch from glycolysis to gluconeogenesis was observed both in laboratory experiment and simulation. As mentioned above, the amount of glucose 6‐phosphate derived from gluconeogenesis is about 40% at OD 0.220 based on the laboratory experiment. The model simulates this also quite accurately as 45% of glucose 6‐phosphate was derived from gluconeogenesis at 5.75 h (OD 0.22).

The carbon ratio of the polysaccharide subunit secretion rate and biomass production rate predicted by the model (Fig. 4B) stays constant at ~13.1 mol_C_·mol_C_^−1^ until 5 h of growth. After the subsequent change of metabolism, the ratio stays constant at ~2.6 mol_C_·mol_C_^−1^ until 8 h of growth. Constant ratios represent a growth‐associated product formation which indicates in turn the formation of a capsule or biofilm. In fact, capsule 27 and biofilm 28 formation have already been observed in different strains of *C. difficile*. We observed stirring and shaking as growth inhibiting effects for *C. difficile* cultures in our laboratory (data not shown). The growth inhibiting effect can be explained by the capsule formation, as detached polysaccharide is replaced rather than new biomass produced and *C. difficile* capsules were reported to be fragile 27.

### The glycolysis reaction fluxes are constant during linear growth in MDM

**Figure 5:**
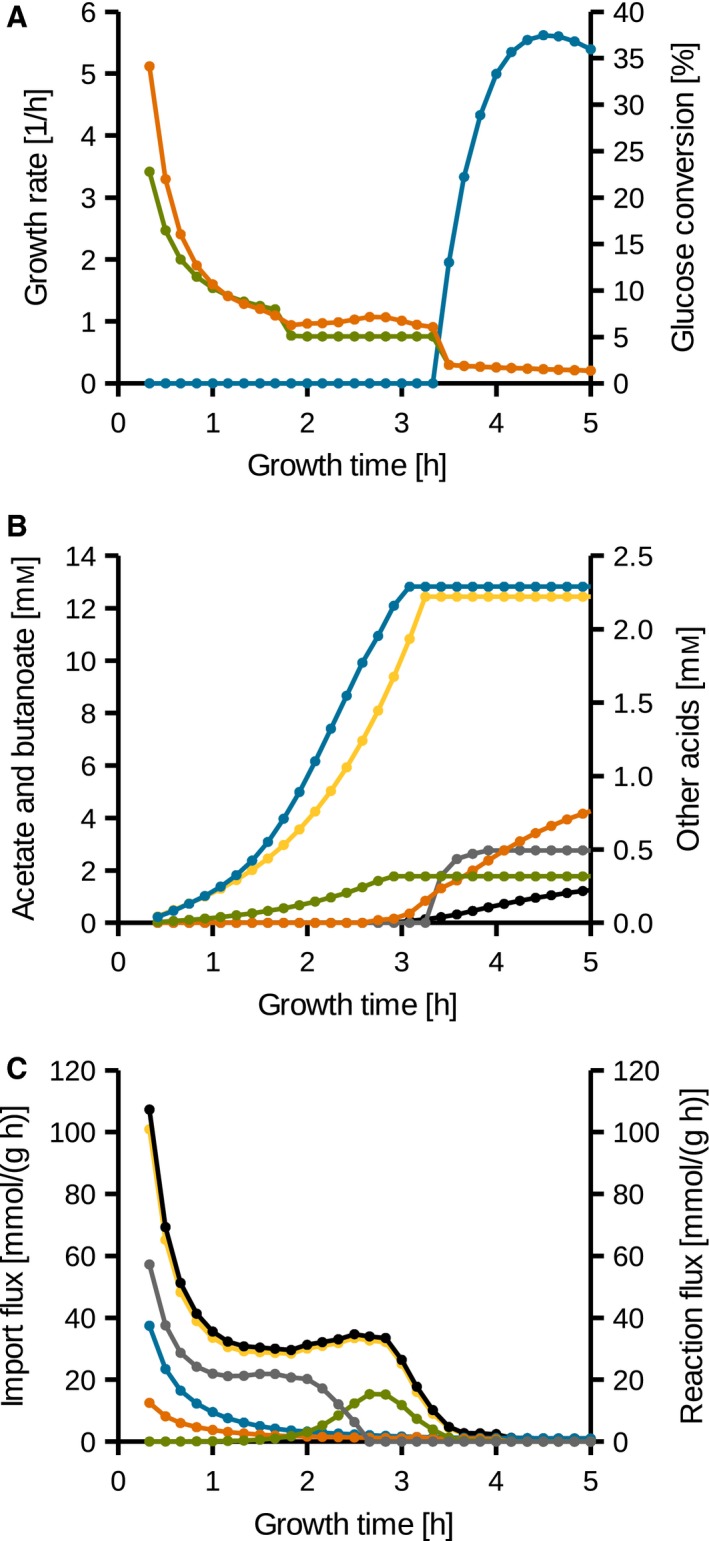
Simulation results of *C. difficile* 630Δ*erm* growth in CDMM. (A) Green: growth rate based on OD, orange: predicted growth rate, blue: predicted glucose converted to secreted polysaccharide per total consumed glucose. (B) Predicted side products concentrations over time. For simplification, side product secretion prior 0.4 h was neglected. Blue: acetate, green: propanoate, orange: butanoate, yellow: isovalerate, gray: isocaproate, black: pentanoate. (C) Fluxes related to reductive acetyl‐CoA biosynthesis per biomass. Amino acid uptake rates are derived from laboratory experiment. Yellow: predicted activity of the acetyl‐CoA synthase complex (EC 2.3.1.169), green: serine uptake, blue: threonine uptake, orange: glycine uptake, gray: predicted activity of the methylenetetrahydrofolate dehydrogenase (EC 1.5.1.5), black: theoretical one‐carbon availability based on amino acid uptake and methylenetetrahydrofolate dehydrogenase activity.

The predicted activity of phosphoglycerate kinase (EC 2.7.2.3) is shown exemplarily in Fig. 4C. The flux remains constant at about 24.5 mmol·(g_Biomass_ h)^−1^ until 5 h of growth, drops below zero for about an hour when the reaction flux is inverted during gluconeogenesis activity and slowly rises again to about 8.0 mmol·(g_Biomass_ h)^−1^ until 7.5 h of growth. During unrestricted exponential growth, all fluxes stay constant over time. In contrast, *C. difficile* 630Δ*erm* generates most of the biomass during a quasilinear growth 10 when the growth rate drops nearly logarithmically (Fig. 4C). Linear growth can be caused by limited or constant supply of an essential metabolite resulting in a constant activity of a single enzyme or a system of enzymes requiring this metabolite (e.g., vitamin B12) 29. As *C. difficile* was grown anaerobically in batch culture and all substrates were fully solved, the latter can be ruled out. Growth inhibiting reactions are difficult to identify as other reactions scale down analogously. As the organism shows exponential growth in CDMM (Table S4 and Fig. 5A), the limitation of an amino acid biosynthesis has to be the reason for linear growth. A constant glycolysis is nevertheless remarkable. As glucose is constantly taken up for synthesis of the secreted polysaccharide, glucose 6‐phosphate is available in the cell. The glycolysis activity is therefore explainable by a constitutive presence of glycolysis enzymes, acting nearly at their maximum speed.

### Alanine secretion is an unintended side effect

After the metabolic switch at 5 h of growth, when the amino acid uptake is significantly increased, alanine is secreted by *C. difficile* in MDM 10. The model predicts the degradation of alanine to be more favorable than its secretion at all time points of cultivation. The alanine secretion is hence probably an unintended side effect: the increased glutamate dehydrogenase demand due to the change in the substrate uptake is putatively not instantly satisfiable. To recreate the necessary amount of 2‐oxoglutarate, other oxoacids are aminated instead, leading to the secretion of the relatively small molecule alanine as an overflow metabolite.

### The model accurately simulates growth in CDMM

As no polysaccharide was secreted in CDMM during the exponential growth phase (Table 2), the simulations were optimized for biomass generation. After the metabolic switch at 3.5 h (see also Table S4), connected to an increase of glucose uptake, the maximization of polysaccharide secretion was the objective of optimization. The predicted growth rates are similar to the experimentally determined ones (Fig. 5A). The nearly constant growth rate of 1.0 h^−1^ between 1.5 and 3.33 h indicates a strictly exponential growth. The model also predicts 33% of the imported glucose until 4 h of growth to be bound in the secreted polysaccharide. This is in the range of the 36 ± 3% experimentally determined at an OD of 0.725, after 4 h of growth. The predicted amounts of the produced volatile fatty acids (Fig. 5B) are also in agreement with laboratory results. Leucine is first degraded using the oxidative Stickland pathway (Fig. 5A) and later degraded by both pathways and finally only degraded using the reductive Stickland pathway (Fig. 5B). In addition, after 3 h of growth, butanoate production is started and the production of acetate and propanoate is discontinued, leading to an acetate concentration of 12.81 mm in the linear growth phase, which is close to the 14.01 ± 0.41 mm determined in laboratory experiment (Table 2).

### The reductive acetyl‐CoA pathway and its importance

In the reductive acetyl‐CoA pathway, also known as Wood–Ljungdahl pathway, acetyl‐CoA is reductively generated from a one‐carbon group and CO_2_. In this pathway, the one‐carbon group is bound to a tetrahydrofolate. In the model *i*HD992, this group can either originate from formate, using the complete pathway, or originate from amino acids and arrive the pathway at 5,10‐methylene‐tetrahydrofolate. 5,10‐methylene‐tetrahydrofolate can be formed from tetrahydrofolate by the glycine hydroxymethyltransferase (EC 2.1.2.1), which degrades L‐serine to glycine, or by the glycine‐cleavage‐complex. In addition, the degradation of threonine by L‐threonine aldolase (EC 4.1.2.5) also leads to glycine.

As long as the necessary one‐carbon groups to regenerate 5,10‐methylene‐tetrahydrofolate from tetrahydrofolate are available, *C. difficile* uses the Wood–Ljungdahl pathway as primary electron sink. The activity of the acetyl‐CoA synthase complex (EC 2.3.1.169), the terminal enzyme of the reductive acetyl‐CoA pathway, is nearly constant between 1.0 and 3.0 h of growth (Fig. 5C). In contrast, the sources of one‐carbon groups vary. However, in the beginning ~50% originate from threonine and glycine. At a later stage, the importance of formate as methyl source increases until serine is taken up in significant amounts and nearly solely provides the necessary methyl groups.

### *Clostridioides difficile* ATP synthases contain at least 12 c subunits

Several H^+^/ATP and Na^+^/ATP ratios of the ATP synthases were tested in the model for the best reproduction of the laboratory results. Eventually, both ratios were set to 4, as – according to model prediction – 3.33333 or lower ratios would lead to butanoate production instead of reductive acetyl‐CoA synthesis and at 3.66667 the propanoate production would be more favorable than butanoate production.

Alternatively, a acryloyl‐CoA reductase without the electron bifurcation mechanism, leading to an energetically less feasible propanoyl‐CoA pathway, would also cause butanoate production at an Ion/ATP ratio of 3.66667. It has been shown that the enzyme of *Clostridium propionicum* can act without bifurcation 30. In addition, it was suggested that the enzymes of *C. propionicum* and *Clostridium homopropionicum* do not bifurcate 31. Nevertheless, the structure of the enzyme, consisting of a catalytic subunit as well as the EtfA and EtfB, clearly indicates an electron bifurcation mechanism as other complexes with EtfA and EtfB bifurcate electrons 32, 33, 34.

The most striking indication for an electron bifurcating acyloyl‐CoA reductase in *C. difficile* is the production of pentanoate after 2.5 h of growth in CDMM reported by Neumann‐Schaal *et al*. 10: Pentanoate can only be formed by the enzymes of the butanoate fermentation pathway using acetyl‐CoA and propanoyl‐CoA as substrates. Using a acyloyl‐CoA reductase without electron bifurcation is less energy efficient than forming butanoate, leading to no propanoyl‐CoA and thus no pentanoate formation.

The amount of imported protons or sodium ions per three ATP molecules formed is equal to the number of c subunits in the ATP synthase complex 35. We therefore conclude that *C. difficile* possesses at least 12 c subunits per ATP synthases. The number of c subunits is organism‐specific and can have any value between 10 and 15 36, 37, 38, 39, 40, 41 resulting in a ratio of 3⅓ and 5. A low ratio is more energy efficient, what is especially important for anaerobic organisms using Stickland reactions like *C. difficile*. These organisms generate organic acids as byproducts and therefore generate less ATP per mole substrate than aerobic organisms.

## Conclusions

The simulation results presented in this paper were achieved using a newly constructed model of *C. difficile* 630Δ*erm* and DFBA. It could been shown that quantitative growth and product secretion can be accurately predicted under different growth conditions in a time‐resolved manner. Thus, the quality of available *C. difficile* simulations was significantly enhanced.

*Clostridioides difficile* has remarkably metabolic peculiarities. The organism possesses a fragmented TCA cycle which is still capable to produce all metabolic precursors. In addition, the reductive acetyl‐CoA synthesis pathway is used for the depletion of nearly all excess electrons from oxidations under most of the growth conditions. The model shows the decrease of the reductive acetyl‐CoA synthesis pathway due to a lack of methyl donors during growth in CDMM. As a result, butanoate is produced instead to utilize excess electrons. This might explain the observed coupling of butanoate activity and toxin production 8, 9. The organism could be in need of methyl donors for the preferred reductive acetyl‐CoA synthesis pathway and starts to produce toxins, making the necessary amino acids available by damaging host cells.

In this paper, we describe the composition and growth coupled formation of a previously unknown exopolysaccharide of *C. difficile*. As several strains of *C. difficile* have been reported to produce capsules 27 and form biofilms 28, this polymer could be involved in both processes. As *C. difficile* in biofilms has been shown to be more resistant to vancomycin 28, the understanding and inhibition of biofilm formation is of medical importance.

## Materials and methods

### Strains, media, and growth conditions

All studies were carried out with *C. difficile* 630Δ*erm* (DSM28645) 13 obtained from the Deutsche Sammlung von Mikroorganismen und Zellkulturen (DSMZ, Braunschweig, Germany). The media and growth conditions were described earlier 10.

### Quantification of biomass components

DNA, RNA, and protein content were quantified as described earlier 42 with a prolonged lysozyme incubation for DNA quantification of 4 h.

Estimation of amino acid composition was adapted from Lee *et al*. 43. A cell pellet corresponding to approximately 1.7 mg cell dry weight was washed twice with 0.9% NaCl (w/v), resuspended in 200 μL HCl (6 m), and hydrolyzed for 24 h at 99 °C. Following neutralization, HPLC measurement of released amino acids and *meso*‐diaminopimelate was performed as previously described 44 with modifications to optimize the performance for acidic hydrolyzed samples: The mobile phase A was changed to 10 mm sodium acetate (pH 6.5) and the gradient was altered to 3% for 5.3 min, 3–6% within 0.05 min, 6–7% within 4.65 min, 7% for 1 min, 7–15% within 1 min, 15% for 10 min, 15–25% within 0.5 min, 25% for 2 min, 25–30% within 2.5 min, 30–100% within 0.1 min, and 100% for 2.9 min. To determine the concentration of released *meso*‐diaminopimelate, a calibration curve was prepared in the range of 5–300 μm. The gradient was altered between 11 and 22 min as follows: 7–14% within 1 min, 14% for 2.5 min, 14–15% within 0.25 min, and 15% for 7.25 min.

Lipids were extracted according to the protocol of Matyash *et al*. 45 and quantified gravimetrically. Dried biomass was correlated gravimetrically to the optical density of samples determined with a GENESYS 10S spectrophotometer (Thermo Fisher Scientific, Waltham, MA, USA).

### Polysaccharide analysis

Cell‐free culture supernatants were obtained during exponential, transient, and stationary growth phase from three individual biological samples.

Alkaline hydrolysis was adapted from Häggström and Förberg 24 with some modifications. Culture supernatants were mixed with 1.5 volumes of 5 m NaOH and incubated for 2 h at 22 °C followed by a neutralization step using the same volume of 5 m H_2_SO_4_. For reference samples without hydrolysis, culture supernatants were diluted with three volumes of an 1 : 1 mixture of NaOH (5 m) and H_2_SO_4_ (5 m). Medium samples were used as blanks. Samples were analyzed using the Roche Yellow line Acetic Acid kit (R‐Biopharm, Darmstadt, Germany). Each biological sample was measured in triplicates.

For glucose determination, an acidic hydrolysis was performed to avoid alkaline degradation of glucose 46. Hydrolysis procedure was adapted from Cohen and Johnstone 47 with some modifications. Culture supernatant was mixed with three volumes of 95% H_2_SO_4_, incubated on ice for 10 min, heated to 99 °C for 20 min, and cooled to 22 °C prior to neutralization with NaOH. For reference samples without hydrolysis, culture supernatant samples were diluted with premixed H_2_SO_4_ and NaOH solutions. Medium samples were used as reference for the initial glucose concentrations. Medium samples without glucose addition were used as blanks. Samples were prepared and analyzed using the Roche Yellow line Acetic Acid kit (R‐Biopharm) as described earlier 10. Each biological sample was measured in triplicates.

### GC/MS‐based analysis

Cell extracts were prepared and analyzed as described previously 15. The peak identification was performed targeted with a combined compound library. Isotope patterns were corrected for naturally occurring isotope distribution regarding silicon and carbon atoms. Natural isotopes with less than 0.5% relative abundance were neglected.

### Model generation

Reactions corresponding to the annotated gene functions 15 in *C. difficile* 630Δ*erm* were taken from BKM‐react 48 and supplemented with additional reactions from literature. The resulting reaction list was carefully filtered for metabolically redundant or energetically infeasible reactions and reaction combinations. Reactions implied by unspecific EC numbers but not by more specific product names were excluded as well as side reactions with metabolites not known in *C. difficile* or related organisms. Gaps were closed manually and as stingy as possible. Transporters were included whenever annotated, experimental evidence was existent or a transporter was strictly necessary.

### Biomass composition

The amount of protein, DNA, RNA, and total lipid in the biomass was determined experimentally. To get the full amino acid content of biomass, the measured amino acid content of hydrolyzed cells were modified assuming the measured glutamate was glutamate and glutamine in a mass ratio of 4 : 1, the aspartate was aspartate and asparagine in the mass ratio of 1 : 1, and the mass ratios of histidine, cysteine, tryptophan, and selenocysteine were 100 : 80 : 80 : 1. Assuming all *meso*‐diaminopimelate was part of the peptidoglycan, corresponding amounts of glutamate, alanine, and glycine according to the peptidoglycan structure of *C. difficile* 630 49 were subtracted to get the amino acid composition of the proteins. Based on this and the total protein concentration, the *meso*‐diaminopimelate content of the biomass was corrected and used to calculate amount of peptidoglycan in the biomass (14.5%). The ratio of the peptidoglycan and the cell wall polysaccharide II 50 was set to 4 : 6 51. The nucleotide demand for rRNA and tRNA was calculated from the corresponding genes and for DNA and mRNA from the chromosome sequence of *C. difficile* 630Δ*erm* 15. The methylation of the DNA was estimated from literature 14 to be 8000/genome. The relative amounts of the different RNAs were adapted from *E. coli* 52. The proportions of methylation and hydrogenation (23S rRNA: *E. coli*, 16S rRNA: *C. acetobutylicum*, tRNA: *Bacillus subtilis*) were taken from the MODOMICS database 53. The previously characterized lipoteichoic acid 54 was assumed to be part of the total lipid with a ratio of 8 : 20 to the phospholipids as in *B. subtilis* 55. As phosphatidylglycerol analogs were found in *C. difficile* isolates 56 and the model only includes reactions for the biosynthesis of phosphatidylglycerol and cardiolipin, an equimolar ratio of both lipids in *C. difficile* was assumed. The fatty acid chain length of 16.246 °C and the proportion of unsaturated fatty acids of 16.4% were calculated from literature 57. Glycogen was postulated to be 0.5% of the biomass. For simplification, we constructed the metabolite pool only out of cofactors and other putative important metabolites for growth otherwise not build by the model. The ratios of these metabolites were adapted from *C. acetobutylicum* in the acidogenic phase 58 and the amounts of metabolites not quantified were assumed to the equal to each other and to FAD quantified in *C. acetobutylicum* 58. As polymer precursors and all intermediates were neglected in the metabolite pool, the amount of metabolites in the biomass had to be underestimated. The demand of metabolites and ions was set to 0.5% and 2.2%, respectively. For the ion composition, values from *B. subtilis* were used 55 and complemented with Na^+^, Fe^2+^, Ni^2+^, Cd^2+^, and Zn^2+^ (1% each). The composition of biomass can be found in detail in Table S4.

### Energy requirements

For a metabolic model capable of describing the energy metabolism and quantitative growth, suitable values for the maintenance energy are required. Growth‐associated maintenance (GAM) and nongrowth‐associated maintenance (NGAM) energies are usually calculated from chemostate cultures at different dilution rates 59. As these data are not available for *C. difficile*, the NGAM was estimated as the average (2 mmol·(g_Biomass_ h)^−1^) of five anaerobic rumen bacteria 60, assuming 3.3 ATP/glucose for mixed butanoate/acetate fermentations 31. As the GAM is strongly dependent on the substrates supplied and the biomass composition of the organism, literature data could not be used. Several values for the GAM were tested and eventually the GAM was set to 45 mmol·(g_Biomass_ h)^−1^, leading to the most *in vivo* similar results.

### Data analysis for modeling

Optical densities and relative substrate concentrations during the cultivation of *C. difficile* 630Δ*erm* were derived from literature 10. The values were fitted with the originpro 2015g software (OriginLab, Northampton, MA, USA). The resulting functions of the OD and metabolite concentrations can be found in Table S3 and S4. When necessary, the data of different time frames were fitted separately to get the best mathematical description. The functions were used to compute the concentrations including the OD every 15 min starting at 7.5 min (MDM) or 10 min starting at 5 min (CDMM). Relative substrate concentrations were converted to absolute concentrations setting the concentration at time point zero at the concentration in the initial medium. The biomass was calculated using the correlation of 0.425 g·L^−1^ biomass dry weight per optical density unit at 600 nm wavelength.

### (Time‐resolved) flux balance analysis

Flux balance analysis, extensively described elsewhere 61, was used to analyze the physiological states of *C. difficile* 630Δ*erm*. The metano toolbox 62 was used for all simulations. For the simulation of the complex growth behavior in batch cultures, DFBA (see Antoniewicz 63 for details) was used. As objective function was either the biomass generation equation used or the secretion of the expolysaccharide with a fixed biomass generation flux derived from laboratory experiment. In contrast to earlier published DFBA methods 17, 18, the substrate consumption and some production rates were given as constrains at each time interval. These rates were derived from the fits of the measured substrate and product concentrations over time, similar to the dynamic metabolic flux analysis used to describe the behavior of *E. coli* during change of the limiting substrate 64.

For the modeling of the biosynthesis of the secreted polysaccharide, UDP‐glucose was used as substrate for the polymerization, every second glucose subunit was acetylated with acetyl‐CoA and the hydrolysis of one ATP was assumed as transport cost per glucose subunit across the membrane. To calculate the total conversion of glucose to polysaccharide in MDM, 90% of the taken up glucose prior to the linear growth phase was assumed. The fatty acid synthesis was restricted to use only acetyl phosphate as starter molecule to prevent large‐scale synthesis of fatty acids with up to six carbon atoms via the Stickland reactions and the butanoate pathway.

For the correct reproduction of the experimental data in CDMM, two predefinitions had to be made. First, for an accurate simulation of the side product secretion in CDMM, the methylenetetrahydrofolate dehydrogenase (NADP^+^, EC 1.5.1.5) had to be deactivated after 2.5 h of growth to prevent the refilling of the one‐carbon‐pool from formate. The reason for the inactivation of this pathway *in vivo* remains unclear. Second, the production of propanoate and hexanoate via the butanoate fermentation pathway had to be limited: the maximal fluxes of the acetyl‐CoA C‐acetyltransferase (EC 2.3.1.9) for the substrates propanoyl‐CoA and butanoyl‐CoA were set to 1/3 and 1/20 of the flux with acetyl‐CoA as the substrate.

## Author contributions

HD, DS, and MNS conceived and designed the experiments. HD was responsible for model generation and simulations. SEW and MNS performed the laboratory experiments. HD, DS, and MNS wrote the manuscript. All authors read and approved the final manuscript.

## Supporting information


**Model S1.** Metabolic model *i*HD992 of *C. difficile* 630Δ*erm*.
**Scenario S1.** Exemplary scenario file (MDM, 3 h) for the metabolic model *i*HD992 of *C. difficile* 630Δ*erm*.
**Table S1.** Isotope labeling of glycolysis and glycolysis‐related metabolites.
**Table S2.** Biomass composition used for the model *i*HD992.
**Table S3.** Fits of the substrate concentrations and OD over time of MDM cultures with *C. difficile* 630Δ*erm*.
**Table S4.** Fits of the substrate concentrations and OD over time of CDMM cultures with *C. difficile* 630Δ*erm*.
