# Supplementary material for: Clostridioides difficile 630Δerm in silico and in vivo – quantitative growth and extensive polysaccharide secretion
Source: FEBS Open Bio. 2017 Mar 9;7(4):602–15. doi: 10.1002/2211-5463.12208 (PMC5377389; doi:10.1002/2211-5463.12208)
Supplement: Supplementary file 1 — Model S1. Metabolic model iHD992 of C. difficile 630Δerm. Scenario S1. Exemplary scenario file (MDM, 3 h) for the metabolic model iHD992 of C. difficile 630Δerm. Table S1. Isotope labeling of glycolysis and glycolysis‐related metabolites. Table S2. Biomass composition used for the model iHD992. Table S3. Fits of the substrate concentrations and OD over time of MDM cultures with C. difficile 630Δerm. Table S4. Fits of the substrate concentrations and OD over time of CDMM cultures with C. difficile 630Δerm. [file FEB4-7-602-s001.pdf]

Model S1: **Metabolic model *i*HD922 of *C. difficile* 630Δ*erm***

##

#Biomass

Protein_via_tRNA : 0.59772 |Charged-GLY-tRNAs| + 0.63120 |Charged-ALA-tRNAs| + 0.44284 |Charged-VAL-tRNAs| + 0.62167 |Charged-LEU-tRNAs| + 0.37755 |Charged-ILE-tRNAs| + 0.43067 |Charged-SER-tRNAs| + 0.44035 |Charged-THR-tRNAs| + 0.26329 |Charged-PHE-tRNAs| + 0.21261 |Charged-TYR-tRNAs| + 0.06332 |Charged-TRP-tRNAs| + 0.09966 |Charged-CYS-tRNAs| + 0.07019 |Charged-MET-tRNAs| + 0.65890 |Charged-LYS-tRNAs| + 0.27429 |Charged-ARG-tRNAs| + 0.10099 |Charged-HIS-tRNAs| + 0.47959 |Charged-ASP-tRNAs| + 0.88041 |Charged-GLT-tRNAs| + 0.47953 |Charged-ASN-tRNAs| + 0.29371 |Charged-GLN-tRNAs| + 0.36118 |Charged-PRO-tRNAs| + 0.00094 |Charged-SEC-tRNAs| --> 1.0 Protein + 0.59772 |GLY-tRNAs| + 0.63120 |ALA-tRNAs| + 0.44284 |VAL-tRNAs| + 0.62167 |LEU-tRNAs| + 0.37755 |ILE-tRNAs| + 0.43067 |SER-tRNAs| + 0.44035 |THR-tRNAs| + 0.26329 |PHE-tRNAs| + 0.21261 |TYR-tRNAs| + 0.06332 |TRP-tRNAs| + 0.09966 |CYS-tRNAs| + 0.07019 |MET-tRNAs| + 0.65890 |LYS-tRNAs| + 0.27429 |ARG-tRNAs| + 0.10099 |HIS-tRNAs| + 0.47959 |ASP-tRNAs| + 0.88041 |GLT-tRNAs| + 0.47953 |ASN-tRNAs| + 0.29371 |GLN-tRNAs| + 0.36118 |PRO-tRNAs| + 0.00094 |SEC-tRNAs| + 15.56122 H+

RNA-Biosyn : 0.83394 ATP + 0.69615 UTP + 0.66708 GTP + 0.90005 CTP + 0.02785 S-adenosyl-L-methionine + 0.00657 NADPH --> 0.02128 H+ + 3.09722 diphosphate + 1.0 RNA + 0.02785 S-adenosyl-L-homocysteine + 0.00657 NADP+

DNA-Biosyn : 1.15183 dATP + 1.15183 dTTP + 0.47233 dGTP + 0.47233 dCTP + 0.00303 S-adenosyl-L-methionine --> 0.00303 H+ + 1.0 DNA + 0.00303 S-adenosyl-L-homocysteine + 3.24832 diphosphate

Lipid-Biosyn : 0.69236 Phosphatidylglycerol + 0.36976 a_cardiolipin --> 1.0 Lipid

L-CWG-Biosyn : 2.59231 UDP-N-acetyl-alpha-D-glucosamine + 1.29615 2-phospho-D-glycerate + 0.76635 UDP-alpha-D-glucose + 0.25545 |diacyl-3-Glc-16-Glc-16-Glc-glycerol| + 1.29615 ATP + 0.05676 H2O --> 1.0 L-CWG + 3.35866 UDP + 0.31221 acetate + 1.295615 ADP + 1.29615 phosphate + 3.67087 H+ # H+ = NAcGlu + Gluc + DeAc + ATP - 2PG; H2O = 12DG + 2PG -DeAc -ATP

Peptidoglycan-Biosyn : 1.17591 a_peptidoglycan --> 1.0 Peptidoglycan

P-CWG-Biosyn : 1.76376 UDP-N-acetyl-alpha-D-galactosamine + 0.88188 GDP-alpha-D-mannose + 2.64564 UDP-alpha-D-glucose --> 1.0 P-CWG + 0.88188 GMP + 4.40940 UDP + 5.29128 H+

Glycogen-Biosyn : 6.16732 |Glycogens| --> 1.0 Glycogen

Ion-Biosyn : 20.96743 K+ + 1.87732 Mg2+ + 0.14628 Fe3+ + 0.09752 Ca2+ + 1.04837 phosphate + 0.12190 diphosphate + 0.02438 Na+ + 0.02438 Fe2+ + 0.02438 Ni2+ + 0.02438 Cd2+ + 0.02438 Zn2+ --> 1.0 Ion

Metabolite-Biosyn : 0.33818 ATP + 0.08481 ADP + 0.03094 AMP + 0.17332 acetyl-CoA + 0.06667 FAD + 0.16617 NAD+ + 0.03514 NADH + 0.00043 NADP+ + 0.02501 NADPH + 0.06667 ppGpp + 0.06667 tetrahydropteroyl_tri-L-glutamate + 0.06667 di-trans,octa-cis-undecaprenyl_diphosphate + 0.06667 thiamine_diphosphate + 0.06667 adenosylcobalamin + 0.06667 Mg-protoporphyrin + 0.06667 MoO2-molybdopterin_cofactor + 0.06667 |Carboxybiotin-BCCP| + 0.06667 Me-Thr-P-GluNAc-FliC + 0.06667 pyridoxine_5'-phosphate + 0.06667 L-threonylcarbamoyladenylate --> 1.0 Metabolites + 0.13334 |BCCP-monomers|

Protease : 1.0 Protein + 7.78061 H2O --> 0.59772 glycine + 0.63120 L-alanine + 0.44284 L-valine + 0.62167 L-leucine + 0.37755 L-isoleucine + 0.43067 L-serine + 0.44035 L-threonine + 0.26329 L-phenylalanine + 0.21261 L-tyrosine + 0.06332 L-tryptophan + 0.09966 L-cysteine + 0.07019 L-methionine + 0.65890 L-lysine + 0.27429 L-arginine + 0.10099 L-histidine + 0.47959 L-aspartate + 0.88041 L-glutamate + 0.47953 L-asparagine + 0.29371 L-glutamine + 0.36118 L-proline + 0.0094 L-selenocysteine

RNAase : 1.0 RNA + 0.02858 S-adenosyl-L-homocysteine + 0.00695 NAD+ + 3.13828 H2O --> 0.83394 AMP + 0.69615 UMP + 0.66708 GMP + 0.90005 CMP + 0.02858 S-adenosyl-L-methionine + 0.00695 NADH + 3.11665 H+

DNAase : 1.0 DNA + 0.00303 S-adenosyl-L-homocysteine + 3.24832 H2O --> 1.15220 dAMP + 1.15220 dTMP + 0.47195 dGMP + 0.47195 dCMP + 0.00303 S-adenosyl-L-methionine + 3.24529 H+

Biomass : 0.508 Protein + 0.091 RNA + 0.043 DNA + 0.060 Lipid + 0.024 L-CWG + 0.145 Peptidoglycan + 0.097 P-CWG + 0.005 Glycogen + 0.022 Ion + 0.005 Metabolites + 45 ATP + 45 H2O --> 45 ADP + 45 phosphate + 45 H+

NGAM : 1.0 H2O + 1.0 ATP --> 1.0 ADP + 1.0 phosphate + 1.0 H+

##

#EXCHANGE

###################

H+_exchange : 1.0 H+[ex] <=>

H2O_exchange : 1.0 H2O[ex] <=>

H2_exchange : 1.0 H2[ex] <=>

H2S_exchange : 1.0 hydrogen_sulfide[ex] <=>

CO2_exchange : 1.0 CO2[ex] <=>

CO_exchange : 1.0 carbon_monoxide[ex] <=>

```
ammonium_exchange                              : 1.0 ammonium[ex] <=>
sulfite_exchange                               : 1.0 sulfite[ex] <=>

phosphate_exchange                             : 1.0 phosphate[ex] <=>
selenide_exchange                              : 1.0 selenide[ex] <=>

Co2+_exchange                                  : 1.0 Co2+[ex] <=>
Na+_exchange                                   : 1.0 Na+[ex] <=>
K+_exchange                                    : 1.0 K+[ex] <=>
Mg2+_exchange                                  : 1.0 Mg2+[ex] <=>
Fe2+_exchange                                  : 1.0 Fe2+[ex] <=>
Fe3+_exchange                                  : 1.0 Fe3+[ex] <=>
Ca2+_exchange                                  : 1.0 Ca2+[ex] <=>
Ni2+_exchange                                  : 1.0 Ni2+[ex] <=>
Cd2+_exchange                                  : 1.0 Cd2+[ex] <=>
Zn2+_exchange                                  : 1.0 Zn2+[ex] <=>
molybdate_exchange                             : 1.0 molybdate[ex] <=>

D-Glucose_exchange                            : 1.0 D-glucose[ex] <=>
urea_exchange                                  : 1.0 urea[ex] <=>
methanethiol_exchange                          : 1.0 methanethiol[ex] <=>

ethanol_exchange                              : 1.0 ethanol[ex] <=>
propanol_exchange                             : 1.0 n-propanol[ex] <=>
butanol_exchange                              : 1.0 n-butanol[ex] <=>
isobutanol_exchange                           : 1.0 isobutanol[ex] <=>
4-methylphenol_exchange                       : 1.0 4-methylphenol[ex] <=>
acetone_exchange                              : 1.0 acetone[ex] <=>

(R)-pantothenate_exchange                      : 1.0 (R)-pantothenate[ex] <=>
biotin_exchange                                : 1.0 biotin[ex] <=>
pyridoxine_exchange                            : 1.0 pyridoxine[ex] <=>

2-aminobutanoate_exchange                      : 1.0 (S)-2-aminobutanoate[ex] <=>
S-methyl-5-thio-D-ribose_exchange              : 1.0 S-methyl-5-thio-D-ribose[ex] <=>
5-aminopentanoate_exchange                     : 1.0 5-aminopentanoate[ex] <=>
THMF_exchange                                  : 1.0 (2R,4S)-2-methyl-2,3,3,4-tetrahydroxytetrahydrofuran[ex] <=>
5-deoxy-D-ribose_exchange                      : 1.0 5-deoxy-D-ribose[ex] <=>
beta-alanine_exchange                          : 1.0 beta-alanine[ex] <=>
3-amino-2-methylpropanoate_exchange            : 1.0 (R)-3-amino-2-methylpropanoate[ex] <=>
aminoaceton_exchange                           : 1.0 aminoacetone[ex] <=>
glycolaldehyde_exchange                        : 1.0 glycolaldehyde[ex] <=>
S-methyl-5-thio-alpha-D-ribose_1-phosphate_exchange      : 1.0 S-methyl-5-thio-alpha-D-ribose_1-phosphate[ex] <=>


valine_exchange                            : 1.0 L-valine[ex] <=>
leucine_exchange                           : 1.0 L-leucine[ex] <=>
isoleucine_exchange                        : 1.0 L-isoleucine[ex] <=>
tryptophan_exchange                        : 1.0 L-tryptophan[ex] <=>
cysteine_exchange                          : 1.0 L-cysteine[ex] <=>
methionine_exchange                        : 1.0 L-methionine[ex] <=>
proline_exchange                           : 1.0 L-proline[ex] <=>

glycine_exchange                           : 1.0 glycine[ex] <=>
alanine_exchange                           : 1.0 L-alanine[ex] <=>
aspartate_exchange                         : 1.0 L-aspartate[ex] <=>
glutamate_exchange                         : 1.0 L-glutamate[ex] <=>
serine_exchange                            : 1.0 L-serine[ex] <=>
threonine_exchange                         : 1.0 L-threonine[ex] <=>
tyrosine_exchange                          : 1.0 L-tyrosine[ex] <=>
phenylalanine_exchange                     : 1.0 L-phenylalanine[ex] <=>
arginine_exchange                          : 1.0 L-arginine[ex] <=>
histidine_exchange                         : 1.0 L-histidine[ex] <=>
```

```
lysine_exchange                            : 1.0 L-lysine[ex] <=>


formate_exchange                              : 1.0 formate[ex] <=>
acetate_exchange                              : 1.0 acetate[ex] <=>
propanoate_exchange                           : 1.0 propanoate[ex] <=>
butanoate_exchange                            : 1.0 butanoate[ex] <=>
valerate_exchange                             : 1.0 pentanoate[ex] <=>
hexanoate_exchange                            : 1.0 hexanoate[ex] <=>
(S)-lactate_exchange                          : 1.0 (S)-lactate[ex] <=>
(R)-lactate_exchange                          : 1.0 (R)-lactate[ex] <=>
isocaproate_exchange                          : 1.0 isocaproate[ex] <=>
(R)-3-hydroxybutanoate_exchange               : 1.0 (R)-3-hydroxybutanoate[ex] <=>

indole-3-acetate_exchange                     : 1.0 indole-3-acetate[ex] <=>
4-hydroxyphenylacetate_exchange               : 1.0 4-hydroxyphenylacetate[ex] <=>
phenylacetate_exchange                        : 1.0 phenylacetate[ex] <=>
4-imidazoleacetate_exchange                   : 1.0 4-imidazoleacetate[ex] <=>

3-phenylpropanoate_exchange                   : 1.0 3-phenylpropanoate[ex] <=>
3-(4-hydroxyphenyl)propanoate_exchange        : 1.0 3-(4-hydroxyphenyl)propanoate[ex] <=>

isobutyrate_exchange                          : 1.0 isobutyrate[ex] <=>
isovalerate_exchange                          : 1.0 isovalerate[ex] <=>
2-methylbutanoate_exchange                    : 1.0 2-methylbutanoate[ex] <=>
##############################################################################################################################################################################
#TRANSPORT
###################
#Diffusion

H2O_transport                                  : 1.0 H2O <=> 1.0 H2O[ex]
H2_transport                                   : 1.0 H2 <=> 1.0 H2[ex]
CO2_transport                                  : 1.0 CO2 <=> 1.0 CO2[ex]
CO_transport                                   : 1.0 carbon_monoxide <=> 1.0 carbon_monoxide[ex]
H2S_transport                                  : 1.0 hydrogen_sulfide <=> hydrogen_sulfide[ex]
methanethiol_transport                         : 1.0 methanethiol <=> 1.0 methanethiol[ex]

#No ammonium transporter (amtB/nrgA)
#Ammonium can diffuse with the gradient across the membrane (Kleiner 1985)
#Usually no import necessary due to amino acid degradation
spontaneous_no-pathway_RXN-11811               : 1.0 ammonia + 1.0 H+ <=> 1.0 ammonium  # metacyc
ammonia_transport                              : 1.0 ammonia <=> 1.0 ammonia[ex]
spontaneous_no-pathway_RXN-11811[ex]           : 1.0 ammonia[ex] + 1.0 H+[ex] <=> 1.0 ammonium[ex]  # metacyc

#Diffusion as uncharged molecule assumed
spontaneous_sulfite_dissotiation               : 1.0 sulfite + 2.0 H+ <=> 1.0 SO2 + H2O
sulfite_transport                              : 1.0 SO2 <=> 1.0 SO2[ex]
spontaneous_sulfite_dissotiation[ex]           : 1.0 sulfite[ex] + 2.0 H+[ex] <=> 1.0 SO2[ex] + H2O[ex]

#Diffusion as uncharged molecule assumed
spontaneous_selenide_dissotiation              : 1.0 hydrogen_selenide <=> 2.0 H+ + 1.0 selenide
selenide_transport                             : 1.0 hydrogen_selenide <=> 1.0 hydrogen_selenide[ex]
spontaneous_selenide_dissotiation[ex]          : 1.0 hydrogen_selenide[ex] <=> 2.0 H+[ex] + 1.0 selenide[ex]

#products
ethanol_transport                              : 1.0 ethanol <=> 1.0 ethanol[ex]
propanol_transport                             : 1.0 n-propanol <=> 1.0 n-propanol[ex]
butanol_transport                              : 1.0 n-butanol <=> 1.0 n-butanol[ex]
isobutanol_transport                           : 1.0 isobutanol <=> 1.0 isobutanol[ex]
4-methylphenol_transport                       : 1.0 4-methylphenol <=> 1.0 4-methylphenol[ex]
acetone_transport                              : 1.0 acetone <=> 1.0 acetone[ex]

#small-quantity products/deadends
aminoaceton_transport                          : 1.0 aminoacetone <=> 1.0 aminoacetone[ex]
```

```
urea_transport                                : 1.0 urea <=> 1.0 urea[ex]
THMF_transport                                : 1.0 (2R,4S)-2-methyl-2,3,3,4-tetrahydroxytetrahydrofuran <=> 1.0 (2R,4S)-2-methyl-2,3,3,4-tetrahydroxytetrahydrofuran[ex]
S-methyl-5-thio-D-ribose_transport                       : 1.0 S-methyl-5-thio-D-ribose <=> 1.0 S-methyl-5-thio-D-ribose[ex]
S-methyl-5-thio-alpha-D-ribose_1-phosphate_transport      : 1.0 S-methyl-5-thio-alpha-D-ribose_1-phosphate <=> 1.0 S-methyl-5-thio-alpha-D-ribose_1-phosphate[ex]
5-deoxy-D-ribose_transport                               : 1.0 5-deoxy-D-ribose <=> 1.0 5-deoxy-D-ribose[ex]
glycolaldehyde_transport                                 : 1.0 glycolaldehyde <=> 1.0 glycolaldehyde[ex]
###################
#Weak acids

##CDIF630erm_03708,CDIF630erm_02463
formate_transport                             : 1.0 H+ + 1.0 formate <=> 1.0 formate[ex] + 1.0 H+[ex]
acetate_transport                             : 1.0 H+ + 1.0 acetate <=> 1.0 acetate[ex] + 1.0 H+[ex]
propanoate_transport                          : 1.0 H+ + 1.0 propanoate <=> 1.0 propanoate[ex] + 1.0 H+[ex]
n-butanoate_transport                         : 1.0 H+ + 1.0 butanoate <=> 1.0 butanoate[ex] + 1.0 H+[ex]
pentanoate_transport                          : 1.0 H+ + 1.0 pentanoate <=> 1.0 pentanoate[ex] + 1.0 H+[ex]
hexanoate_transport                           : 1.0 H+ + 1.0 hexanoate <=> 1.0 hexanoate[ex] + 1.0 H+[ex]
(R)-3-hydroxybutanoate_transport              : 1.0 H+ + 1.0 (R)-3-hydroxybutanoate <=> 1.0 (R)-3-hydroxybutanoate[ex] + 1.0 H+[ex]
##CDIF630erm_00920
(S)-lactate_transport                         : 1.0 Na+ + 1.0 (S)-lactate <=> 1.0 (S)-lactate[ex] + 1.0 Na+[ex]
(R)-lactate_transport                         : 1.0 Na+ + 1.0 (R)-lactate <=> 1.0 (R)-lactate[ex] + 1.0 Na+[ex]

indole-3-acetate_transport                    : 1.0 H+ + 1.0 indole-3-acetate <=> 1.0 indole-3-acetate[ex] + 1.0 H+[ex]
4-hydroxyphenylacetate_transport              : 1.0 H+ + 1.0 4-hydroxyphenylacetate <=> 1.0 4-hydroxyphenylacetate[ex] + 1.0 H+[ex]
phenylacetate_transport                       : 1.0 H+ + 1.0 phenylacetate <=> 1.0 phenylacetate[ex] + 1.0 H+[ex]
4-imidazoleacetate_transport                  : 1.0 H+ + 1.0 4-imidazoleacetate <=> 1.0 4-imidazoleacetate[ex] + 1.0 H+[ex]

3-phenylpropanoate_transport                  : 1.0 H+ + 1.0 3-phenylpropanoate <=> 3-phenylpropanoate[ex] + 1.0 H+[ex]
3-(4-hydroxyphenyl)propanoate_transport       : 1.0 H+ + 1.0 3-(4-hydroxyphenyl)propanoate <=> 3-(4-hydroxyphenyl)propanoate[ex] + 1.0 H+[ex]

isobutyrate_transport                         : 1.0 H+ + 1.0 isobutyrate <=> 1.0 isobutyrate[ex] + 1.0 H+[ex]
isovalerate_transport                         : 1.0 H+ + 1.0 isovalerate <=> 1.0 isovalerate[ex] + 1.0 H+[ex]
2-methylbutanoate_transport                   : 1.0 H+ + 1.0 2-methylbutanoate <=> 1.0 2-methylbutanoate[ex] + 1.0 H+[ex]
isocaproate_transport                         : 1.0 H+ + 1.0 isocaproate <=> 1.0 isocaproate[ex] + 1.0 H+[ex]
###################
#Amino acids

##CDIF630erm_02711; CDIF630erm_01724
threonine_antiport                            : 1.0 L-serine[ex] + 1.0 L-threonine <=> 1.0 L-serine + 1.0 L-threonine[ex]

##CDIF630erm_02948
serine_transport                              : 1.0 Na+ + 1.0 L-serine <=> 1.0 Na+[ex] + 1.0 L-serine[ex]
threonine_transport                           : 1.0 Na+ + 1.0 L-threonine <=> 1.0 Na+[ex] + 1.0 L-threonine[ex]

##CDIF630erm_02511, CDIF630erm_02664
alanine_transport                             : 1.0 Na+ + 1.0 L-alanine <=> 1.0 Na+[ex] + 1.0 L-alanine[ex]
#glycine similar to alanine
glycine_transport                             : 1.0 Na+ + 1.0 glycine <=> 1.0 Na+[ex] + 1.0 glycine[ex]

##CDIF630erm_02521, CDIF630erm_02522; CDIF630erm_02958; CDIF630erm_01412; CDIF630erm_01413; CDIF630erm_00949
valine_transport                              : 1.0 Na+ + 1.0 L-valine <=> 1.0 Na+[ex] + 1.0 L-valine[ex]
leucine_transport                             : 1.0 Na+ + 1.0 L-leucine <=> 1.0 Na+[ex] + 1.0 L-leucine[ex]
isoleucine_transport                          : 1.0 Na+ + 1.0 L-isoleucine <=> 1.0 Na+[ex] + 1.0 L-isoleucine[ex]

##CDIF630erm_02197
tryptophan_transport                          : 1.0 Na+ + 1.0 L-tryptophan <=> 1.0 Na+[ex] + 1.0 L-tryptophan[ex]

##CDIF630erm_00949? Used by the Leu-Operon; CDIF630erm_02197? Similar to trp
tyrosine_transport                            : 1.0 Na+ + 1.0 L-tyrosine <=> 1.0 Na+[ex] + 1.0 L-tyrosine[ex]
phenylalanine_transport                       : 1.0 Na+ + 1.0 L-phenylalanine <=> 1.0 Na+[ex] + 1.0 L-phenylalanine[ex]

##CDIF630erm_01940
glutamate_transport                           : 1.0 Na+ + 1.0 L-glutamate <=> 1.0 L-glutamate[ex] + 1.0 Na+[ex]

#Transport unknown, Na+ symport as a guess
```

```
proline_transport                              : 1.0 Na+ + 1.0 L-proline <=> 1.0 Na+[ex] + 1.0 L-proline[ex]
cysteine_transport                             : 1.0 Na+ + 1.0 L-cysteine <=> 1.0 Na+[ex] + 1.0 L-cysteine[ex]
lysine_transport                               : 1.0 Na+ + 1.0 L-lysine <=> 1.0 Na+[ex] + 1.0 L-lysine[ex]
5-aminopentanoate_transport                    : 1.0 Na+ + 1.0 5-aminopentanoate <=> 1.0 Na+[ex] + 1.0 5-aminopentanoate[ex]
2-aminobutanoate_transport                     : 1.0 Na+ + 1.0 (S)-2-aminobutanoate <=> 1.0 Na+[ex] + 1.0 (S)-2-aminobutanoate[ex]
beta-alanine_transport                         : 1.0 Na+ + 1.0 beta-alanine <=> 1.0 Na+[ex] + 1.0 beta-alanine[ex]
(R)-pantothenate_transport                     : 1.0 Na+ + 1.0 (R)-pantothenate <=> 1.0 Na+[ex] + 1.0 (R)-pantothenate[ex]
(R)-3-amino-2-methylpropanoate_transport       : 1.0 Na+ + 1.0 (R)-3-amino-2-methylpropanoate[ex] <=> 1.0 Na+[ex] + 1.0 (R)-3-amino-2-methylpropanoate

#CDIF630erm_00868-CDIF630erm_00870
arginine_3.6.3.-_transport                     : 1.0 H2O + 1.0 ATP + 1.0 L-arginine[ex] --> 1.0 H+ + 1.0 phosphate + 1.0 ADP + 1.0 L-arginine

##CDIF630erm_01654-CDIF630erm_01656
methionine_transport                           : 1.0 L-methionine[ex] + 1.0 H2O + 1.0 ATP --> 1.0 ADP + 1.0 L-methionine + 1.0 phosphate + 1.0 H+

#unknown, ABC-transporter is a guess
##CDIF630erm_01972-CDIF630erm_01974?; CDIF630erm_00868-CDIF630erm_00870?
aspartate_transport                            : 1.0 H2O + 1.0 L-aspartate[ex] + 1.0 ATP --> 1.0 L-aspartate + 1.0 ADP + 1.0 phosphate + 1.0 H+
histidine_transport                            : 1.0 H2O + 1.0 ATP + 1.0 L-histidine[ex] --> 1.0 H+ + 1.0 phosphate + 1.0 ADP + 1.0 L-histidine
pyridoxine_transport                           : 1.0 H2O + 1.0 ATP + 1.0 pyridoxine[ex] --> 1.0 H+ + 1.0 phosphate + 1.0 ADP + 1.0 pyridoxine
###################
#ABC-Transport

phosphate_transport                            : 1.0 H2O + 1.0 ATP + 1.0 phosphate[ex] --> 1.0 ADP + 2.0 phosphate + 1.0 H+

Ni2+_transport                                 : 1.0 H2O + 1.0 ATP + 1.0 Ni2+[ex] --> 1.0 Ni2+ + 1.0 ADP + 1.0 phosphate + 1.0 H+
#CDIF630erm_00452-CDIF630erm_00455
Co2+_transport_3.6.3.2_3.6.3.5                 : 1.0 H2O + 1.0 Co2+[ex] + 1.0 ATP --> 1.0 phosphate + 1.0 ADP + 1.0 Co2+ + 1.0 H+
Mg2+_transport_3.6.3.2_                        : 1.0 H2O + 1.0 Mg2+[ex] + 1.0 ATP --> 1.0 Mg2+ + 1.0 phosphate + 1.0 ADP + 1.0 H+
Fe3+_transport_3.6.3.30                        : 1.0 ATP + 1.0 Fe3+[ex] + 1.0 H2O --> 1.0 ADP + 1.0 Fe3+ + 1.0 phosphate + 1.0 H+
K+_transport_3.6.3.12                          : 1.0 H2O + 1.0 K+[ex] + 1.0 ATP --> 1.0 K+ + 1.0 phosphate + 1.0 ADP + 1.0 H+
Ca2+_transport_3.6.3.8                         : 1.0 ATP + 1.0 H2O + 1.0 Ca2+[ex] --> 1.0 phosphate + 1.0 ADP + 1.0 Ca2+ + 1.0 H+
##CDIF630erm_00989-CDIF630erm_00991
molybdate_transport_3.6.3.29                   : 1.0 H2O + 1.0 ATP + 1.0 molybdate[ex] --> 1.0 phosphate + 1.0 ADP + 1.0 molybdate + 1.0 H+
##CDIF630erm_01641 - CDIF630erm_01643; CDIF630erm_01684, CDIF630erm_01685; CDIF630erm_01939; CDIF630erm_03573, CDIF630erm_03574
Fe2+_transport                                 : 1.0 GTP + 1.0 H2O + 1.0 Fe2+[ex] --> 1.0 phosphate + 1.0 H+ + 1.0 GDP  + 1.0 Fe2+
Cd2+_transport_3.6.3.3                         : 1.0 H2O + 1.0 ATP + 1.0 Cd2+[ex] <=> 1.0 Cd2+ + 1.0 ADP + 1.0 phosphate + 1.0 H+
Zn2+_transport_3.6.3.5                         : 1.0 Zn2+[ex] + 1.0 ATP + 1.0 H2O --> 1.0 Zn2+ + 1.0 phosphate + 1.0 ADP + 1.0 H+

###################
#Other

##CDIF630erm_03256
biotin_transport                               : 1.0 biotin[ex] <=> 1.0 biotin

##CDIF630erm_02351
Mg2+_transport                                 : 1.0 Mg2+[ex] <-- 1.0 Mg2+

##CDIF630erm_01919
PO4/Na_transport                               : 1.0 Na+[ex] + 1.0 phosphate[ex] <=> 1.0 Na+ + 1.0 phosphate

##CDIF630erm_02923; CDIF630erm_02325; CDIF630erm_03939
potassium_transport                            : 1.0 K+[ex] + 1.0 H+ <=> 1.0 K+ + 1.0 H+[ex]


###################
#Proton and Na+ transport

#10 c-subunits (9-12 normal?) in E.coli (Jiang et al., 2001) --> 3.3 H+/ATP
#F-type-ATPase_transport_3.6.3.14_mod_ATPSYN-RXN                          : 1.0 H2O + 1.0 ATP + 2.33333 H+ <=> 1.0 phosphate + 1.0 ADP + 3.33333 H+[ex]
#F-type-ATPase_transport_3.6.3.14_mod_ATPSYN-RXN                          : 1.0 H2O + 1.0 ATP + 2.66667 H+ <=> 1.0 phosphate + 1.0 ADP + 3.66667 H+[ex]
#F-type-ATPase_transport_3.6.3.14_mod_ATPSYN-RXN                          : 1.0 H2O + 1.0 ATP + 3 H+ <=> 1.0 phosphate + 1.0 ADP + 4 H+[ex]
F-type-ATPase_transport_3.6.3.14_mod_ATPSYN-RXN                          : 1.0 H2O + 1.0 ATP + 3.33333 H+ <=> 1.0 phosphate + 1.0 ADP + 4.33333 H+[ex]
```

```
#Subunit K is highly similar to E. hirae; E. hirae has 10 K-subunits per ATPase (Murata et al., 2005) --> 3.3 Na+/ATP
#V-type-ATPase_transport_3.6.3.15_mod_3.6.3.15-RXN                       : 1.0 H2O + 1.0 ATP + 3.33333 Na+ <=> 1.0 phosphate + 1.0 ADP + 3.33333 Na+[ex] + 1.0 H+
#V-type-ATPase_transport_3.6.3.15_mod_3.6.3.15-RXN                       : 1.0 H2O + 1.0 ATP + 3.66667 Na+ <=> 1.0 phosphate + 1.0 ADP + 3.66667 Na+[ex] + 1.0 H+
#V-type-ATPase_transport_3.6.3.15_mod_3.6.3.15-RXN                       : 1.0 H2O + 1.0 ATP + 4 Na+ <=> 1.0 phosphate + 1.0 ADP + 4 Na+[ex] + 1.0 H+
V-type-ATPase_transport_3.6.3.15_mod_3.6.3.15-RXN                       : 1.0 H2O + 1.0 ATP + 4.33333 Na+ <=> 1.0 phosphate + 1.0 ADP + 4.33333 Na+[ex] + 1.0 H+

##CDIF630erm_01284-CDIF630erm_01289
#Proton dependence assumed because the Rnf-complex of C. ljungdahlii is proton dependent (Tremblay et al., 2013)
#Reversible in vitro in A. woodii (Hess et al., 2013)
rnfABCDEG_transport                                                     : 2.0 |Reduced-ferredoxins| + 1.0 NAD+ + 3.0 H+ <=> 2.0 |Oxidized-ferredoxins| + 1.0 NADH + 2.0 H+[ex]

##CDIF630erm_00770; CDIF630erm_02996; CDIF630erm_02923
sodium_transport                                                        : 1.0 Na+[ex] + 1.0 H+ <=> 1.0 Na+ + 1.0 H+[ex]

#############Glucose PTS-system#############
PTS-system_2.7.3.9_2.7.3.9-RXN                              : 1.0 phosphoenolpyruvate + 1.0 |PTS-I-Histidines| --> 1.0 pyruvate + 1.0 |PTS-I-pi-phospho-L-histidines|  # source:
MetaCyc (queried 2.7.3.9)
glucose_transport_2.7.1.199                                 : 1.0 |PTS-I-pi-phospho-L-histidines| + 1.0 D-glucose[ex] --> 1.0 |PTS-I-Histidines| + 1.0 D-glucose_6-phosphate
#######################################################################################################################################################################
#glycolysis I:

glycolysis-I/1_5.3.1.9_PGLUCISOM-RXN                        : 1.0 D-glucose_6-phosphate <=> 1.0 beta-D-fructofuranose_6-phosphate  # source: MetaCyc, BRENDA, KEGG (queried
5.3.1.9)
glycolysis-I/2b_3.1.3.11_F16BDEPHOS-RXN                     : 1.0 fructose_1,6-bisphosphate + 1.0 H2O --> 1.0 beta-D-fructofuranose_6-phosphate + 1.0 phosphate  # source: MetaCyc,
BRENDA, KEGG (queried 3.1.3.11)
glycolysis-I/2a_2.7.1.11_6PFRUCTPHOS-RXN                    : 1.0 beta-D-fructofuranose_6-phosphate + 1.0 ATP --> 1.0 fructose_1,6-bisphosphate + 1.0 H+ + 1.0 ADP  # source:
MetaCyc, BRENDA, KEGG (queried 2.7.1.11)
glycolysis-I/3_4.1.2.13_F16ALDOLASE-RXN                     : 1.0 fructose_1,6-bisphosphate <=> 1.0 D-glyceraldehyde_3-phosphate + 1.0 glycerone_phosphate  # source: MetaCyc,
BRENDA, KEGG (queried 4.1.2.13)
glycolysis-I/p4_5.3.1.1_TRIOSEPISOMERIZATION-RXN            : 1.0 D-glyceraldehyde_3-phosphate <=> 1.0 glycerone_phosphate  # source: MetaCyc, BRENDA, KEGG (queried 5.3.1.1)
glycolysis-I/4_1.2.1.12_GAPDHSYNEC-RXN_WOP                  : 1.0 D-glyceraldehyde_3-phosphate + 1.0 phosphate + 1.0 NAD+ <=> 1.0 1,3-bisphospho-D-glycerate + 1.0 NADH + 1.0 H+  #
ource: MetaCyc, BRENDA, KEGG (queried 1.2.1.12) // other EC numbers for this reaction: 1.2.1.13, 1.2.1.59
glycolysis-I/5_2.7.2.3_PHOSGLYPHOS-RXN                      : 1.0 3-phospho-D-glycerate + 1.0 ATP <=> 1.0 ADP + 1.0 1,3-bisphospho-D-glycerate  # source: MetaCyc, BRENDA, KEGG
(queried 2.7.2.3)
glycolysis-I/6_5.4.2.12_3PGAREARR-RXN                       : 1.0 2-phospho-D-glycerate <=> 1.0 3-phospho-D-glycerate  # source: MetaCyc, BRENDA, KEGG (queried 5.4.2.12) // other
EC numbers for this reaction: 5.4.2.11
glycolysis-I/7_4.2.1.11_2PGADEHYDRAT-RXN                    : 1.0 2-phospho-D-glycerate <=> 1.0 H2O + 1.0 phosphoenolpyruvate  # source: MetaCyc, BRENDA, KEGG (queried 4.2.1.11)
glycolysis-I/8_2.7.1.40_PEPDEPHOS-RXN                       : 1.0 ATP + 1.0 pyruvate <=> 1.0 phosphoenolpyruvate + 1.0 ADP + 1.0 H+  # source: MetaCyc, BRENDA, KEGG (queried
2.7.1.40)

#glycolysis IV
glycolysis-IV/2c_2.7.1.90_2.7.1.90-RXN                      : 1.0 diphosphate + 1.0 beta-D-fructofuranose_6-phosphate <=> 1.0 fructose_1,6-bisphosphate + 1.0 phosphate + 1.0 H+  #
source: MetaCyc, BRENDA, KEGG (queried 2.7.1.90)
glycolysis-IV/4b_1.2.1.9_1.2.1.9-RXN                        : 1.0 NADP+ + 1.0 H2O + 1.0 D-glyceraldehyde_3-phosphate --> 1.0 3-phospho-D-glycerate + 2.0 H+ + 1.0 NADPH  # source:
MetaCyc, BRENDA, KEGG (queried 1.2.1.9) // other EC numbers for this reaction: 1.2.1.90
#
glycolysis-C/8b_2.7.9.1_PYRUVATEORTHOPHOSPHATE-DIKINASE-RXN : 1.0 phosphate + 1.0 ATP + 1.0 pyruvate --> 1.0 diphosphate + 1.0 phosphoenolpyruvate + 1.0 H+ + 1.0 AMP  # source:
MetaCyc, BRENDA, KEGG (queried 2.7.9.1)
#######################################################################################################################################################################
#methylglyoxal-degradation-I
#S-lactoyl-glutathione not found in all tested Clostridia (Fahey et al., 1978) and no 6.3.2.2/3 found in Cdiff; probably other coenzyme

methylglyoxal-degradation-I/p1_4.2.3.3_METHGLYSYN-RXN       : 1.0 glycerone_phosphate --> 1.0 methylglyoxal + 1.0 phosphate  # source: MetaCyc, BRENDA, KEGG (queried 4.2.3.3)
methylglyoxal-degradation-I/1_4.4.1.5_GLYOXI-RXN            : 1.0 (R)-S-lactoylglutathione <=> 1.0 methylglyoxal + 1.0 glutathione  # source: MetaCyc, BRENDA, KEGG (queried
4.4.1.5)
methylglyoxal-degradation-I/2_3.1.2.6_GLYOXII-RXN           : 1.0 H2O + 1.0 (R)-S-lactoylglutathione --> 1.0 glutathione + 1.0 (R)-lactate + 1.0 H+  # source: MetaCyc, BRENDA,
KEGG (queried 3.1.2.6)
#######################################################################################################################################################################
#Wood-Ljungdahl
#Pathway can act in the reverse direction (Spormann and Thauer, 1988, Schauder et al., 1988)

#Reversible in C. cylindrosporum (Curthoys and Rabinowitz, 1972)
Wood-Ljungdahl/1_moddir_6.3.4.3_FORMATETHFLIG-RXN           : 1.0 formate + 1.0 ATP + 1.0 tetrahydropteroyl_tri-L-glutamate <=> 1.0 ADP + 1.0 10-formyltetrahydrofolate + 1.0
phosphate  # source: MetaCyc (queried 6.3.4.3)
```

```
Wood-Ljungdahl/2_moddir_3.5.4.9_METHENYLTHFCYCLOHYDRO-RXN   : 1.0 5,10-methenyltetrahydrofolate + 1.0 H2O <=> 1.0 H+ + 1.0 10-formyltetrahydrofolate  # source: MetaCyc, BRENDA,
KEGG (queried 3.5.4.9)
Wood-Ljungdahl/3_1.5.1.5_METHYLENETHFDEHYDROG-NADP-RXN      : 1.0 5,10-methylene-tetrahydrofolate + 1.0 NADP+ <=> 1.0 5,10-methenyltetrahydrofolate + 1.0 NADPH  # source: MetaCyc,
BRENDA, KEGG (queried 1.5.1.5)
#Bifurcating methylene-tetrahydrofolate reductase as proposed C. ljungdahlii and C. autoethanogenum (Köpke et al. 2010, Nagarajan et al. 2013, Mock et al. 2015)
Wood-Ljungdahl/4_manual_1.5.-_HD-MetFV                      : 1.0 N5-methyl-tetrahydrofolate  + 2.0 |Reduced-ferredoxins| + 2.0 NAD+ <-- 1.0 5,10-methylene-tetrahydrofolate + 2.0
NADH + 2.0 |Oxidized-ferredoxins|
Wood-Ljungdahl/5_2.1.1.258_METHCOCLTH-RXN                   : 1.0 |Methylated-corrinoid-Fe-S-Proteins| + 1.0 tetrahydropteroyl_tri-L-glutamate <=> 1.0 H+ + 1.0 |CoI-Corrinoid-Fe-
S-proteins| + 1.0 N5-methyl-tetrahydrofolate  # source: MetaCyc (queried 2.1.1.258)
#CDIF630erm_00832: 1.2.7.4 in Wood-Ljungdahl-Operon; production of CO with Ferredoxins used; only <-- because of CO degradation (see below)
Wood-Ljungdahl/p6_moddir_1.2.7.4_1.2.7.4-RXN                : 1.0 H2O + 1.0 carbon_monoxide + 2.0 |Oxidized-ferredoxins| <-- 2.0 H+ + 1.0 CO2 + 2.0 |Reduced-ferredoxins|  #
source: MetaCyc, KEGG (queried 1.2.7.4) // other EC numbers for this reaction: 1.2.99.2
Wood-Ljungdahl/6_2.3.1.169_ACETYLSYNCLTH-RXN                : 1.0 H+ + 1.0 acetyl-CoA + 1.0 |CoI-Corrinoid-Fe-S-proteins| <=> 1.0 |Methylated-corrinoid-Fe-S-Proteins| + 1.0
coenzyme_A + 1.0 carbon_monoxide  # source: MetaCyc, BRENDA (queried 2.3.1.169)
##########################################################################################################################################################################################
#CO degradation C (CDIF630erm_00296-CDIF630erm_00298)
CO-drgradation-C_manual_HD-CO-DEHYD                         : 1.0 carbon_monoxide + 1.0 H2O + 1.0 NAD+ --> 1.0 CO2 + 1.0 NADH + 1.0 H+
##########################################################################################################################################################################################
#Dihydrogen
dihydrogen-production-A_moddir_1.12.7.2_HYDROG-RXN                : 2.0 |Oxidized-ferredoxins| + 1.0 H2 <-- 2.0 H+ + 2.0 |Reduced-ferredoxins|  # source: MetaCyc, KEGG (queried
1.12.7.2) // other EC numbers for this reaction: 1.12.99
#Homolog to the bifurcating hydrogenase of T. maritima (Schut and Adams, 2009)
dihydrogen-production-B_manual_1.12.1.-_HD-Bifur-Hyd              : 2.0 |Oxidized-ferredoxins| + 2.0 H2 + 1.0 NADP+ <=> 3.0 H+ + 1.0 NADPH + 2.0 |Reduced-ferredoxins|
#CDIF630erm_03614-CDIF630erm_03619 (Calusinska et al. 2010)
dihydrogen-production-C_manual_1.1.99.33_1.12.1.3_HD-Form-Hyd     : 1.0 H+ + 1.0 formate --> 1.0 CO2 + 1.0 H2
##########################################################################################################################################################################################
#ppp non-ox
ppp-non-ox/1a_5.3.1.6_RIB5PISOM-RXN                         : 1.0 D-ribose_5-phosphate <=> 1.0 D-ribulose_5-phosphate  # source: MetaCyc, BRENDA, KEGG (queried 5.3.1.6)
ppp-non-ox/1b_5.1.3.1_RIBULP3EPIM-RXN                       : 1.0 D-ribulose_5-phosphate <=> 1.0 D-xylulose_5-phosphate  # source: MetaCyc, BRENDA, KEGG (queried 5.1.3.1)
ppp-non-ox/2_2.2.1.1_R06863                                 : 1.0 thiamine_diphosphate + 1.0 D-sedoheptulose_7-phosphate <=> 1.0 D-ribose_5-phosphate + 1.0 alpha,beta-
Dihydroxyethyl-TPP  # source: KEGG (queried 2.2.1.1)
ppp-non-ox/3_2.2.1.1_R06861                                 : 1.0 D-xylulose_5-phosphate + 1.0 thiamine_diphosphate <=> 1.0 D-glyceraldehyde_3-phosphate + 1.0 alpha,beta-
Dihydroxyethyl-TPP  # source: KEGG (queried 2.2.1.1) // other EC numbers for this reaction: 2.2.1.3
ppp-non-ox/4_2.2.1.2_TRANSALDOL-RXN                         : 1.0 D-glyceraldehyde_3-phosphate + 1.0 D-sedoheptulose_7-phosphate <=> 1.0 beta-D-fructofuranose_6-phosphate + 1.0 D-
erythrose_4-phosphate  # source: MetaCyc, BRENDA, KEGG (queried 2.2.1.2)
ppp-non-ox/5_2.2.1.1_2TRANSKETO-RXN                         : 1.0 D-xylulose_5-phosphate + 1.0 D-erythrose_4-phosphate <=> 1.0 D-glyceraldehyde_3-phosphate + 1.0 beta-D-
fructofuranose_6-phosphate  # source: MetaCyc, BRENDA, KEGG (queried 2.2.1.1) // other EC numbers for this reaction: 2.2.1.3

PRPP-biosynthesis-I_2.7.6.1_PRPPSYN-RXN                     : 1.0 D-ribose_5-phosphate + 1.0 ATP <=> 1.0 H+ + 1.0 AMP + 1.0 5-phospho-alpha-D-ribose_1-diphosphate  # source:
MetaCyc, BRENDA, KEGG (queried 2.7.6.1)

#side reactions
sedoheptulose-bisphosphate-bypass_2.7.1.11_RXN0-6541                        : 1.0 D-sedoheptulose_7-phosphate + 1.0 ATP --> 1.0 ADP + 1.0 D-sedoheptulose-1,7-bisphosphate + 1.0 H+
# source: MetaCyc, KEGG (queried 2.7.1.11) // other EC numbers for this reaction: 2.7.1
sedoheptulose-bisphosphate-bypass_4.1.2.13_SEDOBISALDOL-RXN                 : 1.0 glycerone_phosphate + 1.0 D-erythrose_4-phosphate <=> 1.0 D-sedoheptulose-1,7-bisphosphate  #
source: MetaCyc, BRENDA, KEGG (queried 4.1.2.13) // other EC numbers for this reaction: 4.1.2
Calvin-Benson-Bassham-cycle_3.1.3.11_SEDOHEPTULOSE-BISPHOSPHATASE-RXN       : 1.0 H2O + 1.0 D-sedoheptulose-1,7-bisphosphate --> 1.0 D-sedoheptulose_7-phosphate + 1.0 phosphate  #
source: MetaCyc, BRENDA, KEGG (queried 3.1.3.11) // other EC numbers for this reaction: 3.1.3.37
##########################################################################################################################################################################################
#TCA
TCA-cycle/1_4.2.1.1_CARBODEHYDRAT-RXN                        : 1.0 hydrogen_carbonate <=> 1.0 CO2 + 1.0 H2O  # source: MetaCyc, BRENDA, KEGG (queried 4.2.1.1)
TCA-cycle/2_6.4.1.1_PYRUVATE-CARBOXYLASE-RXN                 : 1.0 hydrogen_carbonate + 1.0 ATP + 1.0 pyruvate --> 1.0 phosphate + 1.0 oxaloacetate + 1.0 ADP + 1.0 H+  # source:
MetaCyc, BRENDA, KEGG (queried 6.4.1.1)
TCA-cycle/3_2.3.3.3_CITSYN-RXN                               : 1.0 H2O + 1.0 acetyl-CoA + 1.0 oxaloacetate --> 1.0 H+ + 1.0 coenzyme_A + 1.0 citrate  # source: MetaCyc, BRENDA,
KEGG (queried 2.3.3.3) // other EC numbers for this reaction: 2.3.3.16, 2.3.3.1
TCA-cycle/4_4.2.1.3_ACONITATEDEHYDR-RXN                      : 1.0 citrate <=> 1.0 H2O + 1.0 cis-aconitate  # source: MetaCyc, BRENDA, KEGG (queried 4.2.1.3)
TCA-cycle/5_4.2.1.3_ACONITATEHYDR-RXN                        : 1.0 cis-aconitate + 1.0 H2O <=> 1.0 D-threo-isocitrate  # source: MetaCyc, BRENDA, KEGG (queried 4.2.1.3)
TCA-cycle/6_1.1.1.41_ISOCITRATE-DEHYDROGENASE-NAD+-RXN       : 1.0 D-threo-isocitrate + 1.0 NAD+ --> 1.0 NADH + 1.0 2-oxoglutarate + 1.0 CO2  # source: MetaCyc, BRENDA, KEGG
(queried 1.1.1.41) // other EC numbers for this reaction: 1.1.1.286

TCA-cycle/7_4.2.1.2_FUMHYDR-RXN                              : 1.0 (S)-malate <=> 1.0 H2O + 1.0 fumarate  # source: MetaCyc, BRENDA, KEGG (queried 4.2.1.2)
TCA-cycle/8_1.1.1.38_1.1.1.39-RXN                            : 1.0 (S)-malate + 1.0 NAD+ --> 1.0 NADH + 1.0 CO2 + 1.0 pyruvate  # source: MetaCyc, BRENDA, KEGG (queried
1.1.1.38) // other EC numbers for this reaction: 1.1.1.39, 1.3.1.7
TCA-cycle/9_1.1.1.38_OXALODECARB-RXN                         : 1.0 H+ + 1.0 oxaloacetate --> 1.0 CO2 + 1.0 pyruvate  # source: MetaCyc, BRENDA, KEGG (queried 1.1.1.38) // other EC
```

numbers for this reaction: 1.1.1.40, 4.1.1.3
##
#Fatty acids I

#C. kluyveri complex characterized (Wang et al., 2010)
NADP-reduction_manual_HD-NfnAB : 2.0 |Reduced-ferredoxins| + 1.0 NADH + 2.0 NADP+ + 1.0 H+ <=> 2.0 |Oxidized-ferredoxins| + 1.0 NAD+ + 2.0 NADPH

fatty-acid-I/1_6.4.1.2_ACETYL-COA-CARBOXYLTRANSFER-RXN : 1.0 hydrogen_carbonate + 1.0 ATP + 1.0 acetyl-CoA --> 1.0 ADP + 1.0 H+ + 1.0 phosphate + 1.0 malonyl-CoA # source:
MetaCyc, BRENDA, KEGG (queried 6.4.1.2)
fatty-acid-I/2_2.3.1.39_MALONYL-COA-ACP-TRANSACYL-RXN : 1.0 malonyl-CoA + 1.0 Acyl-carrier_protein <=> 1.0 coenzyme_A + 1.0 Malonyl-[acyl-carrier_protein] # source:
MetaCyc, BRENDA, KEGG (queried 2.3.1.39) // other EC numbers for this reaction: 2.3.1, 2.3.1.86, 2.3.1.85

#chain lenght: 16.246
#unsaturated: 0.164
#from C. difficile NCIB 10666 (Elsden et al., 1980)
#2.3.1.41/179/180/n2_1.1.1.100_1.3.1.9_4.2.1.59
fatty-acid-I/3a_manual_HD-acet : 1.0 acetyl_phosphate + 7.123 Malonyl-[acyl-carrier_protein] + 21.205 H+ + 14.082 NADPH --> 1.0 Acyl-[acyl-
carrier_protein] + 6.123 Acyl-carrier_protein + 7.123 H2O + 7.123 CO2 + 14.082 NADP+ + 1.0 phosphate
fatty-acid-I/3b_manual_HD-prop : 1.0 propanoyl_phosphate + 6.623 Malonyl-[acyl-carrier_protein] + 19.705 H+ + 13.082 NADPH --> 1.0 Acyl-[acyl-
carrier_protein] + 5.623 Acyl-carrier_protein + 6.623 H2O + 6.623 CO2 + 13.082 NADP+ + 1.0 phosphate
fatty-acid-I/3c_manual_HD-buta : 1.0 butanoyl_phosphate + 6.123 Malonyl-[acyl-carrier_protein] + 18.205 H+ + 12.082 NADPH --> 1.0 Acyl-[acyl-
carrier_protein] + 5.123 Acyl-carrier_protein + 6.123 H2O + 6.123 CO2 + 12.082 NADP+ + 1.0 phosphate
fatty-acid-I/3d_manual_HD-ibut : 1.0 isobutyryl_phosphate + 6.123 Malonyl-[acyl-carrier_protein] + 18.205 H+ + 12.082 NADPH --> 1.0 Acyl-[acyl-
carrier_protein] + 5.123 Acyl-carrier_protein + 6.123 H2O + 6.123 CO2 + 12.082 NADP+ + 1.0 phosphate
fatty-acid-I/3e_manual_HD-vale : 1.0 pentanoyl_phosphate + 5.623 Malonyl-[acyl-carrier_protein] + 16.705 H+ + 11.082 NADPH --> 1.0 Acyl-[acyl-
carrier_protein] + 4.623 Acyl-carrier_protein + 5.623 H2O + 5.623 CO2 + 11.082 NADP+ + 1.0 phosphate
fatty-acid-I/3f_manual_HD-ival : 1.0 isovaleryl_phosphate + 5.623 Malonyl-[acyl-carrier_protein] + 16.705 H+ + 11.082 NADPH --> 1.0 Acyl-[acyl-
carrier_protein] + 4.623 Acyl-carrier_protein + 5.623 H2O + 5.623 CO2 + 11.082 NADP+ + 1.0 phosphate
fatty-acid-I/3g_manual_HD-aval : 1.0 2-methylbutanoyl_phosphate + 5.623 Malonyl-[acyl-carrier_protein] + 16.705 H+ + 11.082 NADPH --> 1.0 Acyl-[acyl-
carrier_protein] + 4.623 Acyl-carrier_protein + 5.623 H2O + 5.623 CO2 + 11.082 NADP+ + 1.0 phosphate
fatty-acid-I/3h_manual_HD-icap : 1.0 isocaproyl_phosphate + 5.123 Malonyl-[acyl-carrier_protein] + 15.205 H+ + 10.082 NADPH --> 1.0 Acyl-[acyl-
carrier_protein] + 4.123 Acyl-carrier_protein + 5.123 H2O + 5.123 CO2 + 10.082 NADP+ + 1.0 phosphate
fatty-acid-I/3i_manual_HD-hexa : 1.0 hexanoyl_phosphate + 5.123 Malonyl-[acyl-carrier_protein] + 15.205 H+ + 10.082 NADPH --> 1.0 Acyl-[acyl-
carrier_protein] + 4.123 Acyl-carrier_protein + 5.123 H2O + 5.123 CO2 + 10.082 NADP+ + 1.0 phosphate

fatty-acid-I/4_manual_2.3.1.n2_HD-2.3.1.n2 : 1.0 Acyl-[acyl-carrier_protein] + 1.0 phosphate <=> 1.0 fatty_acid_phosphate + 1.0 Acyl-carrier_protein
##
#CDP-diacylglycerol-biosyn-I

CDP-diacylglycerol-biosyn-I/1_modnames_1.1.1.94_1.1.1.261-RXN : 1.0 sn-glycerol_3-phosphate + 1.0 NADP+ <-- 1.0 H+ + 1.0 glycerone_phosphate + 1.0 NADPH # source: MetaCyc,
BRENDA, KEGG (queried 1.1.1.94) // other EC numbers for this reaction: 1.1.1.261
CDP-diacylglycerol-biosyn-I/2_manual_2.3.1.n3_HD-2.3.1.n3 : 1.0 fatty_acid_phosphate + 1.0 sn-glycerol_3-phosphate <=> 1.0 1-acyl-sn-glycerol_3-phosphate + 1.0
phosphate
CDP-diacylglycerol-biosyn-I/3_2.3.1.51_BS321039 : 1.0 Acyl-[acyl-carrier_protein] + 1.0 1-acyl-sn-glycerol_3-phosphate <=> 1.0 1,2-diacyl-sn-glycerol_3-
phosphate + 1.0 Acyl-carrier_protein # source: MetaCyc, BRENDA, KEGG (queried 2.3.1.51)
CDP-diacylglycerol-biosyn-I/4_2.7.7.41_CDPDIGLYSYN-RXN : 1.0 1,2-diacyl-sn-glycerol_3-phosphate + 1.0 CTP + 1.0 H+ --> 1.0 diphosphate + 1.0 CDP-1,2-diacyl-sn-
glycerol # source: MetaCyc, BRENDA, KEGG (queried 2.7.7.41)
##
#cardiolipin biosynthesis I
cardiolipin-biosyn-I/1_2.7.8.5_PHOSPHAGLYPSYN-RXN : 1.0 CDP-1,2-diacyl-sn-glycerol + 1.0 sn-glycerol_3-phosphate <=> 1.0 H+ + 1.0 CMP + 1.0
Phosphatidylglycerophosphate # source: MetaCyc, BRENDA, KEGG (queried 2.7.8.5)
cardiolipin-biosyn-I/2_3.1.3.27_PGPPHOSPHA-RXN : 1.0 Phosphatidylglycerophosphate + 1.0 H2O --> 1.0 phosphate + 1.0 Phosphatidylglycerol # source: MetaCyc,
BRENDA, KEGG (queried 3.1.3.27)
cardiolipin-biosyn-I/3_2.7.8.-_CARDIOLIPSYN-RXN : 2.0 Phosphatidylglycerol --> 1.0 a_cardiolipin + 1.0 glycerol # source: MetaCyc, BRENDA, KEGG (queried
2.7.8.-) // other EC numbers for this reaction: 2.7.8.B10, 2.7.8
##
#glycerol degradation I

glycerol-degradation-I_2.7.1.30_GLYCEROL-KIN-RXN : 1.0 glycerol + 1.0 ATP --> 1.0 ADP + 1.0 sn-glycerol_3-phosphate + 1.0 H+ # source: MetaCyc, BRENDA, KEGG
(queried 2.7.1.30)
##
#UDP-N-acetyl-D-glucosamine biosynthesis I

UDP-N-acetyl-D-glucosamine-biosyn-I/1_2.6.1.16_L-GLN-FRUCT-6-P-AMINOTRANS-RXN : 1.0 L-glutamine + 1.0 beta-D-fructofuranose_6-phosphate <=> 1.0 D-glucosamine_6-phosphate + 1.0

```
L-glutamate  # source: MetaCyc, BRENDA, KEGG (queried 2.6.1.16)
UDP-N-acetyl-D-glucosamine-biosyn-I/2_5.4.2.10_5.4.2.10-RXN                       : 1.0 D-glucosamine_6-phosphate <=> 1.0 D-glucosamine_1-phosphate  # source: MetaCyc, BRENDA, KEGG (queried 5.4.2.10)
UDP-N-acetyl-D-glucosamine-biosyn-I/3_2.3.1.157_2.3.1.157-RXN                     : 1.0 acetyl-CoA + 1.0 D-glucosamine_1-phosphate --> 1.0 N-acetyl-alpha-D-glucosamine_1-phosphate + 1.0 coenzyme_A + 1.0 H+  # source: MetaCyc, BRENDA, KEGG (queried 2.3.1.157)
UDP-N-acetyl-D-glucosamine-biosyn-I/4_2.7.7.23_NAG1P-URIDYLTRANS-RXN              : 1.0 H+ + 1.0 UTP + 1.0 N-acetyl-alpha-D-glucosamine_1-phosphate --> 1.0 UDP-N-acetyl-alpha-D-glucosamine + 1.0 diphosphate  # source: MetaCyc, BRENDA, KEGG (queried 2.7.7.23)
###########################################################################################################################################################################
#UDP-N-acetyl-D-galactosamine biosynthesis I
UDP-N-acetyl-D-galactosamine-biosyn-I_5.1.3.2_UDP-N-ACETYLGLUCOSAMINE-4-EPIMERASE-RXN      : 1.0 UDP-N-acetyl-alpha-D-glucosamine <=> 1.0 UDP-N-acetyl-alpha-D-galactosamine  # source: MetaCyc, BRENDA, KEGG (queried 5.1.3.2) // other EC numbers for this reaction: 5.1.3.7
###########################################################################################################################################################################
#GDP-mannose biosyn

GDP-mannose-biosyn/1_5.3.1.8_MANNPISOM-RXN                                         : 1.0 D-mannopyranose_6-phosphate <=> 1.0 beta-D-fructofuranose_6-phosphate  # source: MetaCyc (queried 5.3.1.8)
GDP-mannose-biosyn/2_5.4.2.8_PHOSMANMUT-RXN                                        : 1.0 alpha-D-mannose_1-phosphate <=> 1.0 D-mannopyranose_6-phosphate  # source: MetaCyc (queried 5.4.2.8)
GDP-mannose-biosyn/3_2.7.7.13_2.7.7.13-RXN                                         : 1.0 alpha-D-mannose_1-phosphate + 1.0 H+ + 1.0 GTP --> 1.0 GDP-alpha-D-mannose + 1.0 diphosphate  # source: MetaCyc, BRENDA, KEGG (queried 2.7.7.13) // other EC numbers for this reaction: 2.7.7.34, 2.7.7.30
###########################################################################################################################################################################
#UDP-glucose biosyn
UDP-glucose-biosyn/1_5.4.2.2_5.4.2.8_PHOSPHOGLUCMUT-RXN                            : 1.0 alpha-D-glucopyranose_1-phosphate <=> 1.0 D-glucose_6-phosphate  # source: MetaCyc, BRENDA, KEGG (queried 5.4.2.2, 5.4.2.8) // other EC numbers for this reaction: 5.4.2.5
UDP-glucose-biosyn/2_2.7.7.9_GLUC1PURIDYLTRANS-RXN                                 : 1.0 UTP + 1.0 alpha-D-glucopyranose_1-phosphate + 1.0 H+ <=> 1.0 UDP-alpha-D-glucose + 1.0 diphosphate  # source: MetaCyc, BRENDA, KEGG (queried 2.7.7.9) // other EC numbers for this reaction: 2.7.7.64
###########################################################################################################################################################################
#peptidoglycan biosynthesis I (meso-diaminopimelate containing)

peptidoglycan-biosyn-I/1_2.5.1.7_UDPNACETYLGLUCOSAMENOLPYRTRANS-RXN                : 1.0 UDP-N-acetyl-alpha-D-glucosamine + 1.0 phosphoenolpyruvate --> 1.0 phosphate + 1.0 UDP-N-acetyl-alpha-D-glucosamine-enolpyruvate  # source: MetaCyc, BRENDA, KEGG (queried 2.5.1.7)
peptidoglycan-biosyn-I/2_1.3.1.98_UDPNACETYLMURAMATEDEHYDROG-RXN                   : 1.0 NADP+ + 1.0 UDP-N-acetyl-alpha-D-muramate <-- 1.0 H+ + 1.0 NADPH + 1.0 UDP-N-acetyl-alpha-D-glucosamine-enolpyruvate  # source: MetaCyc, BRENDA, KEGG (queried 1.3.1.98)
peptidoglycan-biosyn-I/3_6.3.2.8_UDP-NACMUR-ALA-LIG-RXN                            : 1.0 L-alanine + 1.0 ATP + 1.0 UDP-N-acetyl-alpha-D-muramate --> 1.0 phosphate + 1.0 UDP-N-acetyl-alpha-D-muramoyl-L-alanine + 1.0 ADP + 1.0 H+  # source: MetaCyc, BRENDA, KEGG (queried 6.3.2.8)

D-glutamate_5.1.1.3_GLUTRACE-RXN                                                   : 1.0 L-glutamate <=> 1.0 D-glutamate  # source: MetaCyc, BRENDA, KEGG (queried 5.1.1.3)

peptidoglycan-biosyn-I/4_6.3.2.9_UDP-NACMURALA-GLU-LIG-RXN                         : 1.0 D-glutamate + 1.0 UDP-N-acetyl-alpha-D-muramoyl-L-alanine + 1.0 ATP --> 1.0 phosphate + 1.0 ADP + 1.0 UDP-N-acetyl-alpha-D-muramoyl-L-alanyl-D-glutamate + 1.0 H+  # source: MetaCyc, BRENDA, KEGG (queried 6.3.2.9)
peptidoglycan-biosyn-I/5_6.3.2.13_UDP-NACMURALGLDAPLIG-RXN                         : 1.0 UDP-N-acetyl-alpha-D-muramoyl-L-alanyl-D-glutamate + 1.0 meso-diaminopimelate + 1.0 ATP --> 1.0 UDP-N-acetyl-alpha-D-muramoyl-L-alanyl-gamma-D-glutamyl-meso-2,6-diaminopimelate + 1.0 H+ + 1.0 phosphate + 1.0 ADP  # source: MetaCyc, BRENDA, KEGG (queried 6.3.2.13)

D-alanyl-D-alanine/1_5.1.1.1_ALARACECAT-RXN                                        : 1.0 L-alanine <=> 1.0 D-alanine  # source: MetaCyc, BRENDA, KEGG (queried 5.1.1.1)
D-alanyl-D-alanine/2_6.3.2.4_DALADALALIG-RXN                                       : 2.0 D-alanine + 1.0 ATP --> 1.0 phosphate + 1.0 D-alanyl-D-alanine + 1.0 ADP + 1.0 H+  # source: MetaCyc, BRENDA, KEGG (queried 6.3.2.4)

peptidoglycan-biosyn-I/6_6.3.2.10_UDP-NACMURALGLDAPAALIG-RXN                       : 1.0 UDP-N-acetyl-alpha-D-muramoyl-L-alanyl-gamma-D-glutamyl-meso-2,6-diaminopimelate + 1.0 D-alanyl-D-alanine + 1.0 ATP --> 1.0 H+ + 1.0 phosphate + 1.0 UDP-N-acetyl-alpha-D-muramoyl-L-alanyl-gamma-D-glutamyl-meso-2,6-diaminopimeloyl-D-alanyl-D-alanine + 1.0 ADP  # source: MetaCyc, KEGG (queried 6.3.2.10)
peptidoglycan-biosyn-I/p7_3.6.1.27_UNDECAPRENYL-DIPHOSPHATASE-RXN                  : 1.0 H2O + 1.0 di-trans,octa-cis-undecaprenyl_diphosphate --> 1.0 H+ + 1.0 di-trans,octa-cis-undecaprenyl_phosphate + 1.0 phosphate  # source: MetaCyc, BRENDA, KEGG (queried 3.6.1.27)
peptidoglycan-biosyn-I/7_2.7.8.13_PHOSNACMURPENTATRANS-RXN                         : 1.0 di-trans,octa-cis-undecaprenyl_phosphate + 1.0 UDP-N-acetyl-alpha-D-muramoyl-L-alanyl-gamma-D-glutamyl-meso-2,6-diaminopimeloyl-D-alanyl-D-alanine --> 1.0 undecaprenyldiphospho-N-acetylmuramoyl-L-alanyl-gamma-D-glutamyl-meso-2,6-diaminopimeloyl-D-alanyl-D-alanine + 1.0 UMP  # source: MetaCyc, KEGG (queried 2.7.8.13)
peptidoglycan-biosyn-I/8_2.4.1.227_NACGLCTRANS-RXN                                 : 1.0 undecaprenyldiphospho-N-acetylmuramoyl-L-alanyl-gamma-D-glutamyl-meso-2,6-diaminopimeloyl-D-alanyl-D-alanine + 1.0 UDP-N-acetyl-alpha-D-glucosamine <=> 1.0 ditrans,octacis-undecaprenyldiphospho-N-acetyl-(N-acetylglucosaminyl)muramoyl-L-alanyl-gamma-D-glutamyl-meso-2,6-diaminopimeloyl-D-alanyl-D-alanine + 1.0 H+ + 1.0 UDP  # source: MetaCyc, KEGG (queried 2.4.1.227)

#Based on HPLC-MS-studies (Peltier et al., 2011)
#Gly: -0.0453
#meso-DAP: +0.0035
#D-Ala: 2-0.6109 = +1.3891
#Cross: 0.3107
```

```
#deAc: 0.9278
#H2O: -deAc -meso-DAP -D-Ala + Cross - Gly = -1.9644
peptidoglycan-biosyn-I/10_manual_3.4.16.4_3.5.1.104_HD-murein-mods                : 1.0 ditrans,octacis-undecaprenyldiphospho-N-acetyl-(N-acetylglucosaminyl)muramoyl-L-alanyl-gamma-D-glutamyl-meso-2,6-diaminopimeloyl-D-alanyl-D-alanine + 1.9644 H2O + 0.0453 glycine + 1.3891 D-alanine --> 0.9278 acetate + 0.0035 meso-diaminopimelate + 1.0 di-trans,octa-cis-undecaprenyl_diphosphate + 1.0 H+ + 1.0 a_peptidoglycan
##########################################################################################################################################################################
#peptidoglycan degradation
#H2O: 2 - Anh - acetate + 1 + glycine = 2.114
#1x ABC transport (appABCF)
peptidoglycan-degr/1_manual_3.5.1.28_3.2.1.96_3.5.1.104_HD-appABCF                : 1.0 a_peptidoglycan + 0.9278 acetate + 3.114 H2O + 1.0 ATP --> 0.0035 1,6-anhydro-N-acetyl-beta-muramate + 0.9965 N-acetylmuramate + 1.0 N-acetyl-beta-D-glucosamine + 0.6109 L-alanyl-gamma-D-glutamyl-meso-2,6-diaminopimeloyl-D-alanine + 0.3856 L-alanyl-gamma-D-glutamyl-meso-diaminopimelate + 0.0035 L-alanyl-D-glutamate + 0.0453 glycine + 1.0 ADP + 1.0 H+ + 1.0 phosphate
peptidoglycan-degr/2a_2.7.1.170_RXN0-4621                                         : 1.0 1,6-anhydro-N-acetyl-beta-muramate + 1.0 ATP + 1.0 H2O --> 1.0 H+ + 1.0 N-acetyl-beta-muramate_6-phosphate + 1.0 ADP  # source: MetaCyc, BRENDA, KEGG (queried 2.7.1.170)
#Additional reaction of EC 2.7.1.59 shown for C. acetobutylicum (Reith et al., 2011)
peptidoglycan-degr/2b_manual_2.7.1.59_HD-2.7.1.59                                 : 1.0 N-acetylmuramate + 1.0 ATP --> 1.0 ADP + 1.0 H+ + 1.0 N-acetyl-beta-muramate_6-phosphate
peptidoglycan-degr/3_4.2.1.126_RXN0-4641                                          : 1.0 H2O + 1.0 N-acetyl-beta-muramate_6-phosphate <=> 1.0 N-acetyl-D-glucosamine_6-phosphate + 1.0 (R)-lactate  # source: MetaCyc (queried 4.2.1.126)
peptidoglycan-degr/4_2.7.1.59_N-ACETYLGLUCOSAMINE-KINASE-RXN                      : 1.0 N-acetyl-beta-D-glucosamine + 1.0 ATP --> 1.0 ADP + 1.0 H+ + 1.0 N-acetyl-D-glucosamine_6-phosphate  # source: MetaCyc, BRENDA, KEGG (queried 2.7.1.59)

#N-acetylglucosamine degradation I
N-acetylglucosamine-degr-I/1_3.5.1.25_NAG6PDEACET-RXN                             : 1.0 H2O + 1.0 N-acetyl-D-glucosamine_6-phosphate --> 1.0 acetate + 1.0 D-glucosamine_6-phosphate  # source: MetaCyc, BRENDA, KEGG (queried 3.5.1.25)
N-acetylglucosamine-degr-I/2_3.5.99.6_BS363214                                    : 1.0 H2O + 1.0 D-glucosamine_6-phosphate --> 1.0 ammonia + 1.0 beta-D-fructofuranose_6-phosphate  # source: BRENDA, KEGG (queried 3.5.99.6)

#muropeptide degradation
muropeptide-degr/1_3.4.17.13_RXN0-5227                                            : 1.0 H2O + 1.0 L-alanyl-gamma-D-glutamyl-meso-2,6-diaminopimeloyl-D-alanine --> 1.0 L-alanyl-gamma-D-glutamyl-meso-diaminopimelate + 1.0 D-alanine  # source: MetaCyc (queried 3.4.17.13)
muropeptide-degr/2_RXN0-961                                                       : 1.0 L-alanyl-gamma-D-glutamyl-meso-diaminopimelate + 1.0 H2O --> 1.0 L-alanyl-D-glutamate
muropeptide-degr/3_5.1.1.20_RXN0-5228                                             : 1.0 L-alanyl-D-glutamate <=> 1.0 L-alanyl-L-glutamate  # source: MetaCyc, BRENDA, KEGG (queried 5.1.1.20)
muropeptide-degr/4_3.4.13.18_RXN0-6981                                            : 1.0 H2O + 1.0 L-alanyl-L-glutamate --> 1.0 L-glutamate + 1.0 L-alanine  # source: MetaCyc, BRENDA (queried 3.4.13.18) // other EC numbers for this reaction: 3.4.11.14
##########################################################################################################################################################################
#L-CWG biosynthesis
L-CWG-biosyn/1a_3.1.3.4_PHOSPHATIDATE-PHOSPHATASE-RXN                          : 1.0 1,2-diacyl-sn-glycerol_3-phosphate + 1.0 H2O --> 1.0 1,2-diacyl-sn-glycerol + 1.0 phosphate  # source: MetaCyc, BRENDA, KEGG (queried 3.1.3.4) // other EC numbers for this reaction: 3.1.3.81
L-CWG-biosyn/1b_2.7.1.107_DIACYLGLYKIN-RXN                                     : 1.0 ATP + 1.0 1,2-diacyl-sn-glycerol --> 1.0 H+ + 1.0 ADP + 1.0 1,2-diacyl-sn-glycerol_3-phosphate  # source: MetaCyc, BRENDA, KEGG (queried 2.7.1.107)
L-CWG-biosyn/2_2.4.1.315_2.4.1.337_2.4.1.157-RXN                               : 1.0 1,2-diacyl-sn-glycerol + 1.0 UDP-alpha-D-glucose --> 1.0 3-D-Glucosyl-1,2-diacylglycerol + 1.0 H+ + 1.0 UDP  # source: MetaCyc, BRENDA, KEGG (queried 2.4.1.315, 2.4.1.337) // other EC numbers for this reaction: 2.4.1.336, 2.4.1.157, 2.4.1.cp
L-CWG-biosyn/3_2.4.1.315_RXN-15117                                             : 1.0 3-D-Glucosyl-1,2-diacylglycerol + 1.0 UDP-alpha-D-glucose <=> 1.0 |diacyl-3-O-glucl-1-6-gluc-sn-glycerol| + 1.0 UDP + 1.0 H+  # source: MetaCyc (queried 2.4.1.315)
L-CWG-biosyn/4_2.4.1.315_RXN-15118                                             : 1.0 |diacyl-3-O-glucl-1-6-gluc-sn-glycerol| + 1.0 UDP-alpha-D-glucose <=> 1.0 H+ + 1.0 |diacyl-3-Glc-16-Glc-16-Glc-glycerol| + 1.0 UDP  # source: MetaCyc (queried 2.4.1.315)
##########################################################################################################################################################################
#Flagellin modification (Faulds-Pain et al. 2014)
flagellin-modification/1_manual_2.4.-_HD-fla1                                  : 1.0 UDP-N-acetyl-alpha-D-glucosamine --> 1.0 UDP + 1.0 H+ + 1.0 GluNAc-FliC
flagellin-modification/p1_manual_3.1.3.-_HD-fla2                               : 1.0 L-threonine + 1.0 phosphate <=> 1.0 H2O + 1.0 3-phospho-L-threonine
flagellin-modification/2_manual_3.1.4.-_HD-fla3                                : 1.0 GluNAc-FliC + 1.0 3-phospho-L-threonine + 1.0 H+ --> 1.0 H2O + 1.0 Thr-P-GluNAc-FliC
flagellin-modification/3_manual_2.1.1.-_HD-fla4                                : 1.0 Thr-P-GluNAc-FliC + 1.0 S-adenosyl-L-methionine --> 1.0 Me-Thr-P-GluNAc-FliC + 2.0 H+ + 1.0 S-adenosyl-L-homocysteine
##########################################################################################################################################################################
#glycogen biosynthesis I + degradation

glycogen-biosyn-I/1_2.7.7.27_GLUC1PADENYLTRANS-RXN                             : 1.0 H+ + 1.0 alpha-D-glucopyranose_1-phosphate + 1.0 ATP --> 1.0 diphosphate + 1.0 ADP-alpha-D-glucose  # source: MetaCyc, BRENDA, KEGG (queried 2.7.7.27)
glycogen-biosyn-I/2_manual_2.4.1.21_HD-2.4.1.21                                : 1.0 ADP-alpha-D-glucose --> 1.0 ADP + 1.0 |1-4-alpha-D-Glucan| + 1.0 H+
glycogen-biosyn-I/3_2.4.1.18_3.2.1.54_3.2.1.41_3.2.1.1_GLYCOGEN-BRANCH-RXN      : 1.0 |1-4-alpha-D-Glucan| --> 1.0 |Glycogens|  # source: MetaCyc, BRENDA (queried 2.4.1.18, 3.2.1.54, 3.2.1.41, 3.2.1.1) // other EC numbers for this reaction: 2.4.1.25, 3.2.1.68, 3.2.1.116

glycogen-degr_manual_3.2.1.54_3.2.1.41_3.2.1.1_HD-Glycogen                     : 3.0 |Glycogens| + 1.0 H2O --> 1.0 |Maltodextrins|  # source: MetaCyc, BRENDA (queried 2.4.1.18,
```

```
3.2.1.54, 3.2.1.41, 3.2.1.1) // other EC numbers for this reaction: 2.4.1.25, 3.2.1.68, 3.2.1.116
glycogen-degr_3.2.1.133_3.2.1.1_RXN-12188                                : 1.0 |Maltodextrins| + 1.0 H2O --> 1.0 D-glucose + 1.0 alpha,alpha-trehalose  # source: MetaCyc,
BRENDA (queried 3.2.1.133, 3.2.1.1) // there are 4 more EC numbers for this reaction
glycogen-degr_2.4.1.64_2.4.1.8_ALPHAALPHA-TREHALOSE-PHOSPHORYLASE-RXN           : 1.0 phosphate + 1.0 alpha,alpha-trehalose <=> 1.0 beta-D-glucose_1-phosphate + 1.0 D-glucose  #
source: MetaCyc, BRENDA, KEGG (queried 2.4.1.64, 2.4.1.8)
glycogen-degr_3.2.1.10_RXN-15910                                         : 1.0 alpha,alpha-trehalose + 1.0 H2O --> 2.0 D-glucose  # source: MetaCyc, BRENDA (queried
3.2.1.10) // other EC numbers for this reaction: 3.2.1.20

glucose-degr/1_2.7.1.2_GLUCOKIN-RXN                                      : 1.0 D-glucose + 1.0 ATP --> 1.0 D-glucose_6-phosphate + 1.0 H+ + 1.0 ADP  # source: MetaCyc,
BRENDA, KEGG (queried 2.7.1.2) // other EC numbers for this reaction: 2.7.1.1
glucose-degr/2_5.4.2.6_BETA-PHOSPHOGLUCOMUTASE-RXN                       : 1.0 beta-D-glucose_1-phosphate <=> 1.0 beta-D-glucose_6-phosphate  # source: MetaCyc, BRENDA,
KEGG (queried 5.4.2.6)
glucose-degr/3_5.3.1.9_R03321                                            : 1.0 beta-D-glucose_6-phosphate <=> 1.0 beta-D-fructofuranose_6-phosphate  # source: KEGG (queried
5.3.1.9)
######################################################################################################################################################################
#polymer biosynthesis
exopolysaccharide-biosynthesis/1_manual_2.4.1.12_HD-2.4.1.12             : 1.0 UDP-alpha-D-glucose --> 1.0 UDP + 1.0 cellulose + 1.0 H+
#1 ATP per glucose subunit
exopolysaccharide-biosynthesis/2_transport                               : 1.0 cellulose + 1.0 ATP + 1.0 H2O --> 1.0 cellulose[ex] + 1.0 ADP + 1.0 phosphate + 1.0 H+
#CDIF630erm_02795; 50 % acetylation
exopolysaccharide-biosynthesis/3_manual_2.3.1.-_HD-PSac                  : 1.0 cellulose[ex] + 0.5 acetyl-CoA <=> 0.5 coenzyme_A + 1.0 acetyl-cellulose[ex]
#trimming; chain-length: 100
exopolysaccharide-biosynthesis/4_manual_3.2.1.4_HD-3.2.1.4               : 1.0 acetyl-cellulose[ex] + 0.01 H2O --> 1.0 exopolysaccharide[ex]

exopolysaccharide_exchange                                               : exopolysaccharide[ex] -->
######################################################################################################################################################################
#glutamate/mine
glutamate_6.3.1.2_GLUTAMINESYN-RXN                                       : 1.0 ammonium + 1.0 L-glutamate + 1.0 ATP --> 1.0 L-glutamine + 1.0 ADP + 1.0 H+ + 1.0 phosphate
# source: MetaCyc, BRENDA, KEGG (queried 6.3.1.2)
glutamate_3.5.1.2_6.3.5.2_6.3.5.5_6.3.5.4_GLUTAMIN-RXN                   : 1.0 H2O + 1.0 L-glutamine --> 1.0 ammonium + 1.0 L-glutamate  # source: MetaCyc, BRENDA, KEGG
(queried 3.5.1.2, 6.3.5.2, 6.3.5.5, 6.3.5.4) // there are 6 more EC numbers for this reaction
glutamate_1.4.1.2_GLUTAMATE-DEHYDROGENASE-NADP+-RXN_WOP                  : 1.0 NAD+ + 1.0 H2O + 1.0 L-glutamate <=> 1.0 H+ + 1.0 NADH + 1.0 2-oxoglutarate + 1.0 ammonium  #
source: MetaCyc, BRENDA, KEGG (queried 1.4.1.2) // other EC numbers for this reaction: 1.4.1.3
######################################################################################################################################################################
#asp biosyn,asn biosyn I+II,asn degr. I
aspartate_2.6.1.1_ASPAMINOTRANS-RXN                                      : 1.0 2-oxoglutarate + 1.0 L-aspartate <=> 1.0 L-glutamate + 1.0 oxaloacetate  # source: MetaCyc,
BRENDA, KEGG (queried 2.6.1.1) // other EC numbers for this reaction: 2.6.1.39
aspartate_6.3.5.4_ASNSYNB-RXN                                            : 1.0 L-glutamine + 1.0 ATP + 1.0 H2O + 1.0 L-aspartate --> 1.0 L-asparagine + 1.0 AMP + 1.0 H+ +
1.0 diphosphate + 1.0 L-glutamate  # source: MetaCyc, BRENDA, KEGG (queried 6.3.5.4)
aspartate_6.3.1.1_6.3.5.4_ASNSYNA-RXN                                    : 1.0 ATP + 1.0 ammonium + 1.0 L-aspartate --> 1.0 L-asparagine + 1.0 AMP + 1.0 H+ + 1.0
diphosphate  # source: MetaCyc, BRENDA, KEGG (queried 6.3.1.1, 6.3.5.4)
aspartate_3.5.5.4_3.5.1.1_ASPARAGHYD-RXN                                 : 1.0 H2O + 1.0 L-asparagine --> 1.0 ammonium + 1.0 L-aspartate  # source: MetaCyc, BRENDA, KEGG
(queried 3.5.5.4, 3.5.1.1) // other EC numbers for this reaction: 3.5.1.38
######################################################################################################################################################################
#lys biosyn II
lysine-biosyn-II/1_2.7.2.4_ASPARTATEKIN-RXN                              : 1.0 ATP + 1.0 L-aspartate --> 1.0 ADP + 1.0 L-aspartyl-4-phosphate  # source: MetaCyc, BRENDA,
KEGG (queried 2.7.2.4)
lysine-biosyn-II/2_1.2.1.11_ASPARTATE-SEMIALDEHYDE-DEHYDROGENASE-RXN     : 1.0 NADP+ + 1.0 L-aspartate-semialdehyde + 1.0 phosphate <=> 1.0 L-aspartyl-4-phosphate + 1.0 H+
+ 1.0 NADPH  # source: MetaCyc, BRENDA, KEGG (queried 1.2.1.11)
lysine-biosyn-II/3_4.3.3.7_DIHYDRODIPICSYN-RXN                           : 1.0 pyruvate + 1.0 L-aspartate-semialdehyde --> 1.0 (2S,4S)-4-hydroxy-2,3,4,5-
tetrahydrodipicolinate + 1.0 H+ + 1.0 H2O  # source: MetaCyc, BRENDA, KEGG (queried 4.3.3.7)
lysine-biosyn-II/4_1.17.1.8_RXN-14014                                    : 1.0 (S)-2,3,4,5-tetrahydrodipicolinate + 1.0 H2O + 1.0 NADP+ <-- 1.0 H+ + 1.0 NADPH + 1.0
(2S,4S)-4-hydroxy-2,3,4,5-tetrahydrodipicolinate  # source: MetaCyc, BRENDA, KEGG (queried 1.17.1.8)
lysine-biosyn-II/5_2.3.1.89_2.3.1.89-RXN                                 : 1.0 H2O + 1.0 (S)-2,3,4,5-tetrahydrodipicolinate + 1.0 acetyl-CoA --> 1.0 coenzyme_A + 1.0 L-2-
acetamido-6-oxoheptanedioate  # source: MetaCyc, BRENDA, KEGG (queried 2.3.1.89) // other EC numbers for this reaction: 2.3.1
lysine-biosyn-II/6_2.6.1.-_RXN-4822                                      : 1.0 L-2-acetamido-6-oxoheptanedioate + 1.0 L-glutamate --> 1.0 2-oxoglutarate + 1.0 N-acetyl-L,L-
2,6-diaminopimelate  # source: MetaCyc, KEGG (queried 2.6.1.-) // other EC numbers for this reaction: 2.6.1
lysine-biosyn-II/7_3.5.1.47_N-ACETYLDIAMINOPIMELATE-DEACETYLASE-RXN      : 1.0 N-acetyl-L,L-2,6-diaminopimelate + 1.0 H2O --> 1.0 acetate + 1.0 L,L-diaminopimelate  #
source: MetaCyc, BRENDA, KEGG (queried 3.5.1.47)
lysine-biosyn-II/8_5.1.1.7_DIAMINOPIMEPIM-RXN                            : 1.0 L,L-diaminopimelate <=> 1.0 meso-diaminopimelate  # source: MetaCyc, BRENDA, KEGG (queried
5.1.1.7)
lysine-biosyn-II/9_4.1.1.20_DIAMINOPIMDECARB-RXN                         : 1.0 H+ + 1.0 meso-diaminopimelate --> 1.0 CO2 + 1.0 L-lysine  # source: MetaCyc, BRENDA, KEGG
(queried 4.1.1.20)
######################################################################################################################################################################
```

```
#arginine biosyn II
arginine-biosyn-II/1_2.3.1.1_N-ACETYLTRANSFER-RXN                               : 1.0 L-glutamate + 1.0 acetyl-CoA <=> 1.0 N-acetyl-L-glutamate + 1.0 coenzyme_A + 1.0 H+  #
source: MetaCyc, BRENDA, KEGG (queried 2.3.1.1)
arginine-biosyn-II/2_2.7.2.8_ACETYLGLUTKIN-RXN                                  : 1.0 ATP + 1.0 N-acetyl-L-glutamate --> 1.0 N-acetylglutamyl-phosphate + 1.0 ADP  # source:
MetaCyc, BRENDA, KEGG (queried 2.7.2.8)
arginine-biosyn-II/3_1.2.1.38_N-ACETYLGLUTPREDUCT-RXN                           : 1.0 phosphate + 1.0 NADP+ + 1.0 N-acetyl-L-glutamate_5-semialdehyde <=> 1.0 N-acetylglutamyl-
phosphate + 1.0 H+ + 1.0 NADPH  # source: MetaCyc, BRENDA, KEGG (queried 1.2.1.38)
arginine-biosyn-II/4_2.6.1.11_ACETYLORNTRANSAM-RXN                              : 1.0 N-acetyl-L-ornithine + 1.0 2-oxoglutarate <=> 1.0 N-acetyl-L-glutamate_5-semialdehyde + 1.0
L-glutamate  # source: MetaCyc, BRENDA, KEGG (queried 2.6.1.11)
arginine-biosyn-II/5_2.3.1.35_GLUTAMATE-N-ACETYLTRANSFERASE-RXN                 : 1.0 N-acetyl-L-ornithine + 1.0 L-glutamate --> 1.0 N-acetyl-L-glutamate + 1.0 L-ornithine  #
source: MetaCyc, BRENDA, KEGG (queried 2.3.1.35)
arginine-biosyn-II/p6_6.3.5.5_CARBPSYN-RXN                                      : 2.0 ATP + 1.0 H2O + 1.0 L-glutamine + 1.0 hydrogen_carbonate --> 2.0 ADP + 1.0 phosphate + 1.0 L-
glutamate + 1.0 carbamoyl_phosphate + 2.0 H+  # source: MetaCyc, BRENDA, KEGG (queried 6.3.5.5)
arginine-biosyn-II/6_2.1.3.3_ORNCARBAMTRANSFER-RXN                              : 1.0 carbamoyl_phosphate + 1.0 L-ornithine <=> 1.0 phosphate + 1.0 L-citrulline + 1.0 H+  #
source: MetaCyc, BRENDA, KEGG (queried 2.1.3.3) // other EC numbers for this reaction: 3.5.3.12
arginine-biosyn-II/7_6.3.4.5_ARGSUCCINSYN-RXN                                   : 1.0 L-citrulline + 1.0 L-aspartate + 1.0 ATP --> 1.0 diphosphate + 1.0 L-arginino-succinate + 1.0
AMP + 1.0 H+  # source: MetaCyc, BRENDA, KEGG (queried 6.3.4.5)
arginine-biosyn-II/8_4.3.2.1_ARGSUCCINLYA-RXN                                   : 1.0 L-arginino-succinate <=> 1.0 fumarate + 1.0 L-arginine  # source: MetaCyc, BRENDA, KEGG
(queried 4.3.2.1)
#############################################################################################################################################################################
#arginine degr
arginine-degradation/1_3.5.3.6_ARGININE-DEIMINASE-RXN                           : 1.0 L-arginine + 1.0 H2O --> 1.0 ammonium + 1.0 L-citrulline  # source: MetaCyc, BRENDA, KEGG
(queried 3.5.3.6) // other EC numbers for this reaction: 3.5.3.15
#3.5.1.87 assumed to use citrulline as substrate
arginine-degradation/2_3.5.1.87_CITRULLINASE-RXN                                : 2.0 H+ + 1.0 L-citrulline + 1.0 H2O --> 1.0 CO2 + 1.0 ammonium + 1.0 L-ornithine
#############################################################################################################################################################################
#putrescine/spermidine biosyn

putrescine-biosyn-I/1_4.1.1.19_ARGDECARBOX-RXN                                  : 1.0 H+ + 1.0 L-arginine --> 1.0 CO2 + 1.0 agmatine  # source: MetaCyc, BRENDA, KEGG (queried
4.1.1.19)
putrescine-biosyn-I/2_3.5.3.11_AGMATIN-RXN                                      : 1.0 H2O + 1.0 agmatine --> 1.0 putrescine + 1.0 urea  # source: MetaCyc, BRENDA, KEGG (queried
3.5.3.11)
spermidine-biosyn-I/1_4.1.1.50_SAMDECARB-RXN                                    : 1.0 S-adenosyl-L-methionine + 1.0 H+ --> 1.0 S-adenosyl_3-(methylthio)propylamine + 1.0 CO2  #
source: MetaCyc, BRENDA, KEGG (queried 4.1.1.50)
spermidine-biosyn-I/2_2.5.1.16_SPERMIDINESYN-RXN                                : 1.0 putrescine + 1.0 S-adenosyl_3-(methylthio)propylamine <=> 1.0 H+ + 1.0 S-methyl-5'-
thioadenosine + 1.0 spermidine  # source: MetaCyc, BRENDA, KEGG (queried 2.5.1.16)

#possible side reaction
spermine-biosynthesis_2.5.1.16_SPERMINE-SYNTHASE-RXN                            : 1.0 spermidine + 1.0 S-adenosyl_3-(methylthio)propylamine --> 1.0 H+ + 1.0 S-methyl-5'-
thioadenosine + 1.0 spermine  # source: MetaCyc, BRENDA, KEGG (queried 2.5.1.16) // other EC numbers for this reaction: 2.5.1.22

N-acetylputrescine-biosyn_2.3.1.57_RXN-0                                        : 1.0 putrescine + 1.0 acetyl-CoA --> 1.0 coenzyme_A + 1.0 N-acetylputrescine + 1.0 H+  # source:
MetaCyc, BRENDA, KEGG (queried 2.3.1.57)
N-acetylspermidine-biosyn_2.3.1.57_SPERMACTRAN-RXN                              : 1.0 acetyl-CoA + 1.0 spermidine --> 1.0 coenzyme_A + 1.0 N1-acetylspermidine + 1.0 H+  # source:
MetaCyc, BRENDA (queried 2.3.1.57)
N-acetylspermine-biosyn_2.3.1.57_RXN-9939                                       : 1.0 spermine + 1.0 acetyl-CoA --> 1.0 coenzyme_A + 1.0 N1-acetylspermine + 1.0 H+  # source:
MetaCyc (queried 2.3.1.57)
N-diacetylspermine-biosyn_2.3.1.57_BS321087                                     : 1.0 acetyl-CoA + 1.0 N1-acetylspermine --> 1.0 N1,N12-diacetylspermine + 1.0 coenzyme_A  #
source: BRENDA (queried 2.3.1.57)
#############################################################################################################################################################################
#S-methyl-5'-thioadenosine degradation
S-methyl-5'-thioadenosine-degr_3.2.2.16_3.2.2.9_METHYLTHIOADENOSINE-NUCLEOSIDASE-RXN         : 1.0 H2O + 1.0 S-methyl-5'-thioadenosine --> 1.0 adenine + 1.0 S-methyl-5-thio-D-
ribose  # source: MetaCyc, BRENDA, KEGG (queried 3.2.2.16, 3.2.2.9)
S-methyl-5'-thioadenosine-degr_3.2.2.9_5-METHYLTHIOADENOSINE-PHOSPHORYLASE-RXN               : 1.0 S-methyl-5'-thioadenosine + 1.0 phosphate --> 1.0 adenine + 1.0 S-methyl-5-thio-
alpha-D-ribose_1-phosphate  # source: MetaCyc, BRENDA, KEGG (queried 3.2.2.9) // other EC numbers for this reaction: 2.4.2.28
#############################################################################################################################################################################
#S-adenosyl-L-methionine cycle I

S-adenosyl-L-methionine-cycle-I/1_3.2.2.9_ADENOSYLHOMOCYSTEINE-NUCLEOSIDASE-RXN               : 1.0 S-adenosyl-L-homocysteine + 1.0 H2O --> 1.0 adenine + 1.0 S-ribosyl-L-
homocysteine  # source: MetaCyc, BRENDA, KEGG (queried 3.2.2.9)
S-adenosyl-L-methionine-cycle-I/2_4.4.1.21_RIBOSYLHOMOCYSTEINASE-RXN                          : 1.0 S-ribosyl-L-homocysteine --> 1.0 autoinducer_2 + 1.0 L-homocysteine  # source:
MetaCyc, BRENDA, KEGG (queried 4.4.1.21)
S-adenosyl-L-methionine-cycle-I/3_2.1.1.13_HOMOCYSMETB12-RXN                                  : 1.0 N5-methyl-tetrahydrofolate + 1.0 L-homocysteine --> 1.0 tetrahydropteroyl_tri-
L-glutamate + 1.0 L-methionine  # source: MetaCyc (queried 2.1.1.13)
```

```
S-adenosyl-L-methionine-cycle-I/4_2.5.1.6_S-ADENMETSYN-RXN                                   : 1.0 L-methionine + 1.0 ATP + 1.0 H2O --> 1.0 diphosphate + 1.0 S-adenosyl-L-
methionine + 1.0 phosphate  # source: MetaCyc, BRENDA, KEGG (queried 2.5.1.6)
#GAP: 2.1.1.; mostly DNA or t/rRNA
#auto-inducer
autoinducer-AI-2-biosyn/1_spontaneous_RXN-10015                                              : 1.0 autoinducer_2 --> 1.0 (2R,4S)-2-methyl-2,4-dihydroxydihydrofuran-3-one  #
source: MetaCyc
autoinducer-AI-2-biosyn/2_spontaneous_RXN-10017                                              : 1.0 H2O + 1.0 (2R,4S)-2-methyl-2,4-dihydroxydihydrofuran-3-one --> 1.0 (2R,4S)-2-
methyl-2,3,3,4-tetrahydroxytetrahydrofuran  # source: MetaCyc
############################################################################################################################################################################
#met biosyn C
methionine-biosyn/1_2.3.1.46_HOMSUCTRAN-RXN                                     : 1.0 succinyl-CoA + 1.0 L-homoserine --> 1.0 coenzyme_A + 1.0 O-succinyl-L-homoserine  # source:
MetaCyc, BRENDA, KEGG (queried 2.3.1.46)
methionine-biosyn/2_2.5.1.49_RXN-9384                                           : 1.0 O-succinyl-L-homoserine + 1.0 hydrogen_sulfide <=> 1.0 L-homocysteine + 1.0 H+ + 1.0
succinate  # source: MetaCyc, BRENDA, KEGG (queried 2.5.1.49) // other EC numbers for this reaction: 2.5.1.48, 2.5.1
############################################################################################################################################################################
#met degr II
methionine-degr-II_4.4.1.11_RXN-14301                                           : 1.0 L-methionine --> 1.0 H+ + 1.0 (2Z)-2-aminobut-2-enoate + 1.0 methanethiol  # source:
MetaCyc, KEGG (queried 4.4.1.11)
############################################################################################################################################################################
#2-amino-2-enoate degradation
2-aminobut-2-enoate-degr/1_spontaneous_4.4.1.1_4.3.1.19_3.5.99.7_4.4.1.2_RXN-15121                    : 1.0 (2Z)-2-aminobut-2-enoate --> 1.0 2-iminobutanoate  # source:
MetaCyc, BRENDA, KEGG (queried 4.4.1.1, 4.3.1.19, 3.5.99.7, 4.4.1.2)
2-aminobut-2-enoate-degr/2_spontaneous_3.5.99.7_3.5.99.10_4.4.1.1_4.3.1.19_4.4.1.2_3.5.99.-_RXN-15123         : 1.0 H2O + 1.0 H+ + 1.0 2-iminobutanoate --> 1.0 2-oxobutanoate +
1.0 ammonium  # source: MetaCyc, BRENDA, KEGG (queried 3.5.99.7, 3.5.99.10, 4.4.1.1, 4.3.1.19, 4.4.1.2, 3.5.99.-) // other EC numbers for this reaction: 3.5.99

2-aminoprop-2-enoate-degr/1_spontaneous_4.4.1.8_4.4.1.13_4.4.1.25_4.3.1.17_4.4.1.28_RXN-15124                 : 1.0 2-aminoprop-2-enoate --> 1.0 2-iminopropanoate  # source:
MetaCyc, BRENDA, KEGG (queried 4.4.1.8, 4.4.1.13, 4.4.1.25, 4.3.1.17, 4.4.1.28)
2-aminoprop-2-enoate-degr/2_spontaneous_4.4.1.8_4.4.1.13_4.3.1.17_4.4.1.28_3.5.99.10_4.4.1.25_RXN-15127       : 1.0 H2O + 1.0 2-iminopropanoate + 1.0 H+ --> 1.0 ammonium + 1.0
pyruvate  # source: MetaCyc, BRENDA, KEGG (queried 4.4.1.8, 4.4.1.13, 4.3.1.17, 4.4.1.28, 3.5.99.10, 4.4.1.25)
############################################################################################################################################################################
#2-aminobutanoate
2-aminobutanoate_2.6.1.42_BS356546                                                 : 1.0 (S)-2-aminobutanoate + 1.0 2-oxoglutarate <-- 1.0 2-oxobutanoate + 1.0 L-glutamate  #
source: BRENDA, KEGG (queried 2.6.1.42)
############################################################################################################################################################################
#threonine biosyn
threonine-from-homoserine/0_1.1.1.3_HOMOSERDEHYDROG-RXN                            : 1.0 NADP+ + 1.0 L-homoserine <-- 1.0 H+ + 1.0 L-aspartate-semialdehyde + 1.0 NADPH  # source:
MetaCyc, BRENDA, KEGG (queried 1.1.1.3)
threonine-from-homoserine/1_2.7.1.39_HOMOSERKIN-RXN                                : 1.0 L-homoserine + 1.0 ATP --> 1.0 H+ + 1.0 ADP + 1.0 O-phospho-L-homoserine  # source:
MetaCyc, BRENDA, KEGG (queried 2.7.1.39)
threonine-from-homoserine/2_4.2.3.1_THRESYN-RXN                                    : 1.0 H2O + 1.0 O-phospho-L-homoserine --> 1.0 phosphate + 1.0 L-threonine  # source: MetaCyc,
BRENDA, KEGG (queried 4.2.3.1)
############################################################################################################################################################################
#threonine degr
threonine-degr-IV_4.1.2.5_THREONINE-ALDOLASE-RXN                                   : 1.0 L-threonine --> 1.0 glycine + 1.0 acetaldehyde  # source: MetaCyc, BRENDA, KEGG (queried
4.1.2.5) // other EC numbers for this reaction: 4.1.2.48

threonine-degr-V_4.3.1.19_RXN-15122                                                : 1.0 L-threonine --> 1.0 (2Z)-2-aminobut-2-enoate + 1.0 H+ + 1.0 H2O  # source: MetaCyc,
BRENDA (queried 4.3.1.19)

threonine-degr-II/1_1.1.1.103_THREODEHYD-RXN                                       : 1.0 L-threonine + 1.0 NAD+ --> 1.0 L-2-amino-3-oxobutanoate + 1.0 NADH + 1.0 H+  # source:
MetaCyc, BRENDA, KEGG (queried 1.1.1.103)
threonine-degr-II/2_spontaneous_THREOSPON-RXN                                      : 1.0 L-2-amino-3-oxobutanoate + 1.0 H+ --> 1.0 aminoacetone + 1.0 CO2
############################################################################################################################################################################
#homoserine degr
#C. sporogenes (Kreis and Hession, 1973) enzyme homolog
homoserine-degr_4.4.1.1_4.3.1.19_4.4.1.11_HOMOSERDEAM-RXN                          : 1.0 L-homoserine --> 1.0 2-oxobutanoate + 1.0 ammonium  # source: MetaCyc, BRENDA (queried
4.4.1.1, 4.3.1.19, 4.4.1.11)
############################################################################################################################################################################
#serine biosyn
serine-biosyn_2.1.2.1_GLYOHMETRANS-RXN                                             : 1.0 tetrahydropteroyl_tri-L-glutamate + 1.0 L-serine <=> 1.0 glycine + 1.0 H2O + 1.0 5,10-
methylene-tetrahydrofolate  # source: MetaCyc (queried 2.1.2.1)
############################################################################################################################################################################
#serine degr
serine-degr-A_4.3.1.17_RXN-15125                                                   : 1.0 L-serine --> 1.0 H2O + 1.0 2-aminoprop-2-enoate + 1.0 H+  # source: MetaCyc, BRENDA, KEGG
```

```
(queried 4.3.1.17)

serine-degr-B/1a_2.6.1.51_SERINE--PYRUVATE-AMINOTRANSFERASE-RXN                     : 1.0 L-serine + 1.0 pyruvate <=> 1.0 hydroxypyruvate + 1.0 L-alanine  # source: MetaCyc,
BRENDA, KEGG (queried 2.6.1.51)
serine-degr-B/1b_2.6.1.2_RXN-974                                                    : 1.0 L-serine + 1.0 2-oxoglutarate <=> 1.0 L-glutamate + 1.0 hydroxypyruvate  # source:
MetaCyc, BRENDA (queried 2.6.1.2)
serine-degr-B/2a_1.1.1.81_1.1.1.60_GLYCERATE-DEHYDROGENASE-RXN                      : 1.0 NAD+ + 1.0 D-glycerate <-- 1.0 NADH + 1.0 hydroxypyruvate + 1.0 H+  # source: MetaCyc,
BRENDA, KEGG (queried 1.1.1.81, 1.1.1.60) // other EC numbers for this reaction: 1.1.1.29, 1.1.1.26, 1.1.1.79
serine-degr-B/2b_1.1.1.81_RXN0-300                                                  : 1.0 D-glycerate + 1.0 NADP+ <-- 1.0 hydroxypyruvate + 1.0 NADPH + 1.0 H+  # source: MetaCyc,
BRENDA, KEGG (queried 1.1.1.81) // other EC numbers for this reaction: 1.1.1, 1.1.1.215, 1.1.1.79
serine-degr-B/3_2.7.1.31_GLY3KIN-RXN                                                : 1.0 ATP + 1.0 D-glycerate --> 1.0 ADP + 1.0 3-phospho-D-glycerate + 1.0 H+  # source:
MetaCyc, BRENDA, KEGG (queried 2.7.1.31)
############################################################################################################################################################################
#sec biosyn I
selenocysteine-biosyn-I/1_2.7.9.3_2.7.9.3-RXN                                       : 1.0 H2O + 1.0 selenide + 1.0 ATP --> 1.0 selenophosphate + 1.0 phosphate + 1.0 AMP  # source:
MetaCyc, BRENDA, KEGG (queried 2.7.9.3)
selenocysteine-biosyn-I/2_2.9.1.1_2.9.1.1-RXN                                       : 1.0 selenophosphate + 1.0 |L-seryl-SEC-tRNAs| --> 1.0 phosphate + 1.0 |Charged-SEC-tRNAs|  #
source: MetaCyc (queried 2.9.1.1)

seleno-amino-acid-biosynthesis_2.5.1.47_RXN-12726                                   : 1.0 O-acetyl-L-serine + 1.0 hydrogen_selenide --> 1.0 acetate + 1.0 L-selenocysteine + 1.0 H+
# source: MetaCyc, BRENDA, KEGG (queried 2.5.1.47)
############################################################################################################################################################################
#sec degr C
#NADH as a guess
selenocysteine-degr_mod_4.4.1.16_mod_SELENOCYSTEINE-LYASE-RXN                       : 1.0 NADPH + 1.0 L-selenocysteine --> 1.0 selenide + 1.0 NADP+ + 1.0 L-alanine + 1.0 H+ #
source: MetaCyc, BRENDA, KEGG (queried 4.4.1.16)
############################################################################################################################################################################
#cys biosyn I
cysteine-biosyn-I/1_2.5.1.47_ACSERLY-RXN                                            : 1.0 hydrogen_sulfide + 1.0 O-acetyl-L-serine <=> 1.0 acetate + 1.0 L-cysteine + 1.0 H+  #
source: MetaCyc, BRENDA, KEGG (queried 2.5.1.47) // other EC numbers for this reaction: 2.5.1.65
cysteine-biosyn-I/2_2.3.1.30_SERINE-O-ACETTRAN-RXN                                  : 1.0 L-serine + 1.0 acetyl-CoA --> 1.0 O-acetyl-L-serine + 1.0 coenzyme_A  # source: MetaCyc,
BRENDA, KEGG (queried 2.3.1.30)
############################################################################################################################################################################
#cys degr II
cysteine-degr-II_4.4.1.8_4.4.1.1_LCYSDESULF-RXN                                     : 1.0 H2O + 1.0 L-cysteine --> 1.0 pyruvate + 1.0 hydrogen_sulfide + 1.0 ammonium  # source:
MetaCyc, BRENDA, KEGG (queried 4.4.1.8, 4.4.1.1) // other EC numbers for this reaction: 4.4.1.28
############################################################################################################################################################################
#sulfite degr
sulfite-degr_manual_1.8.1.-_HD-sulf                                                 : 1.0 sulfite + 3.0 NADH + 5.0 H+ <=> 3.0 H2O + 1.0 hydrogen_sulfide + 3.0 NAD+
############################################################################################################################################################################
#histidine biosyn
histidine-biosyn/1_2.4.2.17_ATPPHOSPHORIBOSYLTRANS-RXN                              : 1.0 diphosphate + 1.0 1-(5-phospho-beta-D-ribosyl)-ATP <=> 1.0 H+ + 1.0 ATP + 1.0 5-phospho-
alpha-D-ribose_1-diphosphate  # source: MetaCyc, BRENDA, KEGG (queried 2.4.2.17)
histidine-biosyn/2_3.6.1.31_HISTPRATPHYD-RXN                                        : 1.0 H2O + 1.0 1-(5-phospho-beta-D-ribosyl)-ATP --> 1.0 1-(5-phospho-beta-D-ribosyl)-AMP + 2.0
H+ + 1.0 diphosphate  # source: MetaCyc, BRENDA, KEGG (queried 3.6.1.31)
histidine-biosyn/3_3.5.4.19_HISTCYCLOHYD-RXN                                        : 1.0 1-(5-phospho-beta-D-ribosyl)-AMP + 1.0 H2O --> 1.0 1-(5-phospho-beta-D-ribosyl)-5-[(5-
phosphoribosylamino)methylideneamino]imidazole-4-carboxamide  # source: MetaCyc, BRENDA, KEGG (queried 3.5.4.19)
histidine-biosyn/4_5.3.1.16_PRIBFAICARPISOM-RXN                                     : 1.0 1-(5-phospho-beta-D-ribosyl)-5-[(5-phosphoribosylamino)methylideneamino]imidazole-4-
carboxamide --> 1.0 phosphoribulosylformimino-AICAR-P  # source: MetaCyc, BRENDA, KEGG (queried 5.3.1.16)
histidine-biosyn/5_4.1.3.-_2.4.2.-_GLUTAMIDOTRANS-RXN                               : 1.0 phosphoribulosylformimino-AICAR-P + 1.0 L-glutamine --> 1.0 D-erythro-imidazole-glycerol-
phosphate + 1.0 H+ + 1.0 5-amino-1-(5-phospho-D-ribosyl)imidazole-4-carboxamide + 1.0 L-glutamate  # source: MetaCyc, BRENDA, KEGG (queried 4.1.3.-, 2.4.2.-) // other EC numbers
for this reaction: 2.4.2, 4.3.1.B2, 4.1.3
histidine-biosyn/6_4.2.1.19_IMIDPHOSDEHYD-RXN                                       : 1.0 D-erythro-imidazole-glycerol-phosphate --> 1.0 imidazole_acetol-phosphate + 1.0 H2O  #
source: MetaCyc, BRENDA, KEGG (queried 4.2.1.19)
histidine-biosyn/7_2.6.1.9_HISTAMINOTRANS-RXN                                       : 1.0 L-glutamate + 1.0 imidazole_acetol-phosphate --> 1.0 2-oxoglutarate + 1.0 L-histidinol-
phosphate  # source: MetaCyc, BRENDA, KEGG (queried 2.6.1.9)
histidine-biosyn/8_3.1.3.15_HISTIDPHOS-RXN                                          : 1.0 H2O + 1.0 L-histidinol-phosphate --> 1.0 histidinol + 1.0 phosphate  # source: MetaCyc,
BRENDA, KEGG (queried 3.1.3.15)
histidine-biosyn/9_1.1.1.23_HISTOLDEHYD-RXN                                         : 1.0 histidinol + 1.0 NAD+ --> 1.0 NADH + 1.0 histidinal + 1.0 H+  # source: MetaCyc, BRENDA,
KEGG (queried 1.1.1.23)
histidine-biosyn/10_1.1.1.23_HISTALDEHYD-RXN                                        : 1.0 histidinal + 1.0 H2O + 1.0 NAD+ --> 2.0 H+ + 1.0 NADH + 1.0 L-histidine  # source:
MetaCyc, BRENDA, KEGG (queried 1.1.1.23)
############################################################################################################################################################################
#alanine biosyn
```

alanine-biosyn_4.1.1.12_ASPARTATE-4-DECARBOXYLASE-RXN : 1.0 H+ + 1.0 L-aspartate --> 1.0 CO2 + 1.0 L-alanine # source: MetaCyc, BRENDA, KEGG (queried 4.1.1.12)
alanine-biosyn_2.6.1.2_ALANINE-AMINOTRANSFERASE-RXN : 1.0 L-alanine + 1.0 2-oxoglutarate <=> 1.0 pyruvate + 1.0 L-glutamate # source: MetaCyc, BRENDA, KEGG (queried 2.6.1.2)
##
#ornithine degr. II
ornithine-degr-II/1_5.1.1.12_ORNITHINE-RACEMASE-RXN : 1.0 L-ornithine <=> 1.0 D-ornithine # source: MetaCyc, BRENDA, KEGG (queried 5.1.1.12) // other EC numbers for this reaction: 5.1.1.9, 5.1.1.10
ornithine-degr-II/2_5.4.3.5_ORNMUTST-RXN : 1.0 D-ornithine <=> 1.0 (2R,4S)-2,_4-diaminopentanoate # source: MetaCyc, BRENDA, KEGG (queried 5.4.3.5)
ornithine-degr-II/3_1.4.1.12_24-DIAMINOPENTANOATE-DEHYDROGENASE-RXN_WOP : 1.0 (2R,4S)-2,_4-diaminopentanoate + 1.0 H2O + 1.0 NAD+ <=> 1.0 ammonium + 1.0 NADH + 1.0 H+ + 1.0 2-amino-4-oxopentanoate # source: MetaCyc, BRENDA, KEGG (queried 1.4.1.12)
ornithine-degr-II/4_2.3.1.B10_AKPTHIOL-RXN : 1.0 2-amino-4-oxopentanoate + 1.0 coenzyme_A <=> 1.0 acetyl-CoA + 1.0 D-alanine # source: MetaCyc, BRENDA, KEGG (queried 2.3.1.B10) // other EC numbers for this reaction: 2.3.1
##
#pro degr
proline-degr/1_5.1.1.4_PROLINE-RACEMASE-RXN : 1.0 L-proline <=> 1.0 D-proline # source: MetaCyc, BRENDA, KEGG (queried 5.1.1.4)
proline-degr/2_1.21.4.1_BS370299 : 1.0 H+ + 1.0 D-proline + 1.0 NADH <=> 1.0 5-aminopentanoate + 1.0 NAD+ # source: BRENDA (queried 1.21.4.1) // balanced // contains implicit reactant(s): H+, H2O
##
#glycine degr.
glycine-cleavage/1_moddir_1.4.4.2_GCVP-RXN : 1.0 glycine + 1.0 lipoamide + 1.0 H+ <=> 1.0 [glycine-cleavage_complex_H_protein]_N6-aminomethyldihydrolipoyl-L-lysine + 1.0 CO2 # source: MetaCyc (queried 1.4.4.2)
glycine-cleavage/2_2.1.2.10_GCVT-RXN : 1.0 tetrahydropteroyl_tri-L-glutamate + 1.0 [glycine-cleavage_complex_H_protein]_N6-aminomethyldihydrolipoyl-L-lysine <=> 1.0 dihydrolipoamide + 1.0 5,10-methylene-tetrahydrofolate + 1.0 ammonium # source: MetaCyc (queried 2.1.2.10)
glycine-cleavage/3_1.8.1.4_RXN-7719 : 1.0 NAD+ + 1.0 dihydrolipoamide <=> 1.0 lipoamide + 1.0 NADH + 1.0 H+ # source: MetaCyc (queried 1.8.1.4)

glycine-degr/1_1.21.4.2_RXN-7566 : 1.0 |Ox-Thioredoxin| + 1.0 H2O + 1.0 ammonium + 1.0 acetyl_phosphate <-- 1.0 |Red-Thioredoxin| + 1.0 H+ + 1.0 phosphate + 1.0 glycine # source: MetaCyc, KEGG (queried 1.21.4.2)
glycine-degr/2_1.8.1.9_THIOREDOXIN-REDUCT-NADPH-RXN : 1.0 NADP+ + 1.0 |Red-Thioredoxin| <-- 1.0 NADPH + 1.0 H+ + 1.0 |Ox-Thioredoxin| # source: MetaCyc, KEGG (queried 1.8.1.9)
##
#chorismate biosyn I
chorismate-biosyn-I/1_2.5.1.54_DAHPSYN-RXN : 1.0 H2O + 1.0 phosphoenolpyruvate + 1.0 D-erythrose_4-phosphate <=> 1.0 phosphate + 1.0 3-deoxy-D-arabino-heptulosonate-7-phosphate # source: MetaCyc, BRENDA, KEGG (queried 2.5.1.54)
chorismate-biosyn-I/2_4.2.3.4_3-DEHYDROQUINATE-SYNTHASE-RXN : 1.0 3-deoxy-D-arabino-heptulosonate-7-phosphate --> 1.0 phosphate + 1.0 3-dehydroquinate # source: MetaCyc, BRENDA, KEGG (queried 4.2.3.4)
chorismate-biosyn-I/3_4.2.1.10_3-DEHYDROQUINATE-DEHYDRATASE-RXN : 1.0 3-dehydroquinate <=> 1.0 H2O + 1.0 3-dehydroshikimate # source: MetaCyc, BRENDA, KEGG (queried 4.2.1.10)
chorismate-biosyn-I/4_1.1.1.25_RXN-7968 : 1.0 shikimate + 1.0 NADP+ <-- 1.0 3-dehydroshikimate + 1.0 H+ + 1.0 NADPH # source: MetaCyc, BRENDA, KEGG (queried 1.1.1.25) // other EC numbers for this reaction: 1.1.1.282, 1.1.1.24
chorismate-biosyn-I/5_2.7.1.71_SHIKIMATE-KINASE-RXN : 1.0 ATP + 1.0 shikimate --> 1.0 H+ + 1.0 ADP + 1.0 shikimate_3-phosphate # source: MetaCyc, BRENDA, KEGG (queried 2.7.1.71)
chorismate-biosyn-I/6_2.5.1.19_2.5.1.19-RXN : 1.0 phosphoenolpyruvate + 1.0 shikimate_3-phosphate <=> 1.0 5-enolpyruvyl-shikimate_3-phosphate + 1.0 phosphate # source: MetaCyc, BRENDA, KEGG (queried 2.5.1.19)
chorismate-biosyn-I/7_4.2.3.5_CHORISMATE-SYNTHASE-RXN : 1.0 5-enolpyruvyl-shikimate_3-phosphate --> 1.0 phosphate + 1.0 chorismate # source: MetaCyc, BRENDA, KEGG (queried 4.2.3.5)
##
#tyr/phe biosyn

tyr/phe-biosyn_5.4.99.5_CHORISMATEMUT-RXN : 1.0 chorismate <=> 1.0 prephenate # source: MetaCyc, BRENDA, KEGG (queried 5.4.99.5)

tyrosine-biosyn_I/1_1.3.1.12_PREPHENATEDEHYDROG-RXN : 1.0 prephenate + 1.0 NAD+ --> 1.0 4-hydroxyphenylpyruvate + 1.0 CO2 + 1.0 NADH # source: MetaCyc, BRENDA, KEGG (queried 1.3.1.12)
tyrosine-biosyn_I/2a_2.6.1.9_2.6.1.1_2.6.1.57_TYROSINE-AMINOTRANSFERASE-RXN : 1.0 L-tyrosine + 1.0 2-oxoglutarate <=> 1.0 4-hydroxyphenylpyruvate + 1.0 L-glutamate # source: MetaCyc, BRENDA, KEGG (queried 2.6.1.9, 2.6.1.1, 2.6.1.57) // other EC numbers for this reaction: 2.6.1.5
tyrosine-biosyn_I/2b_2.6.1.51_RXN3O-4157 : 1.0 L-tyrosine + 1.0 pyruvate <=> 1.0 L-alanine + 1.0 4-hydroxyphenylpyruvate # source: MetaCyc, BRENDA, KEGG (queried 2.6.1.51) // other EC numbers for this reaction: 2.6.1.44, 2.6.1.58

phenylalanine-biosyn-I/1_4.2.1.51_PREPHENATEDEHYDRAT-RXN : 1.0 prephenate + 1.0 H+ --> 1.0 2-oxo-3-phenylpropanoate + 1.0 CO2 + 1.0 H2O # source: MetaCyc, BRENDA, KEGG (queried 4.2.1.51) // other EC numbers for this reaction: 4.2.1.91
phenylalanine-biosyn-I/2a_2.6.1.57_2.6.1.9_2.6.1.1_2.6.1.42_PHEAMINOTRANS-RXN : 1.0 L-glutamate + 1.0 2-oxo-3-phenylpropanoate <=> 1.0 2-oxoglutarate + 1.0 L-phenylalanine # source: MetaCyc, BRENDA, KEGG (queried 2.6.1.57, 2.6.1.9, 2.6.1.1, 2.6.1.42) // there are 5 more EC numbers for this reaction

```
phenylalanine-biosyn-I/2b_2.6.1.57_RXN-7708                                       : 1.0 2-oxo-3-phenylpropanoate + 1.0 L-methionine <=> 1.0 2-oxo-4-methylthiobutanoate + 1.0 L-
phenylalanine  # source: MetaCyc, BRENDA (queried 2.6.1.57) // other EC numbers for this reaction: 2.6.1.28

#side reactions
phenylalanine-biosyn-II/1_2.6.1.57_PREPHENATE-ASP-TRANSAMINE-RXN                  : 1.0 oxaloacetate + 1.0 L-arogenate <=> 1.0 L-aspartate + 1.0 prephenate  # source: MetaCyc,
BRENDA, KEGG (queried 2.6.1.57) // other EC numbers for this reaction: 2.6.1.78
phenylalanine-biosyn-II/2_4.2.1.51_CARBOXYCYCLOHEXADIENYL-DEHYDRATASE-RXN         : 1.0 H+ + 1.0 L-arogenate --> 1.0 L-phenylalanine + 1.0 CO2 + 1.0 H2O  # source: MetaCyc,
BRENDA, KEGG (queried 4.2.1.51) // other EC numbers for this reaction: 4.2.1.91

methionine-from-2-oxo-4-methylthiobutanoate_2.6.1.57_RXN-14147                    : 1.0 2-oxoglutarate + 1.0 L-methionine <=> 1.0 2-oxo-4-methylthiobutanoate + 1.0 L-glutamate
# source: MetaCyc, BRENDA, KEGG (queried 2.6.1.57) // other EC numbers for this reaction: 2.6.1.5, 2.6.1.7
########################################################################################################################################################################
#trp/tyr/phe/his degr

tryptophan-degr_2.6.1.27_TRYPTOPHAN-AMINOTRANSFERASE-RXN                          : 1.0 L-tryptophan + 1.0 2-oxoglutarate <=> 1.0 L-glutamate + 1.0 indole-3-pyruvate  # source:
MetaCyc, BRENDA, KEGG (queried 2.6.1.27)
tryptophan-degr_2.6.1.57_RXN-14685                                                : 1.0 indole-3-pyruvate + 1.0 L-methionine <=> 1.0 2-oxo-4-methylthiobutanoate + 1.0 L-
tryptophan  # source: MetaCyc, BRENDA (queried 2.6.1.57)
histidine-degr_2.6.1.38_HISTTRANSAM-RXN                                           : 1.0 L-histidine + 1.0 2-oxoglutarate <=> 1.0 imidazole-pyruvate + 1.0 L-glutamate  # source:
MetaCyc, BRENDA, KEGG (queried 2.6.1.38)

tryptophan-ox/1_1.2.7.8_1.2.7.8-RXN                                               : 1.0 coenzyme_A + 2.0 |Oxidized-ferredoxins| + 1.0 indole-3-pyruvate --> 2.0 |Reduced-
ferredoxins| + 1.0 S-2-(indol-3_yl)acetyl-CoA + 1.0 H+ + 1.0 CO2  # source: MetaCyc, KEGG (queried 1.2.7.8)
tyrosine-ox/1_manual_1.2.7.8_HD-1.2.7.8-tyr                                       : 1.0 4-hydroxyphenylpyruvate + 2.0 |Oxidized-ferredoxins| + 1.0 coenzyme_A --> 1.0 CO2 + 2.0 |
Reduced-ferredoxins| + 1.0 4-hydroxyphenylacetyl-CoA  + 1.0 H+
phenylalanine-ox/1_manual_1.2.7.8_HD-1.2.7.8-phe                                  : 1.0 2-oxo-3-phenylpropanoate + 2.0 |Oxidized-ferredoxins| + 1.0 coenzyme_A --> 1.0 CO2 + 2.0
|Reduced-ferredoxins| + 1.0 phenylacetyl-CoA
histidine-ox/1_manual_1.2.7.8_HD-1.2.7.8-his                                      : 1.0 imidazole-pyruvate + 2.0 |Oxidized-ferredoxins| + 1.0 coenzyme_A --> 1.0 CO2 + 2.0 |
Reduced-ferredoxins| + 1.0 imidazolylacetyl-CoA

tryptophan-ox/2_manual_2.3.1.-_HD-2.3.1.-trp                                      : 1.0 S-2-(indol-3_yl)acetyl-CoA + 1.0 phosphate --> 1.0 indole-3-acetyl_phosphate + 1.0
coenzyme_A
tyrosine-ox/2_2.3.1.-_manual_HD-2.3.1.-tyr                                        : 1.0 4-hydroxyphenylacetyl-CoA + 1.0 phosphate --> 1.0 4-hydroxyphenylacetyl_phosphate + 1.0
coenzyme_A
phenylalanine-ox/2_2.3.1.-_manual_HD-2.3.1.-phe                                   : 1.0 phenylacetyl-CoA + 1.0 phosphate --> 1.0 phenylacetyl_phosphate + 1.0 coenzyme_A
histidine-ox/2_2.3.1.-_manual_HD-2.3.1.-his                                       : 1.0 imidazolylacetyl-CoA + 1.0 phosphate --> 1.0 imidazolylacetyl_phosphate + 1.0 coenzyme_A

tryptophan-ox/3_manual_2.7.2.-_HD-2.7.2.-trp                                      : 1.0 indole-3-acetyl_phosphate + 1.0 ADP --> 1.0 indole-3-acetate + 1.0 ATP
tyrosine-ox/3_manual_2.7.2.-_HD-2.7.2.-tyr                                        : 1.0 4-hydroxyphenylacetyl_phosphate + 1.0 ADP --> 1.0 4-hydroxyphenylacetate + 1.0 ATP
phenylalanine-ox/3_manual_2.7.2.-_HD-2.7.2.-phe                                   : 1.0 phenylacetyl_phosphate + 1.0 ADP --> 1.0 phenylacetate + 1.0 ATP
histidine-ox/3_manual_2.7.2.-_HD-2.7.2.-his                                       : 1.0 imidazolylacetyl_phosphate + 1.0 ADP --> 1.0 4-imidazoleacetate + 1.0 ATP

tyrosine-ox/4_4.1.1.83_4.1.1.83-RXN                                               : 1.0 H+ + 1.0 4-hydroxyphenylacetate --> 1.0 CO2 + 1.0 4-methylphenol  # source: MetaCyc,
BRENDA, KEGG (queried 4.1.1.83)
########################################################################################################################################################################
#arom red
phe-red/1_1.1.1.222_BS186998                                                      : 1.0 (R)-phenyllactate + 1.0 NAD+ <-- 1.0 H+ + 1.0 NADH + 1.0 2-oxo-3-phenylpropanoate  #
source: BRENDA, KEGG (queried 1.1.1.222) // other EC numbers for this reaction: 1.1.1.110, 1.1.1.237 // balanced // contains implicit reactant(s): H+, H2O
phe-red/2_manual_2.8.3.-_HD-2.8.3.-phe                                            : 1.0 (R)-phenyllactoyl-CoA + 1.0 3-phenylpropanoate <=> 1.0 3-phenylpropanoyl-CoA + 1.0 (R)-
phenyllactate
phe-red/3_manual_4.2.1.-_HD-4.2.1.-phe                                            : 1.0 (R)-phenyllactoyl-CoA --> 1.0 3-phenylacryloyl-CoA + 1.0 H2O
phe-red/4_manual_1.3.98.-_HD-1.3.98.-phe                                          : 1.0 3-phenylacryloyl-CoA + 2.0 NADH + 2.0 |Oxidized-ferredoxins| <=> 1.0 3-phenylpropanoyl-
CoA + 2.0 |Reduced-ferredoxins| + 2.0 NAD+

tyr-red/1_1.1.1.222_1.1.1.222-RXN_WOP                                             : 1.0 NADH + 1.0 4-hydroxyphenylpyruvate + 1.0 H+ <=> 1.0 (R)-3-(4-hydroxyphenyl)lactate + 1.0
NAD+  # source: MetaCyc, BRENDA, KEGG (queried 1.1.1.222)
tyr-red/2_manual_2.8.3.-_HD-2.8.3.-tyr                                            : 1.0 (R)-(4-hydroxyphenyl)lactoyl-CoA + 1.0 3-(4-hydroxyphenyl)propanoate <=> 1.0 3-(4-
hydroxyphenyl)propanoyl-CoA + 1.0 (R)-3-(4-hydroxyphenyl)lactate
tyr-red/3_manual_4.2.1.-_HD-4.2.1.-tyr                                            : 1.0 (R)-(4-hydroxyphenyl)lactoyl-CoA --> 1.0 3-(4-hydroxyphenyl)acryloyl-CoA + 1.0 H2O
tyr-red/4_manual_1.3.98.-_HD-1.3.98.-tyr                                          : 1.0 3-(4-hydroxyphenyl)acryloyl-CoA + 2.0 NADH + 2.0 |Oxidized-ferredoxins| <=> 1.0 3-(4-
hydroxyphenyl)propanoyl-CoA + 2.0 |Reduced-ferredoxins| + 2.0 NAD+
########################################################################################################################################################################
#valine biosyn
valine-biosyn/1_2.2.1.6_RXN-12583                                                 : 1.0 thiamine_diphosphate + 1.0 pyruvate + 1.0 H+ --> 1.0 2-(alpha-
```

```
hydroxyethyl)thiamine_diphosphate + 1.0 CO2  # source: MetaCyc, KEGG (queried 2.2.1.6) // other EC numbers for this reaction: 4.1.1.1, 1.2.4.1
valine-biosyn/2_2.2.1.6_RXN-14037                                         : 1.0 (S)-2-acetolactate + 1.0 thiamine_diphosphate <=> 1.0 2-(alpha-
hydroxyethyl)thiamine_diphosphate + 1.0 pyruvate  # source: MetaCyc, KEGG (queried 2.2.1.6)
valine-biosyn/3_1.1.1.86_RXN-16061                                        : 1.0 NADP+ + 1.0 (2R)-2,3-dihydroxy-3-methylbutanoate <=> 1.0 H+ + 1.0 (S)-2-acetolactate +
1.0 NADPH  # source: MetaCyc, BRENDA, KEGG (queried 1.1.1.86) // other EC numbers for this reaction: 1.1.1.383
valine-biosyn/4_4.2.1.9_DIHYDROXYISOVALDEHYDRAT-RXN                       : 1.0 (2R)-2,3-dihydroxy-3-methylbutanoate --> 1.0 H2O + 1.0 3-methyl-2-oxobutanoate  # source:
MetaCyc, KEGG (queried 4.2.1.9)

valine-biosyn/5_2.6.1.42_BRANCHED-CHAINAMINOTRANSFERVAL-RXN               : 1.0 2-oxoglutarate + 1.0 L-valine <=> 1.0 3-methyl-2-oxobutanoate + 1.0 L-glutamate  #
source: MetaCyc, BRENDA, KEGG (queried 2.6.1.42) // other EC numbers for this reaction: 2.6.1.6
#####################################################################################################################################################################################
#leucine biosyn
leucine-biosyn/1_2.3.3.13_2-ISOPROPYLMALATESYN-RXN                        : 1.0 H2O + 1.0 acetyl-CoA + 1.0 3-methyl-2-oxobutanoate --> 1.0 H+ + 1.0 coenzyme_A + 1.0
(2S)-2-isopropylmalate  # source: MetaCyc, BRENDA, KEGG (queried 2.3.3.13)
leucine-biosyn/2_4.2.1.33_RXN-13163                                       : 1.0 (2R,3S)-3-isopropylmalate <=> 1.0 (2S)-2-isopropylmalate  # source: MetaCyc, BRENDA, KEGG
(queried 4.2.1.33)
leucine-biosyn/3_1.1.1.85_3-ISOPROPYLMALDEHYDROG-RXN                      : 1.0 NAD+ + 1.0 (2R,3S)-3-isopropylmalate <=> 1.0 H+ + 1.0 NADH + 1.0 (2S)-2-isopropyl-3-
oxosuccinate  # source: MetaCyc, BRENDA, KEGG (queried 1.1.1.85)
leucine-biosyn/4_spontaneous_1.1.1.85_RXN-7800                            : 1.0 (2S)-2-isopropyl-3-oxosuccinate + 1.0 H+ --> 1.0 4-methyl-2-oxopentanoate + 1.0 CO2  #
source: MetaCyc, KEGG (queried 1.1.1.85)

leucine-biosyn/5_2.6.1.42_BRANCHED-CHAINAMINOTRANSFERLEU-RXN              : 1.0 L-leucine + 1.0 2-oxoglutarate <=> 1.0 4-methyl-2-oxopentanoate + 1.0 L-glutamate  #
source: MetaCyc, BRENDA, KEGG (queried 2.6.1.42) // other EC numbers for this reaction: 2.6.1.67, 2.6.1.6
#####################################################################################################################################################################################
#isoleucine biosyn

isoleucine-biosyn-II/1_2.3.1.182_RXN-7743                                 : 1.0 pyruvate + 1.0 acetyl-CoA + 1.0 H2O --> 1.0 H+ + 1.0 (R)-citramalate + 1.0 coenzyme_A  #
source: MetaCyc, BRENDA, KEGG (queried 2.3.1.182)
isoleucine-biosyn-II/2_4.2.1.35_R-2-METHYLMALATE-DEHYDRATASE-RXN          : 1.0 (R)-citramalate <=> 1.0 H2O + 1.0 citraconate  # source: MetaCyc, BRENDA, KEGG (queried
4.2.1.35)
isoleucine-biosyn-II/3_4.2.1.35_RXN-7744                                  : 1.0 citraconate + 1.0 H2O --> 1.0 (2R,3S)-3-methylmalate  # source: MetaCyc, KEGG (queried
4.2.1.35)
isoleucine-biosyn-II/4_1.1.1.85_RXN-7745                                  : 1.0 NAD+ + 1.0 (2R,3S)-3-methylmalate --> 1.0 CO2 + 1.0 2-oxobutanoate + 1.0 NADH  # source:
MetaCyc, KEGG (queried 1.1.1.85) // other EC numbers for this reaction: 1.1.1
isoleucine-biosyn-II/5_2.2.1.6_R04673                                     : 1.0 2-oxobutanoate + 1.0 2-(alpha-hydroxyethyl)thiamine_diphosphate <=> 1.0
thiamine_diphosphate + 1.0 (S)-2-aceto-2-hydroxybutanoate  # source: KEGG (queried 2.2.1.6)
isoleucine-biosyn-II/6_1.1.1.86_ACETOOHBUTREDUCTOISOM-RXN                  : 1.0 (R)-2,3-dihydroxy-3-methylpentanoate + 1.0 NADP+ <-- 1.0 NADPH + 1.0 (S)-2-aceto-2-
hydroxybutanoate + 1.0 H+  # source: MetaCyc (queried 1.1.1.86)
isoleucine-biosyn-II/7_4.2.1.9_DIHYDROXYMETVALDEHYDRAT-RXN                : 1.0 (R)-2,3-dihydroxy-3-methylpentanoate --> 1.0 H2O + 1.0 (S)-3-methyl-2-oxopentanoate  #
source: MetaCyc, KEGG (queried 4.2.1.9)
isoleucine-biosyn-II/8_2.6.1.42_BRANCHED-CHAINAMINOTRANSFERILEU-RXN       : 1.0 L-isoleucine + 1.0 2-oxoglutarate <=> 1.0 (S)-3-methyl-2-oxopentanoate + 1.0 L-glutamate
# source: MetaCyc, BRENDA, KEGG (queried 2.6.1.42) // other EC numbers for this reaction: 2.6.1.67
#####################################################################################################################################################################################
#val/leu/ile degr ox

valine-ox/1_1.2.7.7_1.2.7.7-RXN                                           : 1.0 coenzyme_A + 1.0 3-methyl-2-oxobutanoate + 2.0 |Oxidized-ferredoxins| --> 1.0 CO2 + 2.0 |
Reduced-ferredoxins| + 1.0 H+ + 1.0 isobutanoyl-CoA  # source: MetaCyc, KEGG (queried 1.2.7.7)
leucine-ox/1_manual_1.2.7.7_HD-1.2.7.7-leu                                : 1.0 4-methyl-2-oxopentanoate + 2.0 |Oxidized-ferredoxins| + 1.0 coenzyme_A --> 1.0
isovaleryl-CoA + 1.0 CO2 + 2.0 |Reduced-ferredoxins| + 1.0 H+
isoleucine-ox/1_manual_1.2.7.7_HD-1.2.7.7-ile                             : 1.0 (S)-3-methyl-2-oxopentanoate + 2.0 |Oxidized-ferredoxins| + 1.0 coenzyme_A --> 1.0 2-
methylbutanoyl-CoA + 1.0 CO2 + 2.0 |Reduced-ferredoxins| + 1.0 H+

valine-ox/2_manual_2.3.1.-_HD-2.3.1.-val                                  : 1.0 isobutanoyl-CoA + 1.0 phosphate --> 1.0 isobutyryl_phosphate + 1.0 coenzyme_A
leucine-ox/2_manual_2.3.1.-_HD-2.3.1.-leu                                 : 1.0 isovaleryl-CoA + 1.0 phosphate --> 1.0 isovaleryl_phosphate + 1.0 coenzyme_A
isoleucine-ox/2_manual_2.3.1.-_HD-2.3.1.-ile                              : 1.0 2-methylbutanoyl-CoA + 1.0 phosphate --> 1.0 2-methylbutanoyl_phosphate + 1.0 coenzyme_A

valine-ox/3_manual_2.7.2.-_HD-2.7.2.-val                                  : 1.0 isobutyryl_phosphate + 1.0 ADP --> 1.0 isobutyrate + 1.0 ATP
leucine-ox/3_manual_2.7.2.-_HD-.7.2.-leu                                  : 1.0 isovaleryl_phosphate + 1.0 ADP --> 1.0 isovalerate + 1.0 ATP
isoleucine-ox/3_manual_2.7.2.-_HD-2.7.2.-ile                              : 1.0 2-methylbutanoyl_phosphate + 1.0 ADP --> 1.0 2-methylbutanoate + 1.0 ATP
#####################################################################################################################################################################################
#leu degr red
leu-red/1_manual_1.1.1.345_HD-1.1.1.345                                   : 1.0 D-2-hydroxyisocaproate + 1.0 NAD+ <-- 1.0 4-methyl-2-oxopentanoate + 1.0 H+ + 1.0 NADH
leu-red/2a_manual_2.8.3.-_HD-2.8.3.-leu-A                                 : 1.0 D-2-hydroxyisocaproyl-CoA + 1.0 isocaproate <=> 1.0 isocaproyl-CoA + 1.0 D-2-
hydroxyisocaproate
```

```
leu-red/3_manual_4.2.1.-_HD-4.2.1.-leu                                    : 1.0 D-2-hydroxyisocaproyl-CoA --> 1.0 2-isocaprenoyl-CoA + 1.0 H2O
leu-red/4_manual_1.3.98.-_HD-1.3.98.-leu                                  : 1.0 2-isocaprenoyl-CoA + 2.0 NADH + 2.0 |Oxidized-ferredoxins| <=> 1.0 isocaproyl-CoA + 2.0 |
Reduced-ferredoxins| + 2.0 NAD+ # Li et al. 2007: Coupled Ferredoxin and Crotonyl ...; Blast and position
#for the fatty acids
leu-red/2b_manual_2.8.3.-_HD-2.8.3.-leu-B                                 : 1.0 D-2-hydroxyisocaproyl-CoA + 1.0 acetate <=> 1.0 acetyl-CoA + 1.0 D-2-hydroxyisocaproate
leu-red/5_manual_2.3.1.-_HD-2.3.1.-leu-red                                : 1.0 isocaproyl-CoA + 1.0 phosphate --> 1.0 isocaproyl_phosphate + 1.0 coenzyme_A
########################################################################################################################################################################
#pyruvate decarbox.
formate_2.3.1.54_PYRUVFORMLY-RXN                                          : 1.0 formate + 1.0 acetyl-CoA <=> 1.0 coenzyme_A + 1.0 pyruvate  # source: MetaCyc, BRENDA,
KEGG (queried 2.3.1.54)
formate_2.3.1.54_KETOBUTFORMLY-RXN                                        : 1.0 coenzyme_A + 1.0 2-oxobutanoate --> 1.0 formate + 1.0 propanoyl-CoA  # source: MetaCyc,
BRENDA, KEGG (queried 2.3.1.54) // other EC numbers for this reaction: 2.3.1
########################################################################################################################################################################
#ferm. EtOH/PropOH/ButOH:
ferm-EtOH/1_1.2.1.10_ACETALD-DEHYDROG-RXN                                 : 1.0 NAD+ + 1.0 coenzyme_A + 1.0 acetaldehyde <=> 1.0 acetyl-CoA + 1.0 H+ + 1.0 NADH  #
source: MetaCyc, BRENDA, KEGG (queried 1.2.1.10)
ferm-EtOH/2_1.1.1.1_ALCOHOL-DEHYDROGENASE-NADP+-RXN_WOP                   : 1.0 ethanol + 1.0 NAD+ <=> 1.0 H+ + 1.0 NADH + 1.0 acetaldehyde  # source: MetaCyc, BRENDA,
KEGG (queried 1.1.1.1) // other EC numbers for this reaction: 1.1.1.71

ferm-PropOH/1_1.2.1.87_RXN-12736                                          : 1.0 coenzyme_A + 1.0 NAD+ + 1.0 propanal <=> 1.0 NADH + 1.0 H+ + 1.0 propanoyl-CoA  # source:
MetaCyc, BRENDA, KEGG (queried 1.2.1.87)
ferm-PropOH/2_1.1.1.1_RXN-13198                                           : 1.0 n-propanol + 1.0 NAD+ <=> 1.0 H+ + 1.0 propanal + 1.0 NADH  # source: MetaCyc, BRENDA,
KEGG (queried 1.1.1.1) // other EC numbers for this reaction: 1.1.1.202, 1.1.1.265

ferm-ButOH/1_1.2.1.10_1.2.1.87_BUTANAL-DEHYDROGENASE-RXN_WOP              : 1.0 butanal + 1.0 coenzyme_A + 1.0 NAD+ <=> 1.0 butanoyl-CoA + 1.0 NADH + 1.0 H+  # source:
MetaCyc, BRENDA, KEGG (queried 1.2.1.10, 1.2.1.87) // other EC numbers for this reaction: 1.2.1.57
ferm-ButOH/2_1.1.1.-_RXN-161                                              : 1.0 n-butanol + 1.0 NAD+ <-- 1.0 NADH + 1.0 butanal + 1.0 H+  # source: MetaCyc, BRENDA, KEGG
(queried 1.1.1.-) // other EC numbers for this reaction: 1.1.1, 1.1.1.265

ferm-IbutOH/1_1.2.1.10_RXN-13671                                          : 1.0 NAD+ + 1.0 coenzyme_A + 1.0 isobutanal <=> 1.0 H+ + 1.0 NADH + 1.0 isobutanoyl-CoA  #
source: MetaCyc, BRENDA (queried 1.2.1.10)
ferm-IbutOH/2_1.1.1.1_RXN-7657                                            : 1.0 NAD+ + 1.0 isobutanol <=> 1.0 NADH + 1.0 isobutanal + 1.0 H+  # source: MetaCyc, BRENDA
(queried 1.1.1.1)
########################################################################################################################################################################
#ferm acetate/propanoate
ferm-acetate/1_1.2.7.1_PYRUFLAVREDUCT-RXN                                 : 1.0 coenzyme_A + 1.0 pyruvate + 2.0 |Oxidized-ferredoxins| <=> 1.0 acetyl-CoA + 2.0 |Reduced-
ferredoxins| + 1.0 H+ + 1.0 CO2  # source: MetaCyc, KEGG (queried 1.2.7.1) // other EC numbers for this reaction: 1.2.7.11
ferm-acetate/2_2.3.1.8_PHOSACETYLTRANS-RXN                                : 1.0 acetyl-CoA + 1.0 phosphate <=> 1.0 acetyl_phosphate + 1.0 coenzyme_A  # source: MetaCyc,
BRENDA, KEGG (queried 2.3.1.8)
ferm-acetate/3_2.7.2.1_moddir_ACETATEKIN-RXN                              : 1.0 ATP + 1.0 acetate <=> 1.0 acetyl_phosphate + 1.0 ADP  # source: MetaCyc, BRENDA, KEGG
(queried 2.7.2.1) // other EC numbers for this reaction: 2.7.2.15

ferm-propanoate/1_1.2.7.1_2-OXOBUTYRATE-SYNTHASE-RXN                      : 1.0 2-oxobutanoate + 1.0 coenzyme_A + 2.0 |Oxidized-ferredoxins| <=> 1.0 H+ + 1.0 propanoyl-
CoA + 1.0 CO2 + 2.0 |Reduced-ferredoxins|  # source: MetaCyc, KEGG (queried 1.2.7.1) // other EC numbers for this reaction: 1.2.7
ferm-propanoate/2_2.3.1.222_2.3.1.8_PTAALT-RXN                            : 1.0 phosphate + 1.0 propanoyl-CoA <=> 1.0 coenzyme_A + 1.0 propanoyl_phosphate  # source:
MetaCyc, BRENDA, KEGG (queried 2.3.1.222, 2.3.1.8)
ferm-propanoate/3_2.7.2.1_moddir_PROPKIN-RXN                              : 1.0 propanoate + 1.0 ATP <=> 1.0 ADP + 1.0 propanoyl_phosphate  # source: MetaCyc, BRENDA,
KEGG (queried 2.7.2.1) // other EC numbers for this reaction: 2.7.2.15
########################################################################################################################################################################
#6.2.1.13
#Homolog to priviously characterized Isoenzyme I of Archaeoglobus fulgidus (Musfeldt and Schönheit, 2002)
acetyl-CoA-to-acid_6.2.1.13_ACETATE--COA-LIGASE-ADP-FORMING-RXN           : 1.0 ATP + 1.0 coenzyme_A + 1.0 acetate <=> 1.0 ADP + 1.0 acetyl-CoA + 1.0 phosphate  #
source: MetaCyc, BRENDA, KEGG (queried 6.2.1.13)
propanoyl-CoA-to-acid_6.2.1.13_PROPANOATECOA-LIGASE-RXN                   : 1.0 propanoate + 1.0 coenzyme_A + 1.0 ATP <=> 1.0 propanoyl-CoA + 1.0 phosphate + 1.0 ADP  #
source: MetaCyc, BRENDA, KEGG (queried 6.2.1.13)
butanoyl-CoA-to-acid_manual_6.2.1.13_HD-6.2.1.13                          : 1.0 ATP + 1.0 coenzyme_A + 1.0 butanoate <=> 1.0 ADP + 1.0 butanoyl-CoA + 1.0 phosphate
########################################################################################################################################################################
#fermentation to propanoate C (stickland-reductive-like)
propanoate-ferm/1a_2.8.3.-_PROPIONLACT-RXN                                          : 1.0 propanoyl-CoA + 1.0 (S)-lactate <=> 1.0 (S)-lactoyl-CoA + 1.0 propanoate  #
source: MetaCyc, KEGG (queried 2.8.3.-) // other EC numbers for this reaction: 2.8.3.1, 2.8.3
propanoate-ferm/2a_4.2.1.54_RXN-781                                                 : 1.0 (S)-lactoyl-CoA <=> 1.0 H2O + 1.0 acryloyl-CoA  # source: MetaCyc, BRENDA, KEGG
(queried 4.2.1.54) // other EC numbers for this reaction: 4.2.1
propanoate-ferm/1b_2.8.3.-_LACCOA-RXN                                               : 1.0 propanoyl-CoA + 1.0 (R)-lactate <=> 1.0 (R)-lactoyl-CoA + 1.0 propanoate  #
source: MetaCyc, BRENDA (queried 2.8.3.-) // other EC numbers for this reaction: 2.8.3.1, 2.8.3
```

```
propanoate-ferm/2b_4.2.1.54_LACTOYL-COA-DEHYDRATASE-RXN                                           : 1.0 (R)-lactoyl-CoA <=> 1.0 acryloyl-CoA + 1.0 H2O  # source: MetaCyc, BRENDA
(queried 4.2.1.54)
propanoate-ferm/3_manual_1.3.98.-_HD-1.3.98.-prop                                                 : 1.0 acryloyl-CoA + 2.0 NADH + 2.0 |Oxidized-ferredoxins| --> 1.0 propanoyl-CoA +
2.0 |Reduced-ferredoxins| + 2.0 NAD+ # Bifurcating like Li et al. 2007: Coupled Ferredoxin and Crotonyl ...
#propanoate-ferm/3_1.3.1.95_RXN-8568                                                               : 1.0 acryloyl-CoA + 1.0 NADH + 1.0 H+ --> 1.0 propanoyl-CoA + 1.0 NAD+  # source:
MetaCyc, BRENDA, KEGG (queried 1.3.1.95)
############################################################################################################################################################################
#lactate metabolism
#Both lactate dehydrogenases irreversible to prevent cycles
pyruvate-ferm-to-lactate_moddir_1.1.1.27_L-LACTATE-DEHYDROGENASE-RXN                              : 1.0 (S)-lactate + 1.0 NAD+ <-- 1.0 H+ + 1.0 NADH + 1.0 pyruvate  # source: MetaCyc,
BRENDA, KEGG (queried 1.1.1.27)
lactate-recemation_5.1.2.1_LACTATE-RACEMASE-RXN                                                   : 1.0 (S)-lactate <=> 1.0 (R)-lactate  # source: MetaCyc, BRENDA, KEGG (queried
5.1.2.1)
lactate-oxidation_manual_1.1.98.-_HD-1.1.98.-pyr                                                  : 1.0 pyruvate + 2.0 NADH + 2.0 |Oxidized-ferredoxins| <-- 1.0 (R)-lactate + 2.0 |
Reduced-ferredoxins| + 2.0 NAD+
############################################################################################################################################################################
#ferm. butanoate/butyrate
succinate-to-butyrate/1_2.8.3.18_RXN-8807                                                         : 1.0 acetate + 1.0 succinyl-CoA <=> 1.0 acetyl-CoA + 1.0 succinate  # source:
MetaCyc, BRENDA, KEGG (queried 2.8.3.18)
succinate-to-butyrate/2_1.2.1.76_RXN-8891                                                         : 1.0 succinate_semialdehyde + 1.0 NADP+ + 1.0 coenzyme_A <-- 1.0 succinyl-CoA + 1.0
H+ + 1.0 NADPH  # source: MetaCyc, BRENDA, KEGG (queried 1.2.1.76)
succinate-to-butyrate/3_1.1.1.61_RXN-6903_WOP                                                     : 1.0 NAD+ + 1.0 4-hydroxybutanoate <=> 1.0 H+ + 1.0 NADH + 1.0
succinate_semialdehyde  # source: MetaCyc, BRENDA, KEGG (queried 1.1.1.61) // other EC numbers for this reaction: 1.1.1
succinate-to-butyrate/4a_2.8.3.-_RXN-8889                                                         : 1.0 acetyl-CoA + 1.0 4-hydroxybutanoate --> 1.0 4-hydroxybutanoyl-CoA + 1.0 acetate
# source: MetaCyc, BRENDA, KEGG (queried 2.8.3.-) // other EC numbers for this reaction: 2.8.3.1, 2.8.3
succinate-to-butyrate/4b_manual_2.8.3.-_HD-4hbut                                                  : 1.0 4-hydroxybutanoate + 1.0 butanoyl-CoA --> 1.0 4-hydroxybutanoyl-CoA + 1.0
butanoate
succinate-to-butyrate/5a_4.2.1.120_RXN-8890                                                       : 1.0 4-hydroxybutanoyl-CoA <=> 1.0 H2O + 1.0 crotonyl-CoA  # source: MetaCyc,
BRENDA, KEGG (queried 4.2.1.120)
succinate-to-butyrate/5b_moddir_4.2.1.120_BS403853                                                : 1.0 4-hydroxybutanoyl-CoA <=> 1.0 vinylacetyl-CoA + 1.0 H2O  # source: BRENDA, KEGG
(queried 4.2.1.120)
succinate-to-butyrate/6_5.3.3.3_VINYLACETYL-COA-DELTA-ISOMERASE-RXN                               : 1.0 vinylacetyl-CoA <=> 1.0 crotonyl-CoA  # source: MetaCyc, BRENDA, KEGG (queried
5.3.3.3)

acetyl-CoA-to-butyrate-II/1_2.3.1.9_ACETYL-COA-ACETYLTRANSFER-RXN                                 : 2.0 acetyl-CoA <=> 1.0 acetoacetyl-CoA + 1.0 coenzyme_A  # source: MetaCyc, BRENDA,
KEGG (queried 2.3.1.9)
#The enzyme of C. difficile uses NADH (Aboulnaga et al., 2013)
acetyl-CoA-to-butyrate-II/2_1.1.1.35_HD-1.1.1.35                                                  : 1.0 (S)-3-hydroxybutanoyl-CoA + 1.0 NAD+ <-- 1.0 acetoacetyl-CoA + 1.0 NADH + 1.0
H+
acetyl-CoA-to-butyrate-II/3_4.2.1.150_RXN-11667                                                   : 1.0 (S)-3-hydroxybutanoyl-CoA <=> 1.0 crotonyl-CoA + 1.0 H2O  # source: MetaCyc,
BRENDA, KEGG (queried 4.2.1.150) // other EC numbers for this reaction: 4.2.1.17

crotonyl-CoA-to-butanoate/1_manual_1.3.98.-_HD-1.3.98.-but                                        : 1.0 crotonyl-CoA + 2.0 NADH + 2.0 |Oxidized-ferredoxins| <=> 1.0 butanoyl-CoA + 2.0
|Reduced-ferredoxins| + 2.0 NAD+
crotonyl-CoA-to-butanoate/2_2.3.1.19_PHOSPHATE-BUTYRYLTRANSFERASE-RXN                             : 1.0 phosphate + 1.0 butanoyl-CoA <=> 1.0 coenzyme_A + 1.0 butanoyl_phosphate  #
source: MetaCyc, BRENDA, KEGG (queried 2.3.1.19)
crotonyl-CoA-to-butanoate/3_moddir_2.7.2.7_BUTYRATE-KINASE-RXN                                    : 1.0 butanoate + 1.0 ATP <=> 1.0 ADP + 1.0 butanoyl_phosphate  # source: MetaCyc,
BRENDA, KEGG (queried 2.7.2.7)

(R)-3-hydroxybutanoate-ferm/1a_2.8.3.9_2.8.3.9-RXN                                                : 1.0 acetoacetate + 1.0 butanoyl-CoA <=> 1.0 acetoacetyl-CoA + 1.0 butanoate  #
source: MetaCyc, BRENDA, KEGG (queried 2.8.3.9)
(R)-3-hydroxybutanoate-ferm/1b_2.8.3.9_ACETOACETYL-COA-TRANSFER-RXN                               : 1.0 acetyl-CoA + 1.0 acetoacetate <=> 1.0 acetoacetyl-CoA + 1.0 acetate  # source:
MetaCyc, BRENDA, KEGG (queried 2.8.3.9) // other EC numbers for this reaction: 2.8.3.8, 2.8.3
(R)-3-hydroxybutanoate-ferm/2_1.1.1.30_3-HYDROXYBUTYRATE-DEHYDROGENASE-RXN                        : 1.0 (R)-3-hydroxybutanoate + 1.0 NAD+ <=> 1.0 NADH + 1.0 acetoacetate + 1.0 H+  #
source: MetaCyc, BRENDA, KEGG (queried 1.1.1.30)

#spontaneous (Han et al. 2011)
acetone-fermentation_spontaneous_ACETOACETATE-DECARBOXYLASE-RXN                                   : 1.0 acetoacetate + 1.0 H+ --> 1.0 acetone + 1.0 CO2  # source: MetaCyc, BRENDA,
KEGG
acetone-fermentation-ex_spontaneous_ACETOACETATE-DECARBOXYLASE-RXN                                : 1.0 acetoacetate[ex] + 1.0 H+[ex] --> 1.0 acetone[ex] + 1.0 CO2[ex]  # source:
MetaCyc, BRENDA, KEGG
############################################################################################################################################################################
#ferm. pentanoate
```

```
pentanoate-ferm/1_2.3.1.9_RXN-12561                                        : 1.0 acetyl-CoA + 1.0 propanoyl-CoA <=> 1.0 coenzyme_A + 1.0 beta-ketovaleryl-CoA  # source: MetaCyc, BRENDA (queried 2.3.1.9) // other EC numbers for this reaction: 2.3.1.16
pentanoate-ferm/2_manual_1.1.1.157_HD-1.1.1.157-pent                       : 1.0 (R)-3-hydroxypentanoyl-CoA + 1.0 NAD+ <-- 1.0 H+ + 1.0 NADH + 1.0 beta-ketovaleryl-CoA
pentanoate-ferm/3_manual_4.2.1.150_HD-4.2.1.150-pent                       : 1.0 (R)-3-hydroxypentanoyl-CoA --> 1.0 2-pentenoyl-CoA + 1.0 H2O
pentanoate-ferm/4_manual_1.3.98.-_HD-1.3.98.-pent                          : 1.0 2-pentenoyl-CoA + 2.0 NADH + 2.0 |Oxidized-ferredoxins| <=> 1.0 pentanoyl-CoA + 2.0 |Reduced-ferredoxins| + 2.0 NAD+
pentanoate-ferm/5_manual_2.3.1.19_HD-2.3.1.19-pent                         : 1.0 pentanoyl-CoA + 1.0 phosphate --> 1.0 pentanoyl_phosphate + 1.0 coenzyme_A
pentanoate-ferm/6_manual_2.7.2.7_HD-2.7.2.7-pent                           : 1.0 pentanoyl_phosphate + 1.0 ADP --> 1.0 pentanoate + 1.0 ATP
############################################################################################################################################################################
#ferm. hexanoate
hexanoate-ferm/1_2.3.1.9_RXN-12565                                         : 1.0 acetyl-CoA + 1.0 butanoyl-CoA --> 1.0 coenzyme_A + 1.0 3-oxohexanoyl-CoA  # source: MetaCyc, BRENDA, KEGG (queried 2.3.1.9) // other EC numbers for this reaction: 2.3.1.16, 2.3.1.155
hexanoate-ferm/2_manual_1.1.1.157_HD-1.1.1.157-hex                         : 1.0 (R)-3-hydroxyhexanoyl-CoA + 1.0 NAD+ <-- 1.0 H+ + 1.0 NADH + 1.0 3-oxohexanoyl-CoA
hexanoate-ferm/3_manual_4.2.1.150_HD-4.2.1.150-hex                         : 1.0 (R)-3-hydroxyhexanoyl-CoA --> 1.0 2-hexenoyl-CoA + 1.0 H2O
hexanoate-ferm/4_manual_1.3.98.-_HD-1.3.98.-hex                            : 1.0 2-hexenoyl-CoA + 2.0 NADH + 2.0 |Oxidized-ferredoxins| <=> 1.0 hexanoyl-CoA + 2.0 |Reduced-ferredoxins| + 2.0 NAD+
hexanoate-ferm/5_manual_2.3.1.19_HD-2.3.1.19-hex                           : 1.0 hexanoyl-CoA + 1.0 phosphate --> 1.0 hexanoyl_phosphate + 1.0 coenzyme_A
hexanoate-ferm/6_manual_2.7.2.7_HD-2.7.2.7-hex                             : 1.0 hexanoyl_phosphate + 1.0 ADP --> 1.0 hexanoate + 1.0 ATP
############################################################################################################################################################################
#5-aminoimidazole ribonucleotide biosynthesis II
5-aminoimidazole-ribonucleotide-biosyn-II/1_2.4.2.14_PRPPAMIDOTRANS-RXN                       : 1.0 diphosphate + 1.0 L-glutamate + 1.0 5-phospho-beta-D-ribosylamine <=> 1.0 L-glutamine + 1.0 H2O + 1.0 5-phospho-alpha-D-ribose_1-diphosphate  # source: MetaCyc, BRENDA, KEGG (queried 2.4.2.14)
5-aminoimidazole-ribonucleotide-biosyn-II/2_6.3.4.13_GLYRIBONUCSYN-RXN                        : 1.0 glycine + 1.0 ATP + 1.0 5-phospho-beta-D-ribosylamine --> 1.0 H+ + 1.0 phosphate + 1.0 ADP + 1.0 N1-(5-phospho-beta-D-ribosyl)glycinamide  # source: MetaCyc, BRENDA, KEGG (queried 6.3.4.13)
5-aminoimidazole-ribonucleotide-biosyn-II/3_2.1.2.2_GART-RXN                                  : 1.0 10-formyltetrahydrofolate + 1.0 N1-(5-phospho-beta-D-ribosyl)glycinamide <=> 1.0 tetrahydropteroyl_tri-L-glutamate + 1.0 N2-formyl-N1-(5-phospho-beta-D-ribosyl)glycinamide + 1.0 H+  # source: MetaCyc (queried 2.1.2.2)
5-aminoimidazole-ribonucleotide-biosyn-II/4_6.3.5.3_FGAMSYN-RXN                               : 1.0 H2O + 1.0 L-glutamine + 1.0 ATP + 1.0 N2-formyl-N1-(5-phospho-beta-D-ribosyl)glycinamide --> 1.0 ADP + 1.0 2-(formamido)-N1-(5-phospho-beta-D-ribosyl)acetamidine + 1.0 H+ + 1.0 phosphate + 1.0 L-glutamate  # source: MetaCyc, BRENDA, KEGG (queried 6.3.5.3)
5-aminoimidazole-ribonucleotide-biosyn-II/5_6.3.3.1_AIRS-RXN                                  : 1.0 2-(formamido)-N1-(5-phospho-beta-D-ribosyl)acetamidine + 1.0 ATP --> 1.0 phosphate + 1.0 ADP + 1.0 5-amino-1-(5-phospho-beta-D-ribosyl)imidazole + 1.0 H+  # source: MetaCyc, BRENDA, KEGG (queried 6.3.3.1)
############################################################################################################################################################################
#inosine-5'-phosphate biosynthesis
#E. coli class I 5.4.99.18 (PurE) without 6.3.4.18 (PurK) sufficient in vitro under high bicarbonate concentrations (Meyer et al., 1992).
#Overexpression of purE complements purK deletion in E. coli (Patrick et al., 2007).
#Several archea with class I 5.4.99.18 (PurE) and missing purK found (Brown et al., 2011)
inosine-5'-phosphate-biosyn/1_manual_spontaneous_5.4.99.18_HD-5.4.99.18                       : 1.0 hydrogen_carbonate + 1.0 5-amino-1-(5-phospho-beta-D-ribosyl)imidazole --> 1.0 H+ + 1.0 N5-carboxyaminoimidazole_ribonucleotide  # metacyc
inosine-5'-phosphate-biosyn/2_5.4.99.18_RXN0-743                                              : 1.0 N5-carboxyaminoimidazole_ribonucleotide --> 1.0 5-amino-1-(5-phospho-D-ribosyl)imidazole-4-carboxylate  # source: MetaCyc, BRENDA, KEGG (queried 5.4.99.18)
inosine-5'-phosphate-biosyn/3_6.3.2.6_SAICARSYN-RXN                                           : 1.0 5-amino-1-(5-phospho-D-ribosyl)imidazole-4-carboxylate + 1.0 L-aspartate + 1.0 ATP --> 1.0 5'-phosphoribosyl-4-(N-succinocarboxamide)-5-aminoimidazole + 1.0 ADP + 1.0 H+ + 1.0 phosphate  # source: MetaCyc, BRENDA, KEGG (queried 6.3.2.6)
inosine-5'-phosphate-biosyn/4_4.3.2.2_AICARSYN-RXN                                            : 1.0 5'-phosphoribosyl-4-(N-succinocarboxamide)-5-aminoimidazole <=> 1.0 5-amino-1-(5-phospho-D-ribosyl)imidazole-4-carboxamide + 1.0 fumarate  # source: MetaCyc, BRENDA, KEGG (queried 4.3.2.2)
inosine-5'-phosphate-biosyn/5_2.1.2.3_AICARTRANSFORM-RXN                                      : 1.0 10-formyltetrahydrofolate + 1.0 5-amino-1-(5-phospho-D-ribosyl)imidazole-4-carboxamide <=> 1.0 tetrahydropteroyl_tri-L-glutamate + 1.0 5-formamido-1-(5-phospho-D-ribosyl)-imidazole-4-carboxamide  # source: MetaCyc (queried 2.1.2.3)
inosine-5'-phosphate-biosyn/6_moddir_3.5.4.10_2.1.2.3_IMPCYCLOHYDROLASE-RXN                   : 1.0 H2O + 1.0 IMP <=> 1.0 5-formamido-1-(5-phospho-D-ribosyl)-imidazole-4-carboxamide  # source: MetaCyc, BRENDA, KEGG (queried 3.5.4.10, 2.1.2.3)
############################################################################################################################################################################
#tRNA
tRNA-charging_6.1.1.10_METHIONINE--TRNA-LIGASE-RXN                      : 1.0 ATP + 1.0 |MET-tRNAs| + 1.0 H+ + 1.0 L-methionine --> 1.0 |Charged-MET-tRNAs| + 1.0 diphosphate + 1.0 AMP  # source: MetaCyc, KEGG (queried 6.1.1.10)
tRNA-charging_6.1.1.11_SERINE--TRNA-LIGASE-RXN                          : 1.0 H+ + 1.0 ATP + 1.0 |SER-tRNAs| + 1.0 L-serine --> 1.0 |Charged-SER-tRNAs| + 1.0 diphosphate + 1.0 AMP  # source: MetaCyc, KEGG (queried 6.1.1.11)
tRNA-charging_6.1.1.12_ASPARTATE--TRNA-LIGASE-RXN                       : 1.0 |ASP-tRNAs| + 1.0 H+ + 1.0 L-aspartate + 1.0 ATP --> 1.0 |Charged-ASP-tRNAs| + 1.0 diphosphate + 1.0 AMP  # source: MetaCyc, KEGG (queried 6.1.1.12) // other EC numbers for this reaction: 6.1.1.23
tRNA-charging_6.1.1.14_GLYCINE--TRNA-LIGASE-RXN                         : 1.0 |GLY-tRNAs| + 1.0 H+ + 1.0 glycine + 1.0 ATP --> 1.0 |Charged-GLY-tRNAs| + 1.0 diphosphate + 1.0 AMP  # source: MetaCyc, KEGG (queried 6.1.1.14)
tRNA-charging_6.1.1.15_PROLINE--TRNA-LIGASE-RXN                         : 1.0 ATP + 1.0 L-proline + 1.0 |PRO-tRNAs| + 1.0 H+ --> 1.0 |Charged-PRO-tRNAs| + 1.0 diphosphate + 1.0 AMP  # source: MetaCyc, KEGG (queried 6.1.1.15)
tRNA-charging_6.1.1.16_CYSTEINE--TRNA-LIGASE-RXN                        : 1.0 |CYS-tRNAs| + 1.0 H+ + 1.0 L-cysteine + 1.0 ATP --> 1.0 AMP + 1.0 |Charged-CYS-tRNAs| + 1.0 diphosphate  # source: MetaCyc, KEGG (queried 6.1.1.16)
```

```
tRNA-charging_6.1.1.17_GLURS-RXN                                        : 1.0 |GLT-tRNAs| + 1.0 H+ + 1.0 L-glutamate + 1.0 ATP --> 1.0 |Charged-GLT-tRNAs| + 1.0 AMP + 1.0
diphosphate  # source: MetaCyc, KEGG (queried 6.1.1.17)
tRNA-charging_6.1.1.18_GLUTAMINE--TRNA-LIGASE-RXN                       : 1.0 H+ + 1.0 L-glutamine + 1.0 ATP + 1.0 |GLN-tRNAs| --> 1.0 |Charged-GLN-tRNAs| + 1.0 diphosphate + 1.0
AMP  # source: MetaCyc, KEGG (queried 6.1.1.18)
tRNA-charging_6.1.1.19_ARGININE--TRNA-LIGASE-RXN                        : 1.0 L-arginine + 1.0 |ARG-tRNAs| + 1.0 H+ + 1.0 ATP --> 1.0 |Charged-ARG-tRNAs| + 1.0 diphosphate + 1.0
AMP  # source: MetaCyc, KEGG (queried 6.1.1.19)
tRNA-charging_6.1.1.1_TYROSINE--TRNA-LIGASE-RXN                         : 1.0 ATP + 1.0 |TYR-tRNAs| + 1.0 L-tyrosine + 1.0 H+ --> 1.0 |Charged-TYR-tRNAs| + 1.0 diphosphate + 1.0
AMP  # source: MetaCyc, KEGG (queried 6.1.1.1)
tRNA-charging_6.1.1.20_PHENYLALANINE--TRNA-LIGASE-RXN                   : 1.0 H+ + 1.0 L-phenylalanine + 1.0 ATP + 1.0 |PHE-tRNAs| --> 1.0 |Charged-PHE-tRNAs| + 1.0 AMP + 1.0
diphosphate  # source: MetaCyc, KEGG (queried 6.1.1.20)
tRNA-charging_6.1.1.21_HISTIDINE--TRNA-LIGASE-RXN                       : 1.0 |HIS-tRNAs| + 1.0 H+ + 1.0 L-histidine + 1.0 ATP --> 1.0 |Charged-HIS-tRNAs| + 1.0 diphosphate + 1.0
AMP  # source: MetaCyc, KEGG (queried 6.1.1.21)
tRNA-charging_6.1.1.22_ASPARAGINE--TRNA-LIGASE-RXN                      : 1.0 |ASN-tRNAs| + 1.0 H+ + 1.0 ATP + 1.0 L-asparagine --> 1.0 |Charged-ASN-tRNAs| + 1.0 diphosphate + 1.0
AMP  # source: MetaCyc, KEGG (queried 6.1.1.22)
tRNA-charging_6.1.1.2_TRYPTOPHAN--TRNA-LIGASE-RXN                       : 1.0 ATP + 1.0 |TRP-tRNAs| + 1.0 L-tryptophan + 1.0 H+ --> 1.0 |Charged-TRP-tRNAs| + 1.0 diphosphate + 1.0
AMP  # source: MetaCyc, KEGG (queried 6.1.1.2)
tRNA-charging_6.1.1.3_THREONINE--TRNA-LIGASE-RXN                        : 1.0 |THR-tRNAs| + 1.0 L-threonine + 1.0 H+ + 1.0 ATP --> 1.0 |Charged-THR-tRNAs| + 1.0 diphosphate + 1.0
AMP  # source: MetaCyc, KEGG (queried 6.1.1.3)
tRNA-charging_6.1.1.4_LEUCINE--TRNA-LIGASE-RXN                          : 1.0 ATP + 1.0 |LEU-tRNAs| + 1.0 H+ + 1.0 L-leucine --> 1.0 |Charged-LEU-tRNAs| + 1.0 diphosphate + 1.0
AMP  # source: MetaCyc, KEGG (queried 6.1.1.4)
tRNA-charging_6.1.1.5_ISOLEUCINE--TRNA-LIGASE-RXN                       : 1.0 ATP + 1.0 |ILE-tRNAs| + 1.0 H+ + 1.0 L-isoleucine --> 1.0 |Charged-ILE-tRNAs| + 1.0 AMP + 1.0
diphosphate  # source: MetaCyc, KEGG (queried 6.1.1.5)
tRNA-charging_6.1.1.6_LYSINE--TRNA-LIGASE-RXN                           : 1.0 ATP + 1.0 |LYS-tRNAs| + 1.0 H+ + 1.0 L-lysine --> 1.0 |Charged-LYS-tRNAs| + 1.0 diphosphate + 1.0 AMP
# source: MetaCyc, KEGG (queried 6.1.1.6)
tRNA-charging_6.1.1.7_ALANINE--TRNA-LIGASE-RXN                          : 1.0 H+ + 1.0 |ALA-tRNAs| + 1.0 L-alanine + 1.0 ATP --> 1.0 AMP + 1.0 |Charged-ALA-tRNAs| + 1.0
diphosphate  # source: MetaCyc, KEGG (queried 6.1.1.7)
tRNA-charging_6.1.1.9_VALINE--TRNA-LIGASE-RXN                           : 1.0 H+ + 1.0 ATP + 1.0 |VAL-tRNAs| + 1.0 L-valine --> 1.0 |Charged-VAL-tRNAs| + 1.0 diphosphate + 1.0 AMP
# source: MetaCyc, KEGG (queried 6.1.1.9)
selenocysteine-biosynthesis_6.1.1.11_RXN0-2161                          : 1.0 |SEC-tRNAs| + 1.0 L-serine + 1.0 H+ + 1.0 ATP --> 1.0 AMP + 1.0 |L-seryl-SEC-tRNAs| + 1.0 diphosphate
# source: MetaCyc, KEGG (queried 6.1.1.11)
############################################################################################################################################################################
#NAD biosyn I/NADP
NAD-biosyn-I/1a_1.4.3.16_L-ASPARTATE-OXID-RXN                           : 1.0 oxygen + 1.0 L-aspartate --> 1.0 2-iminosuccinate + 1.0 hydrogen_peroxide + 1.0 H+  # source:
MetaCyc, BRENDA, KEGG (queried 1.4.3.16)
NAD-biosyn-I/1b_1.4.3.16_RXN-9772                                       : 1.0 L-aspartate + 1.0 fumarate --> 1.0 2-iminosuccinate + 1.0 H+ + 1.0 succinate  # source: MetaCyc,
BRENDA (queried 1.4.3.16) // other EC numbers for this reaction: 1.5.99
NAD-biosyn-I/2_2.5.1.72_QUINOLINATE-SYNTHA-RXN                          : 1.0 2-iminosuccinate + 1.0 glycerone_phosphate --> 1.0 quinolinate + 1.0 phosphate + 2.0 H2O  # source:
MetaCyc, BRENDA, KEGG (queried 2.5.1.72)
NAD-biosyn-I/3_2.4.2.19_QUINOPRIBOTRANS-RXN                             : 1.0 diphosphate + 1.0 beta-nicotinate_D-ribonucleotide + 1.0 CO2 <-- 1.0 5-phospho-alpha-D-ribose_1-
diphosphate + 2.0 H+ + 1.0 quinolinate  # source: MetaCyc, BRENDA, KEGG (queried 2.4.2.19)
NAD-biosyn-I/4_2.7.7.18_NICONUCADENYLYLTRAN-RXN                         : 1.0 beta-nicotinate_D-ribonucleotide + 1.0 H+ + 1.0 ATP --> 1.0 nicotinate_adenine_dinucleotide + 1.0
diphosphate  # source: MetaCyc, BRENDA, KEGG (queried 2.7.7.18) // other EC numbers for this reaction: 2.7.7.1
NAD-biosyn-I/5_6.3.1.5_NAD-SYNTH-NH3-RXN                                : 1.0 ATP + 1.0 ammonium + 1.0 nicotinate_adenine_dinucleotide --> 1.0 NAD+ + 1.0 AMP + 1.0 H+ + 1.0
diphosphate  # source: MetaCyc, BRENDA, KEGG (queried 6.3.1.5) // other EC numbers for this reaction: 6.3.5.1
NADP-biosyn_2.7.1.23_NAD-KIN-RXN                                        : 1.0 NAD+ + 1.0 ATP --> 1.0 ADP + 1.0 NADP+ + 1.0 H+  # source: MetaCyc, BRENDA, KEGG (queried 2.7.1.23)
############################################################################################################################################################################
#NADH repair
NADH-repair_spontaneous_RXN-12753                                       : 1.0 NADH + 1.0 H2O --> 1.0 (S)-NADHX  # source: MetaCyc
NADH-repair_spontaneous_RXN-12754                                       : 1.0 H2O + 1.0 NADH --> 1.0 (R)-NADHX  # source: MetaCyc
NADH-repair_5.1.99.6_RXN-12752                                          : 1.0 (R)-NADHX --> 1.0 (S)-NADHX  # source: MetaCyc, BRENDA, KEGG (queried 5.1.99.6)
NADH-repair_4.2.1.136_RXN0-6727                                         : 1.0 (S)-NADHX + 1.0 ADP --> 1.0 H+ + 1.0 phosphate + 1.0 NADH + 1.0 AMP  # source: MetaCyc, BRENDA, KEGG
(queried 4.2.1.136)

NADPH-repair_manual_spontaneous_HD-(S)-NADPHX                           : 1.0 NADPH + 1.0 H2O --> 1.0 (S)-NADPHX
NADPH-repair_manual_spontaneous_HD-(R)-NADPHX                           : 1.0 H2O + 1.0 NADPH --> 1.0 (R)-NADPHX
NADPH-repair_5.1.99.6_RXN-13142                                         : 1.0 (R)-NADPHX <=> 1.0 (S)-NADPHX  # source: MetaCyc, BRENDA, KEGG (queried 5.1.99.6)
NADPH-repair_4.2.1.136_RXN-13141                                        : 1.0 (S)-NADPHX + 1.0 ADP --> 1.0 NADPH + 1.0 AMP + 1.0 H+ + 1.0 phosphate  # source: MetaCyc, BRENDA,
KEGG (queried 4.2.1.136)
############################################################################################################################################################################
#nicotine transitions
nicotine-transitions-A/1_3.1.3.5_RXN-14227                              : 1.0 beta-nicotinate_D-ribonucleotide + 1.0 H2O --> 1.0 beta-D-ribosylnicotinate + 1.0 phosphate  #
source: MetaCyc, KEGG (queried 3.1.3.5) // other EC numbers for this reaction: 3.1.3
nicotine-transitions-A/2_2.4.2.1_R02295                                 : 1.0 phosphate + 1.0 beta-D-ribosylnicotinate <=> 1.0 alpha-D-ribose-1-phosphate + 1.0 nicotinate  #
source: KEGG (queried 2.4.2.1) // balanced // contains implicit reactant(s): H+, H2O
```

nicotine-transitions-B_3.6.1.9_BS165396   : 1.0 nicotinate_adenine_dinucleotide + 1.0 H2O --> 1.0 AMP + 1.0 beta-nicotinate_D-ribonucleotide # source: BRENDA, KEGG (queried 3.6.1.9) // other EC numbers for this reaction: 3.6.1.22

nicotine-transitions-C/1_3.6.1.9_NADPYROPHOSPHAT-RXN   : 1.0 H2O + 1.0 NAD+ --> 2.0 H+ + 1.0 beta-nicotinamide_D-ribonucleotide + 1.0 AMP # source: MetaCyc, BRENDA, KEGG (queried 3.6.1.9) // other EC numbers for this reaction: 3.6.1.22
nicotine-transitions-C/2_3.1.3.5_3.1.3.2_RXN-5841   : 1.0 H2O + 1.0 beta-nicotinamide_D-ribonucleotide --> 1.0 1-(beta-D_ribofuranosyl)nicotinamide + 1.0 phosphate # source: MetaCyc, BRENDA, KEGG (queried 3.1.3.5, 3.1.3.2) // other EC numbers for this reaction: 3.1.3
nicotine-transitions-C/3_3.2.2.1_BS94861   : 1.0 1-(beta-D_ribofuranosyl)nicotinamide + 1.0 H2O --> 1.0 nicotinamide + 1.0 beta-D-ribopyranose # source: BRENDA, KEGG (queried 3.2.2.1) // other EC numbers for this reaction: 3.2.2.3, 3.2.2.8
nicotine-transitions-C/4a_3.5.1.19_NICOTINAMID-RXN   : 1.0 H2O + 1.0 nicotinamide --> 1.0 ammonium + 1.0 nicotinate # source: MetaCyc, BRENDA, KEGG (queried 3.5.1.19)
nicotine-transitions-C/4b_2.4.2.1_RXN0-7092   : 1.0 alpha-D-ribose-1-phosphate + 1.0 H+ + 1.0 nicotinamide <=> 1.0 phosphate + 1.0 1-(beta-D_ribofuranosyl)nicotinamide # source: MetaCyc, BRENDA, KEGG (queried 2.4.2.1) // other EC numbers for this reaction: 2.4.2
nicotine-transitions-C/5_2.7.7.18_2.7.7.1-RXN   : 1.0 H+ + 1.0 beta-nicotinamide_D-ribonucleotide + 1.0 ATP --> 1.0 diphosphate + 1.0 NAD+ # source: MetaCyc, BRENDA, KEGG (queried 2.7.7.18) // other EC numbers for this reaction: 2.7.7.1
##
#FAD biosyn I
flavin-biosyn-I/1_3.5.4.25_GTP-CYCLOHYDRO-II-RXN   : 3.0 H2O + 1.0 GTP --> 1.0 diphosphate + 1.0 formate + 1.0 2,5-diamino-6-(5-phospho-D-ribosylamino)pyrimidin-4(3H)-one + 2.0 H+ # source: MetaCyc, BRENDA, KEGG (queried 3.5.4.25)
flavin-biosyn-I/2_3.5.4.26_RIBOFLAVINSYNDEAM-RXN   : 1.0 H2O + 1.0 H+ + 1.0 2,5-diamino-6-(5-phospho-D-ribosylamino)pyrimidin-4(3H)-one --> 1.0 ammonium + 1.0 5-amino-6-(5-phospho-D-ribosylamino)uracil # source: MetaCyc, BRENDA, KEGG (queried 3.5.4.26)
flavin-biosyn-I/3_1.1.1.193_RIBOFLAVINSYNREDUC-RXN   : 1.0 NADP+ + 1.0 5-amino-6-(5-phospho-D-ribitylamino)uracil <-- 1.0 H+ + 1.0 5-amino-6-(5-phospho-D-ribosylamino)uracil + 1.0 NADPH # source: MetaCyc, BRENDA, KEGG (queried 1.1.1.193)
flavin-biosyn-I/4_3.1.3.-_RIBOPHOSPHAT-RXN   : 1.0 H2O + 1.0 5-amino-6-(5-phospho-D-ribitylamino)uracil --> 1.0 5-amino-6-(D-ribitylamino)uracil + 1.0 phosphate # source: MetaCyc, KEGG (queried 3.1.3.-) // other EC numbers for this reaction: 3.1.3
flavin-biosyn-I/5_4.1.99.12_DIOHBUTANONEPSYN-RXN   : 1.0 D-ribulose_5-phosphate --> 1.0 H+ + 1.0 1-deoxy-L-glycero-tetrulose_4-phosphate + 1.0 formate # source: MetaCyc, BRENDA, KEGG (queried 4.1.99.12)
flavin-biosyn-I/6_2.5.1.78_LUMAZINESYN-RXN   : 1.0 1-deoxy-L-glycero-tetrulose_4-phosphate + 1.0 5-amino-6-(D-ribitylamino)uracil --> 1.0 H+ + 1.0 phosphate + 1.0 6,7-dimethyl-8-(1-D-ribityl)lumazine + 2.0 H2O # source: MetaCyc, BRENDA, KEGG (queried 2.5.1.78)
flavin-biosyn-I/7_2.5.1.9_RIBOFLAVIN-SYN-RXN   : 1.0 H+ + 2.0 6,7-dimethyl-8-(1-D-ribityl)lumazine --> 1.0 5-amino-6-(D-ribitylamino)uracil + 1.0 riboflavin # source: MetaCyc, BRENDA, KEGG (queried 2.5.1.9)
flavin-biosyn-I/8_2.7.1.26_RIBOFLAVINKIN-RXN   : 1.0 riboflavin + 1.0 ATP --> 1.0 FMN + 1.0 ADP + 1.0 H+ # source: MetaCyc, BRENDA, KEGG (queried 2.7.1.26)
flavin-biosyn-I/9_2.7.7.2_FADSYN-RXN   : 1.0 H+ + 1.0 FMN + 1.0 ATP --> 1.0 FAD + 1.0 diphosphate # source: MetaCyc, BRENDA, KEGG (queried 2.7.7.2)
##
#phosphopantothenate biosynthesis I
phosphopantothenate-biosyn-I/1_2.1.2.11_3-CH3-2-OXOBUTANOATE-OH-CH3-XFER-RXN   : 1.0 5,10-methylene-tetrahydrofolate + 1.0 H2O + 1.0 3-methyl-2-oxobutanoate <=> 1.0 2-dehydropantoate + 1.0 tetrahydropteroyl_tri-L-glutamate # source: MetaCyc (queried 2.1.2.11)
phosphopantothenate-biosyn-I/2_1.1.1.169_2-DEHYDROPANTOATE-REDUCT-RXN   : 1.0 (R)-pantoate + 1.0 NADP+ <-- 1.0 NADPH + 1.0 2-dehydropantoate + 1.0 H+ # source: MetaCyc, BRENDA, KEGG (queried 1.1.1.169)
phosphopantothenate-biosyn-I/3_6.3.2.1_PANTOATE-BETA-ALANINE-LIG-RXN   : 1.0 (R)-pantoate + 1.0 beta-alanine + 1.0 ATP --> 1.0 diphosphate + 1.0 (R)-pantothenate + 1.0 AMP + 1.0 H+ # source: MetaCyc, BRENDA, KEGG (queried 6.3.2.1)
phosphopantothenate-biosyn-I/4_2.7.1.33_PANTOTHENATE-KIN-RXN   : 1.0 (R)-pantothenate + 1.0 ATP --> 1.0 (R)-4'-phosphopantothenate + 1.0 H+ + 1.0 ADP # source: MetaCyc, BRENDA, KEGG (queried 2.7.1.33)
##
#coenzyme A biosyn
coenzyme-A-biosyn/1_6.3.2.5_P-PANTOCYSLIG-RXN   : 1.0 L-cysteine + 1.0 CTP + 1.0 (R)-4'-phosphopantothenate --> 1.0 diphosphate + 1.0 CMP + 1.0 R-4'-phosphopantothenoyl-L-cysteine + 1.0 H+ # source: MetaCyc, BRENDA, KEGG (queried 6.3.2.5)
coenzyme-A-biosyn/2_4.1.1.36_P-PANTOCYSDECARB-RXN   : 1.0 R-4'-phosphopantothenoyl-L-cysteine + 1.0 H+ --> 1.0 CO2 + 1.0 4'-phosphopantetheine # source: MetaCyc, BRENDA, KEGG (queried 4.1.1.36)
coenzyme-A-biosyn/3_2.7.7.3_PANTEPADENYLYLTRAN-RXN   : 1.0 H+ + 1.0 4'-phosphopantetheine + 1.0 ATP --> 1.0 diphosphate + 1.0 3'-dephospho-CoA # source: MetaCyc, BRENDA, KEGG (queried 2.7.7.3)
coenzyme-A-biosyn/4_2.7.1.24_DEPHOSPHOCOAKIN-RXN   : 1.0 ATP + 1.0 3'-dephospho-CoA --> 1.0 ADP + 1.0 coenzyme_A + 1.0 H+ # source: MetaCyc, BRENDA, KEGG (queried 2.7.1.24)
##
#4-aminobenzoate biosynthesis
4-aminobenzoate-biosyn/1_2.6.1.85_PABASYN-RXN   : 1.0 chorismate + 1.0 L-glutamine <=> 1.0 4-amino-4-deoxychorismate + 1.0 L-glutamate # source: MetaCyc, BRENDA, KEGG (queried 2.6.1.85)
4-aminobenzoate-biosyn/2_4.1.3.38_ADCLY-RXN   : 1.0 4-amino-4-deoxychorismate <=> 1.0 4-aminobenzoate + 1.0 pyruvate + 1.0 H+ # source: MetaCyc, BRENDA, KEGG (queried 4.1.3.38)
##
#6-hydroxymethyl-dihydropterin diphosphate biosynthesis I

```
6-hydroxymethyl-dihydropterin-PP-biosyn-I/1_3.5.4.16_R00428                                  : 1.0 GTP + 1.0 H2O --> 1.0 Formamidopyrimidine_nucleoside_triphosphate  # source: KEGG
(queried 3.5.4.16)
6-hydroxymethyl-dihydropterin-PP-biosyn-I/2_3.5.4.16_R05046                                  : 1.0 Formamidopyrimidine_nucleoside_triphosphate + 1.0 H2O --> 1.0 formate + 1.0 N-
(2,5-diamino-6-oxo-1,6-dihydropyrimidin-4-yl)-beta-D-ribofuranosylamine_5'-triphosphate  # source: KEGG (queried 3.5.4.16)
6-hydroxymethyl-dihydropterin-PP-biosyn-I/3_3.5.4.16_R05048                                  : 1.0 N-(2,5-diamino-6-oxo-1,6-dihydropyrimidin-4-yl)-beta-D-ribofuranosylamine_5'-
triphosphate <=> 1.0 2,5-Diamino-6-(5'-triphosphoryl-3,4'-trihydroxy-2'-oxopentyl)-amino-4-oxopyrimidine  # source: KEGG (queried 3.5.4.16)
6-hydroxymethyl-dihydropterin-PP-biosyn-I/4_moddir_3.5.4.16_R04639                           : 1.0 7,8-dihydroneopterin_3'-triphosphate + 1.0 H2O <=> 1.0 2,5-Diamino-6-(5'-
triphosphoryl-3,4'-trihydroxy-2'-oxopentyl)-amino-4-oxopyrimidine  # source: KEGG (queried 3.5.4.16)
6-hydroxymethyl-dihydropterin-PP-biosyn-I/5_3.6.1.67_H2NEOPTERINP3PYROPHOSPHOHYDRO-RXN       : 1.0 H2O + 1.0 7,8-dihydroneopterin_3'-triphosphate --> 1.0 diphosphate + 1.0 7,8-
dihydroneopterin_3'-phosphate + 1.0 H+  # source: MetaCyc, BRENDA, KEGG (queried 3.6.1.67) // other EC numbers for this reaction: 3.6.1.B15
6-hydroxymethyl-dihydropterin-PP-biosyn-I/6_3.6.1.-_DIHYDRONEOPTERIN-MONO-P-DEPHOS-RXN       : 1.0 H2O + 1.0 7,8-dihydroneopterin_3'-phosphate --> 1.0 7,8-dihydroneopterin + 1.0
phosphate  # source: MetaCyc, KEGG (queried 3.6.1.-) // other EC numbers for this reaction: 3.6.1
6-hydroxymethyl-dihydropterin-PP-biosyn-I/7_4.1.2.25_H2NEOPTERINALDOL-RXN                    : 1.0 7,8-dihydroneopterin <=> 1.0 glycolaldehyde + 1.0 6-hydroxymethyl-7,8-
dihydropterin  # source: MetaCyc, BRENDA, KEGG (queried 4.1.2.25)
6-hydroxymethyl-dihydropterin-PP-biosyn-I/8_2.7.6.3_H2PTERIDINEPYROPHOSPHOKIN-RXN            : 1.0 6-hydroxymethyl-7,8-dihydropterin + 1.0 ATP --> 1.0 6-hydroxymethyl-7,8-
dihydropterin_diphosphate + 1.0 AMP + 1.0 H+  # source: MetaCyc, BRENDA, KEGG (queried 2.7.6.3)
###############################################################################################################################################################################
#THF biosyn
tetrahydrofolate-biosynthesis_2.5.1.15_H2PTEROATESYNTH-RXN                                   : 1.0 4-aminobenzoate + 1.0 6-hydroxymethyl-7,8-dihydropterin_diphosphate --> 1.0 7,8-
dihydropteroate + 1.0 diphosphate  # source: MetaCyc, BRENDA, KEGG (queried 2.5.1.15)
tetrahydrofolate-biosynthesis_6.3.2.17_DIHYDROFOLATESYNTH-RXN                                : 1.0 L-glutamate + 1.0 ATP + 1.0 7,8-dihydropteroate --> 1.0 ADP + 1.0 H+ + 1.0
phosphate + 1.0 7,8-dihydrofolate_monoglutamate  # source: MetaCyc, BRENDA, KEGG (queried 6.3.2.17) // other EC numbers for this reaction: 6.3.2.12
tetrahydrofolate-biosyn/3_manual_1.5.1.3_HD-1.5.1.3                                          : 1.0 NADP+ + 1.0 tetrahydropteroyl_mono-L-glutamate <-- 1.0 H+ + 1.0 NADPH + 1.0 7,8-
dihydrofolate_monoglutamate
#primary triglutamyl in Clostridium acidi-urici (Curthoys et al. 1972)
tetrahydrofolate-biosyn/4_manual_6.3.2.17_HD-6.3.2.17                                        : 1.0 tetrahydropteroyl_mono-L-glutamate + 2.0 L-glutamate + 2.0 ATP --> 1.0
tetrahydropteroyl_tri-L-glutamate + 2.0 ADP + 2.0 phosphate
###############################################################################################################################################################################
#folate transformations
folate-transformations/1_3.5.4.9_2.1.2.10_RXN-6321                                           : 1.0 5,10-methenyltetrahydrofolate + 1.0 H2O --> 1.0 H+ + 1.0 |N5-Formyl-THF-Glu-N|  #
source: MetaCyc, BRENDA, KEGG (queried 3.5.4.9, 2.1.2.10)
folate-transformations/2_6.3.3.2_5-FORMYL-THF-CYCLO-LIGASE-RXN                               : 1.0 ATP + 1.0 |N5-Formyl-THF-Glu-N| --> 1.0 ADP + 1.0 phosphate + 1.0 5,10-
methenyltetrahydrofolate  # source: MetaCyc, BRENDA, KEGG (queried 6.3.3.2)
###############################################################################################################################################################################
#methylerythr path
methylerythritol-phosphate-pathway/1_2.2.1.7_DXS-RXN                                         : 1.0 pyruvate + 1.0 H+ + 1.0 D-glyceraldehyde_3-phosphate --> 1.0 CO2 + 1.0 1-deoxy-D-
xylulose_5-phosphate  # source: MetaCyc, BRENDA, KEGG (queried 2.2.1.7)
methylerythritol-phosphate-pathway/2_1.1.1.267_DXPREDISOM-RXN                                : 1.0 2-C-methyl-D-erythritol_4-phosphate + 1.0 NADP+ <=> 1.0 NADPH + 1.0 1-deoxy-D-
xylulose_5-phosphate + 1.0 H+  # source: MetaCyc, BRENDA, KEGG (queried 1.1.1.267)
methylerythritol-phosphate-pathway/3_2.7.7.60_2.7.7.60-RXN                                   : 1.0 H+ + 1.0 2-C-methyl-D-erythritol_4-phosphate + 1.0 CTP --> 1.0 diphosphate + 1.0
4-(cytidine_5'-diphospho)-2-C-methyl-D-erythritol  # source: MetaCyc, BRENDA, KEGG (queried 2.7.7.60)
methylerythritol-phosphate-pathway/4_2.7.1.148_2.7.1.148-RXN                                 : 1.0 ATP + 1.0 4-(cytidine_5'-diphospho)-2-C-methyl-D-erythritol --> 1.0 ADP + 1.0 2-
phospho-4-(cytidine_5'-diphospho)-2-C-methyl-D-erythritol + 1.0 H+  # source: MetaCyc, BRENDA, KEGG (queried 2.7.1.148)
methylerythritol-phosphate-pathway/5_4.6.1.12_RXN0-302                                       : 1.0 2-phospho-4-(cytidine_5'-diphospho)-2-C-methyl-D-erythritol --> 1.0 2-C-methyl-D-
erythritol-2,4-cyclodiphosphate + 1.0 CMP  # source: MetaCyc, BRENDA, KEGG (queried 4.6.1.12)
methylerythritol-phosphate-pathway/6_1.17.7.1_RXN0-882                                       : 2.0 |Oxidized-ferredoxins| + 1.0 H2O + 1.0 1-hydroxy-2-methyl-2-(E)-butenyl_4-
diphosphate <-- 2.0 |Reduced-ferredoxins| + 1.0 H+ + 1.0 2-C-methyl-D-erythritol-2,4-cyclodiphosphate  # source: MetaCyc, KEGG (queried 1.17.7.1)
methylerythritol-phosphate-pathway/7a_1.17.7.4_ISPH2-RXN                                     : 1.0 isopentenyl_diphosphate + 2.0 |Oxidized-ferredoxins| + 1.0 H2O <-- 2.0 H+ + 1.0
1-hydroxy-2-methyl-2-(E)-butenyl_4-diphosphate + 2.0 |Reduced-ferredoxins|  # source: MetaCyc, KEGG (queried 1.17.7.4) // other EC numbers for this reaction: 1.17.7.c
methylerythritol-phosphate-pathway/7b_1.17.7.4_RXN0-884                                      : 2.0 |Oxidized-ferredoxins| + 1.0 H2O + 1.0 dimethylallyl_diphosphate <-- 1.0 1-
hydroxy-2-methyl-2-(E)-butenyl_4-diphosphate + 2.0 |Reduced-ferredoxins| + 2.0 H+  # source: MetaCyc, KEGG (queried 1.17.7.4) // other EC numbers for this reaction: 1.17.7.c
###############################################################################################################################################################################
#farnesyl PP biosyn
farnesyl-PP-biosyn/1_2.5.1.1_GPPSYN-RXN                                                      : 1.0 dimethylallyl_diphosphate + 1.0 isopentenyl_diphosphate <=> 1.0
geranyl_diphosphate + 1.0 diphosphate  # source: MetaCyc, BRENDA, KEGG (queried 2.5.1.1)
farnesyl-PP-biosyn/2a_2.5.1.10_FPPSYN-RXN                                                    : 1.0 geranyl_diphosphate + 1.0 isopentenyl_diphosphate <=> 1.0 (2E,6E)-
farnesyl_diphosphate + 1.0 diphosphate  # source: MetaCyc, BRENDA, KEGG (queried 2.5.1.10)
farnesyl-PP-biosyn/2b_2.5.1.10_BS421953                                                      : 1.0 dimethylallyl_diphosphate + 1.0 geranyl_diphosphate <=> 1.0 diphosphate + 1.0
(2E,6E)-farnesyl_diphosphate  # source: BRENDA (queried 2.5.1.10)
###############################################################################################################################################################################
#undecaprenyl PP biosyn
undecaprenyl-PP-biosyn_2.5.1.31_RXN-8999                                                     : 1.0 (2E,6E)-farnesyl_diphosphate + 8.0 isopentenyl_diphosphate --> 8.0 diphosphate +
1.0 di-trans,octa-cis-undecaprenyl_diphosphate  # source: MetaCyc, BRENDA, KEGG (queried 2.5.1.31)
###############################################################################################################################################################################
```

```
#4-amino-2-methyl-5-diphosphomethylpyrimidine biosynthesis
4-amino-2-methyl-5-diphosphomethylpyrimidine-biosyn/1_4.1.99.17_PYRIMSYN1-RXN               : 1.0 S-adenosyl-L-methionine + 1.0 5-amino-1-(5-phospho-beta-D-ribosyl)imidazole -->
1.0 4-amino-2-methyl-5-phosphomethylpyrimidine + 3.0 H+ + 1.0 L-methionine + 1.0 carbon_monoxide + 1.0 formate + 1.0 5'-deoxyadenosine  # source: MetaCyc, BRENDA, KEGG (queried
4.1.99.17)
4-amino-2-methyl-5-diphosphomethylpyrimidine-biosyn/2_2.7.4.7_PYRIMSYN3-RXN                 : 1.0 4-amino-2-methyl-5-phosphomethylpyrimidine + 1.0 ATP --> 1.0 ADP + 1.0 4-amino-2-
methyl-5-diphosphomethylpyrimidine  # source: MetaCyc, BRENDA, KEGG (queried 2.7.4.7)
############################################################################################################################################################################
#5'-deoxyadenosine degradation
5'-deoxyadenosine-degr_3.2.2.16_3.2.2.1_3.2.2.9_RXN0-6550                                   : 1.0 H2O + 1.0 5'-deoxyadenosine --> 1.0 adenine + 1.0 5-deoxy-D-ribose  # source:
MetaCyc, BRENDA (queried 3.2.2.16, 3.2.2.1, 3.2.2.9)
############################################################################################################################################################################
#molybdenum cofactor biosynthesis
molybdenum-cofactor-biosynthesis/1_2.8.1.7_RXN0-308                                         : 1.0 |Cysteine-Desulfurase-L-cysteine| + 1.0 L-cysteine --> 1.0 L-alanine + 1.0 |L-
Cysteine-Desulfurase-persulfide|  # source: MetaCyc (queried 2.8.1.7)
molybdenum-cofactor-biosynthesis/2_2.7.7.80_RXN-11361                                       : 1.0 ATP + 1.0 |MPT-Synthases| + 1.0 H+ --> 1.0 diphosphate + 1.0 |Carboxyadenylated-
MPT-synthases|  # source: MetaCyc (queried 2.7.7.80)
molybdenum-cofactor-biosynthesis/3_mod_2.8.1.11_RXN-12473                                   : 2.0 |Reduced-ferredoxins| + 1.0 H+ + 1.0 |Carboxyadenylated-MPT-synthases| + 1.0 |L-
Cysteine-Desulfurase-persulfide| --> 1.0 |Cysteine-Desulfurase-L-cysteine| + 1.0 AMP + 1.0 |Thiocarboxylated-MPT-synthases| + 2.0 |Oxidized-ferredoxins|  # source: MetaCyc
(queried 2.8.1.11)
molybdenum-cofactor-biosynthesis/4_4.1.99.18_RXN-8340                                       : 1.0 GTP --> 1.0 cyclic_pyranopterin_phosphate + 1.0 diphosphate  # source: MetaCyc,
BRENDA, KEGG (queried 4.1.99.18)
molybdenum-cofactor-biosynthesis/5_2.8.1.12_RXN-8342                                        : 1.0 cyclic_pyranopterin_phosphate + 2.0 |Thiocarboxylated-MPT-synthases| + 1.0 H2O
--> 1.0 molybdopterin + 2.0 |MPT-Synthases| + 4.0 H+  # source: MetaCyc (queried 2.8.1.12)
molybdenum-cofactor-biosynthesis/6_2.7.7.75_RXN-8344                                        : 1.0 ATP + 1.0 H+ + 1.0 molybdopterin --> 1.0 molybdopterin_adenine_dinucleotide + 1.0
diphosphate  # source: MetaCyc, BRENDA, KEGG (queried 2.7.7.75)
molybdenum-cofactor-biosynthesis/7_2.10.1.1_RXN-8348                                        : 1.0 molybdopterin_adenine_dinucleotide + 1.0 molybdate + 1.0 H+ --> 1.0 MoO2-
molybdopterin_cofactor + 1.0 AMP + 1.0 H2O  # source: MetaCyc, BRENDA, KEGG (queried 2.10.1.1)
############################################################################################################################################################################
#thiazole biosynthesis
thiazole-biosynthesis/1_2.8.1.7_RXN-9787                                                    : 1.0 |Sulfur-Carrier-Proteins-ThiI| + 1.0 L-cysteine <=> 1.0 L-alanine + 1.0 |
Sulfurylated-ThiI|  # source: MetaCyc (queried 2.8.1.7)
thiazole-biosynthesis/2_2.7.7.73_RXN-9789                                                   : 1.0 H+ + 1.0 |Thi-S| + 1.0 ATP --> 1.0 |Adenylated-ThiS-Proteins| + 1.0 diphosphate
# source: MetaCyc, BRENDA (queried 2.7.7.73)
thiazole-biosynthesis/3_2.8.1.-_RXN-9788                                                    : 2.0 |Reduced-ferredoxins| + 1.0 |Adenylated-ThiS-Proteins| + 1.0 |Sulfurylated-ThiI|
--> 1.0 |Thiocarboxyadenylated-ThiS-Proteins| + 1.0 |Sulfur-Carrier-Proteins-ThiI| + 2.0 |Oxidized-ferredoxins| + 1.0 AMP  # source: MetaCyc (queried 2.8.1.-) // other EC numbers
for this reaction: 2.8.1
thiazole-biosynthesis/p4_4.1.99.19_RXN-11319                                                : 1.0 S-adenosyl-L-methionine + 1.0 NADPH + 1.0 L-tyrosine --> 1.0 5'-deoxyadenosine +
1.0 H+ + 1.0 NADP+ + 1.0 L-methionine + 1.0 2-iminoacetate + 1.0 4-methylphenol  # source: MetaCyc, BRENDA (queried 4.1.99.19)
thiazole-biosynthesis/4_2.8.1.10_THIAZOLSYN2-RXN                                            : 1.0 1-deoxy-D-xylulose_5-phosphate + 1.0 |Thiocarboxyadenylated-ThiS-Proteins| + 1.0
2-iminoacetate --> 2.0 H2O + 1.0 |Thi-S| + 1.0 2-[(2R,5Z)-2-carboxy-4-methylthiazol-5(2H)-ylidene]ethyl_phosphate  # source: MetaCyc (queried 2.8.1.10)
############################################################################################################################################################################
#thiamin diphosphate biosynthesis III (Staphylococcus)
thiamin-diphosphate-biosyn-III/1_2.5.1.3_RXN-12611                                          : 2.0 H+ + 1.0 2-[(2R,5Z)-2-carboxy-4-methylthiazol-5(2H)-ylidene]ethyl_phosphate + 1.0
4-amino-2-methyl-5-diphosphomethylpyrimidine --> 1.0 thiamine_phosphate + 1.0 diphosphate + 1.0 CO2  # source: MetaCyc, BRENDA (queried 2.5.1.3)
thiamin-diphosphate-biosyn-III/2_3.1.3.2_RXNQT-4191                                         : 1.0 thiamine_phosphate + 1.0 H2O --> 1.0 phosphate + 1.0 thiamine  # source: MetaCyc,
BRENDA, KEGG (queried 3.1.3.2) // other EC numbers for this reaction: 3.1.3, 3.1.3.w
thiamin-diphosphate-biosyn-III/3_2.7.6.2_THIAMIN-PYROPHOSPHOKINASE-RXN                      : 1.0 thiamine + 1.0 ATP --> 1.0 H+ + 1.0 AMP + 1.0 thiamine_diphosphate  # source:
MetaCyc, BRENDA, KEGG (queried 2.7.6.2)
############################################################################################################################################################################
#thiamin salvage
thiamin-salvage/1_3.5.99.2_THIAMINASE-RXN                                                   : 1.0 thiamine + 1.0 H2O --> 1.0 H+ + 1.0 4-amino-2-methyl-5-pyrimidinemethanol + 1.0
5-(2-hydroxyethyl)-4-methylthiazole  # source: MetaCyc, BRENDA, KEGG (queried 3.5.99.2)
thiamin-salvage/2_2.7.1.50_THIAZOLSYN3-RXN                                                  : 1.0 ATP + 1.0 5-(2-hydroxyethyl)-4-methylthiazole --> 1.0 H+ + 1.0 ADP + 1.0 4-
methyl-5-(2-phosphonooxyethyl)thiazole  # source: MetaCyc, BRENDA, KEGG (queried 2.7.1.50)
thiamin-salvage/p3_2.7.1.49_OHMETPYRKIN-RXN                                                 : 1.0 ATP + 1.0 4-amino-2-methyl-5-pyrimidinemethanol --> 1.0 ADP + 1.0 4-amino-2-
methyl-5-phosphomethylpyrimidine + 1.0 H+  # source: MetaCyc, BRENDA, KEGG (queried 2.7.1.49)
thiamin-salvage/4_2.5.1.3_THI-P-SYN-RXN                                                     : 1.0 4-methyl-5-(2-phosphonooxyethyl)thiazole + 1.0 4-amino-2-methyl-5-
diphosphomethylpyrimidine + 1.0 H+ --> 1.0 diphosphate + 1.0 thiamine_phosphate  # source: MetaCyc, BRENDA, KEGG (queried 2.5.1.3)
############################################################################################################################################################################
#tetrapyrrole biosynthesis I
tetrapyrrole-biosynthesis/1_1.2.1.70_GLUTRNAREDUCT-RXN                                      : 1.0 |GLT-tRNAs| + 1.0 NADP+ + 1.0 (S)-4-amino-5-oxopentanoate <-- 1.0 |Charged-GLT-
tRNAs| + 1.0 NADPH  # source: MetaCyc (queried 1.2.1.70)
tetrapyrrole-biosynthesis/2_5.4.3.8_GSAAMINOTRANS-RXN                                       : 1.0 (S)-4-amino-5-oxopentanoate --> 1.0 5-aminolevulinate  # source: MetaCyc, BRENDA,
KEGG (queried 5.4.3.8)
```

```
tetrapyrrole-biosynthesis/3_4.2.1.24_PORPHOBILSYNTH-RXN                                        : 2.0 5-aminolevulinate --> 1.0 H+ + 1.0 porphobilinogen + 2.0 H2O  # source: MetaCyc, BRENDA, KEGG (queried 4.2.1.24)
tetrapyrrole-biosynthesis/4_2.5.1.61_OHMETHYLBILANESYN-RXN                                     : 4.0 porphobilinogen + 1.0 H2O --> 4.0 ammonium + 1.0 preuroporphyrinogen  # source: MetaCyc, BRENDA, KEGG (queried 2.5.1.61)
tetrapyrrole-biosynthesis/5_4.2.1.75_UROGENIIISYN-RXN                                          : 1.0 preuroporphyrinogen --> 1.0 H2O + 1.0 uroporphyrinogen-III  # source: MetaCyc, BRENDA, KEGG (queried 4.2.1.75)
##################################################################################################################################################################################
#cobyrinate biosyn I
cobyrinate-biosyn-I/1_2.1.1.107_UROPORIIIMETHYLTRANSA-RXN              : 1.0 uroporphyrinogen-III + 1.0 S-adenosyl-L-methionine --> 1.0 precorrin-1 + 1.0 S-adenosyl-L-homocysteine + 1.0 H+  # source: MetaCyc, BRENDA, KEGG (queried 2.1.1.107)
cobyrinate-biosyn-I/2_2.1.1.107_RXN-8675                               : 1.0 S-adenosyl-L-methionine + 1.0 precorrin-1 --> 1.0 S-adenosyl-L-homocysteine + 1.0 precorrin-2  # source: MetaCyc, BRENDA, KEGG (queried 2.1.1.107)
cobyrinate-biosyn-I/3_1.3.1.76_DIMETHUROPORDEHYDROG-RXN                : 1.0 NAD+ + 1.0 precorrin-2 <=> 2.0 H+ + 1.0 NADH + 1.0 sirohydrochlorin  # source: MetaCyc, BRENDA, KEGG (queried 1.3.1.76)
cobyrinate-biosyn-I/4_4.99.1.3_4.99.1.3-RXN                            : 1.0 sirohydrochlorin + 1.0 Co2+ --> 1.0 cobalt-sirohydrochlorin + 2.0 H+  # source: MetaCyc, BRENDA, KEGG (queried 4.99.1.3)
cobyrinate-biosyn-I/5_2.1.1.151_2.1.1.151-RXN                          : 1.0 cobalt-sirohydrochlorin + 1.0 S-adenosyl-L-methionine --> 1.0 cobalt-factor_III + 1.0 S-adenosyl-L-homocysteine + 1.0 H+  # source: MetaCyc, BRENDA, KEGG (queried 2.1.1.151)
#Electron donor may be thioredoxin (Moore et al. 2013)
cobyrinate-biosyn-I/6_mod_2.1.1.272_RXN-14314                          : 1.0 |Red-Thioredoxin| + 1.0 cobalt-factor_III + 1.0 S-adenosyl-L-methionine --> 1.0 cobalt-precorrin-4 + 1.0 |Ox-Thioredoxin| + 1.0 S-adenosyl-L-homocysteine  # source: MetaCyc, BRENDA (queried 2.1.1.272)
cobyrinate-biosyn-I/7_2.1.1.271_RXN-8762                               : 1.0 cobalt-precorrin-4 + 1.0 S-adenosyl-L-methionine --> 1.0 S-adenosyl-L-homocysteine + 1.0 cobalt-precorrin-5A + 1.0 H+  # source: MetaCyc, BRENDA, KEGG (queried 2.1.1.271)
cobyrinate-biosyn-I/8_3.7.1.12_RXN-8763                                : 1.0 cobalt-precorrin-5A + 1.0 H2O --> 1.0 acetaldehyde + 1.0 cobalt-precorrin-5B + 1.0 H+  # source: MetaCyc, BRENDA, KEGG (queried 3.7.1.12)
cobyrinate-biosyn-I/9_2.1.1.195_RXN-8764                               : 1.0 cobalt-precorrin-5B + 1.0 S-adenosyl-L-methionine --> 1.0 S-adenosyl-L-homocysteine + 1.0 cobalt-precorrin-6A  # source: MetaCyc, BRENDA, KEGG (queried 2.1.1.195)
cobyrinate-biosyn-I/10_1.3.1.106_RXN-8765                              : 1.0 cobalt-precorrin-6B + 1.0 NAD+ <-- 1.0 cobalt-precorrin-6A + 1.0 NADH  # source: MetaCyc, BRENDA, KEGG (queried 1.3.1.106)
cobyrinate-biosyn-I/11_2.1.1.196_RXN-8766                              : 1.0 cobalt-precorrin-6B + 1.0 S-adenosyl-L-methionine + 1.0 H+ --> 1.0 cobalt-precorrin-7 + 1.0 CO2 + 1.0 S-adenosyl-L-homocysteine  # source: MetaCyc, BRENDA, KEGG (queried 2.1.1.196)
cobyrinate-biosyn-I/12_2.1.1.289_RXN-8767                              : 1.0 S-adenosyl-L-methionine + 1.0 cobalt-precorrin-7 --> 1.0 S-adenosyl-L-homocysteine + 1.0 cobalt-precorrin-8  # source: MetaCyc, BRENDA, KEGG (queried 2.1.1.289)
cobyrinate-biosyn-I/13_5.4.99.60_RXN-8768                              : 1.0 cobalt-precorrin-8 --> 1.0 H+ + 1.0 cobyrinate  # source: MetaCyc, BRENDA, KEGG (queried 5.4.99.60)
cobyrinate-biosyn-I/14_6.3.5.11_RXN-14256                              : 1.0 H2O + 1.0 L-glutamine + 1.0 cobyrinate + 1.0 ATP --> 1.0 cob(II)yrinate_c-monoamide + 1.0 ADP + 1.0 H+ + 1.0 L-glutamate + 1.0 phosphate  # source: MetaCyc, BRENDA, KEGG (queried 6.3.5.11)
cobyrinate-biosyn-I/15_6.3.5.11_RXN-14257                              : 1.0 L-glutamine + 1.0 cob(II)yrinate_c-monoamide + 1.0 ATP + 1.0 H2O --> 1.0 L-glutamate + 1.0 cob(II)yrinate_a,c-diamide + 1.0 ADP + 1.0 H+ + 1.0 phosphate  # source: MetaCyc, BRENDA, KEGG (queried 6.3.5.11)
##################################################################################################################################################################################
#5,6-dimethylbenzimidazole biosyn II
#4.1.99.M1: Ferredoxins as a guess
5,6-dimethylbenzimidazole-biosyn-II/1_4.1.99.M1_RXN-17122                                   : 1.0 5-amino-1-(5-phospho-beta-D-ribosyl)imidazole + 1.0 S-adenosyl-L-methionine + 2.0 |Reduced-ferredoxins| --> 1.0 5-hydroxy-benzimidazole + 1.0 ammonium + 1.0 formate + 1.0 5'-deoxyadenosine + 1.0 L-methionine + 1.0 phosphate + 2.0 |Oxidized-ferredoxins|
5,6-dimethylbenzimidazole-biosyn-II/2_2.1.1.M9_RXN-17123                                    : 1.0 5-hydroxy-benzimidazole + 1.0 S-adenosyl-L-methionine --> 1.0 5-methoxy-benzimidazole + 1.0 S-adenosyl-L-homocysteine + 1.0 H+
5,6-dimethylbenzimidazole-biosyn-II/3_2.1.1.M10_RXN-17124                                   : 1.0 5-methoxy-benzimidazole + 1.0 S-adenosyl-L-methionine --> 1.0 5-methoxy-6-methylbenzimidazole + 1.0 S-adenosyl-L-homocysteine + 1.0 H+
#mechanism completely unclear
5,6-dimethylbenzimidazole-biosyn-II/4_unknown_RXN-17125                                     : 1.0 5-methoxy-6-methylbenzimidazole --> 5,6-dimethylbenzimidazole
##################################################################################################################################################################################
#adenosylcobalamin biosyn I
#1.16.8.1: NADH as a guess
adenosylcobalamin-biosynthesis-I/1_mod_1.16.8.1_R343-RXN                                    : 2.0 H+ + 1.0 NAD+ + 2.0 cob(I)yrinate_a,c-diamide <-- 2.0 cob(II)yrinate_a,c-diamide + 1.0 NADH  # source: MetaCyc, BRENDA, KEGG (queried 1.16.8.1)
adenosylcobalamin-biosynthesis-I/2_2.5.1.17_R344-RXN                                        : 1.0 cob(I)yrinate_a,c-diamide + 1.0 ATP --> 1.0 adenosyl-cobyrinate_a,c-diamide + 1.0 PPPi  # source: MetaCyc, BRENDA, KEGG (queried 2.5.1.17)
adenosylcobalamin-biosynthesis-I/3_6.3.5.10_R345-RXN                                        : 4.0 L-glutamine + 1.0 adenosyl-cobyrinate_a,c-diamide + 4.0 ATP + 4.0 H2O --> 4.0 ADP + 4.0 H+ + 4.0 L-glutamate + 4.0 phosphate + 1.0 adenosylcobyrate  # source: MetaCyc, BRENDA, KEGG (queried 6.3.5.10)
adenosylcobalamin-biosynthesis-I/pp4_2.7.1.177_RXN-8626                                     : 1.0 L-threonine + 1.0 ATP --> 1.0 L-threonine_3-O-phosphate + 1.0 ADP + 1.0 H+  # source: MetaCyc, BRENDA, KEGG (queried 2.7.1.177)
adenosylcobalamin-biosynthesis-I/p4_4.1.1.81_4.1.1.81-RXN                                   : 1.0 L-threonine_3-O-phosphate + 1.0 H+ --> 1.0 CO2 + 1.0 (R)-1-amino-2-propanol_O-2-phosphate  # source: MetaCyc, BRENDA, KEGG (queried 4.1.1.81)
adenosylcobalamin-biosynthesis-I/4_6.3.1.10_RXN-6261                                        : 1.0 ATP + 1.0 (R)-1-amino-2-propanol_O-2-phosphate + 1.0 adenosylcobyrate --> 1.0 phosphate + 1.0 ADP + 1.0 adenosyl-cobinamide_phosphate + 1.0 H+  # source: MetaCyc, BRENDA, KEGG (queried 6.3.1.10)
```

```
adenosylcobalamin-biosynthesis-I/5_2.7.7.62_COBINPGUANYLYLTRANS-RXN                    : 1.0 H+ + 1.0 GTP + 1.0 adenosyl-cobinamide_phosphate <=> 1.0 diphosphate + 1.0
adenosylcobinamide-GDP  # source: MetaCyc, BRENDA, KEGG (queried 2.7.7.62)
adenosylcobalamin-biosynthesis-I/pp6_6.3.4.21_NICOTINATEPRIBOSYLTRANS-RXN              : 1.0 ATP + 1.0 H2O + 1.0 5-phospho-alpha-D-ribose_1-diphosphate + 1.0 nicotinate --> 1.0
beta-nicotinate_D-ribonucleotide + 1.0 phosphate + 1.0 ADP + 1.0 diphosphate  # source: MetaCyc, BRENDA, KEGG (queried 6.3.4.21)
adenosylcobalamin-biosynthesis-I/p6_2.4.2.21_DMBPPRIBOSYLTRANS-RXN                     : 1.0 beta-nicotinate_D-ribonucleotide + 1.0 5,6-dimethylbenzimidazole --> 1.0 nicotinate
+ 1.0 alpha-ribazole_5'-phosphate + 1.0 H+  # source: MetaCyc, BRENDA, KEGG (queried 2.4.2.21)
adenosylcobalamin-biosynthesis-I/6a_2.7.8.26_COBALAMIN5PSYN-RXN                        : 1.0 alpha-ribazole_5'-phosphate + 1.0 adenosylcobinamide-GDP --> 1.0
adenosylcobalamin_5'-phosphate + 1.0 H+ + 1.0 GMP  # source: MetaCyc, BRENDA (queried 2.7.8.26)
adenosylcobalamin-biosynthesis-I/7a_3.1.3.73_RXN-8770                                  : 1.0 adenosylcobalamin_5'-phosphate + 1.0 H2O --> 1.0 adenosylcobalamin + 1.0 phosphate
# source: MetaCyc, BRENDA (queried 3.1.3.73)
adenosylcobalamin-biosynthesis-I/6b_3.1.3.73_RIBAZOLEPHOSPHAT-RXN                      : 1.0 alpha-ribazole_5'-phosphate + 1.0 H2O --> 1.0 alpha-ribazole + 1.0 phosphate  #
source: MetaCyc, BRENDA, KEGG (queried 3.1.3.73)
adenosylcobalamin-biosynthesis-I/7b_2.7.8.26_COBALAMINSYN-RXN                          : 1.0 alpha-ribazole + 1.0 adenosylcobinamide-GDP <=> 1.0 adenosylcobalamin + 1.0 GMP +
1.0 H+  # source: MetaCyc, BRENDA, KEGG (queried 2.7.8.26)
#####################################################################################################################################################################################
#protoporphyrin biosynthesis
protoporphyrin-biosyn/1_4.1.1.37_UROGENDECARBOX-RXN                                    : 1.0 uroporphyrinogen-III + 4.0 H+ --> 4.0 CO2 + 1.0 coproporphyrinogen_III  # source:
MetaCyc, BRENDA, KEGG (queried 4.1.1.37)
protoporphyrin-biosyn/2_modnames_1.3.99.22_HEMN-RXN                                    : 1.0 coproporphyrinogen_III + 2.0 S-adenosyl-L-methionine --> 1.0 protoporphyrin_IX + 2.0
L-methionine + 2.0 5'-deoxyadenosine + 2.0 CO2  # source: MetaCyc, BRENDA, KEGG (queried 1.3.99.22)
protoporphyrin-biosyn/3_6.6.1.1_RXN1F-20                                               : 1.0 ATP + 1.0 H2O + 1.0 protoporphyrin_IX + 1.0 Mg2+ --> 1.0 phosphate + 1.0 Mg-
protoporphyrin + 1.0 ADP + 3.0 H+  # source: MetaCyc, BRENDA, KEGG (queried 6.6.1.1)
#####################################################################################################################################################################################
#biotin carboxyl carrier protein assembly
biotin-carboxyl-carrier-protein-assembly/1_6.3.4.15_BIOTINLIG-RXN                      : 1.0 biotin + 1.0 ATP + 1.0 |BCCP-monomers| --> 1.0 H+ + 1.0 diphosphate + 1.0 AMP + 1.0
|BCCP-biotin-monomers|  # source: MetaCyc (queried 6.3.4.15)
biotin-carboxyl-carrier-protein-assembly/2_spontaneous_RXN-7101                        : 2.0 |BCCP-biotin-monomers| --> 1.0 |BCCP-dimers|  # source: MetaCyc
biotin-carboxyl-carrier-protein-assembly/3_6.3.4.14_BIOTIN-CARBOXYL-RXN                : 1.0 ATP + 1.0 |BCCP-dimers| + 1.0 hydrogen_carbonate --> 1.0 H+ + 1.0 phosphate + 1.0
ADP + 1.0 |Carboxybiotin-BCCP|  # source: MetaCyc (queried 6.3.4.14)
#####################################################################################################################################################################################
#pyridoxal
PLP-biosyn_2.7.1.49_PNKIN-RXN                                                          : 1.0 ATP + 1.0 pyridoxine --> 1.0 ADP + 1.0 pyridoxine_5'-phosphate + 1.0 H+  # source:
MetaCyc, BRENDA, KEGG (queried 2.7.1.49) // other EC numbers for this reaction: 2.7.1.35
#####################################################################################################################################################################################
#L-threonylcarbamoyladenylate
L-threonylcarbamoyladenylate_2.7.7.87_RXN-14569                                        : 1.0 hydrogen_carbonate + 1.0 L-threonine + 1.0 ATP <=> 1.0 diphosphate + 1.0 L-
threonylcarbamoyladenylate + 1.0 H2O  # source: MetaCyc, BRENDA, KEGG (queried 2.7.7.87)
#####################################################################################################################################################################################
#NXP de novo biosyn
diphosphate_3.6.1.1_INORGPYROPHOSPHAT-RXN                                              : 1.0 H2O + 1.0 diphosphate --> 2.0 phosphate + 1.0 H+  # source: MetaCyc, BRENDA, KEGG
(queried 3.6.1.1) // other EC numbers for this reaction: 2.7.99.1
triphosphate_3.6.1.1_modb_BS383385                                                     : 2.0 H2O + 1.0 PPPi --> 3.0 phosphate + 1.0 H+  # source: BRENDA (queried 3.6.1.1) //
balanced // contains implicit reactant(s): H+, H2O

diphosphate_2.7.11.32_RXN-13757                                                        : 1.0 ADP + 1.0 |pyruvate-phosphate-dikinase| --> 1.0 H+ + 1.0 AMP + 1.0 |pyruvate-
phosphate-dikinase-phosphate|  # source: MetaCyc, BRENDA (queried 2.7.11.32)
diphosphate_2.7.4.27_RXN-13756                                                         : 1.0 |pyruvate-phosphate-dikinase-phosphate| + 1.0 H+ + 1.0 phosphate <=> 1.0 diphosphate
+ 1.0 |pyruvate-phosphate-dikinase|  # source: MetaCyc, BRENDA (queried 2.7.4.27)


#AMP
adenosine-ribonucleotides-de-novo-biosyn/1_6.3.4.4_ADENYLOSUCCINATE-SYNTHASE-RXN       : 1.0 L-aspartate + 1.0 IMP + 1.0 GTP --> 1.0 GDP + 1.0 adenylo-succinate + 2.0 H+ + 1.0
phosphate  # source: MetaCyc, BRENDA, KEGG (queried 6.3.4.4)
adenosine-ribonucleotides-de-novo-biosyn/2_4.3.2.2_AMPSYN-RXN                          : 1.0 adenylo-succinate <=> 1.0 fumarate + 1.0 AMP  # source: MetaCyc, BRENDA, KEGG
(queried 4.3.2.2)

#GMP
guanosine-ribonucleotides-de-novo-biosyn/1_1.1.1.205_IMP-DEHYDROG-RXN                  : 1.0 H2O + 1.0 NAD+ + 1.0 IMP <=> 1.0 NADH + 1.0 XMP + 1.0 H+  # source: MetaCyc, BRENDA,
KEGG (queried 1.1.1.205)
guanosine-ribonucleotides-de-novo-biosyn/2_6.3.5.2_GMP-SYN-GLUT-RXN                    : 1.0 L-glutamine + 1.0 ATP + 1.0 XMP + 1.0 H2O --> 1.0 AMP + 1.0 diphosphate + 2.0 H+ +
1.0 GMP + 1.0 L-glutamate  # source: MetaCyc, BRENDA, KEGG (queried 6.3.5.2)

#UMP
UMP-biosyn/1_2.1.3.2_ASPCARBTRANS-RXN                                                  : 1.0 carbamoyl_phosphate + 1.0 L-aspartate <=> 1.0 N-carbamoyl-L-aspartate + 1.0
```

phosphate + 1.0 H+ # source: MetaCyc, BRENDA, KEGG (queried 2.1.3.2)
UMP-biosyn/2_moddir_3.5.2.3_DIHYDROOROT-RXN : 1.0 H2O + 1.0 (S)-dihydroorotate <=> 1.0 N-carbamoyl-L-aspartate + 1.0 H+ # source:
MetaCyc, BRENDA, KEGG (queried 3.5.2.3)
UMP-biosyn/3_1.3.1.14_OROTATE-REDUCTASE-NADH-RXN : 1.0 NAD+ + 1.0 (S)-dihydroorotate <=> 1.0 orotate + 1.0 H+ + 1.0 NADH # source:
MetaCyc, BRENDA, KEGG (queried 1.3.1.14)
UMP-biosyn/4_2.4.2.10_OROPRIBTRANS-RXN : 1.0 diphosphate + 1.0 orotidine_5'-phosphate <=> 1.0 orotate + 1.0 5-phospho-alpha-D-
ribose_1-diphosphate # source: MetaCyc, BRENDA, KEGG (queried 2.4.2.10)
UMP-biosyn/5_4.1.1.23_OROTPDECARB-RXN : 1.0 orotidine_5'-phosphate + 1.0 H+ --> 1.0 CO2 + 1.0 UMP # source: MetaCyc, BRENDA,
KEGG (queried 4.1.1.23)

#CTP
CTP-biosy2_6.3.4.2_CTPSYN-RXN : 1.0 L-glutamine + 1.0 H2O + 1.0 UTP + 1.0 ATP --> 1.0 CTP + 1.0 ADP + 2.0 H+ + 1.0
phosphate + 1.0 L-glutamate # source: MetaCyc, BRENDA, KEGG (queried 6.3.4.2)

#UTP
UTP-biosyn_3.5.4.5_RXN-14118 : 1.0 H2O + 1.0 H+ + 1.0 CTP --> 1.0 ammonium + 1.0 UTP # source: MetaCyc, BRENDA, KEGG
(queried 3.5.4.5) // other EC numbers for this reaction: 3.5.4.13

#XDP -> dXDP
deoxyribonucleotides-biosyn_1.17.4.1_ADPREDUCT-RXN : 1.0 dADP + 1.0 |Ox-Thioredoxin| + 1.0 H2O <=> 1.0 ADP + 1.0 |Red-Thioredoxin| # source:
MetaCyc, KEGG (queried 1.17.4.1)
deoxyribonucleotides-biosyn_1.17.4.1_GDPREDUCT-RXN : 1.0 |Ox-Thioredoxin| + 1.0 H2O + 1.0 dGDP <-- 1.0 GDP + 1.0 |Red-Thioredoxin| # source:
MetaCyc, KEGG (queried 1.17.4.1)
deoxyribonucleotides-biosyn_1.17.4.1_CDPREDUCT-RXN : 1.0 H2O + 1.0 |Ox-Thioredoxin| + 1.0 dCDP <-- 1.0 |Red-Thioredoxin| + 1.0 CDP # source:
MetaCyc, KEGG (queried 1.17.4.1)
deoxyribonucleotides-biosyn_1.17.4.1_UDPREDUCT-RXN : 1.0 |Ox-Thioredoxin| + 1.0 H2O + 1.0 dUDP <-- 1.0 |Red-Thioredoxin| + 1.0 UDP # source:
MetaCyc, KEGG (queried 1.17.4.1)

#|Oxidized-flavodoxins| --> |Ox-Thioredoxin| because of BRENDA 1.17.4.2 and Clostridium sp.
deoxyribonucleotides-biosyn_mod_1.17.4.2_RXN0-723 : 1.0 |Ox-Thioredoxin| + 1.0 dCTP + 1.0 H2O <-- 1.0 CTP + 1.0 |Red-Thioredoxin| # source:
MetaCyc (queried 1.17.4.2)
deoxyribonucleotides-biosyn_mod_1.17.4.2_RXN0-724 : 1.0 H2O + 1.0 |Ox-Thioredoxin| + 1.0 dUTP <-- 1.0 UTP + 1.0 |Red-Thioredoxin| # source:
MetaCyc (queried 1.17.4.2)
deoxyribonucleotides-biosyn_mod_1.17.4.2_RXN0-745 : 1.0 |Ox-Thioredoxin| + 1.0 dATP + 1.0 H2O <-- 1.0 |Red-Thioredoxin| + 1.0 ATP # source:
MetaCyc (queried 1.17.4.2)
deoxyribonucleotides-biosyn_mod_1.17.4.2_RXN0-746 : 1.0 |Ox-Thioredoxin| + 1.0 H2O + 1.0 dGTP <-- 1.0 GTP + 1.0 |Red-Thioredoxin| # source:
MetaCyc (queried 1.17.4.2)

#dTTP
dTTP-biosyn/1_3.6.1.23_DUTP-PYROP-RXN : 1.0 H2O + 1.0 dUTP --> 1.0 diphosphate + 1.0 dUMP + 1.0 H+ # source: MetaCyc, BRENDA,
KEGG (queried 3.6.1.23) // other EC numbers for this reaction: 3.6.1.19
dTTP-biosyn/2_2.1.1.148_RXN-8850 : 1.0 NADPH + 1.0 5,10-methylene-tetrahydrofolate + 1.0 H+ + 1.0 dUMP --> 1.0
tetrahydropteroyl_tri-L-glutamate + 1.0 dTMP + 1.0 NADP+ # source: MetaCyc (queried 2.1.1.148)
##
##(d)NMP degradation

#RNA-Abbau
#CMP
CMP-salvage/1_3.1.3.5_RXN-14026 : 1.0 H2O + 1.0 CMP --> 1.0 cytidine + 1.0 phosphate # source: MetaCyc, BRENDA, KEGG
(queried 3.1.3.5) // other EC numbers for this reaction: 3.1.3.91
CMP-salvage/2a_2.7.1.48_CYTIKIN-RXN : 1.0 cytidine + 1.0 ATP --> 1.0 ADP + 1.0 H+ + 1.0 CMP # source: MetaCyc, BRENDA, KEGG
(queried 2.7.1.48) // other EC numbers for this reaction: 2.7.1.B24
CMP-salvage/2b_3.5.4.5_CYTIDEAM2-RXN : 1.0 H2O + 1.0 H+ + 1.0 cytidine --> 1.0 ammonium + 1.0 uridine # source: MetaCyc,
BRENDA, KEGG (queried 3.5.4.5)
CMP-salvage/2c_2.4.2.2_BS355365 : 1.0 cytidine + 1.0 phosphate <=> 1.0 cytosine + 1.0 alpha-D-ribose-1-phosphate #
source: BRENDA, KEGG (queried 2.4.2.2) // other EC numbers for this reaction: 2.4.2.3
#UMP
UMP-salvage/1_3.1.3.5_3.1.3.2_RXN-14025 : 1.0 H2O + 1.0 UMP --> 1.0 uridine + 1.0 phosphate # source: MetaCyc, BRENDA, KEGG
(queried 3.1.3.5, 3.1.3.2)
UMP-salvage/2a_2.7.1.48_URIDINEKIN-RXN : 1.0 ATP + 1.0 uridine --> 1.0 H+ + 1.0 ADP + 1.0 UMP # source: MetaCyc, BRENDA, KEGG
(queried 2.7.1.48)
UMP-salvage/2b_2.4.2.2_URPHOS-RXN : 1.0 phosphate + 1.0 uridine <=> 1.0 alpha-D-ribose-1-phosphate + 1.0 uracil # source:
MetaCyc, BRENDA, KEGG (queried 2.4.2.2) // other EC numbers for this reaction: 2.4.2.3
UMP-salvage/3_2.4.2.9_URACIL-PRIBOSYLTRANS-RXN : 1.0 UMP + 1.0 diphosphate <-- 1.0 5-phospho-alpha-D-ribose_1-diphosphate + 1.0 uracil #

source: MetaCyc, BRENDA, KEGG (queried 2.4.2.9)
#AMP
AMP-salvage/1_3.1.3.5_3.1.3.2_AMP-DEPHOSPHORYLATION-RXN : 1.0 H2O + 1.0 AMP --> 1.0 phosphate + 1.0 adenosine # source: MetaCyc, BRENDA, KEGG (queried 3.1.3.5, 3.1.3.2)
AMP-salvage/2_2.4.2.1_ADENPHOSPHOR-RXN : 1.0 adenosine + 1.0 phosphate <=> 1.0 adenine + 1.0 alpha-D-ribose-1-phosphate # source: MetaCyc, BRENDA, KEGG (queried 2.4.2.1) // other EC numbers for this reaction: 2.4.2.44
AMP-salvage/3_2.4.2.8_2.4.2.7_ADENPRIBOSYLTRAN-RXN : 1.0 AMP + 1.0 diphosphate <-- 1.0 adenine + 1.0 5-phospho-alpha-D-ribose_1-diphosphate # source: MetaCyc, BRENDA, KEGG (queried 2.4.2.8, 2.4.2.7)
#GMP
GMP-salvage/1_3.1.3.5_3.1.3.2_RXN-7609 : 1.0 H2O + 1.0 GMP --> 1.0 guanosine + 1.0 phosphate # source: MetaCyc, BRENDA, KEGG (queried 3.1.3.5, 3.1.3.2)
GMP-salvage/2_2.4.2.1_RXN0-5199 : 1.0 guanosine + 1.0 phosphate <=> 1.0 guanine + 1.0 alpha-D-ribose-1-phosphate # source: MetaCyc, BRENDA, KEGG (queried 2.4.2.1) // other EC numbers for this reaction: 2.4.2.15
GMP-salvage/3_3.5.4.3_GUANINE-DEAMINASE-RXN : 1.0 H2O + 1.0 H+ + 1.0 guanine --> 1.0 ammonium + 1.0 xanthine # source: MetaCyc, BRENDA, KEGG (queried 3.5.4.3)
GMP-salvage/4_2.4.2.8_2.4.2.22_XANPRIBOSYLTRAN-RXN : 1.0 XMP + 1.0 diphosphate <-- 1.0 xanthine + 1.0 5-phospho-alpha-D-ribose_1-diphosphate # source: MetaCyc, BRENDA, KEGG (queried 2.4.2.8, 2.4.2.22)
#XMP
XMP-salvage/1_3.1.3.5_XMPXAN-RXN : 1.0 XMP + 1.0 H2O --> 1.0 phosphate + 1.0 xanthosine # source: MetaCyc, BRENDA, KEGG (queried 3.1.3.5)
XMP-salvage/2_2.4.2.1_XANTHOSINEPHOSPHORY-RXN : 1.0 phosphate + 1.0 xanthosine --> 1.0 alpha-D-ribose-1-phosphate + 1.0 xanthine # source: MetaCyc, BRENDA, KEGG (queried 2.4.2.1) // other EC numbers for this reaction: 2.4.2
#IMP
IMP-salvage_3.1.3.5_RXN-7607 : 1.0 IMP + 1.0 H2O --> 1.0 inosine + 1.0 phosphate # source: MetaCyc, BRENDA, KEGG (queried 3.1.3.5) // other EC numbers for this reaction: 3.1.3.v

#adenosine salvage
adenosine-salvage/pa1_3.1.3.5_3.1.3.2_BS73811 : 2.0 H2O + 1.0 ADP --> 2.0 phosphate + 1.0 adenosine # source: BRENDA (queried 3.1.3.5, 3.1.3.2) // other EC numbers for this reaction: 3.6.1.5
adenosine-salvage/pb1_3.1.3.5_BS73810 : 1.0 H2O + 1.0 ADP --> 1.0 diphosphate + 1.0 adenosine # source: BRENDA (queried 3.1.3.5) // other EC numbers for this reaction: 3.1.3.31, 3.6.1.19
adenosine-salvage/1_3.5.4.4_ADENODEAMIN-RXN : 1.0 H2O + 1.0 adenosine + 1.0 H+ --> 1.0 ammonium + 1.0 inosine # source: MetaCyc, BRENDA, KEGG (queried 3.5.4.4)
adenosine-salvage/2_2.4.2.1_INOPHOSPHOR-RXN : 1.0 phosphate + 1.0 inosine <=> 1.0 alpha-D-ribose-1-phosphate + 1.0 hypoxanthine # source: MetaCyc, BRENDA, KEGG (queried 2.4.2.1) // other EC numbers for this reaction: 2.4.2.15, 2.4.2.3, 2.4.2.44
adenosine-salvage/3_1.17.1.4_RXN-7682 : 1.0 H2O + 1.0 NAD+ + 1.0 hypoxanthine --> 1.0 xanthine + 1.0 H+ + 1.0 NADH # source: MetaCyc, BRENDA, KEGG (queried 1.17.1.4) // other EC numbers for this reaction: 1.17.3.2

#DNA-Abbau
#dCMP
dCMP-salvage/1a_3.1.3.5_RXN0-5292 : 1.0 dCMP + 1.0 H2O --> 1.0 2'-deoxycytidine + 1.0 phosphate # source: MetaCyc, BRENDA, KEGG (queried 3.1.3.5) // other EC numbers for this reaction: 3.1.3.89
dCMP-salvage/2_3.5.4.5_CYTIDEAM-RXN : 1.0 H+ + 1.0 H2O + 1.0 2'-deoxycytidine --> 1.0 2'-deoxyuridine + 1.0 ammonium # source: MetaCyc, BRENDA, KEGG (queried 3.5.4.5)
dCMP-salvage/11b_3.5.4.12_DCMP-DEAMINASE-RXN : 1.0 H2O + 1.0 H+ + 1.0 dCMP --> 1.0 dUMP + 1.0 ammonium # source: MetaCyc, BRENDA, KEGG (queried 3.5.4.12)
#dUMP
dUMP-salvage/1_3.1.3.5_RXN-14143 : 1.0 H2O + 1.0 dUMP --> 1.0 phosphate + 1.0 2'-deoxyuridine # source: MetaCyc, BRENDA, KEGG (queried 3.1.3.5) // other EC numbers for this reaction: 3.1.3.89, 3.1.3.34
dUMP-salvage/2_2.4.2.1_2.4.2.2_URA-PHOSPH-RXN : 1.0 phosphate + 1.0 2'-deoxyuridine <=> 1.0 uracil + 1.0 2-deoxy-alpha-D-ribose_1-phosphate # source: MetaCyc, BRENDA, KEGG (queried 2.4.2.1, 2.4.2.2) // other EC numbers for this reaction: 2.4.2.3, 2.4.2.4
#dAMP
dAMP-salvage/1_3.1.3.5_3.1.3.2_RXN-14161 : 1.0 H2O + 1.0 dAMP --> 1.0 2'-deoxyadenosine + 1.0 phosphate # source: MetaCyc, BRENDA, KEGG (queried 3.1.3.5, 3.1.3.2) // other EC numbers for this reaction: 3.1.3.89
dAMP-salvage/2a_2.4.2.1_DEOXYADENPHOSPHOR-RXN : 1.0 phosphate + 1.0 2'-deoxyadenosine <=> 1.0 adenine + 1.0 2-deoxy-alpha-D-ribose_1-phosphate # source: MetaCyc, BRENDA, KEGG (queried 2.4.2.1)
dAMP-salvage/2b_3.5.4.4_ADDALT-RXN : 1.0 H+ + 1.0 H2O + 1.0 2'-deoxyadenosine --> 1.0 ammonium + 1.0 2'-deoxyinosine # source: MetaCyc, BRENDA, KEGG (queried 3.5.4.4) // other EC numbers for this reaction: 3.5.4.17
dAMP-salvage/3_2.4.2.1_DEOXYINOPHOSPHOR-RXN : 1.0 phosphate + 1.0 2'-deoxyinosine <=> 1.0 hypoxanthine + 1.0 2-deoxy-alpha-D-ribose_1-phosphate # source: MetaCyc, BRENDA, KEGG (queried 2.4.2.1) // other EC numbers for this reaction: 2.4.2.4
#dGMP
dGMP-salvage/1_3.1.3.5_3.1.3.2_RXN-14142 : 1.0 H2O + 1.0 dGMP --> 1.0 phosphate + 1.0 2'-deoxyguanosine # source: MetaCyc, BRENDA, KEGG (queried 3.1.3.5, 3.1.3.2) // there are 4 more EC numbers for this reaction
dGMP-salvage/2_2.4.2.1_DEOXYGUANPHOSPHOR-RXN : 1.0 phosphate + 1.0 2'-deoxyguanosine <=> 1.0 2-deoxy-alpha-D-ribose_1-phosphate + 1.0

```
guanine  # source: MetaCyc, BRENDA, KEGG (queried 2.4.2.1) // other EC numbers for this reaction: 2.4.2.15, 2.4.2.4
#dTMP
dTMP-salvage/1_3.1.3.5_THYMIDYLATE-5-PHOSPHATASE-RXN                                 : 1.0 H2O + 1.0 dTMP --> 1.0 phosphate + 1.0 thymidine  # source: MetaCyc, BRENDA, KEGG
(queried 3.1.3.5) // other EC numbers for this reaction: 3.1.3.89, 3.1.3.35
dTMP-salvage/2_2.4.2.2_THYM-PHOSPH-RXN                                               : 1.0 thymidine + 1.0 phosphate <=> 1.0 thymine + 1.0 2-deoxy-alpha-D-ribose_1-phosphate
# source: MetaCyc, BRENDA, KEGG (queried 2.4.2.2) // other EC numbers for this reaction: 2.4.2.4

#Salvage - like guanine and guanosine salvage II
#adenosine
adenosine-salvage/1_3.2.2.1_BR16044                                                  : 1.0 adenosine + 1.0 H2O --> 1.0 beta-D-ribopyranose + 1.0 adenine  # source: BRENDA,
KEGG (queried 3.2.2.1) // other EC numbers for this reaction: 3.2.2.8, 3.2.2.7
adenosine-salvage/2_3.5.4.2_ADENINE-DEAMINASE-RXN                                    : 1.0 H2O + 1.0 H+ + 1.0 adenine --> 1.0 hypoxanthine + 1.0 ammonium  # source: MetaCyc,
BRENDA, KEGG (queried 3.5.4.2)
#guanosine
guanosine-salvage/1_3.2.2.1_RXN0-366                                                 : 1.0 H2O + 1.0 guanosine --> 1.0 beta-D-ribopyranose + 1.0 guanine  # source: MetaCyc
(queried 3.2.2.1)
guanosine-salvage/2_2.4.2.8_2.4.2.7_2.4.2.22_GUANPRIBOSYLTRAN-RXN                    : 1.0 GMP + 1.0 diphosphate <=> 1.0 guanine + 1.0 5-phospho-alpha-D-ribose_1-diphosphate
# source: MetaCyc, BRENDA, KEGG (queried 2.4.2.8, 2.4.2.7, 2.4.2.22)
#inosine
inosine-salvage/1_3.2.2.1_BR16042                                                    : 1.0 inosine + 1.0 H2O --> 1.0 hypoxanthine + 1.0 beta-D-ribopyranose  # source: BRENDA,
KEGG (queried 3.2.2.1) // other EC numbers for this reaction: 3.2.2.2, 3.2.2.8
inosine-salvage/2_2.4.2.8_HYPOXANPRIBOSYLTRAN-RXN                                    : 1.0 diphosphate + 1.0 IMP <=> 1.0 hypoxanthine + 1.0 5-phospho-alpha-D-ribose_1-
diphosphate  # source: MetaCyc, BRENDA, KEGG (queried 2.4.2.8)
#xanthosine
xanthosine-salvage_3.2.2.1_RXN0-363                                                  : 1.0 xanthosine + 1.0 H2O --> 1.0 beta-D-ribopyranose + 1.0 xanthine  # source: MetaCyc
(queried 3.2.2.1)
#cytidine
cytidine-salvage_3.2.2.1_BS214504                                                                            : 1.0 cytidine + 1.0 H2O --> 1.0 cytosine + 1.0 beta-D-
ribopyranose  # source: BRENDA, KEGG (queried 3.2.2.1) // other EC numbers for this reaction: 3.2.2.3, 3.2.2.8
######################################################################################################################################################
#2'-deoxy-α-D-ribose 1-phosphate degradation

2'-deoxy-alpha-D-ribose-1-phosphate-degr/1_5.4.2.7_D-PPENTOMUT-RXN                   : 1.0 2-deoxy-alpha-D-ribose_1-phosphate <=> 1.0 2-deoxy-D-ribose_5-phosphate  # source:
MetaCyc, BRENDA, KEGG (queried 5.4.2.7)
2'-deoxy-alpha-D-ribose-1-phosphate-degr/2_4.1.2.4_DEOXYRIBOSE-P-ALD-RXN             : 1.0 2-deoxy-D-ribose_5-phosphate <=> 1.0 D-glyceraldehyde_3-phosphate + 1.0 acetaldehyde
# source: MetaCyc, BRENDA, KEGG (queried 4.1.2.4)

acetaldehyde-degradation_1.2.1.3_1.17.1.4_RXN66-3                                    : 1.0 H2O + 1.0 NAD+ + 1.0 acetaldehyde --> 1.0 acetate + 2.0 H+ + 1.0 NADH  # source:
MetaCyc, BRENDA, KEGG (queried 1.2.1.3, 1.17.1.4) // there are 20 more EC numbers for this reaction

#alpha-D-ribose-1-phosphate degradation
PRPP-biosynthesis-II_5.4.2.2_5.4.2.7_PPENTOMUT-RXN                                   : 1.0 alpha-D-ribose-1-phosphate <=> 1.0 D-ribose_5-phosphate  # source: MetaCyc, BRENDA,
KEGG (queried 5.4.2.2, 5.4.2.7)

#beta-D-ribopyranose degradation
beta-D-ribopyranose-degradation_modb_2.7.1.15_BR47755                                : 1.0 ATP + 1.0 beta-D-ribopyranose --> 1.0 D-ribose_5-phosphate + 1.0 ADP + 1.0 H+ #
source: BRENDA, KEGG (queried 2.7.1.15)
######################################################################################################################################################
#(d)NXP-transitions (2.7.4.25,2.7.4.3,2.7.4.22,2.7.4.8,2.7.4.9 + not sequence based: 2.7.4.6)

NXP-transitions_2.7.4.3_RXN-14074                                    : 1.0 GTP + 1.0 AMP --> 1.0 ADP + 1.0 GDP  # source: MetaCyc, BRENDA (queried 2.7.4.3) // other EC numbers
for this reaction: 2.7.4.4, 2.7.4.10
NXP-transitions_2.7.4.8_GUANYL-KIN-RXN                               : 1.0 ATP + 1.0 GMP --> 1.0 ADP + 1.0 GDP  # source: MetaCyc, BRENDA, KEGG (queried 2.7.4.8)
NXP-transitions_2.7.4.6_GDPKIN-RXN                                   : 1.0 ATP + 1.0 GDP --> 1.0 ADP + 1.0 GTP  # source: MetaCyc, BRENDA, KEGG (queried 2.7.4.6)

NXP-transitions_2.7.4.22_RXN-12002                                   : 1.0 UMP + 1.0 ATP --> 1.0 ADP + 1.0 UDP  # source: MetaCyc, BRENDA, KEGG (queried 2.7.4.22) // other EC
numbers for this reaction: 2.7.4.4, 2.7.4.14
NXP-transitions_2.7.4.3_BS358186                                     : 1.0 UTP + 1.0 AMP <-- 1.0 ADP + 1.0 UDP  # source: BRENDA, KEGG (queried 2.7.4.3) // other EC numbers for
this reaction: 2.7.4.10

NXP-transitions_2.7.4.25_2.7.4.3_RXN-11832                           : 1.0 CMP + 1.0 ATP --> 1.0 ADP + 1.0 CDP  # source: MetaCyc, BRENDA, KEGG (queried 2.7.4.25, 2.7.4.3) //
other EC numbers for this reaction: 2.7.4.14
NXP-transitions_2.7.4.3_CDPKIN-RXN                                   : 1.0 ATP + 1.0 CDP --> 1.0 ADP + 1.0 CTP  # source: MetaCyc, BRENDA, KEGG (queried 2.7.4.3) // other EC
numbers for this reaction: 2.7.4.6
```

```
NXP-transitions_2.7.4.3_ADENYL-KIN-RXN                                 : 1.0 ATP + 1.0 AMP --> 2.0 ADP  # source: MetaCyc, BRENDA, KEGG (queried 2.7.4.3)

NXP-transitions_2.7.4.3_DEOXYADENYLATE-KINASE-RXN                      : 1.0 dAMP + 1.0 ATP --> 1.0 ADP + 1.0 dADP  # source: MetaCyc, BRENDA, KEGG (queried 2.7.4.3) // other EC
numbers for this reaction: 2.7.4.13, 2.7.4.11
NXP-transitions_2.7.4.8_GMKALT-RXN                                     : 1.0 dGMP + 1.0 ATP --> 1.0 ADP + 1.0 dGDP  # source: MetaCyc, BRENDA, KEGG (queried 2.7.4.8) // other EC
numbers for this reaction: 2.7.4.12, 2.7.4.13
NXP-transitions_2.7.4.25_RXN-7913                                      : 1.0 ATP + 1.0 dCMP --> 1.0 ADP + 1.0 dCDP  # source: MetaCyc, BRENDA, KEGG (queried 2.7.4.25) // other EC
numbers for this reaction: 2.7.4.14, 2.7.4.13
NXP-transitions_2.7.4.9_DTMPKI-RXN                                     : 1.0 dTMP + 1.0 ATP --> 1.0 dTDP + 1.0 ADP  # source: MetaCyc, BRENDA, KEGG (queried 2.7.4.9) // other EC
numbers for this reaction: 2.7.4.12, 2.7.4.13
NXP-transitions_2.7.4.9_RXN-17141                                      : 1.0 GTP + 1.0 dTMP --> 1.0 GDP + 1.0 dTDP  # source: MetaCyc, BRENDA (queried 2.7.4.9) // other EC numbers
for this reaction: 2.7.4.M1

NXP-transitions_2.7.4.6_DTDPKIN-RXN                                    : 1.0 dTDP + 1.0 ATP --> 1.0 ADP + 1.0 dTTP  # source: MetaCyc, BRENDA, KEGG (queried 2.7.4.6)

NXP-transitions_2.7.4.22_2.7.4.9_2.7.4.25_RXN-14122                    : 1.0 ATP + 1.0 dUMP --> 1.0 ADP + 1.0 dUDP  # source: MetaCyc, BRENDA, KEGG (queried 2.7.4.22, 2.7.4.9,
2.7.4.25) // there are 4 more EC numbers for this reaction

NXP-transitions_2.7.4.6_DADPKIN-RXN                                    : 1.0 dADP + 1.0 ATP --> 1.0 ADP + 1.0 dATP  # source: MetaCyc, BRENDA, KEGG (queried 2.7.4.6)
NXP-transitions_2.7.4.6_DGDPKIN-RXN                                    : 1.0 dGDP + 1.0 ATP --> 1.0 ADP + 1.0 dGTP  # source: MetaCyc, BRENDA, KEGG (queried 2.7.4.6)
NXP-transitions_2.7.4.6_DUDPKIN-RXN                                    : 1.0 ATP + 1.0 dUDP --> 1.0 ADP + 1.0 dUTP  # source: MetaCyc, BRENDA, KEGG (queried 2.7.4.6)
NXP-transitions_2.7.4.6_DCDPKIN-RXN                                    : 1.0 dCDP + 1.0 ATP --> 1.0 ADP + 1.0 dCTP  # source: MetaCyc, BRENDA, KEGG (queried 2.7.4.6)
############################################################################################################################################################################
#ppGpp
ppGpp-biosynthesis_2.7.6.5_GDPPYPHOSKIN-RXN                            : 1.0 GDP + 1.0 ATP --> 1.0 AMP + 1.0 ppGpp  # source: MetaCyc, BRENDA (queried 2.7.6.5) // other EC numbers
for this reaction: 2.7.6.4
ppGpp-biosynthesis_2.7.6.5_GTPPYPHOSKIN-RXN                            : 1.0 GTP + 1.0 ATP --> 1.0 AMP + 1.0 pppGpp  # source: MetaCyc, BRENDA, KEGG (queried 2.7.6.5)
ppGpp-biosynthesis_3.6.1.40_PPPGPPHYDRO-RXN                            : 1.0 H2O + 1.0 pppGpp --> 1.0 ppGpp + 1.0 phosphate + 1.0 H+  # source: MetaCyc, BRENDA, KEGG (queried
3.6.1.40) // other EC numbers for this reaction: 3.6.1.11
############################################################################################################################################################################
#uracil degr I
uracil-degr-I/1_1.3.1.1_DIHYDROURACIL-DEHYDROGENASE-NAD+-RXN           : 1.0 5,6-dihydrouracil + 1.0 NAD+ <=> 1.0 H+ + 1.0 NADH + 1.0 uracil  # source: MetaCyc, BRENDA, KEGG
(queried 1.3.1.1)
uracil-degr-I/2_3.5.2.2_DIHYDROPYRIMIDINASE-RXN                        : 1.0 5,6-dihydrouracil + 1.0 H2O --> 1.0 3-ureidopropanoate + 1.0 H+  # source: MetaCyc, BRENDA, KEGG
(queried 3.5.2.2)
uracil-degr-I/3_3.5.1.6_BETA-UREIDOPROPIONASE-RXN                      : 2.0 H+ + 1.0 3-ureidopropanoate + 1.0 H2O --> 1.0 ammonium + 1.0 CO2 + 1.0 beta-alanine  # source:
MetaCyc, BRENDA, KEGG (queried 3.5.1.6)

#thymine degr
thymine-degradation_1.3.1.1_RXN0-6565                                  : 1.0 NAD+ + 1.0 5,6-dihydrothymine <=> 1.0 thymine + 1.0 H+ + 1.0 NADH  # source: MetaCyc, BRENDA, KEGG
(queried 1.3.1.1)
thymine-degradation_3.5.2.2_RXN-11211                                  : 1.0 5,6-dihydrothymine + 1.0 H2O --> 1.0 (R)-3-ureido-isobutanoate + 1.0 H+  # source: MetaCyc, BRENDA
(queried 3.5.2.2)
thymine-degradation_3.5.1.6_RXN-11210                                  : 1.0 H2O + 2.0 H+ + 1.0 (R)-3-ureido-isobutanoate --> 1.0 (R)-3-amino-2-methylpropanoate + 1.0 CO2 + 1.0
ammonium  # source: MetaCyc, KEGG (queried 3.5.1.6)

############################################################################################################################################################################
###################################################################Non-MDM/CDMM substrates##################################################################################
############################################################################################################################################################################
#cytosine
cytosine_exchange                               : 1.0 cytosine[ex] <=>
thymine_exchange                                : 1.0 thymine[ex] <=>

##CDIF630erm_01905; CDIF630erm_03001
cytosine_transport                              : 1.0 Na+ + 1.0 cytosine <=> 1.0 cytosine[ex] + 1.0 Na+[ex]
thymine_transport                               : 1.0 Na+ + 1.0 thymine <=> 1.0 thymine[ex] + 1.0 Na+[ex]
############################################################################################################################################################################
#adenine/guanine/hypoxanthine
adenine_exchange                                : 1.0 adenine[ex] <=>
guanine_exchange                                : 1.0 guanine[ex] <=>
hypoxanthine_exchange                           : 1.0 hypoxanthine[ex] <=>

##CDIF630erm_02331; CDIF630erm_02961
```

```
adenine_transport                              : 1.0 Na+ + 1.0 adenine <=> 1.0 adenine[ex] + 1.0 Na+[ex]
guanine_transport                              : 1.0 Na+ + 1.0 guanine <=> 1.0 guanine[ex] + 1.0 Na+[ex]
hypoxanthine_transport                         : 1.0 Na+ + 1.0 hypoxanthine <=> 1.0 hypoxanthine[ex] + 1.0 Na+[ex]
############################################################################################################################################################################
#uracil
uracil_exchange                                : 1.0 uracil[ex] <=>

##CDIF630erm_02848
uracil_transport                               : 1.0 Na+ + 1.0 uracil <=> 1.0 uracil[ex] + 1.0 Na+[ex]
############################################################################################################################################################################
#xanthine/urate
xanthine_exchange                              : 1.0 xanthine[ex] <=>
urate_exchange                                 : 1.0 urate[ex] <=>

##CDIF630erm_02298; CDIF630erm_02313; CDIF630erm_02314; CDIF630erm_03472
xanthine_transport                             : 1.0 Na+ + 1.0 xanthine <=> 1.0 xanthine[ex] + 1.0 Na+[ex]
urate_transport                                : 1.0 Na+ + 1.0 urate <=> 1.0 urate[ex] + 1.0 Na+[ex]

urate-degradation_1.17.1.4_RXN0-901            : 1.0 xanthine + 1.0 H2O + 1.0 NAD+ <=> 1.0 urate + 1.0 H+ + 1.0 NADH  # source: MetaCyc, BRENDA, KEGG (queried 1.17.1.4)

############################################################################################################################################################################
asparagine_exchange                            : 1.0 L-asparagine[ex] <=>
glutamine_exchange                             : 1.0 L-glutamine[ex] <=>

#unknown, ABC-transporter is a guess
##CDIF630erm_01972-CDIF630erm_01974?; CDIF630erm_00868-CDIF630erm_00870?
glutamine_transport                            : 1.0 H2O + 1.0 ATP + 1.0 L-glutamine[ex] --> 1.0 L-glutamine + 1.0 ADP + 1.0 phosphate + 1.0 H+
asparagine_transport                           : 1.0 H2O + 1.0 ATP + 1.0 L-asparagine[ex] --> 1.0 H+ + 1.0 phosphate + 1.0 ADP + 1.0 L-asparagine
############################################################################################################################################################################
#4-hydroxyproline
4-hydroxyproline_exchange                                              : 1.0 trans-4-hydroxy-L-proline[ex] <=>
4-hydroxyproline_transport                                             : 1.0 Na+ + 1.0 trans-4-hydroxy-L-proline <=> 1.0 Na+[ex] + 1.0 trans-4-hydroxy-L-proline[ex]
4-hydroxyproline-degr/1_manual_4.2.1.-_HD-Hyp                          : 1.0 trans-4-hydroxy-L-proline <=> 1.0 (S)-1-pyrroline-5-carboxylate + 1.0 H2O
4-hydroxyproline-degr/2_1.5.1.2_PYRROLINECARBREDUCT-RXN                : 1.0 NADP+ + 1.0 L-proline <-- 1.0 (S)-1-pyrroline-5-carboxylate + 1.0 NADPH + 2.0 H+  # source: MetaCyc,
BRENDA, KEGG (queried 1.5.1.2)
############################################################################################################################################################################
#cystathionine
cystathionine_exchange                         : 1.0 L-cystathionine[ex] <=>

##CDIF630erm_02404-CDIF630erm_02406 vgl. Burguière et al., 2004
cystathionine_transport                        : 1.0 H2O + 1.0 ATP + 1.0 L-cystathionine[ex] --> 1.0 H+ + 1.0 phosphate + 1.0 ADP + 1.0 L-cystathionine

cystathionine-degr-A_4.4.1.8_RXN-15131         : 1.0 L-cystathionine --> 1.0 H+ + 1.0 2-aminoprop-2-enoate + 1.0 L-homocysteine  # source: MetaCyc, BRENDA (queried 4.4.1.8)
cystathionine-degr-B_4.4.1.1_RXN-14048         : 1.0 L-cystathionine --> 1.0 (2Z)-2-aminobut-2-enoate + 1.0 H+ + 1.0 L-cysteine  # source: MetaCyc, BRENDA, KEGG (queried 4.4.1.1)
############################################################################################################################################################################
spermidine_exchange                            : 1.0 spermidine[ex] <=>
putrescine_exchange                            : 1.0 putrescine[ex] <=>

##CDIF630erm_01160-CDIF630erm_01163
putrescine_transport_3.6.3.31_modb_BS141431    : 1.0 ATP + 1.0 H2O + 1.0 putrescine[ex] --> 1.0 putrescine + 1.0 ADP + 1.0 phosphate + 1.0 H+   # ource: MetaCyc, BRENDA (queried
3.6.3.31)
spermidine_transport_3.6.3.31_modb_BS141432    : 1.0 ATP + 1.0 H2O + 1.0 spermidine[ex] --> 1.0 spermidine + 1.0 phosphate + 1.0 ADP + 1.0 H+  # source: MetaCyc, BRENDA (queried
3.6.3.31)
############################################################################################################################################################################
#2,3-diaminopropanoate degradation
2,3-diaminopropanoate_exchange                                         : 1.0 |23-Diaminopropanoate|[ex] <=>
2,3-diaminopropanoate_transport                                        : 1.0 ATP + 1.0 |23-Diaminopropanoate|[ex] + 1.0 H2O --> 1.0 ADP + 1.0 phosphate + 1.0 |23-
Diaminopropanoate| + 1.0 H+
2,3-diaminopropanoate-degr_4.3.1.15_4.3.1.15-RXN                       : 1.0 |23-Diaminopropanoate| + 1.0 H2O --> 2.0 ammonium + 1.0 pyruvate  # source: MetaCyc, BRENDA, KEGG
(queried 4.3.1.15)

hydroxylamine_exchange                                                 : 1.0 hydroxylamine[ex] <=>
hydroxylamine_transport                                                : 1.0 hydroxylamine[ex] <=> 1.0 hydroxylamine
hydroxylamine_1.7.99.1_HYDROXYLAMINE-REDUCTASE-NADH-RXN                : 1.0 H2O + 1.0 NAD+ + 1.0 ammonium <=> 2.0 H+ + 1.0 NADH + 1.0 hydroxylamine  # source: MetaCyc, BRENDA,
```

```
KEGG (queried 1.7.99.1) // other EC numbers for this reaction: 1.7.1.10
############################################################################################################################################################################
#ethanolamine utilization
#(Pitts et al., 2012)
ethanolamine_exchange                                                                 : 1.0 ethanolamine[ex] <=>
##CDIF630erm_02128
ethanolamine_transport1                                                               : 1.0 ethanolamine <=> 1.0 ethanolamine[ex]
##CDIF630erm_02119
ethanolamine_transport2                                                               : 1.0 ethanolamine[BMC] <-- 1.0 ethanolamine

ethanolamine-utilization/1_4.3.1.7_ETHAMLY-RXN                                        : 1.0 ethanolamine[BMC] --> 1.0 ammonium + 1.0 acetaldehyde[BMC]  # source: MetaCyc, BRENDA,
KEGG (queried 4.3.1.7)
ethanolamine-utilization/2_1.2.1.10_ACETALD-DEHYDROG-RXN                              : 1.0 NAD+[BMC] + 1.0 coenzyme_A[BMC] + 1.0 acetaldehyde[BMC] <=> 1.0 NADH[BMC] + 1.0 acetyl-
CoA[BMC] + 1.0 H+  # source: MetaCyc, BRENDA, KEGG (queried 1.2.1.10)
ethanolamine-utilization/3_2.3.1.8_PHOSACETYLTRANS-RXN                                : 1.0 acetyl-CoA[BMC] + 1.0 phosphate <=> 1.0 acetyl_phosphate + 1.0 coenzyme_A[BMC]  #
source: MetaCyc, BRENDA, KEGG (queried 2.3.1.8)
ethanolamine-utilization/4_1.1.1.1_ALCOHOL-DEHYDROGENASE-NADP+-RXN_WOP                : 1.0 NAD+[BMC] + 1.0 ethanol <=> 1.0 NADH[BMC] + 1.0 acetaldehyde[BMC] + 1.0 H+  # source:
MetaCyc, BRENDA, KEGG (queried 1.1.1.1) // other EC numbers for this reaction: 1.1.1.71
############################################################################################################################################################################
sn-glycerol-3-phosphate_exchange              : 1.0 sn-glycerol_3-phosphate[ex] <=>

##CDIF630erm_03719-CDIF630erm_03722
sn-glycerol-3-phosphate_transport             : 1.0 ATP + 1.0 sn-glycerol_3-phosphate[ex] + 1.0 H2O --> 1.0 ADP + 1.0 sn-glycerol_3-phosphate + 1.0 phosphate + 1.0 H+
############################################################################################################################################################################
#methylphosphonate degr II
methylphosphonate_exchange                                                           : 1.0 methylphosphonate[ex] <=>
##CDIF630erm_03847-CDIF630erm_03848
methylphosphonate_transport                                                          : 1.0 methylphosphonate + 1.0 ADP + 1.0 H+ <-- 1.0 methylphosphonate[ex] + 1.0 ATP + 1.0 H2O
methane_transport                                                                    : 1.0 methane <=> 1.0 methane[ex]
methane_exchange                                                                     : 1.0 methane[ex] <=>

methylphosphonate-degr-II/1_2.7.8.37_RXN0-6732                                       : 1.0 ATP + 1.0 methylphosphonate --> 1.0 adenine + 1.0 alpha-D-ribose-1-methylphosphonate-5-
triphosphate  # source: MetaCyc, BRENDA, KEGG (queried 2.7.8.37)
methylphosphonate-degr-II/2_3.6.1.63_RXN0-6733                                       : 1.0 H2O + 1.0 alpha-D-ribose-1-methylphosphonate-5-triphosphate --> 1.0 H+ + 1.0 diphosphate
+ 1.0 alpha-D-ribose-1-methylphosphonate_5-phosphate  # source: MetaCyc, BRENDA, KEGG (queried 3.6.1.63)
methylphosphonate-degr-II/3_4.7.1.1_RXN0-6734                                        : 1.0 alpha-D-ribose-1-methylphosphonate_5-phosphate --> 1.0 5-phospho-alpha-D-ribose_1,2-
cyclic_phosphate + 1.0 methane  # source: MetaCyc, BRENDA, KEGG (queried 4.7.1.1)
methylphosphonate-degr-II/4_3.1.4.57_RXN-14995                                       : 1.0 H2O + 1.0 5-phospho-alpha-D-ribose_1,2-cyclic_phosphate --> 1.0 D-ribofuranose_2,5-
bisphosphate + 1.0 H+  # source: MetaCyc, BRENDA, KEGG (queried 3.1.4.57)
methylphosphonate-degr-II/5_3.1.4.57_RXN-14996                                       : 1.0 D-ribofuranose_2,5-bisphosphate + 1.0 H2O --> 1.0 phosphate + 1.0 D-ribose_5-phosphate
# source: MetaCyc, BRENDA, KEGG (queried 3.1.4.57)
############################################################################################################################################################################
#2-aminoethylP degr I
2-aminoethylphosphonate_exchange                                                     : 1.0 (2-aminoethyl)phosphonate[ex] <=>
##CDIF630erm_03847-CDIF630erm_03848
2-aminoethylphosphonate_transport                                                    : 1.0 2-aminoethylphosphonate + 1.0 ADP + 1.0 H+ <-- 1.0 (2-aminoethyl)phosphonate[ex] + 1.0
ATP + 1.0 H2O
2-aminoethylphosph-degr-I/1_2.6.1.37_2.6.1.37-RXN                                    : 1.0 pyruvate + 1.0 (2-aminoethyl)phosphonate <=> 1.0 phosphonoacetaldehyde + 1.0 L-alanine
# source: MetaCyc, BRENDA, KEGG (queried 2.6.1.37)
2-aminoethylphosph-degr-I/2_3.11.1.1_PHOSPHONOACETALDEHYDE-HYDROLASE-RXN             : 1.0 phosphonoacetaldehyde + 1.0 H2O --> 1.0 acetaldehyde + 1.0 phosphate + 1.0 H+  # source:
MetaCyc, BRENDA, KEGG (queried 3.11.1.1)
############################################################################################################################################################################
#phosphonoacetate degradation
phosphonoacetate_exchange                                                            : 1.0 phosphonoacetate[ex] <=>
##CDIF630erm_03847-CDIF630erm_03848
phosphonoacetate_transport                                                           : 1.0 phosphonoacetate + 1.0 ADP + 1.0 H+ + 1.0 phosphate <-- 1.0 phosphonoacetate[ex] + 1.0
ATP + 1.0 H2O
phosphonoacetate-degradation_3.11.1.2_3.11.1.2-RXN                                   : 1.0 H2O + 1.0 phosphonoacetate --> 1.0 acetate + 1.0 phosphate + 1.0 H+  # source: MetaCyc,
BRENDA, KEGG (queried 3.11.1.2)
############################################################################################################################################################################
succinate_exchange                                                                   : 1.0 succinate[ex] <=>
##CDIF630erm_02583
succinate_transport                                                                  : 1.0 Na+ + 1.0 succinate <=> 1.0 succinate[ex] + 1.0 Na+[ex]
############################################################################################################################################################################
```

```
#tartronate semialdehyde
tartronate-semialdehyde_exchange                                          : 1.0 tartronate_semialdehyde[ex] <=>
tartronate-semialdehyde_transport                                         : 1.0 tartronate_semialdehyde[ex] + 1.0 Na+[ex] <=> 1.0 tartronate_semialdehyde + 1.0 Na+

tartronate-semialdehyde-degr_1.1.1.60_TSA-REDUCT-RXN_WOP                  : 1.0 NAD+ + 1.0 D-glycerate <=> 1.0 NADH + 1.0 H+ + 1.0 tartronate_semialdehyde  # source:
MetaCyc, BRENDA, KEGG (queried 1.1.1.60) // other EC numbers for this reaction: 1.1.1
tartronate-semialdehyde-degr_1.1.1.60_TSA-REDUCT-RXN                      : 1.0 NADP+ + 1.0 D-glycerate <-- 1.0 tartronate_semialdehyde + 1.0 H+ + 1.0 NADPH  # source:
MetaCyc, BRENDA, KEGG (queried 1.1.1.60)
###########################################################################################################################################################################
#Acetoin degradation
acetoin_exchange                                                          : 1.0 acetoin[ex] <=>
acetoin_transport                                                         : 1.0 acetoin[ex] + 1.0 H+[ex] <=> 1.0 acetoin + 1.0 H+ # metacyc
acetoin-degradation_2.3.1.190_RXN-9718                                    : 1.0 acetoin + 1.0 NAD+ + 1.0 coenzyme_A --> 1.0 acetyl-CoA + 1.0 acetaldehyde + 1.0 H+ + 1.0
NADH  # source: MetaCyc, BRENDA, KEGG (queried 2.3.1.190)
###########################################################################################################################################################################
#3-hydroxy-3-methylglutarate
3-hydroxy-3-methylglutarate_exchange                                      : 1.0 3-hydroxy-3-methylglutarate[ex] <=>
##CDIF630erm_00917
3-hydroxy-3-methylglutarate_transport                                     : 1.0 H+ + 1.0 3-hydroxy-3-methylglutarate <=> 1.0 H+[ex] + 1.0 3-hydroxy-3-
methylglutarate[ex]
3-hydroxy-3-methylglutarate-degr/1_manual_2.8.3.-_HD-3MG                  : 1.0 acetyl-CoA + 1.0 3-hydroxy-3-methylglutarate --> 1.0 (S)-3-hydroxy-3-methylglutaryl-CoA
+ 1.0 acetate  # source: MetaCyc, BRENDA, KEGG (queried 2.8.3.-) // other EC numbers for this reaction: 2.8.3.1, 2.8.3
3-hydroxy-3-methylglutarate-degr/2_4.1.3.4_HYDROXYMETHYLGLUTARYL-COA-LYASE-RXN       : 1.0 (S)-3-hydroxy-3-methylglutaryl-CoA --> 1.0 acetyl-CoA + 1.0 acetoacetate  # source:
MetaCyc, BRENDA, KEGG (queried 4.1.3.4)
###########################################################################################################################################################################
#oxidized-GTP
hydroxyl-radical_exchange                                                 : 1.0 hydroxyl_radical[ex] <--
hydroxyl-radical_transport                                                : 1.0 hydroxyl_radical[ex] --> 1.0 hydroxyl_radical
oxidized-GTP-and-dGTP-detoxification_spontaneous_RXN-11409                : 1.0 GTP + 2.0 hydroxyl_radical --> 1.0 8-oxo-GTP + 1.0 H2O  # source: MetaCyc
oxidized-GTP-and-dGTP-detoxification_spontaneous_RXN-11410                : 2.0 hydroxyl_radical + 1.0 dGTP --> 1.0 8-oxo-dGTP + 1.0 H2O  # source: MetaCyc
oxidized-GTP-and-dGTP-detoxification_3.6.1.55_RXN-11397                   : 1.0 H2O + 1.0 8-oxo-GTP --> 1.0 8-oxo-GMP + 1.0 diphosphate + 1.0 H+  # source: MetaCyc,
BRENDA (queried 3.6.1.55) // other EC numbers for this reaction: 3.6.1, 3.6.1.19, 3.6.1.56
oxidized-GTP-and-dGTP-detoxification_3.6.1.55_RXN-11396                   : 1.0 H2O + 1.0 8-oxo-dGTP --> 1.0 8-oxo-dGMP + 1.0 diphosphate + 1.0 H+  # source: MetaCyc,
BRENDA, KEGG (queried 3.6.1.55) // other EC numbers for this reaction: 3.6.1.65
8-oxo-GMP_transport                                                       : 1.0 8-oxo-GMP[ex] <-- 1.0 8-oxo-GMP
8-oxo-dGMP_transport                                                      : 1.0 8-oxo-dGMP[ex] <-- 1.0 8-oxo-GMP
8-oxo-GMP_exchange                                                        : 1.0 8-oxo-GMP[ex] -->
8-oxo-dGMP_exchange                                                       : 1.0 8-oxo-dGMP[ex] -->
###########################################################################################################################################################################
#reactive oxygen detoxification
superoxide_exchange                                          : 1.0 superoxide[ex] <--
hydrogen_peroxide_exchange                                   : 1.0 hydrogen_peroxide[ex] <--
oxygen_exchange                                              : 1.0 oxygen[ex] <--
mathanol_exchange                                            : 1.0 methanol[ex] <--
superoxide_transport                                         : 1.0 superoxide <=> 1.0 superoxide[ex]
hydrogen_peroxide_transport                                  : 1.0 hydrogen_peroxide <=> 1.0 hydrogen_peroxide[ex]
oxygen_transport                                             : 1.0 oxygen <=> 1.0 oxygen[ex]
mathanol_transport                                           : 1.0 methanol <=> 1.0 methanol[ex]


superoxide-radicals-degr-A/1_1.15.1.1_SUPEROX-DISMUT-RXN     : 2.0 H+ + 2.0 superoxide --> 1.0 hydrogen_peroxide + 1.0 oxygen  # source: MetaCyc, BRENDA, KEGG (queried
1.15.1.1)
superoxide-radicals-degr-A/2a_1.11.1.6_CATAL-RXN             : 2.0 hydrogen_peroxide --> 2.0 H2O + 1.0 oxygen  # source: MetaCyc, BRENDA, KEGG (queried 1.11.1.6) //
other EC numbers for this reaction: 1.11.1.21
superoxide-radicals-degr-A/2b_1.11.1.6_RXN66-1               : 1.0 ethanol + 1.0 hydrogen_peroxide --> 1.0 acetaldehyde + 2.0 H2O  # source: MetaCyc (queried 1.11.1.6)
superoxide-radicals-degr-A/2c_moddir_1.11.1.6_RXN-14189      : 1.0 methanol + 1.0 hydrogen_peroxide <=> 1.0 formaldehyde + 2.0 H2O  # source: MetaCyc, BRENDA, KEGG
(queried 1.11.1.6) // other EC numbers for this reaction: 1.11.1.7, 1.11.1.21

superoxide-radicals-degr-B/1_1.15.1.2_1.15.1.2-RXN           : 1.0 superoxide + 1.0 |Reduced-Rubredoxins| + 2.0 H+ <=> 1.0 |Oxidized-Rubredoxins| + 1.0 hydrogen_peroxide
# source: MetaCyc, BRENDA, KEGG (queried 1.15.1.2)
superoxide-radicals-degr-B/2_manual_1.11.1.-_HD-1.11.1.-     : 1.0 |Reduced-Rubredoxins| + 2.0 H+ + 1.0 hydrogen_peroxide <=> 1.0 |Oxidized-Rubredoxins| + 2.0 H2O  #
source: MetaCyc, BRENDA, KEGG (queried 1.11.1.1)
superoxide-radicals-degr-B/3_manual_1.4.-_HD-1.4.-           : 2.0 L-glutamate + 1.0 |Oxidized-Rubredoxins| --> 1.0 L-glutamine + 1.0 2-oxoglutarate + 1.0 |Reduced-
Rubredoxins| + 2.0 H+  # source: MetaCyc, KEGG (queried 1.4.7.1)
```

```
oxygen-reduction-A_1.6.3.-_HD-1.6.3.-                                   : 2.0 |Reduced-Rubredoxins| + 4.0 H+ + 1.0 oxygen --> 2.0 |Oxidized-Rubredoxins| + 2.0 H2O

oxygen-reduction-B_1.6.3.4_RXN-14692                                    : 2.0 H+ + 2.0 NADH + 1.0 oxygen --> 2.0 NAD+ + 2.0 H2O  # source: MetaCyc, BRENDA, KEGG (queried 
1.6.3.4) // other EC numbers for this reaction: 1.6.3.2
formaldehyde-assimilation_spontaneous_2.1.2.11_RXN-2881                 : 1.0 formaldehyde + 1.0 tetrahydropteroyl_tri-L-glutamate --> 1.0 H2O + 1.0 5,10-methylene-tetrahydrofolate 
# source: MetaCyc
#####################################################################################################################################################################
#cyanide detoxification I
cyanide_exchange                                                            : 1.0 hydrogen_cyanide[ex] <--
thiocyanate_exchange                                                        : 1.0 thiocyanate[ex] <--
cyanide_transport                                                           : 1.0 hydrogen_cyanide <=> 1.0 hydrogen_cyanide[ex]
thiocyanate_transport                                                       : 1.0 thiocyanate + 1.0 H+ <=> 1.0 thiocyanate[ex] + 1.0 H+[ex]

cyanide-detoxification-I/1_4.4.1.9_L-3-CYANOALANINE-SYNTHASE-RXN            : 1.0 hydrogen_cyanide + 1.0 L-cysteine <=> 1.0 3-cyano-L-alanine + 1.0 hydrogen_sulfide + 1.0 H+  # 
source: MetaCyc, BRENDA, KEGG (queried 4.4.1.9)
cyanide-detoxification-I/2a_3.5.5.4_3.5.5.4-RXN                             : 2.0 H2O + 1.0 3-cyano-L-alanine + 1.0 H+ --> 1.0 ammonium + 1.0 L-aspartate  # source: MetaCyc, 
BRENDA, KEGG (queried 3.5.5.4)
cyanide-detoxification-I/2b_4.2.1.65_3.5.5.4_3-CYANOALANINE-HYDRATASE-RXN  : 1.0 L-asparagine <-- 1.0 3-cyano-L-alanine + 1.0 H+ + 1.0 H2O  # source: MetaCyc, BRENDA, KEGG 
(queried 4.2.1.65, 3.5.5.4)

#cyanide detoxification
cyanide-detoxification-III/1_2.8.1.2_R03105                                 : 1.0 sulfite + 1.0 3-mercaptopyruvate <=> 1.0 pyruvate + 1.0 thiosulfate  # source: KEGG (queried 
2.8.1.2)
cyanide-detoxification-III/2_2.8.1.1_THIOSULFATE-SULFURTRANSFERASE-RXN      : 1.0 thiosulfate + 1.0 hydrogen_cyanide --> 1.0 sulfite + 1.0 thiocyanate + 2.0 H+  # source: MetaCyc, 
BRENDA, KEGG (queried 2.8.1.1)

#Cysteine degr III
L-cysteine-degradation-III/1_2.6.1.1_CYSTEINE-AMINOTRANSFERASE-RXN          : 1.0 L-cysteine + 1.0 2-oxoglutarate <=> 1.0 3-mercaptopyruvate + 1.0 L-glutamate  # source: MetaCyc, 
BRENDA, KEGG (queried 2.6.1.1) // other EC numbers for this reaction: 2.6.1.3
L-cysteine-degradation-III/2_2.8.1.2_MERCAPYSTRANS-RXN                      : 1.0 hydrogen_cyanide + 1.0 3-mercaptopyruvate --> 1.0 thiocyanate + 1.0 H+ + 1.0 pyruvate  # source: 
MetaCyc, BRENDA, KEGG (queried 2.8.1.2)
#####################################################################################################################################################################
#bile acids

taurochenodeoxycholate_exchange                                                        : 1.0 taurochenodeoxycholate[ex] <=>
chenodeoxycholate_exchange                                                             : 1.0 chenodeoxycholate[ex] <=>
taurocholate_exchange                                                                  : 1.0 taurocholate[ex] <=>
cholate_exchange                                                                       : 1.0 cholate[ex] <=>

##DIF630erm_02170
taurochenodeoxycholate_transport                                                       : 1.0 H+[ex] + 1.0 taurochenodeoxycholate[ex] <=> 1.0 H+ + 1.0 taurochenodeoxycholate
chenodeoxycholate_transport                                                            : 1.0 H+[ex] + 1.0 chenodeoxycholate[ex] <=> 1.0 H+ + 1.0 chenodeoxycholate
taurocholate_transport                                                                 : 1.0 H+[ex] + 1.0 taurocholate[ex] <=> 1.0 H+ + 1.0 taurocholate
cholate_transport                                                                      : 1.0 H+[ex] + 1.0 cholate[ex] <=> 1.0 H+ + 1.0 cholate

#C. difficile can degrade taurocholate and no glycocholate (Midtvedt and Norman, 1967)
# => no EC 3.5.1.24; No sequences for EC 3.5.1.74 known
#Highest similarity to mouse enzyme. Mouse enzyme is taurine-specific (Falany et al., 1997)
#Reverse biosynthesis pathway

taurochenodeoxycholate-degr/1_2.3.1.65_RXN-9852                                        : 1.0 chenodeoxycholoyl-CoA + 1.0 taurine --> 1.0 taurochenodeoxycholate + 1.0 coenzyme_A + 
1.0 H+  # source: MetaCyc, BRENDA, KEGG (queried 2.3.1.65)
taurochenodeoxycholate-degr/2_3.1.2.2_RXN-14289                                        : 1.0 chenodeoxycholoyl-CoA + 1.0 H2O --> 1.0 coenzyme_A + 1.0 chenodeoxycholate + 1.0 H+  # 
source: MetaCyc, BRENDA, KEGG (queried 3.1.2.2) // other EC numbers for this reaction: 3.1.2.27, 3.1.2.20
taurochenodeoxycholate-degr/3_manual_1.1.1.-_HD-ChChol                                 : 1.0 chenodeoxycholate + 1.0 NADP+ --> 1.0 7-oxolithocholate + 1.0 NADPH + 1.0 H+

taurocholate-degr/1_moddir_2.3.1.65_RXN-9800                                           : 1.0 choloyl-CoA + 1.0 taurine <=> 1.0 coenzyme_A + 1.0 taurocholate + 1.0 H+  # source: 
MetaCyc, BRENDA, KEGG (queried 2.3.1.65)
taurocholate-degr/2_manual_3.1.2.2_HD-3.1.2.2                                          : 1.0 choloyl-CoA + 1.0 H2O --> 1.0 coenzyme_A + 1.0 cholate + 1.0 H+
taurocholate-degr/3_manual_1.1.1.-_HD-Chol                                             : 1.0 cholate + 1.0 NADP+ --> 1.0 7-oxodeoxycholate + 1.0 NADPH + 1.0 H+

7-oxodeoxycholate_transport                                                            : 1.0 7-oxodeoxycholate + 1.0 H+ --> 1.0 7-oxodeoxycholate[ex] + H+[ex]
7-oxolithocholate_transport                                                            : 1.0 7-oxolithocholate + 1.0 H+ --> 1.0 7-oxolithocholate[ex] + H+[ex]
```

```
taurine_transport : 1.0 taurine + 1.0 H+ --> 1.0 taurine[ex] + 1.0 H+[ex]

7-oxodeoxycholate_exchange : 1.0 7-oxodeoxycholate[ex] -->
7-oxolithocholate_exchange : 1.0 7-oxolithocholate[ex] -->
taurine_exchange : 1.0 taurine[ex] -->
##########################################################################################################################################################################
#choline degradation

choline_exchange : 1.0 choline[ex] <=>
choline_transport : 1.0 H+[ex] + 1.0 choline[ex] <=> 1.0 H+ + 1.0 choline
trimethylamine_exchange : 1.0 trimethylamine[ex] <=>
trimethylamine_transport : 1.0 H+[ex] + 1.0 trimethylamine[ex] <=> 1.0 H+ + 1.0 trimethylamine

choline-degradation-III_4.3.99.4_RXN-13946 : 1.0 choline --> 1.0 trimethylamine + 1.0 acetaldehyde  # source: MetaCyc, BRENDA, KEGG
(queried 4.3.99.4)
##########################################################################################################################################################################
#D-arabinose degr
D-arabinose_exchange : 1.0 D-arabinose[ex] <=>
##CDIF630erm_03311, CDIF630erm_03314; CDIF630erm_02563-CDIF630erm_02565
D-arabinose_transport_2.7.1.- : 1.0 D-arabinose[ex] + 1.0 |PTS-I-pi-phospho-L-histidines| --> 1.0 |PTS-I-Histidines| + 1.0
aldehydo-D-arabinose_5-phosphate
D-arabinose-degr_5.3.1.13_DARAB5PISOM-RXN : 1.0 aldehydo-D-arabinose_5-phosphate <=> 1.0 D-ribulose_5-phosphate  # source: MetaCyc,
BRENDA, KEGG (queried 5.3.1.13)
##########################################################################################################################################################################
#xylose degr I/ xylan degr
#set ratio for chain length
xylan_exchange : 1.0 |Xylans|[ex] <=>
xylose_exchange : 1.0 alpha-D-xylopyranose[ex] <=>
##CDIF630erm_03350-CDIF630erm_03353
xylan_transport : 1.0 |Xylans| <=> 1.0 |Xylans|[ex]
xylose_transport_2.7.1.- : 1.0 D-xylulose_5-phosphate + 1.0 |PTS-I-Histidines| <-- 1.0 alpha-D-xylopyranose[ex] + 1.0
|PTS-I-pi-phospho-L-histidines|

xylan-degradation_3.2.1.177_RXN0-5001 : 1.0 |Xylans| + 1.0 H2O --> 1.0 alpha-D-xylopyranose  # source: MetaCyc (queried 3.2.1.177)
xylose-degradation-I/1_5.3.1.5_XYLISOM-RXN : 1.0 alpha-D-xylopyranose <=> 1.0 D-xylulose  # source: MetaCyc (queried 5.3.1.5)
xylose-degradation-I/2_2.7.1.17_XYLULOKIN-RXN : 1.0 ATP + 1.0 D-xylulose --> 1.0 ADP + 1.0 H+ + 1.0 D-xylulose_5-phosphate  # source:
MetaCyc, BRENDA, KEGG (queried 2.7.1.17)
##########################################################################################################################################################################
#galactose degradation I (Leloir-pathway)
galactose_exchange : 1.0 alpha-D-galactose[ex] <=>
##CDIF630erm_00408-CDIF630erm_00413; CDIF630erm_01218, CDIF630erm_01220-CDIF630erm_01222; CDIF630erm_03576-CDIF630erm_03579?
galactose_transport_2.7.1.- : 1.0 alpha-D-galactose[ex] + 1.0 |PTS-I-pi-phospho-L-histidines| --> 1.0 |PTS-I-Histidines| +
1.0 alpha-D-galactose_1-phosphate

galactose-degr-I/1_2.7.7.12_GALACTURIDYLYLTRANS-RXN : 1.0 alpha-D-galactose_1-phosphate + 1.0 UDP-alpha-D-glucose <=> 1.0 UDP-alpha-D-galactose +
1.0 alpha-D-glucopyranose_1-phosphate  # source: MetaCyc, BRENDA, KEGG (queried 2.7.7.12)
galactose-degr-I/2_5.1.3.2_UDPGLUCEPIM-RXN : 1.0 UDP-alpha-D-glucose <=> 1.0 UDP-alpha-D-galactose  # source: MetaCyc, BRENDA, KEGG
(queried 5.1.3.2)
galactose-degr-I/3_spontaneous_RXN-10639 : 1.0 alpha-D-glucopyranose_1-phosphate <=> 1.0 beta-D-glucose_1-phosphate  # source: MetaCyc
##########################################################################################################################################################################
#xylitol degr A
xylitol_exchange : 1.0 xylitol[ex] <=>
#CDIF630erm_02563-CDIF630erm_02565
xylitol_transport : 1.0 |PTS-I-pi-phospho-L-histidines| + 1.0 xylitol[ex] --> 1.0 xylitol_5-phosphate + 1.0 |
PTS-I-Histidines|
xylitol-degr_manual_1.1.1.-_HD-xyl5 : 1.0 NAD+ + 1.0 xylitol_5-phosphate <=> 1.0 D-xylulose_5-phosphate + 1.0 NADH + 1.0 H+

#xylitol degr B
##CDIF630erm_02515-CDIF630erm_02517
xylitol_transport_2.7.1.- : 1.0 |PTS-I-pi-phospho-L-histidines| + 1.0 xylitol[ex] --> 1.0 xylitol_1-phosphate + 1.0 |
PTS-I-Histidines|
xylitol-degr_manual_1.1.1.-_HD-xyl1 : 1.0 NAD+ + 1.0 xylitol_1-phosphate <=> 1.0 D-xylulose_5-phosphate + 1.0 NADH + 1.0 H+
xylulose-degradation/1_5.1.3.4_RIBULPEPIM-RXN : 1.0 L-ribulose_5-phosphate <=> 1.0 D-xylulose_5-phosphate  # source: MetaCyc, BRENDA, KEGG
(queried 5.1.3.4)
```

```
#####################################################################################################################################################################
#arabitol degr
#arabitol degr
arabitol_exchange                                                    : 1.0 D-arabitol[ex] <=>
##CDIF630erm_02563 - CDIF630erm_02565
arabitol_transport                                                   : 1.0 |PTS-I-pi-phospho-L-histidines| + 1.0 D-arabitol[ex] --> 1.0 D-arabitol_1-phosphate +
1.0 |PTS-I-Histidines|

arabitol-degr_1.1.1.301_RXN-11106                                    : 1.0 D-arabitol_1-phosphate + 1.0 NAD+ <=> 1.0 H+ + 1.0 NADH + 1.0 D-xylulose_5-phosphate  #
source: MetaCyc, BRENDA, KEGG (queried 1.1.1.301)
#####################################################################################################################################################################
#ribitol degr
ribitol_exchange                                                     : 1.0 D-ribitol[ex] <=>
##CCDIF630erm_00620-CDIF630erm_00623
ribitol_transport_2.7.1.-                                            : 1.0 |PTS-I-pi-phospho-L-histidines| + 1.0 D-ribitol[ex] --> 1.0 D-ribitol_5-phosphate + 1.0
|PTS-I-Histidines|  # source: MetaCyc (queried 2.7.1.69)

ribitol-degr_1.1.1.137_RIBITOL-5-PHOSPHATE-2-DEHYDROGENASE-RXN_WOP   : 1.0 D-ribitol_5-phosphate + 1.0 NAD+ <=> 1.0 H+ + 1.0 D-ribulose_5-phosphate + 1.0 NADH  #
source: MetaCyc, BRENDA, KEGG (queried 1.1.1.137)
#####################################################################################################################################################################
#D-sorbitol degradation II
D-sorbitol_exchange                                                  : 1.0 D-sorbitol[ex] <=>
##CDIF630erm_00882-CDIF630erm_00885
D-sorbitol_transport_2.7.1.198                                       : 1.0 D-sorbitol[ex] + 1.0 |PTS-I-pi-phospho-L-histidines| --> 1.0 D-sorbitol_6-phosphate +
1.0 |PTS-I-Histidines|

D-sorbitol-degradation-II_1.1.1.140_SORB6PDEHYDROG-RXN               : 1.0 NAD+ + 1.0 D-sorbitol_6-phosphate <=> 1.0 beta-D-fructofuranose_6-phosphate + 1.0 NADH +
1.0 H+  # source: MetaCyc, BRENDA, KEGG (queried 1.1.1.140)

sorbitol-biosyn_1.1.1.17_BS257116                                    : 1.0 NAD+ + 1.0 D-sorbitol_6-phosphate <-- 1.0 NADH + 1.0 H+ + 1.0 D-glucose_6-phosphate  #
source: BRENDA (queried 1.1.1.17)
#####################################################################################################################################################################
#mannitol degradation I
D-mannitol_exchange                                                  : 1.0 D-mannitol[ex] <=>
##CDIF630erm_02571, CDIF630erm_02573
mannitol_transport_2.7.1.197                                         : 1.0 |PTS-I-pi-phospho-L-histidines| + 1.0 D-mannitol[ex] --> 1.0 D-mannitol_1-phosphate +
1.0 |PTS-I-Histidines|

mannitol-degradation-I_1.1.1.17_MANNPDEHYDROG-RXN                    : 1.0 NAD+ + 1.0 D-mannitol_1-phosphate <=> 1.0 NADH + 1.0 H+ + 1.0 beta-D-fructofuranose_6-
phosphate  # source: MetaCyc, BRENDA, KEGG (queried 1.1.1.17)
#####################################################################################################################################################################
#D-ribofuranose and 2'-deoxyribose degr C
D-ribopyranose_exchange                                              : 1.0 beta-D-ribopyranose[ex] <=>
2'-deoxyribose_exchange                                              : 1.0 2'-deoxyribose[ex] <=>
##CDIF630erm_00425-CDIF630erm_00427
D-ribopyranose_transport                                             : 1.0 ATP + 1.0 H2O + 1.0 beta-D-ribopyranose[ex] --> 1.0 ADP + 1.0 phosphate + 1.0 H+ + 1.0
beta-D-ribopyranose
2'-deoxyribose_transport                                             : 1.0 ATP + 1.0 H2O + 1.0 2'-deoxyribose[ex] --> 1.0 ADP + 1.0 phosphate + 1.0 H+ + 1.0 2'-
deoxyribose

2'-deoxyribose-degradation_2.7.1.15_RXN-14223                        : 1.0 2'-deoxyribose + 1.0 ATP --> 1.0 H+ + 1.0 ADP + 1.0 2-deoxy-D-ribose_5-phosphate  #
source: MetaCyc, BRENDA, KEGG (queried 2.7.1.15)
#####################################################################################################################################################################
#maltose degradation
maltose_exchange                                                     : 1.0 alpha-maltose[ex] <=>
##CDIF630erm_00981-CDIF630erm_00983;CDIF630erm_02759, CDIF630erm_02761; CDIF630erm_03341, CDIF630erm_03344
maltose_transport_2.7.1.208                                          : 1.0 alpha-maltose[ex] + 1.0 |PTS-I-pi-phospho-L-histidines| --> 1.0 alpha-maltose_6'-
phosphate + 1.0 |PTS-I-Histidines|

maltose-degr_3.2.1.122_MALTOSE-6-PHOSPHATE-GLUCOSIDASE-RXN           : 1.0 H2O + 1.0 alpha-maltose_6'-phosphate --> 1.0 D-glucose + 1.0 D-glucose_6-phosphate  #
source: MetaCyc, BRENDA, KEGG (queried 3.2.1.122)
#####################################################################################################################################################################
#sucrose degradation I
sucrose_exchange                                                     : 1.0 sucrose[ex] <=>
```

```
##CDIF630erm_00597
sucrose_transport                                                                       : 1.0 |PTS-I-pi-phospho-L-histidines| + 1.0 sucrose[ex] --> 1.0 sucrose_6-phosphate + 1.0 |
PTS-I-Histidines|

sucrose-degradation-I/1_3.2.1.B3_BR49793                                                : 1.0 sucrose_6-phosphate + 1.0 H2O --> 1.0 beta-D-fructofuranose + 1.0 alpha-D-glucose_6-
phosphate  # source: BRENDA, KEGG (queried 3.2.1.B3) // other EC numbers for this reaction: 3.2.1.26
sucrose-degradation-I/2a_2.7.1.4_FRUCTOKINASE-RXN                                       : 1.0 ATP + 1.0 beta-D-fructofuranose --> 1.0 ADP + 1.0 beta-D-fructofuranose_6-phosphate +
1.0 H+  # source: MetaCyc, KEGG (queried 2.7.1.4) // other EC numbers for this reaction: 2.7.1.1
sucrose-degradation-I/2b_5.3.1.5_BS1055                                                 : 1.0 D-glucose <-- 1.0 beta-D-fructofuranose  # source: BRENDA, KEGG (queried 5.3.1.5)

glucose-iso_5.3.1.9_R02740                                                              : 1.0 alpha-D-glucose_6-phosphate <=> 1.0 beta-D-fructofuranose_6-phosphate  # source: KEGG
(queried 5.3.1.9)
glucose-iso_5.1.3.15_5.3.1.9_spontaneous_GLUCOSE-6-PHOSPHATE-1-EPIMERASE-RXN            : 1.0 alpha-D-glucose_6-phosphate <=> 1.0 beta-D-glucose_6-phosphate  # source: MetaCyc,
BRENDA, KEGG (queried 5.1.3.15, 5.3.1.9)
##################################################################################################################################################################################
#D-tagatose degradation
D-tagatose_exchange                                                                     : 1.0 D-tagatose[ex] <=>
##CDIF630erm_03358
D-tagatose_transport                                                                    : 1.0 D-tagatose[ex] + 1.0 |PTS-I-pi-phospho-L-histidines| --> 1.0 |PTS-I-Histidines| + 1.0 D-
tagatose_1-phosphate
#CDIF630erm_03359
D-tagatose-degr/1_manual_2.7.1.-_HD-tag                                                 : 1.0 D-tagatose_1-phosphate + 1.0 ATP --> 1.0 H+ + 1.0 ADP + 1.0 D-tagatofuranose_1,6-
bisphosphate
D-tagatose-degr/_4.1.2.40_TAGAALDOL-RXN                                                 : 1.0 D-tagatofuranose_1,6-bisphosphate <=> 1.0 D-glyceraldehyde_3-phosphate + 1.0
glycerone_phosphate  # source: MetaCyc, BRENDA, KEGG (queried 4.1.2.40)
##################################################################################################################################################################################
#fructose degr
D-fructofuranose_exchange                                                               : 1.0 beta-D-fructofuranose[ex] <=>
##CDIF630erm_02503
fructose_transport_2.7.1.202                                                            : 1.0 |PTS-I-pi-phospho-L-histidines| + 1.0 beta-D-fructofuranose[ex] --> 1.0 beta-D-
fructofuranose_1-phosphate + 1.0 |PTS-I-Histidines|
fructose-degradation_2.7.1.56_1PFRUCTPHOSN-RXN                                          : 1.0 beta-D-fructofuranose_1-phosphate + 1.0 ATP --> 1.0 ADP + 1.0 fructose_1,6-bisphosphate
+ 1.0 H+  # source: MetaCyc, BRENDA (queried 2.7.1.56)
##################################################################################################################################################################################
#alternative 1-deoxy-D-xylulose_5-phosphate biosynthsis
1-deoxy-D-xylulose_5-phosphate-biosyn/1_4.1.2.13_RXN-8631                               : 1.0 beta-D-fructofuranose_1-phosphate --> 1.0 D-glyceraldehyde + 1.0 glycerone_phosphate  #
source: MetaCyc, BRENDA (queried 4.1.2.13)
1-deoxy-D-xylulose_5-phosphate-biosyn/2a_1.2.1.3_BS426217                               : 1.0 D-glyceraldehyde + 1.0 NAD+ + 1.0 H2O <=> 1.0 NADH + 1.0 D-glycerate + 1.0 H+  # source:
BRENDA, KEGG (queried 1.2.1.3)
1-deoxy-D-xylulose_5-phosphate-biosyn/2b_2.2.1.7_BS260855                               : 1.0 D-glyceraldehyde + 1.0 pyruvate --> 1.0 CO2 + 1.0 1-deoxy-D-xylulose  # source: BRENDA
(queried 2.2.1.7)
1-deoxy-D-xylulose_5-phosphate-biosyn/3_2.7.1.17_RXN0-382                               : 1.0 1-deoxy-D-xylulose + 1.0 ATP --> 1.0 ADP + 1.0 1-deoxy-D-xylulose_5-phosphate + 1.0 H+
# source: MetaCyc, BRENDA (queried 2.7.1.17) // other EC numbers for this reaction: 2.7.1
##################################################################################################################################################################################
#cellobiose degr C
D-cellobiose_exchange                                                                   : 1.0 beta-D-cellobiose[ex] <=>
##CDIF630erm_03148; CDIF630erm_03151; CDIF630erm_03151
cellobiose_transport_2.7.1.205                                                          : 1.0 beta-D-cellobiose[ex] + 1.0 |PTS-I-pi-phospho-L-histidines| --> 1.0 6-phospho-beta-D-
glucosyl-(1,4)-D-glucose + 1.0 |PTS-I-Histidines|
cellobiose-degr_3.2.1.86_6-PHOSPHO-BETA-GLUCOSIDASE-RXN                                 : 1.0 6-phospho-beta-D-glucosyl-(1,4)-D-glucose + 1.0 H2O --> 1.0 D-glucose + 1.0 D-glucose_6-
phosphate  # source: MetaCyc, BRENDA, KEGG (queried 3.2.1.86)
##################################################################################################################################################################################
#chitobiose degradation
chitobiose_exchange                                                                     : 1.0 N,N'-diacetylchitobiose[ex] <=>
##CDIF630erm_03148; CDIF630erm_03151; CDIF630erm_03151
chitobiose_transport_2.7.1.196                                                          : 1.0 N,N'-diacetylchitobiose[ex] + 1.0 |PTS-I-pi-phospho-L-histidines| --> 1.0 |PTS-I-
Histidines| + 1.0 N,N'-diacetylchitobiose_6'-phosphate  # source: MetaCyc (queried 2.7.1.69)

chitobiose-degradation_3.5.1.105_RXN0-7001                                              : 1.0 H2O + 1.0 N,N'-diacetylchitobiose_6'-phosphate --> 1.0 acetate + 1.0 N-
monoacetylchitobiose_6'-phosphate  # source: MetaCyc (queried 3.5.1.105)
chitobiose-degradation_3.2.1.86_RXN0-7002                                               : 1.0 H2O + 1.0 N-monoacetylchitobiose_6'-phosphate --> 1.0 N-acetyl-D-glucosamine_6-phosphate
+ 1.0 D-glucosamine  # source: MetaCyc (queried 3.2.1.86)
#assumed to be side reaction of 2.7.1.59
chitobiose-degradation_2.7.1.59_GLUCOSAMINE-KINASE-RXN                                  : 1.0 D-glucosamine + 1.0 ATP --> 1.0 D-glucosamine_6-phosphate + 1.0 ADP + 1.0 H+  # source:
```

MetaCyc, BRENDA, KEGG (queried 2.7.1.8) // other EC numbers for this reaction: 2.7.1.1
##
#trehalose degr I
trehalose_exchange : 1.0 alpha,alpha-trehalose[ex] <=>
#CDIF630erm_03371-CDIF630erm_03372?
trehalose_transport : 1.0 |PTS-I-pi-phospho-L-histidines| + 1.0 alpha,alpha-trehalose[ex] --> 1.0 |PTS-I-Histidines| + 1.0 alpha,alpha-trehalose_6-phosphate

trehalose-degr-I_3.2.1.122_3.2.1.93_TRE6PHYDRO-RXN : 1.0 alpha,alpha-trehalose_6-phosphate + 1.0 H2O --> 1.0 D-glucose_6-phosphate + 1.0 D-glucose # source: MetaCyc, BRENDA, KEGG (queried 3.2.1.122, 3.2.1.93)
##
#sugars polymers

cyclodextrin_exchange : 1.0 |Cyclodextrins|[ex] <=>
starch_exchange : 1.0 |Starch|[ex] <=>
maltodextrin_exchange : 1.0 |Maltodextrin|[ex] <=>
pullulan_exchange : 1.0 |Pullulans|[ex] <=>
maltotriose_exchange : 1.0 maltotriose[ex] <=>

cyclodextrin-degr_3.2.1.54_CYCLOMALTODEXTRINASE-RXN : 1.0 H2O + 1.0 |Cyclodextrins|[ex] --> 1.0 |Maltodextrins|[ex] # source: MetaCyc, BRENDA, KEGG (queried 3.2.1.54) // other EC numbers for this reaction: 2.4.1.19
starch-degr_manual_2.4.1.18_3.2.1.41_3.2.1.1_HD-starch : 1.0 |Starch|[ex] + 1.0 H2O[ex] --> 1.0 |Maltodextrins|[ex]
maltodextrin-degr_3.2.1.133_3.2.1.1_RXN-12188 : 1.0 |Maltodextrins|[ex] + 1.0 H2O[ex] --> 1.0 D-glucose[ex] + 1.0 alpha,alpha-trehalose[ex] # source: MetaCyc, BRENDA (queried 3.2.1.133, 3.2.1.1) // there are 4 more EC numbers for this reaction
pullulan-degr_3.2.1.54_3.2.1.41_RXN-12302 : 1.0 |Pullulans|[ex] + 1.0 H2O[ex] --> 2.0 maltotriose[ex] # source: MetaCyc, BRENDA (queried 3.2.1.54, 3.2.1.41) // other EC numbers for this reaction: 3.2.1.135
maltotriose-degr_modnames_3.2.1.10_R06199 : 2.0 H2O + 1.0 maltotriose[ex] --> 3.0 D-glucose[ex] # source: KEGG (queried 3.2.1.10) // other EC numbers for this reaction: 3.2.1.3
##
#N-acetylneuraminate and N-acetylmannosamine degradation
N-acetylneuraminate_exchange : 1.0 N-acetylneuraminate[ex] <=>
##CDIF630erm_02472
N-acetylneuraminate_transport : 1.0 N-acetyl-beta-neuraminate + 1.0 Na+ <=> 1.0 N-acetylneuraminate[ex] + 1.0 Na+[ex]

N-acetylneur-degr/1_4.1.3.3_ACNEULY-RXN : 1.0 N-acetyl-beta-neuraminate <=> 1.0 |N-acetyl-D-mannosamine| + 1.0 pyruvate # source: MetaCyc, BRENDA, KEGG (queried 4.1.3.3)
N-acetylneur-degr/2_2.7.1.59_NANK-RXN : 1.0 ATP + 1.0 |N-acetyl-D-mannosamine| --> 1.0 ADP + 1.0 N-acetyl-D-mannosamine_6-phosphate + 1.0 H+ # source: MetaCyc, BRENDA, KEGG (queried 2.7.1.59) // other EC numbers for this reaction: 2.7.1.60
N-acetylneur-degr/3_5.1.3.9_NANE-RXN : 1.0 N-acetyl-D-mannosamine_6-phosphate <=> 1.0 N-acetyl-D-glucosamine_6-phosphate # source: MetaCyc, BRENDA, KEGG (queried 5.1.3.9)
##
#D-galactosamine and N-acetyl-D-galactosamine degradation
N-acetyl-D-galactosamine_exchange : 1.0 |N-acetyl-D-galactosamine|[ex]<=>
D-galactosamine_exchange : 1.0 D-galactosamine[ex] <=>
##CDIF630erm_00408-CDIF630erm_00413; CDIF630erm_01218, CDIF630erm_01220-CDIF630erm_01222; CDIF630erm_03576-CDIF630erm_03579?
N-acetyl-D-galactosamine_transport : 1.0 |PTS-I-pi-phospho-L-histidines| + 1.0 |N-acetyl-D-galactosamine| --> 1.0 |PTS-I-Histidines| + 1.0 N-acetyl-D-galactosamine_6-phosphate
D-galactosamine_transport : 1.0 D-Galactosamine[ex] + 1.0 |PTS-I-pi-phospho-L-histidines| --> 1.0 D-galactosamine_6-phosphate + 1.0 |PTS-I-Histidines| # source: MetaCyc (queried 2.7.1.69)

(N-acetyl-)D-galactosamine-degr/1_3.5.1.25_3.5.1.80-RXN : 1.0 N-acetyl-D-galactosamine_6-phosphate + 1.0 H2O --> 1.0 acetate + 1.0 D-galactosamine_6-phosphate # source: MetaCyc, BRENDA, KEGG (queried 3.5.1.25)
(N-acetyl-)D-galactosamine-degr/2_5.3.1.-_RXN-13548 : 1.0 D-galactosamine_6-phosphate + 1.0 H2O --> 1.0 ammonium + 1.0 D-tagatofuranose_6-phosphate # source: MetaCyc, KEGG (queried 5.3.1.-) // other EC numbers for this reaction: 5.3.1, 3.5.99
(N-acetyl-)D-galactosamine-degr/3_2.7.1.11_2.7.1.144_2.7.1.56_TAGAKIN-RXN : 1.0 D-tagatofuranose_6-phosphate + 1.0 ATP --> 1.0 ADP + 1.0 H+ + 1.0 D-tagatofuranose_1,6-bisphosphate # source: MetaCyc, BRENDA, KEGG (queried 2.7.1.11, 2.7.1.144, 2.7.1.56)
##
#D-glucosaminate-degradation
D-glucosaminate_exchange : 1.0 D-glucosaminate[ex] <=>
##CDIF630erm_00408-CDIF630erm_00413; CDIF630erm_01218, CDIF630erm_01220-CDIF630erm_01222; CDIF630erm_03576-CDIF630erm_03579?
D-glucosaminate_transport : 1.0 |PTS-I-pi-phospho-L-histidines| + 1.0 D-glucosaminate[ex] --> 1.0 D-glucosaminate_6-phosphate + 1.0 |PTS-I-Histidines| # source: MetaCyc (queried 2.7.1.69)

D-glucosaminate-degr/1_4.3.1.29_RXN-14730 : 1.0 D-glucosaminate_6-phosphate --> 1.0 ammonium + 1.0 2-dehydro-3-deoxy-D-gluconate_6-

```
phosphate  # source: MetaCyc, BRENDA, KEGG (queried 4.3.1.29)
D-glucosaminate-degr/2_2.7.1.45_DEOXYGLUCONOKIN-RXN                                  : 1.0 2-dehydro-3-deoxy-D-gluconate + 1.0 ATP --> 1.0 ADP + 1.0 2-dehydro-3-deoxy-D-
gluconate_6-phosphate + 1.0 H+  # source: MetaCyc, BRENDA, KEGG (queried 2.7.1.45) // other EC numbers for this reaction: 2.7.1.178
D-glucosaminate-degr/3_4.1.2.14_KDPGALDOL-RXN                                        : 1.0 2-dehydro-3-deoxy-D-gluconate_6-phosphate --> 1.0 pyruvate + 1.0 D-glyceraldehyde_3-
phosphate  # source: MetaCyc, BRENDA, KEGG (queried 4.1.2.14) // other EC numbers for this reaction: 4.1.2.20, 4.1.2.55
###########################################################################################################################################################################
#2-O-alpha-mannosyl-D-glycerate degradation C
2-O-alpha-mannosyl-D-glycerate_exchange                                              : 1.0 2-O-alpha-mannosyl-D-glycerate[ex] <=>
##CDIF630erm_03369; CDIF630erm_02819-CDIF630erm_02821
2-O-alpha-mannosyl-D-glycerate_transport_2.7.1.195                                   : 1.0 2-O-alpha-mannosyl-D-glycerate[ex] + 1.0 |PTS-I-pi-phospho-L-histidines| --> 1.0 2-O-(6-
phospho-alpha-D-mannosyl)-D-glycerate + 1.0 |PTS-I-Histidines|  # source: MetaCyc (queried 2.7.1.69)

2-O-alpha-mannosyl-D-glycerate-degr_3.2.1.170_RXN0-5216                              : 1.0 H2O + 1.0 2-O-(6-phospho-alpha-D-mannosyl)-D-glycerate --> 1.0 D-glycerate + 1.0 D-
mannopyranose_6-phosphate  # source: MetaCyc (queried 3.2.1.170)
###########################################################################################################################################################################
#altronate and 2-dehydro-3-deoxy-D-gluconate degradation
D-altronate_exchange                                                                 : 1.0 D-altronate <=>
2-dehydro-3-deoxy-D-gluconate_exchange                                               : 1.0 2-dehydro-3-deoxy-D-gluconate <=>
##CDIF630erm_03138
2-dehydro-3-deoxy-D-gluconate_transport                                              : 1.0 2-dehydro-3-deoxy-D-gluconate + 1.0 Na+ <=> 1.0 2-dehydro-3-deoxy-D-gluconate[ex] + 1.0
Na+[ex]
D-altronate_transport                                                                : 1.0 D-altronate + 1.0 Na+ <=> 1.0 D-altronate[ex] + 1.0 Na+[ex]
D-altronate-degr_4.2.1.7_ALTRODEHYDRAT-RXN                                           : 1.0 D-altronate --> 1.0 H2O + 1.0 2-dehydro-3-deoxy-D-gluconate  # source: MetaCyc, BRENDA,
KEGG (queried 4.2.1.7)
###########################################################################################################################################################################
riboflavin_exchange                                                         : 1.0 riboflavin[ex] <=>
##CDIF630erm_00266
riboflavin_transport                                                        : 1.0 H2O + 1.0 ATP + 1.0 riboflavin[ex] --> 1.0 H+ + 1.0 phosphate + 1.0 ADP + 1.0 riboflavin
###########################################################################################################################################################################
#adenosylcobalamin salvage from cobinamide
cobinamide_exchange                                                         : 1.0 cobinamide[ex] <=>
coenzyme-B12_exchange                                                       : 1.0 adenosylcobalamin[ex] <=>

coenzyme-B12_transport_3.6.3.33                                             : 1.0 ATP + 1.0 adenosylcobalamin[ex] + 1.0 H2O --> 1.0 ADP + 1.0 phosphate + 1.0 adenosylcobalamin +
1.0 H+ # source: BRENDA (queried 3.6.3.33)
cobinamide_transport_3.6.3.33                                               : 1.0 ATP + 1.0 cobinamide[ex] + 1.0 H2O --> 1.0 ADP + 1.0 phosphate + 1.0 cobinamide + 1.0 H+ #
source: BRENDA (queried 3.6.3.33)

adenosylcobalamin-salvage-from-cobinamide/1_2.5.1.17_BTUR2-RXN              : 1.0 ATP + 1.0 cobinamide --> 1.0 adenosylcobinamide + 1.0 PPPi  # source: MetaCyc, BRENDA, KEGG
(queried 2.5.1.17)
adenosylcobalamin-salvage-from-cobinamide/2_2.7.1.156_COBINAMIDEKIN-RXN     : 1.0 adenosylcobinamide + 1.0 ATP --> 1.0 ADP + 1.0 adenosyl-cobinamide_phosphate + 1.0 H+  # source:
MetaCyc, BRENDA, KEGG (queried 2.7.1.156)
###########################################################################################################################################################################
#dethiobiotin
dethiobiotin_exchange                                                  : 1.0 dethiobiotin[ex] <=>
##CDIF630erm_03256
dethiobiotin_transport                                                 : 1.0 ATP + 1.0 dethiobiotin[ex] + 1.0 H2O --> 1.0 ADP + 1.0 phosphate + 1.0 dethiobiotin + 1.0 H+ # source:
BRENDA (queried 3.6.3.33)

biotin-biosynthesis_2.8.1.7_RXN-12588                                  : 1.0 |Unsulfurated-Sulfur-Acceptors| + 1.0 L-cysteine --> 1.0 |Sulfurated-Sulfur-Acceptors| + 1.0 L-alanine
# source: MetaCyc (queried 2.8.1.7)
biotin-biosynthesis_modnames_2.8.1.6_2.8.1.6-RXN                       : 1.0 |Sulfurated-Sulfur-Acceptors| + 2.0 |Reduced-Ferredoxins| + 2.0 S-adenosyl-L-methionine + 1.0
dethiobiotin --> 2.0 L-methionine + 2.0 5'-deoxyadenosine + 1.0 biotin + 1.0 |Unsulfurated-Sulfur-Acceptors| + 2.0 |Oxidized-Ferredoxins|  # source: MetaCyc (queried 2.8.1.6)
###########################################################################################################################################################################
#formylaminopyrimidine
formylaminopyrimidine_exchange                                         : 1.0 formylaminopyrimidine[ex] <=>
formylaminopyrimidine_transport                                        : 1.0 ATP + 1.0 formylaminopyrimidine[ex] + 1.0 H2O --> 1.0 ADP + 1.0 phosphate + 1.0 formylaminopyrimidine
+ 1.0 H+ # source: BRENDA (queried 3.6.3.33)

base-degraded-thiamine-salvage_3.5.1.-_RXN-12612                       : 1.0 formylaminopyrimidine + 1.0 H2O --> 1.0 aminomethylpyrimidine + 1.0 formate  # source: MetaCyc
(queried 3.5.1.-) // other EC numbers for this reaction: 3.5.1
base-degraded-thiamine-salvage_3.5.99.2_RXN-12613                      : 1.0 H2O + 1.0 aminomethylpyrimidine --> 1.0 4-amino-2-methyl-5-pyrimidinemethanol + 1.0 ammonium  #
source: MetaCyc, BRENDA, KEGG (queried 3.5.99.2)
###########################################################################################################################################################################
```

```
#sulfolactate
sulfolactate_exchange                                                        : 1.0 (2R)-3-sulfolactate <=>
##CDIF630erm_03289
sulfolactate_transport                                                       : 1.0 (2R)-3-sulfolactate + 1.0 Na+ <=> 1.0 (2R)-3-sulfolactate[ex] + 1.0 Na+[ex]

sulfolactate-degr/1_4.4.1.24_RXN-11691                                       : 1.0 (2R)-3-sulfolactate --> 1.0 H+ + 1.0 pyruvate + 1.0 sulfite  # source: MetaCyc, BRENDA (queried
4.4.1.24)
sulfolactate-degr/2_spontaneous_RXN-8315                                     : 1.0 H+ + 1.0 sulfite <=> 1.0 hydrogen_sulfite  # source: MetaCyc
############################################################################################################################################################################
#cyclic_di-3',5'-guanylate
cGDP_2.7.7.65_RXN0-5359                                                      : 2.0 GTP --> 2.0 diphosphate + 1.0 cyclic_di-3',5'-guanylate  # source: MetaCyc, BRENDA, KEGG (queried
2.7.7.65)
cGDP_3.1.4.52_RXN0-4181                                                      : 1.0 H2O + 1.0 cyclic_di-3',5'-guanylate --> 1.0 H+ + 1.0 linear_dimeric_GMP  # source: MetaCyc, BRENDA,
KEGG (queried 3.1.4.52)
############################################################################################################################################################################


#Reactions without pathway
#############1.1.1.1#############
~no-pathway_1.1.1.1_RXN-6021                                                                                 : 1.0 NAD+ + 1.0 choline --> 1.0 NADH + 1.0
betaine_aldehyde + 1.0 H+  # source: MetaCyc, KEGG (queried 1.1.1.1) // other EC numbers for this reaction: 1.1.1
~no-pathway_1.1.1.1_RXN-12582                                                                                : 1.0 choline + 1.0 NADP+ <=> 1.0 betaine_aldehyde + 1.0
NADPH + 1.0 H+  # source: MetaCyc, KEGG (queried 1.1.1.1) // other EC numbers for this reaction: 1.1.1
#############1.1.1.22#############
~no-pathway_1.1.1.22_UGD-RXN                                                                                 : 2.0 NAD+ + 1.0 UDP-alpha-D-glucose + 1.0 H2O --> 1.0 UDP-
alpha-D-glucuronate + 3.0 H+ + 2.0 NADH  # source: MetaCyc, BRENDA, KEGG (queried 1.1.1.22)
#############1.2.1.3#############
~no-pathway_1.2.1.3_RXN-4142                                                                                 : 1.0 H2O + 1.0 NAD+ + 1.0 |Fatty-Aldehydes| --> 1.0 |
Fatty-Acids| + 2.0 H+ + 1.0 NADH  # source: MetaCyc, BRENDA (queried 1.2.1.3) // other EC numbers for this reaction: 1.2.1.48
~no-pathway_1.2.1.3_AMINOBUTDEHYDROG-RXN                                                                     : 1.0 NAD+ + 1.0 4-aminobutanal + 1.0 H2O --> 1.0 4-
aminobutanoate + 2.0 H+ + 1.0 NADH  # source: MetaCyc, BRENDA, KEGG (queried 1.2.1.3) // other EC numbers for this reaction: 1.2.1.19, 1.2.1
#############1.1.1.86#############
~no-pathway_1.1.1.86_R04440                                                                                  : 1.0 (2R)-2,3-dihydroxy-3-methylbutanoate + 1.0 NADP+ <=>
1.0 NADPH + 1.0 H+ + 1.0 3-hydroxy-3-methyl-2-oxobutanoate  # source: KEGG (queried 1.1.1.86)
#############1.5.1.49#############
~no-pathway_1.5.1.49_PYRROLINE-2-CARBOXYLATE-REDUCTASE-RXN                                                   : 1.0 L-proline + 1.0 NADP+ <-- 2.0 H+ + 1.0 1-pyrroline-2-
carboxylate + 1.0 NADPH  # source: MetaCyc, BRENDA, KEGG (queried 1.5.1.49) // other EC numbers for this reaction: 1.5.1.1, 1.5.1.21
~no-pathway_1.5.1.49_PYRROLINE-2-CARBOXYLATE-REDUCTASE-RXN_WOP                                               : 1.0 L-proline + 1.0 NAD+ <=> 2.0 H+ + 1.0 NADH + 1.0 1-
pyrroline-2-carboxylate  # source: MetaCyc, BRENDA, KEGG (queried 1.5.1.49) // other EC numbers for this reaction: 1.5.1.1
#############1.8.1.14#############
~no-pathway_1.8.1.14_COA-DISULFIDE-REDUCTASE-NADH-RXN                                                        : 1.0 NADP+ + 2.0 coenzyme_A <=> 1.0 CoA-disulfide + 1.0 H+
+ 1.0 NADPH  # source: MetaCyc, BRENDA, KEGG (queried 1.8.1.14)
#############2.1.1.79#############
~no-pathway_2.1.1.79_2.1.1.79-RXN                                                                            : 1.0 S-adenosyl-L-methionine + 1.0 |Phospholipid-Olefinic-
Fatty-Acids| --> 1.0 H+ + 1.0 S-adenosyl-L-homocysteine + 1.0 |Phospholipid-Cyclopropane-Fatty-Acids|  # source: MetaCyc (queried 2.1.1.79)
#############2.3.1.181#############
~no-pathway_2.3.1.181_RXN0-947                                                                               : 1.0 |Octanoyl-ACPs| + 1.0 |Lipoyl-Protein-L-Lysine| -->
1.0 |Octanoylated-domains| + 1.0 Acyl-carrier_protein + 1.0 H+  # source: MetaCyc (queried 2.3.1.181)
#############2.3.1.79#############
~no-pathway_2.3.1.79_MALTACETYLTRAN-RXN                                                                      : 1.0 acetyl-CoA + 1.0 alpha,alpha-trehalose <=> 1.0
coenzyme_A + 1.0 6-O-acetyl-alpha-D-glucopyranosyl-(1rarr4)-D-glucose  # source: MetaCyc, BRENDA, KEGG (queried 2.3.1.79)
#############2.5.1.17#############
~no-pathway_2.5.1.17_COBALADENOSYLTRANS-RXN                                                                  : 1.0 cob(I)alamin + 1.0 ATP --> 1.0 adenosylcobalamin +
1.0 PPPi  # source: MetaCyc, BRENDA, KEGG (queried 2.5.1.17)
#############2.5.1.49#############
~no-pathway_2.5.1.49_O-ACETYLHOMOSERINE-THIOL-LYASE-RXN                                                      : 1.0 methanethiol + 1.0 O-acetyl-L-homoserine --> 1.0
acetate + 1.0 L-methionine + 1.0 H+  # source: MetaCyc, BRENDA, KEGG (queried 2.5.1.49)
#############2.7.1.33#############
~no-pathway_2.7.1.33_PANTETHEINE-KINASE-RXN                                                                  : 1.0 pantetheine + 1.0 ATP --> 1.0 ADP + 1.0 4'-
phosphopantetheine + 1.0 H+  # source: MetaCyc, BRENDA, KEGG (queried 2.7.1.33) // other EC numbers for this reaction: 2.7.1.34
~no-pathway_2.7.1.33_R04391                                                                                  : 1.0 ATP + 1.0 N-[(R)-pantothenoyl]-L-cysteine --> 1.0 R-
4'-phosphopantothenoyl-L-cysteine + 1.0 ADP  # source: KEGG (queried 2.7.1.33)
#############2.7.1.49#############
~no-pathway_2.7.1.49_2.7.1.35_PYRAMKIN-RXN                                                                   : 1.0 pyridoxamine + 1.0 ATP --> 1.0 H+ + 1.0 ADP + 1.0
```

pyridoxamine_5'-phosphate # source: MetaCyc, BRENDA, KEGG (queried 2.7.1.49, 2.7.1.35)
~no-pathway_2.7.1.35_PYRIDOXKIN-RXN : 1.0 pyridoxal + 1.0 ATP --> 1.0 H+ + 1.0 ADP + 1.0 pyridoxal_5'-phosphate # source: MetaCyc, BRENDA, KEGG (queried 2.7.1.35)
#############2.7.1.66##############
~no-pathway_2.7.1.66_UNDECAPRENOL-KINASE-RXN : 1.0 di-trans,octa-cis-undecaprenol + 1.0 ATP --> 1.0 ADP + 1.0 di-trans,octa-cis-undecaprenyl_phosphate + 1.0 H+ # source: MetaCyc, BRENDA, KEGG (queried 2.7.1.66)
#############2.7.7.85#############
~no-pathway_2.7.7.85_RXN-14338 : 2.0 ATP <=> 1.0 cyclic_di-3',5'-adenylate + 2.0 diphosphate # source: MetaCyc, BRENDA, KEGG (queried 2.7.7.85)
#############2.7.8.40#############
~no-pathway_2.7.8.40_RXN-14571 : 1.0 di-trans,octa-cis-undecaprenyl_phosphate + 1.0 UDP-N-acetyl-alpha-D-galactosamine <=> 1.0 N-acetyl-alpha-D-galactosaminyl-diphospho-ditrans,octacis-undecaprenol + 1.0 UMP # source: MetaCyc, BRENDA, KEGG (queried 2.7.8.40) // other EC numbers for this reaction: 2.7.8.6
#############2.7.8.7##############
~no-pathway_2.7.8.7_HOLO-ACP-SYNTH-RXN : 1.0 |apo-ACP| + 1.0 coenzyme_A --> 1.0 adenosine_3',5'-bisphosphate + 1.0 Acyl-carrier_protein + 1.0 H+ # source: MetaCyc, KEGG (queried 2.7.8.7) // other EC numbers for this reaction: 2.7.8
#############3.1.3.18#############
photorespiration_3.1.3.18_GPH-RXN : 1.0 H2O + 1.0 2-phosphoglycolate --> 1.0 glycolate + 1.0 phosphate # source: MetaCyc, BRENDA, KEGG (queried 3.1.3.18)
#############3.1.4.46#############
~no-pathway_3.1.4.46_RXN-14073 : 1.0 glycerophosphoglycerol + 1.0 H2O --> 1.0 sn-glycerol_3-phosphate + 1.0 glycerol + 1.0 H+ # source: MetaCyc, BRENDA (queried 3.1.4.46)
#############3.2.1.10#############
~no-pathway_3.2.1.10_3.2.1.10-RXN : 1.0 beta-isomaltose + 1.0 H2O --> 2.0 D-glucose # source: MetaCyc (queried 3.2.1.10)
#############3.2.1.133#############
~no-pathway_3.2.1.133_3.2.1.133-RXN : 1.0 H2O + 1.0 |Starch| --> 1.0 alpha-maltose # source: MetaCyc (queried 3.2.1.133)
#############3.2.1.37#############
~no-pathway_3.2.1.37_3.2.1.37-RXN : 1.0 H2O + 1.0 |1-4-D-xylooligosaccharides| --> 1.0 beta-D-xylopyranose # source: MetaCyc (queried 3.2.1.37)
#############3.6.1.13#############
~no-pathway_3.6.1.13_RXN0-1441 : 1.0 H2O + 1.0 ADP-D-ribose --> 2.0 H+ + 1.0 AMP + 1.0 D-ribose_5-phosphate # source: MetaCyc, BRENDA, KEGG (queried 3.6.1.13) // other EC numbers for this reaction: 3.6.1, 3.6.1.53, 3.6.1.21
#############4.1.2.17#############
lactate-biosynthesis_fucose-degradation_4.1.2.17_FUCPALDOL-RXN : 1.0 L-fuculose_1-phosphate <=> 1.0 (S)-lactaldehyde + 1.0 glycerone_phosphate # source: MetaCyc, BRENDA, KEGG (queried 4.1.2.17)
#############4.1.3.16#############
~no-pathway_4.1.3.16_RXN-13990 : 1.0 (4S)-4-hydroxy-2-oxoglutarate <=> 1.0 glyoxylate + 1.0 pyruvate # source: MetaCyc, BRENDA, KEGG (queried 4.1.3.16) // other EC numbers for this reaction: 4.1.3.42
#############5.1.1.13#############
~no-pathway_5.1.1.13_ASPARTATE-RACEMASE-RXN : 1.0 L-aspartate <=> 1.0 D-aspartate # source: MetaCyc, BRENDA, KEGG (queried 5.1.1.13)
#############5.1.1.21#############
~no-pathway_5.1.1.21_RXN-16430 : 1.0 L-isoleucine <=> 1.0 D-allo-isoleucine # source: MetaCyc, BRENDA, KEGG (queried 5.1.1.21)
#############5.1.3.14#############
~metabolism-of-biosynthesis_5.1.3.14_UDPGLCNACEPIM-RXN : 1.0 UDP-N-acetyl-alpha-D-glucosamine <=> 1.0 UDP-N-acetyl-alpha-D-mannosamine # source: MetaCyc, BRENDA, KEGG (queried 5.1.3.14)
#############5.4.3.9#############
~no-pathway_5.4.3.9_RXN-13569 : 1.0 L-glutamate <=> 1.0 beta-glutamate # source: MetaCyc, BRENDA, KEGG (queried 5.4.3.9)
#############6.1.1.13#############
~no-pathway_6.1.1.13_6.1.1.13-RXN : 1.0 ATP + 1.0 Poly(ribitol_phosphate) + 1.0 D-alanine --> 1.0 H+ + 1.0 diphosphate + 1.0 O-D-Alanyl-poly(ribitol_phosphate) + 1.0 AMP # source: MetaCyc, BRENDA, KEGG (queried 6.1.1.13)
##
#Genric
#############1.1.3.15#############
~no-pathway_1.1.3.15_S-2-HYDROXY-ACID-OXIDASE-RXN : 1.0 oxygen + 1.0 |S-2-Hydroxyacids| --> 1.0 hydrogen_peroxide + 1.0 |2-Oxo-carboxylates| # source: MetaCyc, BRENDA, KEGG (queried 1.1.3.15)
#############1.11.1.15#############
~no-pathway_1.11.1.15_RXN0-5468 : 1.0 |Red-Thioredoxin| + 1.0 |Alkyl-Hydro-Peroxides| --> 1.0 |Alcohols| + 1.0 H2O + 1.0 |Ox-Thioredoxin| # source: MetaCyc (queried 1.11.1.15)
#############1.2.1.3#############

~no-pathway_1.2.1.3_ALDHDEHYDROG-RXN : 1.0 |Aldehydes| + 1.0 H2O + 1.0 NAD+ --> 1.0 NADH + 1.0 |Carboxylates| + 2.0 H+ # source: MetaCyc, BRENDA, KEGG (queried 1.2.1.3) // other EC numbers for this reaction: 1.2.1.5
############3.1.1.96############
~no-pathway_3.1.1.96_RXN-15041 : 1.0 |D-aminoacyl-tRNAs| + 1.0 H2O --> 1.0 |tRNAs| + 2.0 H+ + 1.0 |D-Amino-Acids| # source: MetaCyc (queried 3.1.1.96)
############3.5.1.3############
~no-pathway_3.5.1.3_OMEGA-AMIDASE-RXN : 1.0 DICARBOXYLIC-ACID-MONOAMIDES + 1.0 H2O --> 1.0 ammonium + 1.0 |dicarboxylate| # source: MetaCyc (queried 3.5.1.3)
############3.5.1.81############
~no-pathway_3.5.1.81_3.5.1.81-RXN : 1.0 N-Acyl-D-amino_acid + 1.0 H2O --> 1.0 |Carboxylates| + 1.0 |D-Amino-Acids| # source: MetaCyc, BRENDA, KEGG (queried 3.5.1.81)
############3.5.1.87############
~no-pathway_3.5.1.87_3.5.1.87-RXN : 1.0 H2O + 2.0 H+ + 1.0 |N-Carbamoyl-L-Amino-Acids| --> 1.0 ammonium + 1.0 |L-Amino-Acids| + 1.0 CO2 # source: MetaCyc, BRENDA, KEGG (queried 3.5.1.87)
############5.5.1.6############
~no-pathway_5.5.1.6_CHALCONE-ISOMERASE-RXN : 1.0 |Chalcones| <=> 1.0 flavanone # source: MetaCyc, BRENDA, KEGG (queried 5.5.1.6)
###
#References
#Aboulnaga, E.-H., Pinkenburg, O., Schiffels, J., El-Refai, A., Buckel, W. & Selmer, T. (2013). Butyrate production in Escherichia coli: Exploitation of an oxygen tolerant bifurcating butyryl-CoA dehydrogenase/electron transferring flavoprotein complex from Clostridium difficile. J Bacteriol JB.00321-13.
#Brown, A. M., Hoopes, S. L., White, R. H. & Sarisky, C. A. (2011). Purine biosynthesis in archaea: variations on a theme. Biology Direct 6, 63.
#Burguière, P., Auger, S., Hullo, M.-F., Danchin, A. & Martin-Verstraete, I. (2004). Three different systems participate in L-cystine uptake in Bacillus subtilis. J Bacteriol 186, 4875–4884.
#Calusinska, M., Happe, T., Joris, B. & Wilmotte, A. (2010). The surprising diversity of clostridial hydrogenases: a comparative genomic perspective. Microbiology 156, 1575–1588.
#Colby, G. D. & Chen, J. S. (1992). Purification and properties of 3-hydroxybutyryl-coenzyme A dehydrogenase from Clostridium beijerinckii (‘Clostridium butylicum’) NRRL B593. Appl Environ Microbiol 58, 3297–3302.
#Curthoys, N. P. & Rabinowitz, J. C. (1972). Formyltetrahydrofolate synthetase. Binding of folate substrates and kinetics of the reverse reaction. J Biol Chem 247, 1965–1971.
#Curthoys, N. P., Scott, J. M. & Rabinowitz, J. C. (1972). Folate Coenzymes of Clostridium acidi-urici THE ISOLATION OF (l)-5,10-METHENYLTETRAHYDROPTEROYLTRIGLUTARMATE, ITS CONVERSION TO (l)-TETRAHYDROPTEROYLTRIGLUTAMATE AND (l)-10-[14C]FORMYLTETRAHYDROPTEROYLTRIGLUTAMATE, AND THE SYNTHESIS OF (l)-10-FORMYL-[6,7-3H2]TETRAHYDROPTEROYLTRIGLUTAMATE AND (l)-[6,7-3H2]TETRAHYDROPTEROYLTRIGLUTAMATE. J Biol Chem 247, 1959–1964.
#Elsden, S. R., Hilton, M. G., Parsley, K. R. & Self, R. (1980). The Lipid Fatty Acids of Proteolytic Clostridia. J Gen Microbiol 118, 115–123.
#Fahey, R. C., Brown, W. C., Adams, W. B. & Worsham, M. B. (1978). Occurrence of glutathione in bacteria. J Bacteriol 133, 1126–1129.
#Falany, C. N., Fortinberry, H., Leiter, E. H. & Barnes, S. (1997). Cloning, expression, and chromosomal localization of mouse liver bile acid CoA:amino acid N-acyltransferase. J Lipid Res 38, 1139–1148.
#Faulds-Pain, A., Twine, S. M., Vinogradov, E., Strong, P. C. R., Dell, A., Buckley, A. M., Douce, G. R., Valiente, E., Logan, S. M. & Wren, B. W. (2014). The post-translational modification of the Clostridium difficile flagellin affects motility, cell surface properties and virulence. Molecular Microbiology 94, 272–289.
#Han, B., Gopalan, V. & Ezeji, T. C. (2011). Acetone production in solventogenic Clostridium species: new insights from non-enzymatic decarboxylation of acetoacetate. Appl Microbiol Biotechnol 91, 565–576.
#Hess, V., Schuchmann, K. & Müller, V. (2013). The Ferredoxin:NAD+ Oxidoreductase (Rnf) from the Acetogen Acetobacterium woodii Requires Na+ and Is Reversibly Coupled to the Membrane Potential. J Biol Chem 288, 31496–31502.
#Hugo, H. v, Schoberth, S., Madan, V. K. & Gottschalk, G. (1972). Coenzyme specificity of dehydrogenases and fermentation of pyruvate by clostridia. Archiv Mikrobiol 87, 189–202.
#Jiang, W., Hermolin, J. & Fillingame, R. H. (2001). The preferred stoichiometry of c subunits in the rotary motor sector of Escherichia coli ATP synthase is 10. PNAS 98, 4966–4971.
#Kleiner, D. (1985). Bacterial ammonium transport. FEMS Microbiology Letters 32, 87–100.
#Köpke, M., Held, C., Hujer, S., Liesegang, H., Wiezer, A., Wollherr, A., Ehrenreich, A., Liebl, W., Gottschalk, G. & Dürre, P. (2010). Clostridium ljungdahlii represents a microbial production platform based on syngas. PNAS 107, 13087–13092.
#Meyer, E., Leonard, N. J., Bhat, B., Stubbe, J. & Smith, J. M. (1992). Purification and characterization of the purE, purK, and purC gene products: identification of a previously unrecognized energy requirement in the purine biosynthetic pathway. Biochemistry 31, 5022–5032.
#Midtvedt, T. & Norman, A. (1967). Bile Acid Transformations by Microbial Strains Belonging to Genera Found in Intestinal Contents. Acta Pathologica Microbiologica Scandinavica 71, 629–638.
#Mock, J., Zheng, Y., Mueller, A. P., Ly, S., Tran, L., Segovia, S., Nagaraju, S., Köpke, M., Dürre, P. & Thauer, R. K. (2015). Energy Conservation Associated with Ethanol Formation from H2 and CO2 in Clostridium autoethanogenum Involving Electron Bifurcation. J Bacteriol 197, 2965–2980.
#Moore, S. J., Biedendieck, R., Lawrence, A. D., Deery, E., Howard, M. J., Rigby, S. E. J. & Warren, M. J. (2013). Characterization of the Enzyme CbiH60 Involved in Anaerobic Ring Contraction of the Cobalamin (Vitamin B12) Biosynthetic Pathway. J Biol Chem 288, 297–305.
#Murata, T., Yamato, I., Kakinuma, Y., Leslie, A. G. W. & Walker, J. E. (2005). Structure of the rotor of the V-Type Na+-ATPase from Enterococcus hirae. Science 308, 654–659.
#Musfeldt, M. & Schönheit, P. (2002). Novel Type of ADP-Forming Acetyl Coenzyme A Synthetase in Hyperthermophilic Archaea: Heterologous Expression and Characterization of Isoenzymes from the Sulfate Reducer Archaeoglobus fulgidus and the Methanogen Methanococcus jannaschii. J Bacteriol 184, 636–644.
#Nagarajan, H., Sahin, M., Nogales, J., Latif, H., Lovley, D. R., Ebrahim, A. & Zengler, K. (2013). Characterizing acetogenic metabolism using a genome-scale metabolic reconstruction of Clostridium ljungdahlii. Microb Cell Fact 12, 118.
#Patrick, W. M., Quandt, E. M., Swartzlander, D. B. & Matsumura, I. (2007). Multicopy Suppression Underpins Metabolic Evolvability. Mol Biol Evol 24, 2716–2722.
#Peltier, J., Courtin, P., Meouche, I. E., Lemée, L., Chapot-Chartier, M.-P. & Pons, J.-L. (2011). Clostridium difficile Has an Original Peptidoglycan Structure with a High Level of N-Acetylglucosamine Deacetylation and Mainly 3-3 Cross-links. J Biol Chem 286, 29053–29062.
#Pitts, A. C., Tuck, L. R., Faulds-Pain, A., Lewis, R. J. & Marles-Wright, J. (2012). Structural Insight into the Clostridium difficile Ethanolamine Utilisation Microcompartment.

Scenario S1: **Exemplary scenario file (MDM, 3 h) for the metabolic model *i*HD922 of *C. difficile* 630Δ*erm***

```
LB    NGAM                                                        2
#to inhibit
fatty-acid-I/2_2.3.1.39_MALONYL-COA-ACP-TRANSACYL-RXN - 7.123 fatty-acid-
I/3a_manual_HD-acet = 0
acetyl-CoA-to-butyrate-II/1_2.3.1.9_ACETYL-COA-ACETYLTRANSFER-RXN - 20 hexanoate-
ferm/1_2.3.1.9_RXN-12565    >   0
acetyl-CoA-to-butyrate-II/1_2.3.1.9_ACETYL-COA-ACETYLTRANSFER-RXN - 3 pentanoate-
ferm/1_2.3.1.9_RXN-12561    >   0
#####Substrates/Products in MDM/CDMM#####
LB    glycine_exchange                                            0
LB    aspartate_exchange                                          0
LB    glutamate_exchange                                          0
LB    serine_exchange                                             0
LB    threonine_exchange                                          0
LB    tyrosine_exchange                                           0
LB    phenylalanine_exchange                                      0
LB    arginine_exchange                                           0
LB    histidine_exchange                                          0
LB    lysine_exchange                                             0
#Gases
LB    H2_exchange                                                 0
LB    CO_exchange                                                 0
UB    CO_exchange                                                 0
LB    methanethiol_exchange                                       0
LB    sulfite_exchange                                            0
#Alcohols
LB    ethanol_exchange                                            0
LB    propanol_exchange                                           0
LB    butanol_exchange                                            0
LB    isobutanol_exchange                                         0
#Acids
LB    formate_exchange                                            0
LB    acetate_exchange                                            0
LB    propanoate_exchange                                         0
LB    butanoate_exchange                                          0
LB    valerate_exchange                                           0
LB    hexanoate_exchange                                          0
LB    (R)-3-hydroxybutanoate_exchange                             0
LB    isocaproate_exchange                                        0
LB    3-phenylpropanoate_exchange                                 0
LB    3-(4-hydroxyphenyl)propanoate_exchange                      0
LB    (S)-lactate_exchange                                        0
LB    (R)-lactate_exchange                                        0
#Other
LB    S-methyl-5-thio-D-ribose_exchange                           0
LB    beta-alanine_exchange                                       0
LB    2-aminobutanoate_exchange                                   0
#####Other Substrates/Products#####
#bases
LB    cytosine_exchange                                           0
LB    adenine_exchange                                            0
LB    guanine_exchange                                            0
LB    hypoxanthine_exchange                                       0
LB    uracil_exchange                                             0
LB    xanthine_exchange                                           0
LB    urate_exchange                                              0
LB    thymine_exchange                                            0
```

```
#amino acids and amines
LB    asparagine_exchange                                         0
LB    glutamine_exchange                                          0
LB    4-hydroxyproline_exchange                                   0
LB    cystathionine_exchange                                      0
LB    spermidine_exchange                                         0
LB    putrescine_exchange                                         0
LB    2,3-diaminopropanoate_exchange                              0
LB    hydroxylamine_exchange                                      0
LB    ethanolamine_exchange                                       0
LB    4-hydroxyphenylacetate_exchange                             0
#with phosporous
LB    sn-glycerol-3-phosphate_exchange                            0
LB    methylphosphonate_exchange                                  0
LB    2-aminoethylphosphonate_exchange                            0
LB    phosphonoacetate_exchange                                   0
#non-sugar carbohydrates
LB    succinate_exchange                                          0
LB    tartronate-semialdehyde_exchange                            0
LB    acetoin_exchange                                            0
LB    3-hydroxy-3-methylglutarate_exchange                        0
#tox
LB    hydroxyl-radical_exchange                                   0
LB    superoxide_exchange                                         0
LB    hydrogen_peroxide_exchange                                  0
LB    oxygen_exchange                                             0
LB    mathanol_exchange                                           0
LB    cyanide_exchange                                            0
LB    thiocyanate_exchange                                        0
#bile acids
LB    taurochenodeoxycholate_exchange                             0
LB    chenodeoxycholate_exchange                                  0
LB    taurocholate_exchange                                       0
LB    cholate_exchange                                            0
#sugars and sugar alcohols
LB    D-arabinose_exchange                                        0
LB    xylan_exchange                                              0
LB    xylose_exchange                                             0
LB    galactose_exchange                                          0
LB    xylitol_exchange                                            0
LB    arabitol_exchange                                           0
LB    ribitol_exchange                                            0
LB    D-sorbitol_exchange                                         0
LB    D-mannitol_exchange                                         0
LB    D-ribopyranose_exchange                                     0
LB    2'-deoxyribose_exchange                                     0
LB    maltose_exchange                                            0
LB    sucrose_exchange                                            0
LB    D-tagatose_exchange                                         0
LB    D-fructofuranose_exchange                                   0
LB    D-cellobiose_exchange                                       0
LB    chitobiose_exchange                                         0
LB    trehalose_exchange                                          0
LB    cyclodextrin_exchange                                       0
LB    starch_exchange                                             0
LB    maltodextrin_exchange                                       0
LB    pullulan_exchange                                           0
LB    maltotriose_exchange                                        0
LB    N-acetylneuraminate_exchange                                0
```

```
LB    N-acetyl-D-galactosamine_exchange                        0
LB    D-galactosamine_exchange                                 0
LB    D-glucosaminate_exchange                                 0
LB    2-O-alpha-mannosyl-D-glycerate_exchange                  0
LB    D-altronate_exchange                                     0
LB    2-dehydro-3-deoxy-D-gluconate_exchange                   0
#other
LB    riboflavin_exchange                                      0
LB    cobinamide_exchange                                      0
LB    coenzyme-B12_exchange                                    0
LB    dethiobiotin_exchange                                    0
LB    sulfolactate_exchange                                    0
LB    choline_exchange                                         0
LB    formylaminopyrimidine_exchange                           0
LB    D-Glucose_exchange                                       -47.3671043246
UB    D-Glucose_exchange                                       -47.3671043246
LB    cysteine_exchange                                        -73.4834483508
LB    methionine_exchange                                      -8.5664873159
LB    tryptophan_exchange                                      -1.0401670259
LB    isoleucine_exchange                                      -5.110423686
LB    leucine_exchange                                         -1.8333183353
LB    valine_exchange                                          -1.8581174684
LB    proline_exchange                                         -15.9071454768
LB    alanine_exchange                                         0
LB    Biomass                                                  0.4139957168
OBJ max exopolysaccharide_exchange
```

Table S1: **Isotope labeling of glycolysis and glycolysis-related metabolites.** Intracellular metabolites analyzed by GC/MS of *C. difficile* 630Δ*erm* cultivated in MDM with full ^{13}C-labeled glucose and harvested at OD 0.22.

Metabolite	Derivative	Fragment	C/Si	Backbone	M+0	M+1	M+2	M+3	M+4	M+5
Glucose	1MeOX/5TMS	319	C13Si3	C2-C5	0.0	0.0	0.0	0.0	100.0	0.0
		205	C8Si2	C5+C6	0.0	0.0	100.0	0.0	0.0	0.0
		364	C14Si3	C1-C4	0.0	0.0	0.0	0.0	100.0	0.0
Glucose 6-phosphate	1MeOX/6TMS	387	C13Si3	C3-C6	36.9±2.2	0.0	0.0	0.0	63.1±4.9	0.0
		471	C16Si4	C2-C6	36.8±3.6	0.0	0.0	0.0	0.0	62.8±3.1
		160	C6Si1	C1+C2	35.9±3.7	0.0	64.1±2.0	0.0	0.0	0.0
Glycerate 3-phosphate	4TMS	387	C12Si3	C1-C3	89.5±7.5	0.0	0.0	10.5±1.5	0.0	0.0
		459	C14Si4	C1-C3	90.2±0.3	0.0	0.0	9.8±0.2	0.0	0.0
Pyruvate	2TMS	217	C8Si2	C1-C3	91.4±3.3	0.0	0.0	8.6±1.3	0.0	0.0
	1MeOX/1TMS	174	C6Si	C1-C3	91.0±3.0	0.0	0.0	9.0±0.6	0.0	0.0
		189	C7Si	C1-C3	90.9±3.4	0.0	0.0	9.1±0.4	0.0	0.0
Alanine	2TMS	116	C5Si	C2+C3	90.1±2.2	0.0	9.9±0.3	0.0	0.0	0.0
		190	C7Si2	2xC	90.1±0.1	0.0	8.9±0.0	0.0	0.0	0.0
Lactate	2TMS	219	C8Si2	C1-C3	91.2±6.3	0.0	0.0	8.8±1.5	0.0	0.0
		117	C5Si	C2+C3	93.0±1.9	0.0	7.0±1.1	0.0	0.0	0.0
Cysteine	3TMS	232	C9Si2	C1-C3	100.0	0.0	0.0	0.0	0.0	0.0

Table S2: **Biomass compostion used for the model *i*HD992**

Component			Cell composition			
Protein	0,508	0,508	±0.014	0,5284	0,4	0,55
RNA	0,091	0,091	±0.001	0,0655	0,12	0,205
DNA	0,043	0,043	±0.002	0,0260	0,03	0,031
Lipid	0,060	0,084	±0.002	0,076	0,04291	0,091
Lipoteichoic acid	0,024			0,0304	0,02709	0,034
Peptidoglycan	0,145			0,1009	0,1344	0,025
Polysaccharide II	0,097			0,1234	0,1056	
Glycogen	0,005			0,0000		0,025
Ions	0,022			0,0321	0,0944	0,01
Metabolites	0,005			0,0173	0,0456	0,029
Sum	1,000	0,726		1,0000	1,00000	1,000

Clostridium difficile NCTC 11223 (Poxton and Cartmill, 1982): Cell wall polysaccharide II 40% by weight of SDS-purified walls
Clostridium difficile 630Δ*erm* (this work)
Bacillus subtilis, dilution rate of 0.1/h (Dauner and Sauer, 2001)
Staphylococcus aureus (Heinemann et al., 2005)
Escherichia coli (Neidhard et al., 1990)

	Molecular weight [g/mol]
Water	18,015
Hydrogen	2,016
Glycerol-3-phosphate	170,06
Glycerol	92,094
Phosphate	95,979
Methane	16,043

Amino acid analysis of hydrolyzed cells

Amino acid	Mass [g]	standard deviation	Masses including assumptions [g]	Molecular weight [g/mol]	Molecular weight in protein [g/mol]	Demand per biomass [mmol]	Demand for peptidoglycan [mmol]	Demand for protein [g]
Glycine	0,0258760	0,0019797	0,0258760	75,07	57,05	0,34471	0,00655	0,01929
Alanine	0,0510480	0,0041868	0,0510480	89,09	71,08	0,57297	0,23278	0,02418
Valine	0,0263600	0,0021179	0,0263600	117,15	99,14	0,22501		0,02231
Leucine	0,0406434	0,0032087	0,0406434	131,17	113,16	0,30985		0,03506
Isoleucine	0,0246838	0,0020485	0,0246838	131,17	113,16	0,18818		0,02129
Serine	0,0234864	0,0017999	0,0234864	105,09	87,08	0,22349		0,01946
Threonine	0,0265723	0,0020727	0,0265723	119,12	101,11	0,22307		0,02255
Phenylalanine	0,0209894	0,0016538	0,0209894	165,19	147,18	0,12706		0,01870
Tyrosine	0,0183924	0,0011384	0,0183924	181,19	163,18	0,10151		0,01656
Tryptophan	not detected		0,0060981	204,23	186,22	0,02986		0,00556
Cysteine	not detected		0,0060981	121,15	103,14	0,05034		0,00519

Methionine	0,0051211	0,0002230	0,0051211	149,21	131,20	0,03432		0,00450
Lysine	0,0475186	0,0065515	0,0475186	147,20	129,19	0,32282		0,04170
Arginine	0,0230321	0,0026527	0,0230321	175,21	157,20	0,13145		0,02066
Histidine	0,0076227	0,0013264	0,0076227	155,16	137,15	0,04913		0,00674
Aspartate	0,0630830	0,0047067	0,0315415	132,10	114,09	0,23877		0,02724
Glutamate	0,1052600	0,0084886	0,0842080	146,12	128,11	0,57629	0,14451	0,05531
Asparagine	not detected		0,0315415	132,12	114,11	0,23873		0,02724
Glutamine	not detected		0,0210520	146,15	128,14	0,14404		0,01846
Proline	0,0211960	0,0024992	0,0211960	115,13	97,12	0,18411		0,01788
Selenocysteine	not detected		0,0000762	168,05	150,04	0,00045		0,00007
Diaminopimelate	0,0273905	0,0013085	0,0273905	190,20	172,19	0,14401	0,14401	0,00000
Sum	0,5582758		0,5705483					0,42997
Clostridium difficile 630Δ*erm* (this work)								

Peptidoglycan/Biomass based on corrected diaminopimelate concentration:	0,145035

Protein

Amino acid	Corrected demand for protein per biomass [g/g]	Demand [mmol/g Protein]
Glycine	0,02279	0,59772
L-alanine	0,02857	0,63120
L-valine	0,02635	0,44284
L-leucine	0,04142	0,62167
L-isoleucine	0,02516	0,37755
L-serine	0,02299	0,43067
L-threonine	0,02665	0,44035
L-phenylalanine	0,02209	0,26329
L-tyrosine	0,01957	0,21261
L-tryptophan	0,00657	0,06332
L-cysteine	0,00613	0,09966
L-methionine	0,00532	0,07019
L-lysine	0,04927	0,65890
L-arginine	0,02441	0,27429
L-histidine	0,00796	0,10099
L-aspartate	0,03218	0,47959
L-glutamate	0,06535	0,88041
L-asparagine	0,03218	0,47953

L-glutamine	0,02181	0,29371
L-proline	0,02112	0,36118
L-selenocysteine	0,00008	0,00094
Sum	0,50800	7,78062

RNA

	Weight per cell [fg]	χ
rRNA	48	0,81356
tRNA	8,6	0,14576
mRNA	2,4	0,04068

rRNA

	Count	χ	Molecular weight [g/mol]	Molecular weight (real) [g/mol]	Mass [g/mol monomer]	Demand [mmol/g RNA]
AMP (ATP)	14915	0,27661	345,21	328,20	90,78370	0,70362
GMP (GTP)	11726	0,21747	361,21	344,20	74,85255	0,55318
CMP (CTP)	15954	0,29588	321,18	304,17	89,99789	0,75264
UMP (UTP)	11326	0,21005	322,17	305,16	64,09889	0,53431
Methylation	360	0,00668	16,04	14,03	0,09365	0,01698
Hydrogenation	24	0,00045		2,02	0,00090	0,00113
	53921				319,82757	2,56186

tRNA

	Count	χ	Molecular weight [g/mol]	Molecular weight (real) [g/mol]	Mass [g/mol monomer]	Demand [mmol/g RNA]
AMP (ATP)	1409	0,19977	345,21	328,20	65,56615	0,08508
GMP (GTP)	1579	0,22388	361,21	344,20	77,05892	0,09535
CMP (CTP)	2134	0,30257	362,21	345,20	104,44679	0,12887
UMP (UTP)	1931	0,27378	363,21	346,20	94,78491	0,11661
Methylation	180	0,02552	16,04	14,03	0,35798	0,01087
Hydrogenation	90	0,01276		2,02	0,02573	0,00543
	7053				342,24047	0,44221

mRNA

	χ	Molecular weight [g/mol]	Molecular weight (real) [g/mol]	Mass [g/mol monomer]	Demand [mmol/g RNA]
AMP (ATP)	0,35459	345,21	328,20	116,37835	0,04524

GMP (GTP)	0,14541	361,21	344,20	50,04967	0,01855
CMP (CTP)	0,14541	321,18	304,17	44,22901	0,01855
UMP (UTP)	0,35459	322,17	305,16	108,20854	0,04524
Methylation	0,00000	16,04	14,03	0,00000	0,00000
Hydrogenation	0,00000		2,02	0,00000	0,00000
				318,86557	0,12757

Abundances of RNA types in *E. coli* (Neidhard *et al.*, 1990)
Methylation and hydrogenation based on Modomics database (Machnicka *et al.*, 2013)
23S rRNA: *E. coli*, 16S rRNA: *C. acetobutylicum*, tRNA: *B.subtilis*
GC contend of genome (unpublished work, Dannheim *et al.*, 2016)

	Demand [mmol/g RNA]
AMP (ATP)	0,83394
GMP (GTP)	0,66708
CMP (CTP)	0,90005
UMP (UTP)	0,69615
Metylation	0,02785
Hydrogenation	0,00657

DNA

	Count	χ	Molecular weight [g/mol]	Molecular weight (real) [g/mol]	Mass [g/mol monomer]	Demand [mmol/g DNA]
dAMP (dATP)	3044566	0,35459	329,21	312,20	110,70487	1,15183
dGMP (dGTP)	1248482	0,14541	345,21	328,20	47,72315	0,47233
dCMP (dCTP)	1248482	0,14541	305,18	288,17	41,90249	0,47233
dTMP (dTTP)	3044566	0,35459	320,20	303,19	107,50999	1,15183
Metylation	8000	0,00093	14,03	12,01	0,01119	0,00303
	8586096				307,85169	

Based on sequence (unpublished work, Dannheim *et al.*, 2016)
Estimated from literature (van Eijk *et al.*, 2015):

Lipid

	Molecular weight [g/mol]
Fatty acid	258,54
Glycerolphosphate	170,06
Glycerol	92,09

Fatty acids

chain length:	16,246
unsaturations:	0,164

	Molecular weight [g/mol]	χ	Demand [mmol/g Lipid]
phosphatidylglycerol	722,17	0,5	0,69236
cardiolipin	1352,24	0,5	0,36976

Fatty acids of *C. difficile* NCIB 10666 (Elsden *et al.*, 1980):

Only phosphatidylglycerol-analogs could be found in *C. difficile* isolates (Drucker *et al.*, 1996)

Lipoteichoic acid

	χ	Molecular weight [g/mol]	Molecular weight (real) [g/mol]	Mass [g/mol monomer]
N-Acetyl-D-Glucosamine	10,14804	221,21	203,20	2062,03
Deacetylation	1,22221	60,05	-42,04	-51,38
Glycerate	5,07402	105,07	87,06	441,72
Glucose	3,00000	180,16	162,15	486,44
1,2-diacyl-sn-glycerol	1,00000	575,16	575,16	575,16
Phosphate	5,07402	95,98	78,97	400,71
				3914,68

	Demand [mmol/g Lipoteichoic acid]
UDP-N-acetyl-D-Glucosamine	2,59231
Deacetylation	0,31221
2-phospho-D-glycerate	1,29615
UDP-Glucose	0,76635
1,2-diacyl-sn-glycerol	0,25545
ATP	1,29615

Composition and chain length in *C. difficile* 630 (Reid *et al.*, 2012)

Average chain length from integration of NMR data: 4,07

Peptidoglycan

	χ	Molecular weight [g/mol]	Molecular weight (real) [g/mol]	Mass [g/mol monomer]	Demand [mmol/g Peptidoglycan]
L-alanine	1,00000	89,09	71,08	71,08	1,17720
D-glutamate	1,00000	146,12	128,11	128,11	1,17720
Diaminopimelate	0,99654	190,20	172,19	171,59	1,17312
D-alanine	0,61085	89,09	71,08	43,42	0,71910
Glycine	0,04532	75,07	57,05	2,59	0,05335
Deacetylation	0,92783	59,04	-42,04	-39,00	1,09224

	χ	Molecular weight [g/mol]	Molecular weight (real) [g/mol]	Mass [g/mol monomer]	Demand
Other modifications	0,31933		-18,02	-5,75	0,37591
N-Acetyl-D-Glucosamine	1,00000	221,21	203,20	203,20	1,17720
N-Acetylmuramic acid	1,00000	292,27	274,26	274,26	1,17720
				849,47	

Peptidoglycan composition of *C. difficile* 630 (Peltier *et al.*, 2011)

Polysaccharide II

	χ	Molecular weight [g/mol]	Molecular weight (real) [g/mol]	Mass [g/mol monomer]	Demand [mmol/g Polysaccharide II]
N-Acetyl-D-Galactosamine	2	221,21	203,20	406,39	1,76376
Glucose	3	180,16	162,15	486,44	2,64564
Mannose	1	180,16	162,15	162,15	0,88188
Phosphate	1	95,98	78,97	78,97	0,88188
				1133,94	

Structure of polysaccharide II (Ganeshapillai *et al.*, 2008):
[→6)-β-D-Glcp-(1→3)-β-D-GalpNAc-(1→4)-α-D-Glcp-(1→4)-[β-D-Glcp-(1→]-β-D-GalpNAc-(1→3)-α-D-Manp-(1→P]

Glycogen

Demand [mmol/g Glycogen]
6,16732

Ions

	χ	Molecular weight [g/mol]	Mass [g/mol monomer]	mmol/g Ions
K	0,86	39,10	33,62	20,96743
Mg	0,077	24,31	1,87	1,87732
Fe(+3)	0,006	55,85	0,34	0,14628
Ca	0,004	40,08	0,16	0,09752
Phosphate	0,043	95,98	4,13	1,04837
Diphospate	0,005	174,95	0,87	0,12190
Na	0,001	22,99	0,02	0,02438
Fe(+2)	0,001	55,85	0,06	0,02438
Ni	0,001	58,70	0,06	0,02438
Cd	0,001	112,40	0,11	0,02438
Zn	0,001	65,38	0,07	0,02438
	1	745,58	41,02	

B. subtilis (Dauner and Sauer, 2001)

Metabolites

Mass [g/mol molecule]	Ratio	Molecular weight (real) [g/mol]	χ	Mass [g/mol Metabolite]	mmol/g Metabolite
ATP	3,2656	503,15	0,20446	102,87165	0,33818
ADP	0,8190	424,18	0,05128	21,75051	0,08481
AMP	0,2988	345,21	0,01871	6,45802	0,03094
Acetyl-CoA	1,6736	805,54	0,10478	84,40614	0,17332
FAD	0,6438	782,53	0,04031	31,54185	0,06667
NAD	1,6046	662,42	0,10046	66,54807	0,16617
NADH	0,3393	663,43	0,02124	14,09335	0,03514
NADP	0,0042	740,39	0,00026	0,19469	0,00043
NADPH	0,2415	741,39	0,01512	11,20983	0,02501
ppGpp	0,6438	598,12	0,04031	24,10874	0,06667
Tetrahydropteroyltriglutamate	0,6438	699,63	0,04031	28,20036	0,06667
Di-trans,octa-cis-undecaprenyl PP	0,6438	924,25	0,04031	37,25424	0,06667
Thiamin diphosphate	0,6438	422,29	0,04031	17,02147	0,06667
Adenosylcobalamin	0,6438	1579,60	0,04031	63,66978	0,06667
Mg-protoporphyrin	0,6438	582,94	0,04031	23,49687	0,06667
MoO2-molybdopterin	0,6438	519,25	0,04031	20,92969	0,06667
Biotin	0,6438	243,3	0,04031	9,80682	0,06667
Me-Thr-P-GluNAc-FliC	0,6438	279,94	0,04031	11,28379	0,06667
Pyridoxine 5'-phosphate	0,6438	247,14	0,04031	9,96160	0,06667
L-threonylcarbamoyladenylate	0,6438	490,32	0,04031	19,76359	0,06667
	15,9722	12255,02	1,00000	604,57108	

C. acetobutylicum in the acidogenic growth phase (Amador-Noguez *et al.*, 2011)

References

Amador-Noguez D, Brasg IA, Feng X-J, Roquet N, Rabinowitz JD: Metabolome Remodeling during the Acidogenic-Solventogenic Transition in *Clostridium acetobutylicum*. Appl Environ Microbiol 2011, 77:7984–7997.

Dauner M, Sauer U: Stoichiometric growth model for riboflavin-producing *Bacillus subtilis*. Biotechnology and Bioengineering 2001, 76:132–143.

Drucker DB, Wardle HM, Boote V: Phospholipid profiles of *Clostridium difficile*. J Bacteriol 1996, 178:5844–5846.

van Eijk E, Anvar SY, Browne HP, Leung WY, Frank J, Schmitz AM, Roberts AP, Smits WK: Complete genome sequence of the *Clostridium difficile* laboratory strain 630Δ*erm* reveals differences from strain 630, including translocation of the mobile element CTn*5*. BMC Genomics 2015, 16:31.

Elsden SR, Hilton MG, Parsley KR, Self R: The Lipid Fatty Acids of Proteolytic *Clostridia*. J Gen Microbiol 1980, 118:115–123.

Ganeshapillai J, Vinogradov E, Rousseau J, Weese JS, Monteiro MA: *Clostridium difficile* cell-surface polysaccharides composed of pentaglycosyl and hexaglycosyl phosphate repeating units. Carbohydrate Research 2008, 343:703–710.

Heinemann M, Kümmel A, Ruinatscha R, Panke S: In silico genome-scale reconstruction and validation of the *Staphylococcus aureus* metabolic network. Biotechnology and Bioengineering 2005, 92:850–864.

Machnicka, M. A., Milanowska, K., Osman Oglou, O., Purta, E., Kurkowska, M., Olchowik, A., Januszewski, W., Kalinowski, S., Dunin-Horkawicz, S. & other authors. (2013). MODOMICS: a database of RNA modification pathways--2013 update. Nucleic Acids Res 41, D262-267.

Neidhardt FC, Ingraham JL, Schaechter M: Physiology of the Bacterial Cell: A Molecular Approach. Sunderland: Sinauer Associates; 1990.

Peltier J, Courtin P, Meouche IE, Lemée L, Chapot-Chartier M-P, Pons J-L: *Clostridium difficile* Has an Original Peptidoglycan Structure with a High Level of N-Acetylglucosamine Deacetylation and Mainly 3-3 Cross-links. J Biol Chem 2011, 286:29053–29062.

Poxton IR, Cartmill TDI: Immunochemistry of the Cell-Surface Carbohydrate Antigens of *Clostridium difficile*. J Gen Microbiol 1982, 128:1365–1370.

Reid CW, Vinogradov E, Li J, Jarrell HC, Logan SM, Brisson J-R: Structural characterization of surface glycans from *Clostridium difficile*. Carbohydrate Research 2012, 354:65–73.

Table S3: **Fits of the substrate concentrations and OD over time of MDM-cultures with *C. difficile* 630Δ*erm*.**

Function	Parameters		Range	R^2
$OD(t) = A_1 + \frac{A_2 - A_1}{(1+((t_0/t)^h)^s}$	$A_1 = 0.00500$<br>$t_0 = 9.09315$<br>$s = 0.10158$	$A_2 = 0.39989$<br>$h = 12.89192$	0.0 h to 12.0 h	0.99234
$Cys_{rel.}(t) = a + b_1 \cdot t + b_2 \cdot t^2 + b_3 \cdot t^3$	$a = 0.99488$<br>$b_2 = -0.01235$	$b_1 = -0.16893$<br>$b_3 = 0.00231$	0.0 h to 6.67 h	0.92454
$Ile_{rel.}(t) = A_1 + \frac{A_2 - A_1}{(1+e^{(t-t_0)/dt})}$	$A_1 = 0.99257$<br>$t_0 = 5.13433$<br>$span = 0.83222$	$A_2 = 0.14788$<br>$dt = 1.53344$<br>$EC_{50} = 169.75062$	0.0 h to 12.0 h	0.98845
$Leu_{rel.}(t) = a + b \cdot t$	$a = 0.96612$	$b = -0.02369$	0.0 h to 5.25 h	0.48804
$Leu_{rel.}(t) = A_1 \cdot e^{-t/x_1} + y_0$	$y_0 = 0.27023$<br>$x_1 = 1.20063$<br>$\tau = 0.83222$	$A_1 = 35.72743$<br>$k = 0.83289$	5.25 h to 12.0 h	0.87374
$Met_{rel.}(t) = a + b_1 \cdot t + b_2 \cdot t^2 + b_3 \cdot t^3$	$a = 1.01491$<br>$b_2 = -0.07927$	$b_1 = -0.07017$<br>$b_3 = 0.01041$	0.0 h to 4.67 h	0.87616
$Pro_{rel.}(t) = A_1 \cdot e^{-t/x_1} + y_0$	$y_0 = 1.31687$<br>$x_1 = -11.33440$<br>$\tau = -7.85641$	$A_1 = -0.32291$<br>$k = -0.08823$	0.0 h to 5.25 h	0.99559
$Pro_{rel.}(t) = A_1 \cdot e^{-t/x_1} + y_0$	$y_0 = 0.02347$<br>$x_1 = 1.07132$<br>$\tau = 0.74259$	$A_1 = 57.12713$<br>$k = 0.93342$	5.25 h to 12.0 h	0.95409
$Trp_{rel.}(t) = A_1 \cdot e^{-t/x_1} + y_0$	$y_0 = 1.34255$<br>$x_1 = -6.93747$<br>$\tau = -4.70769$	$A_1 = -0.37572$<br>$k = -0.14414$	0.0 h to 8.5 h	0.98919
$Val_{rel.}(t) = a + b \cdot t$	$a = 0.98328$	$b = -0.02910$	0.0 h to 5.25 h	0.84721
$Val_{rel.}(t) = A_1 \cdot e^{-t/x_1} + y_0$	$y_0 = 0.32975$<br>$x_1 = 0.88397$<br>$\tau = 0.61272$	$A_1 = 196.70196$<br>$k = 1.13126$	5.25 h to 12.0 h	0.97966

Table S3 – continued from previous page

Function	Parameters		Range	R^2
$Gluc_{rel.}(t) = a + b_1 \cdot t + b_2 \cdot t^2$	$a = 0.98356$<br>$b_2 = -0.01745$	$b_1 = -0.06647$	0.0 h to 5.25 h	0.99923
$Gluc_{rel.}(t) = A_1 + \frac{A_2 - A_1}{(1+((t_0/t)^h)^s}$	$A_1 = 0.05196$<br>$t_0 = 15.87124$<br>$s = 1225.95789$	$A_2 = 2.17470$<br>$h = -9.82418$	5.25 h to 12.0 h	0.99007

Table S4: **Fits of the substrate concentrations and OD over time of CDMM-cultures with *C. difficile* 630Δ*erm*.**

Function	Parameters		Range	R^2
$OD(t) = A_1 \cdot e^{-t/x_1} + y_0$	$y_0 = -0.04045$<br>$x_1 = -1.04652$<br>$\tau = 0.72539$	$A_1 = 0.04110$<br>$k = 0.955552$	0.0 h to 2.0 h	0.95351
$OD(t) = A_1 \cdot e^{-t/x_1} + y_0$	$y_0 = 0.00000$<br>$x_1 = -1.32201$<br>$\tau = 0.91635$	$A_1 = 0.04757$<br>$k = 0.75642$	1.0 h to 3.4 h	0.89764
$OD(t) = a + b \cdot t$	$a = -0.02090$	$b = 0.19075$	3.5 h to 8.0 h	0.86917
$Cys_{rel.}(t) = A_1 + \frac{A_2 - A_1}{(1+e^{(t-t_0)/dt})}$	$A_1 = 0.96740$<br>$t_0 = 2.03742$<br>$span = 0.86195$	$A_2 = 0.10545$<br>$dt = 0.34962$<br>$EC_{50} = 7.67078$	0.0 h to 3.5 h	0.99883
$Cys_{rel.}(t) = a + b \cdot t$	$a = 0.75591$	$b = -0.18272$	3.5 h to 4.0 h	0.99303
$Ile_{rel.}(t) = A_1 \cdot e^{-t/x_1} + y_0$	$y_0 = 1.12163$<br>$x_1 = -1.58509$<br>$\tau = -1.09870$	$A_1 = -0.10272$<br>$k = -0.63088$	0.0 h to 3.5 h	0.92790
$Ile_{rel.}(t) = a + b \cdot t$	$a = 0.58216$	$b = -0.12008$	3.5 h to 4.5 h	0.76521
$Leu_{rel.}(t) = a + b_1 \cdot t + b_2 \cdot t^2 + b_3 \cdot t^3$	$a = 0.99344$<br>$b_2 = 0.03270$	$b_1 = -0.10645$<br>$b_3 = -0.02375$	0.0 h to 3.5 h	0.96144
$Leu_{rel.}(t) = a + b \cdot t$	$a = 0.31681$	$b = -0.08192$	3.5 h to 3.75 h	-
$Met_{rel.}(t) = A_1 \cdot e^{-t/x_1} + y_0$	$y_0 = 0.94952$<br>$x_1 = -1.17058$<br>$\tau = -0.81139$	$A_1 = -0.03582$<br>$k = -0.85428$	0.0 h to 3.75 h	0.99105
$Pro_{rel.}(t) = A_1 + \frac{A_2 - A_1}{(1+e^{(t-t_0)/dt})}$	$A_1 = 1.31883$<br>$t_0 = 1.75619$<br>$span = 1.59886$	$A_2 = -0.28003$<br>$dt = 1.19337$<br>$EC_{50} = 5.79033$	0.0 h to 3.5 h	0.99472
$Trp_{rel.}(t) = A_1 \cdot e^{-t/x_1} + y_0$	$y_0 = 1.71645$<br>$x_1 = -9.26705$<br>$\tau = -6.42343$	$A_1 = -0.72456$<br>$k = -0.10791$	0.0 h to 5.25 h	0.99083

Table S4 – continued from previous page

Function	Parameters		Range	R^2
$Val_{rel.}(t) = A_1 \cdot e^{-t/x_1} + y_0$	$y_0 = 1.02750$<br>$x_1 = -2.33158$<br>$\tau = -1.61613$	$A_1 = -0.09812$<br>$k = -0.42889$	0.0 h to 5.25 h	0.99051
$Ala_{rel.}(t) = A_1 + \frac{A_2 - A_1}{(1+e^{(t-t_0)/dt})}$	$A_1 = 0.96241$<br>$t_0 = 2.44879$<br>$span = 0.63071$	$A_2 = 0.33169$<br>$dt = 0.41349$<br>$EC_{50} = 11.17042$	0.0 h to 3.5 h	0.96921
$Ala_{rel.}(t) = a + b_1 \cdot t + b_2 \cdot t^2$	$a = -1.07653$<br>$b_2 = -0.10834$	$b_1 = 0.80434$	3.5 h to 5.25 h	0.93296
$Arg_{rel.}(t) = A_1 + \frac{A_2 - A_1}{(1+e^{(t-t_0)/dt})}$	$A_1 = 1.05930$<br>$t_0 = 2.41832$<br>$span = 0.69836$	$A_2 = 0.36094$<br>$dt = 0.74592$<br>$EC_{50} = 11.22696$	0.0 h to 11.0 h	0.99115
$Asp_{rel.}(t) = A_1 + \frac{A_2 - A_1}{(1+e^{(t-t_0)/dt})}$	$A_1 = 0.99783$<br>$t_0 = 2.06163$<br>$span = 0.60847$	$A_2 = 0.38936$<br>$dt = 0.25586$<br>$EC_{50} = 7.85877$	0.0 h to 3.5 h	0.99395
$Asp_{rel.}(t) = A_1 \cdot e^{-t/x_1} + y_0$	$y_0 = 0.17968$<br>$x_1 = 0.43935$<br>$\tau = 0.30453$	$A_1 = 619.04429$<br>$k = 2.27611$	3.5 h to 5.25 h	0.97811
$Gly_{rel.}(t) = A_1 \cdot e^{-t/x_1} + y_0$	$y_0 = 1.34096$<br>$x_1 = -2.87096$<br>$\tau = -1.99000$	$A_1 = -0.32757$<br>$k = -0.34832$	3.5 h to 4.0 h	0.99942
$Phe_{rel.}(t) = A_1 \cdot e^{-t/x_1} + y_0$	$y_0 = 1.11495$<br>$x_1 = -1.62541$<br>$\tau = -1.12651$	$A_1 = -0.09174$<br>$k = -0.61530$	0.0 h to 4.0 h	0.99718
$Ser_{rel.}(t) = A_1 + \frac{A_2 - A_1}{(1+e^{(t-t_0)/dt})}$	$A_1 = 0.95887$<br>$t_0 = 2.83070$<br>$span = 0.99020$	$A_2 = -0.03133$<br>$dt = 0.23137$<br>$EC_{50} = 16.95729$	0.0 h to 3.5 h	0.99801
$Ser_{rel.}(t) = a + b \cdot t$	$a = 0.13832$	$b = -0.03361$	3.5 h to 3.75 h	-
$Thr_{rel.}(t) = A_1 \cdot e^{-t/x_1} + y_0$	$y_0 = 2.57581$<br>$x_1 = -9.94436$	$A_1 = -1.49028$<br>$k = -0.10056$	0.0 h to 5.25 h	0.96896

Table S4 – continued from previous page

Function	Parameters		Range	R^2
	$\tau = -6.89291$			
$Tyr_{rel.}(t) = A_1 \cdot e^{-t/x_1} + y_0$	$y_0 = 1.39161$<br>$x_1 = -4.42492$<br>$\tau = -3.06712$	$A_1 = -0.39354$<br>$k = -0.22599$	0.0 h to 4.5 h	0.96196
$His_{rel.}(t) = A_1 + \frac{A_2 - A_1}{(1+e^{(t-t_0)/dt})}$	$A_1 = 1.00560$<br>$t_0 = 2.90486$<br>$span = 0.76985$	$A_2 = 0.23575$<br>$dt = 0.75226$<br>$EC_{50} = 18.26266$	0.0 h to 11.0 h	0.99096
$Gluc_{rel.}(t) = a + b \cdot t$	$a = 0.99484$	$b = -0.02424$	0.0 h to 3.0 h	0.69519
$Gluc_{rel.}(t) = A_1 + \frac{A_2 - A_1}{(1+e^{(t-t_0)/dt})}$	$A_1 = 0.95438$<br>$t_0 = 4.06080$<br>$span = 0.86008$	$A_2 = 0.09430$<br>$dt = 0.35287$<br>$EC_{50} = 58.02097$	1.5 h to 5.5 h	0.99589
